# Spatiotemporal disparity of breast cancer incidence in Iranian female populations at the district level from 2000 to 2021: Bayesian disease mapping

**DOI:** 10.1371/journal.pone.0330017

**Published:** 2025-09-11

**Authors:** Shadi Rahimzadeh, James Bentham, Beata Burczynska, Farshad Farzadfar, Mariachiara Di Cesare

**Affiliations:** 1 Institute of Public Health and Wellbeing, University of Essex, Colchester, United Kingdom; 2 School of Mathematics, Statistics and Physics, Newcastle University, Newcastle upon Tyne, United Kingdom; 3 Department of Natural Science, School of Science and Technology, Middlesex University, London, United Kingdom; 4 Non-Communicable Diseases Research Centre, Endocrinology and Metabolism Population Sciences Institute, Tehran University of Medical Sciences, Tehran, Iran; 5 Endocrinology and Metabolism Research Centre, Endocrinology and Metabolism Clinical Sciences Institute, Tehran University of Medical Sciences, Tehran, Iran; King Faisal University, SAUDI ARABIA

## Abstract

**Introduction:**

While trends in breast cancer incidence in Iran are generally monitored at the national level, little is known about subnational variations in these trends. This study aimed to assess the levels and trends (2000–2021) of the relative risk (RR) of breast cancer incidence at the district level in Iran and its relation to key socioeconomic dimensions to understand the full extent of geographical and social inequalities in the country associated with breast cancer morbidity.

**Methods:**

District-level incidence data by age and sex from the National Cancer Registry System of the Iranian Ministry of Health were used. Related covariates were extracted from the Census and Household Expenditure and Income Survey (HEIS) datasets. The RR of breast cancer incidence was estimated in women above the age of 30 for all 316 districts in Iran from 2000 to 2010 using a Bayesian spatiotemporal model. Finally, predictions were estimated for the period 2011–2021.

**Results:**

The national RR of breast cancer incidence in Iran increased from 0.21 (95% credible interval (CrI): 0.19, 0.22) in 2000 to 0.66 (0.63, 0.68) in 2010 and 1.23 (1.18, 1.28) in 2021. The RR of breast cancer incidence was highest in Yazd (1.96 [1.63, 2.33]), Shiraz (1.90 [1.72, 2.09]) and Shemiranat (1.90 [1.12, 2.91]) in 2010 and in Tehran (3.99 [3.86, 4.33]), Bushehr (3.89 [3.07, 4.77]) and Abadan (3.67 [2.99, 4.39]) in 2021. In contrast, Savojbolagh, Saravan and Nikshahr had the lowest RRs in both 2010 (0.11 [0.05, 0.20], 0.17 [0.08, 0.30] and 0.20 [0.09, 0.36], respectively) and 2021 (0.19 [0.10, 0.33], 0.34 [0.18, 0.54] and 0.35 [0.17, 0.62], respectively). The RR of breast cancer incidence was 60% greater across districts in the highest YOS quintile (average years of schooling: 3.9) than in those in the lowest YOS quintile (average years of schooling: 2.2; relative index of inequality: 1.6).

**Conclusions:**

The results show that the RR of breast cancer incidence has increased over time (2000–2021) at the national and subnational levels in Iran. Breast cancer is one of the few diseases with a positive education gradient, with a greater RR of breast cancer incidence among higher-educated women than among lower-educated women. However, this is likely due to better awareness of diagnostic approaches and access to those approaches rather than reflecting patterns in the true incidence of breast cancer. While social inequalities are a major barrier to reducing the prevalence and incidence of breast cancer, it is important to track the progress made at the district level based on the characteristics of specific policies aimed at reducing health inequalities. A scaling-up in the quality of healthcare services, national and subnational policies addressing prevention and treatment, and more specialised training programmes for women’s health are needed.

## Introduction

Currently, accelerating the pace of progress in cancer diagnosis and treatment is crucial, given the COVID-19 pandemic, which has led to health care provision interruptions worldwide [1–3]. In 2020, female breast cancer was the leading cause of global cancer incidence, with an estimated 2.3 million new cases, representing 11.7% of all cancer diagnoses [4], making it one of the most severely burdensome cancers globally [4,5]. Among women, breast cancer accounts for 1 in 4 cancer cases and 1 in 6 cancer deaths, ranking first in incidence (159 of 185 countries) and mortality (110 of 185 countries) [4].

To address the increasing global burden of cancer, in 2021, the World Health Organization (WHO) established the Global Breast Cancer Initiative (GBCI), with the aim of reducing global breast cancer mortality by 2.5% per year until 2040 via early detection, timely diagnosis, and comprehensive breast cancer management [6,7].

While breast cancer incidence and mortality trends are generally monitored at the national level [8,9], little is known about the distribution and variation of breast cancer at the subnational level. Considering geographical variability in breast cancer incidence across small areas and over time is essential to facilitate appropriate subnational allocation of resources to increase breast cancer screening and achieve better outcomes [10,11]. Improved geographic resource allocation, especially in low- and middle-income settings where resources are scarce, could maximise the impact of screening interventions and possibly reduce geographic variability in mortality [10].

In Iran, breast cancer is the most common cancer and the fifth most common cause of death among females [12]. In 2019, the age-standardised breast cancer incidence rate was 34.1 per 100,000 women (30.7, 37.9), while the age-standardised death rate was 11.9 deaths per 100,000 women (10.8, 13.1), with percentage changes of 71.2 (27.9, 118.3) and 15.5 (−3.7, 31.7), respectively, between 1990 and 2019 [13]. Current trends highlight the need for robust estimates of geographical and temporal patterns to support effective public health policies in a country characterised by insufficient financial resources and a lack of continuity of financing [14]. Here, we estimated the relative risk (RR) of breast cancer incidence in women aged 30 years or older in all 316 districts (S1 Table, S1 Fig) in Iran from 2000 to 2021 and assessed the correlation between breast cancer incidence and socioeconomic status at the district level.

## Methods

### Data

The data used in the present study primarily came from two sources: the National Cancer Registry System of Iran (CRS) (from 2000 to 2010, excluding 2006), which has been used to retrieve breast cancer data, and the Census and Household Expenditure and Income Survey (HEIS) (from 2000 to 2010), which has been used to retrieve population data and covariates. The selection of covariates, including female mean years of schooling, urbanisation, wealth index, and cancer registry completeness, was based on their availability, relevance to breast cancer risk factors, and contribution to model fit as assessed by the Deviance Information Criterion (DIC), ensuring balance between data-driven estimation and model stability. Detailed information on these data sources is given elsewhere [15]. All data were provided at the aggregated district-year level without missing values. Any data cleaning or imputation for missingness was conducted by the original data custodians before aggregation. Several health system components, including the number of health centres/units, number of specialist physicians (consultants), number of general physicians (GPs), number of pharmacies, number of beds in hospitals, number of intensive care units (ICUs), number of nurses and number of community health workers (Behvarzes) per 100,000 people at the province level, have also been used to assess their correlation with the predicted incidence of breast cancer in 2020.

### Statistical analysis

When some populations at risk are small or the disease is rather rare, RRs may be unreliable [16]. Spatiotemporal modelling allows the borrowing of information from neighbouring areas and years, providing estimates for locations and years with little or no data [17]. In this study, a Bayesian Poisson spatiotemporal model was fitted to model the log-risk estimates using a combination of spatially structured and unstructured random effects [18] and covariates. The female mean years of schooling (YOS), female urbanisation, wealth index (WI) and completeness percentage of the cancer registry were used as covariates. We assumed that the number of incident breast cancer cases in each district-year follows a Poisson distribution. The spatiotemporal random effects included both spatially structured and unstructured components. The spatially structured random effect was modelled using a conditional autoregressive (CAR) prior to account for spatial correlation between neighbouring districts, whereas the unstructured component was assigned an independent and identically distributed normal prior. Temporal random effects were modelled assuming exchangeability across years without explicit temporal structure. Independence between the spatially structured and unstructured effects was assumed. It was assumed that the covariates had fixed effects. We examined the relationship between the standardised morbidity ratio (SMR) against the RR of breast cancer incidence for 316 districts. SMRs represent the observed number of cases divided by the expected number of cases [19], which were calculated using an indirect age-standardisation technique. The expected number of incidence cases was estimated by multiplying the mean national rate for Iran in 2010 by the age-specific population for each district-age group.

To assess the probability that each district’s RR exceeded the national RR, we compared the posterior samples of each district to those of the national estimate. For each district we obtained 20,000 posterior samples using Markov Chain Monte Carlo (MCMC) methods. After discarding the initial 5,000 iterations as a burn-in period we used the remaining 10,000 iterations from 2 chains for the analysis (combined to form 20,000 posterior samples). At each iteration, we assessed whether the district’s RR was higher or lower than the national RR. We repeated this process for the 20,000 iterations, and calculated the probability as the proportion of iterations where the district RR exceeded the national RR. Additionally, we used 20,000 posterior samples to estimate the national rates for each year and to project district-level rates from 2011 onward.

Given the absence of data beyond 2011, projecting future trends was necessary. To address this issue, we employed projection methods that leverage the power of Bayesian spatiotemporal modelling while accounting for uncertainty. Our approach involved incorporating the uncertainty from the Bayesian spatiotemporal model to make robust and reliable predictions. The first step in our projection methodology was to derive the RR estimates for each district from the Bayesian spatiotemporal model. From this model, we generated a posterior distribution of RR using two chains. To capture the full range of uncertainty, we drew a large sample of 20,000 values from the posterior distribution for each district. Next, we apply the projection model, which takes each sampled RR value as input. This allowed us to fit multiple projection models, each based on a different sampled value, and capture the variations in the projected outcomes. By running the projection model for each sampled value, we accounted appropriately for the uncertainties in the RR estimates. To obtain a comprehensive understanding of the projected outcomes, we computed summary statistics for the combined projection models. These summary statistics provided a concise representation of the projected trends while encapsulating the variability arising from the uncertainty in the RR estimates.

To examine the association between the predicted RR of breast cancer incidence and several health system indicators in 2020, a Pearson correlation test was used. Specifically, the total number of GPs, total number of pharmacies, total number of ICUs, total number of nurses, total number of specialists, total number of beds in hospitals of medical universities, and total number of health centres and community health workers (Behvarzes) were available for only one year (2020) at the province level. P values less than 0.05 were considered significant [20].

### Model specification

A spatiotemporal model was applied to the district-level data, which incorporated both spatial fitting and temporal considerations (S1 Appendix). The model is specified as follows:


$$Y_{i,t}\sim \text{Poisson}(E_{i,t}\lambda_{i,t})$$

$$log\left(\lambda_{i,t}\right)=\alpha +\beta_{yos}{YOS}_{i,t}+\beta_{urb}{URB}_{i,t}+\beta_{comp}{COMP}_{i,t}+U_{i}+V_{i}+X_{t}+\varepsilon_{i,t}$$

where *i* represents districts, *t* corresponds to years, *Y(i,t)* represents breast cancer incidence by district and year, *E(i,t)* represents the expected number of cases by district and year, *λ(i,t)* represents the RR parameters by district and year, *α* is the intercept, *YOS* is the mean female years of schooling, *URB* is the female urbanisation percentage, *COMP* is the cancer registry completeness percentage, *U*_*i*_ is a spatially structured random term, *V*_*i*_ is an unstructured random term, *X*_*t*_ is a temporal random term, *ε(i,t)* represents residuals by district and year and the *β* vector comprises the spatiotemporal coefficients corresponding to *YOS*, *URB* and *COMP* indicating the change in λ associated with change in *YOS*, *URB* and *COMP* by district and year.

The projection model applied in this study is specified as follows:


$$R_{i,j}=b_{0_{i,j}}+b_{1_{i,j}}X$$

where *i* represents districts (i = 1,2,..., 316), *j* corresponds to the number of posterior samples (j = 1,2,...,20000), *R*_*i j*_ is each posterior sample of RR of breast cancer incidence for each district, *X* is the year, and *b* is a vector of regression coefficients.

The model was fitted using OpenBUGS version 3.2.3 with the Markov chain Monte Carlo (MCMC) algorithm, accessed through the R2OpenBUGS package in R for Windows version 4.2.1 (http://www.r-project.org/) and STATA software (version 11.0). The objective was to estimate the RR of breast cancer incidence by district and year, including the 2.5th and 97.5th percentiles of the posterior distribution, which represent the lower and upper bounds of the 95% credible intervals (CrIs).

### Cross-validation

The validity of the model estimates was assessed using cross-validation. Ten percent of the data was withheld, and the model was fitted using the remaining 90% of the district-level data. Predictions were then generated for the held-out district-years and compared to the actual observed values. Model performance was evaluated using the median relative error, median absolute relative error, median error and median absolute error. In addition, the Bayesian Wilcoxon rank-sum test was used to assess whether the differences between predicted and observed values were statistically significant [21]. The coverage of the 95% CrIs was examined to evaluate the uncertainty in the estimates. A model with good external predictive validity, would have 95% of the held-out value falling within the 95% CrIs of the predicted estimates. In addition, formal diagnostic methods including the Brooks-Gelman-Rubin (R-hat) statistic and the Deviance Information Criterion (DIC) were used to assess model convergence and goodness-of-fit, as described in detail in S2 Appendix (S7 Fig, S2 Table).

### Ethics

For this study, aggregated (by age, sex, province/district) anonymised data were accessed on 11/05/2020 and used for this analysis. Ethical approval was obtained through the Ethics Committee of Middlesex University in the UK (14142.2020) on 11/05/2020 as part of a PhD project. Approval was based on the ethical approval obtained through the Ethics Committee of the National Institute for Medical Research Development in Iran (IR.NIMAD.REC.1396.192) on 10/12/2017 by FF in their role as Director of the Non-Communicable Disease Research Centre (NCDRC), Tehran University of Medical Science. No form of consent was requested because access was limited to the aggregated anonymised data. All authors had access to the data. Consent to participate is “Not applicable”.

## Results

### Bayesian spatiotemporal findings for relative risk of breast cancer incidence

**Fig 1:**
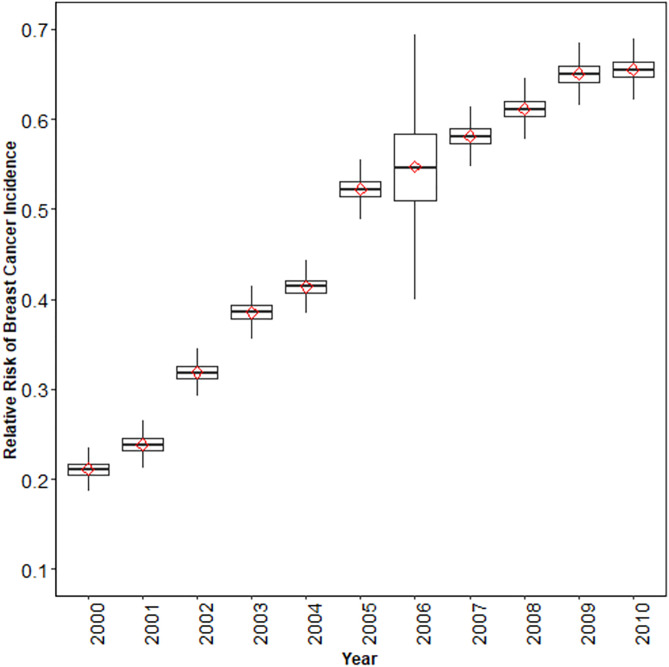
National average for RR of breast cancer incidence by year out of all posterior samples (red diamonds show the mean value). The absence of empirical data in 2006 leads to greater uncertainty in the model estimates for that year, resulting in wider credible intervals compared to years with available registry data.

Among the 316 districts, there were 1095 new breast cancer cases in 2000, with the most in Isfahan (16.8% of all new cases) and 7960 in 2010, with the most in Tehran (25.4%). The national average RR of breast cancer increased from 0.21 (95% CrI: 0.19, 0.22) in 2000 to 0.52 (0.49, 0.54) in 2005 and 0.66 (0.63, 0.68) in 2010 (Fig 1). The analysis at the district level implied having more sparse data and geographical areas with no available data, especially in 2000. In S2 Fig, the estimated RR of breast cancer incidence resulting from the spatiotemporal model (left side) was compared with the raw data (right side) for all years. The comparison shows how the model can smoothly interpolate for districts with missing data. In addition, since there were no data available for 2006, the model could make estimates for all districts for 2006 consistent with earlier and later years.

The three districts with the highest RR of breast cancer incidence in 2000 were Isfahan (1.14 [0.98, 1.31]), Kashan (1.02 [0.71, 1.40]) and Ahvaz (0.74 [0.57, 0.92]) while Savojbolagh (0.05 [0.02, 0.11]), Tehran (0.06 [0.04, 0.08]) and Saravan (0.08 [0.03, 0.16]) had the lowest RRs in 2000. The RR of breast cancer incidence was highest in Yazd (1.96 [1.63, 2.33]), Shiraz (1.90 [1.72, 2.09]) and Shemiranat (1.90 [1.12, 2.91]) in 2010. In contrast, Savojbolagh (0.11 [0.05, 0.20]), Saravan (0.17 [0.08, 0.30]) and Nikshahr (0.20 [0.09, 0.36]) had the lowest RRs in 2010 (S3 Table).

**Fig 2:**
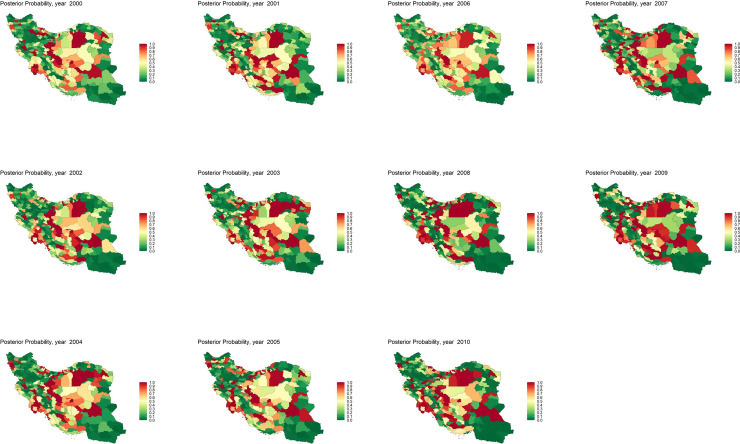
Maps of the posterior probability for each district in which the RR exceeded the national RR by year from 2000 to 2010. (National RR by year: 2000 = 0.21, 2001 = 0.23, 2002 = 0.31, 2003 = 0.38, 2004 = 0.41, 2005 = 0.52, 2006 = 0.55, 2007 = 0.58, 2008 = 0.61, 2009 = 0.65, 2010 = 0.66. Full national RR values with 95% credible intervals are reported in S4 Table).

In addition, the posterior probability of RR for each district being greater than the national RR with values between 0 and 1 were plotted on the map of districts by year (Fig 2). In 2000, with a national mean of 0.21 (0.95% CrI: 0.19, 0.22), there were 94 districts out of 316 in which the posterior probability was more than 50% compared to 121 districts in 2010 with a national mean of 0.66 (0.63, 0.68).

### Breast cancer estimations in relation to years of schooling and wealth index

**Fig 3:**
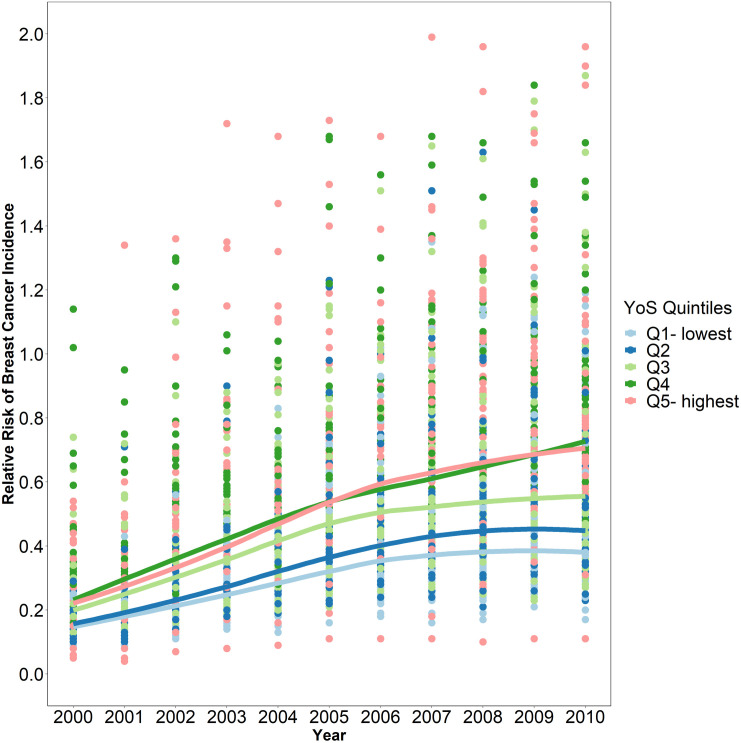
RR of breast cancer incidence categorised by YOS quintiles over time (each point represents a district). Light blue indicates the lowest YOS quintile, and pink indicates the highest YOS quintile.

The average RR of breast cancer incidence increased with the YOS quintile, ranging from 0.15 in the lowest quintile to 0.24 in the highest quintile for 2000 and from 0.48 to 0.79 for 2010. The RR of breast cancer incidence was 60% greater across districts in the highest YOS quintile (average years of schooling: 3.9) than in those in the lowest YOS quintile (average years of schooling: 2.2; relative index of inequality: 1.6). Fig 3 shows that while the RR of breast cancer increased over time, it also increased according to the YOS quintile. The districts in the highest quintile had a greater RR than districts in the lower quintiles. In addition, S3 Fig confirms that the RR of breast cancer incidence increased more quickly according to the mean YOS in recent years than in earlier years. However, the RR slightly changed with increasing mean YOS over Q4 (S3 Fig). The same results were also observed for the WI quintiles; districts in the highest WI quintiles had a greater RR of breast cancer incidence over time (S4 Fig).

### Projections of the relative risk of breast cancer incidence between 2011 and 2021

**Fig 4:**
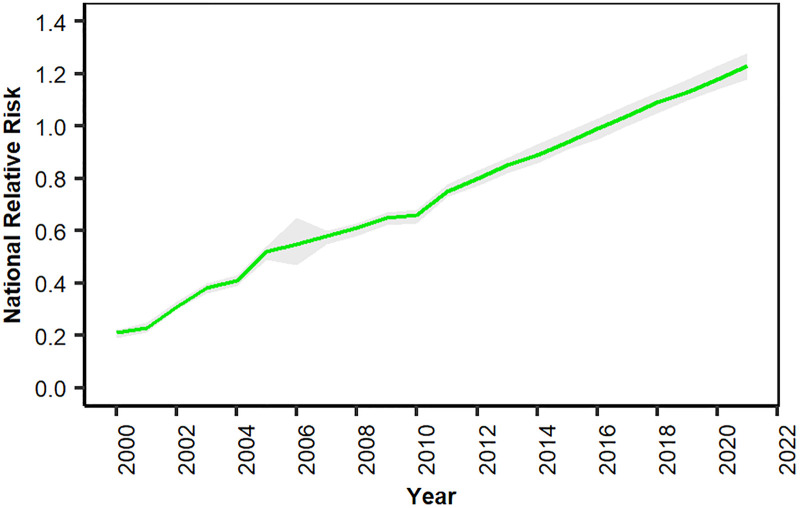
Trend in national RR of breast cancer incidence by year from 2000 to 2021 (grey shading shows the 95% credible interval). The absence of empirical data in 2006 leads to greater uncertainty in the model estimates for that year, resulting in wider credible intervals compared to years with available registry data. The three leading districts with the highest RRs were Tehran (3.99 [95% CrI: 3.86, 4.33]), Bushehr (3.89 [3.07, 4.77]) and Abadan (3.67 [2.99, 4.39]) in 2021. In contrast, Savojbolagh (0.19 [0.10, 0.33]), Saravan (0.34 [0.18, 0.54]) and Nikshahr (0.35 [0.17, 0.62]) had the lowest RRs in 2021 (S5 Table).

If post-2010 trends continue, the RR of breast cancer incidence will reach a value of 1.23 in 2021, almost two times greater than the 2010 level (0.66) (Fig 4, S4 Table). S5 Fig shows how the RR of breast cancer changed from 2011 to 2021 based on the posterior values and uncertainty resulting from the spatiotemporal model for earlier years. The trend results for each district are also presented in S6 Fig.

### Correlation of the RR of breast cancer incidence with health system components in 2020

The RR of breast cancer incidence was associated with multiple components of the health system. The 2020 projected RR of breast cancer was significantly positively correlated with the total number of GPs (r = 0.43, p = 0.01), the total number of pharmacies (r = 0.42, p = 0.01), the total number of ICUs (r = 0.44, p = 0.01) and the total number of nurses (r = 0.43, p = 0.01). While there was also a significant positive correlation between the RR of breast cancer and the total number of specialists (r = 0.50, p = 0.001) and the number of beds in medical university hospitals (r = 0.48, p = 0.001), no significant correlation was detected between the number of health centres (r = 0.03, p = 0.83) and the number of community health workers (Behvarzes) (r = 0.06, p = 0.69). The obtained results confirm that the presence of more health system components may contribute to more effective detection and increased incidence of breast cancer.

### Cross-validation results for the spatiotemporal model at the district level

The results of the cross-validation are presented in S6 Table. The estimates of the RR of breast cancer incidence were unbiased, as evidenced by the median relative errors being very close to zero ranged from −0.10 to 0.24, and the median absolute relative error ranged from 0.22 to 0.51 across the years 2000–2010, indicating good predictive performance of the model. The median errors for the estimated RRs were also small and ranged between −0.24 (2001) and 0.22 (2010). The mean difference between the held-out values and estimates was assessed using the Bayesian Wilcoxon rank test, and the corresponding credible intervals were all nonsignificant (the distributions included zero). The cross-validation therefore confirmed that the statistical model used to estimate the RR of breast cancer incidence at the district level fit well. The 95% CrIs of the estimated RRs covered more than 86% to 100% of the observed values from 2000–2010.

## Discussion

The current study investigated the RR of breast cancer incidence across 316 districts in Iran from 2000 to 2021. The results showed a substantial increase in the incidence of breast cancer nationally and sub nationally and high levels of heterogeneity at the subnational level in Iran.

When attempting to compare the results with those of previous studies, no studies were found to directly compare the RR of breast cancer incidence at the district level per year. This work revealed that all districts had increasing trends in the RR of breast cancer incidence from 2000 to 2021. The corresponding estimates from this study were in line with those of studies on province-level estimations [15,22]. However, here, we present for the first time the RR of breast cancer at the district level over the years, which can be instrumental in obtaining localised insights, optimising healthcare planning, identifying high-risk areas, investigating environmental factors, addressing treatment disparities, and promoting public health awareness [23]. The increase in incidence could be attributable to improvements in the national cancer registry system, resulting in an increase in the number of patients registered [22]. Upgrading diagnostic tools, more extensive healthcare coverage, awareness among the general population of breast cancer symptoms, and greater readiness to undergo screening despite cultural barriers were all possible factors that contributed to the increase in breast cancer incidence [22]. On the other hand, potential lifestyle risk factors, including increased fat intake, smoking and low physical activity (especially in postmenopausal women), could explain the increase in breast cancer incidence observed among Iranian women [24–26]. In addition, changes in cultural habits, such as increased age at childbearing and, in particular, older age at first pregnancy, can partly explain the observed trends [27–29]. However, the specific effect of the factors mentioned above on breast cancer incidence is still controversial and needs further investigation [22].

Our results suggest high levels of geographical heterogeneity in breast cancer incidence across Iranian districts. Previous research shows that cancer incidence in Asian countries has a positive correlation with the country’s level of development, as measured by the Human Development Index (HDI) [30]. In Iran, a direct and substantial association was also found between the incidence of breast cancer and HDI [31]. These findings align with our results in which higher levels of breast cancer incidence are observed across districts with higher levels of female education and wealth. This could be explained by increasing life expectancy, urbanisation, greater exposure to risk factors, delayed childbearing, and a greater rate of screening resulting from higher socioeconomic status [32].

Considering the significance of the healthcare system, extensive research has demonstrated a compelling association between delays in the health system and an elevated likelihood of advanced-stage breast cancer diagnosis in patients [33,34]. Addressing this issue requires the implementation of potential solutions aimed at improving diagnosis timing and, consequently, the clinical stages at which breast cancer is detected. These solutions should encompass strategies targeting the general population, healthcare professionals, and the healthcare system itself [33].

Disparities in breast cancer outcomes can result from modifiable social and health system determinants, such as poor access to care, a lack of health education, a lack of financial resources, challenging patient‐provider interactions, and structural barriers within the health system itself [35], which can guide policymakers in identifying strategies to more equally distribute clinical expertise and health infrastructure across populations [35]. Similarly, our results showed a significant positive association between breast cancer incidence and health workforce size (the number of nurses, GPs, and consultants) and between breast cancer incidence and service delivery (the number of beds in hospitals, the number of pharmacies and the number of ICUs) as components of the health system. The higher quality of the health system may allow earlier detection, leading to an increased number of cases, earlier treatment, and better overall patient outcomes [36].

Our results emphasise the high heterogeneity across districts, confirming the need for a comprehensive and practical plan to control breast cancer that considers subnational variability and a call to improve data collection for breast cancer surveillance in the country. These differences emphasise the crucial need to improve access to diagnosis and treatment facilities in the most deprived areas and reduce inequalities through a stratified resource allocation approach. The observed heterogeneity can reflect lower access, knowledge and acceptance of screening in underprivileged and smaller areas compared to large cities such as Tehran, which have multiple diagnostic facilities, cancer specialists, and healthcare coverage, in addition to higher educational levels of patients, leading to increased screening participation [37].

The Iranian health system has invested in policies focused on the domains of primary and secondary care, training healthcare professionals, and research to reach universal health coverage [38]; however, international sanctions against Iran from 2011 forced some restrictions on all these efforts [39] affecting all aspects of the health system in monitoring morbidity and mortality including access to essential cancer medications and radiotherapy services, which may influence breast cancer incidence patterns seen in this study. Sanctions have led to shortages of modern and effective cancer treatments, forcing oncologists to rely on older, less effective drugs [40]. In addition, difficulties in importing and maintaining radiotherapy equipment have disrupted timely cancer care [41]. These challenges could reduce survival rates and affect registry-based incidence estimates, as survival-dependent prevalence may vary across regions. Therefore, the observed geographic differences in breast cancer incidence may partly reflect the healthcare system limitations caused by sanctions. Policymakers should consider how sanctions indirectly had critical adverse effects on the Iranian population’s health by diminishing access to diagnostic and treatment facilities, specifically for those with cancers [42]. Eventually, the COVID-19 pandemic and its convergence with socioeconomic challenges, economic burdens, and the heavy burden of noncommunicable disease in Iran could directly and indirectly affect the health system for major diseases, especially in the most deprived areas [43].

Our results are based on a model that has accommodated spatial and temporal dependencies that could account for similarities in unobservable factors in neighbouring districts and years. Our specification of space-time interaction allowed each district to have its own temporal pattern. Therefore, this specification allowed us to provide the most comprehensive analysis of breast cancer incidence in Iran at the subnational level to date. The research covers a substantial period (2000–2021), with estimates available for 316 districts in Iran that have not been analysed and published before.

## Limitations

First, we presented estimates at the district level for breast cancer incidence only since no mortality data were available at the district level. Modelling mortality data at a smaller spatial level could yield detailed information regarding mortality trends in the country and critical information for prioritising resource allocation. We also lacked access to tumor stage data at the district level, which limited our ability to assess potential surveillance bias versus etiological effects in the observed education gradient. Second, a Pearson correlation test was used to examine the association of breast cancer incidence with several health system components. During this project, these components were only available for one year (2020) and limited to provincial level. A multivariate analysis using more comprehensive data for the health system over the years and possibly for smaller areas (i.e., district level) is warranted to provide further insight into these associations. In addition, most of the cancer registry data in Iran are pathology-based, which regularly provides beneficial information to improve cancer care systems, especially in low- and middle-income settings where there are no comprehensive population-based cancer registries [44]. However, pathology-based registries may underestimate the true incidence due to underdiagnosis in specific areas or subgroups of the population (e.g., urban areas). Access to alternative data sources or linkage to hospital records was not possible due to data availability. While this limitation may introduce some bias in the RR estimates, cancer registries remain the most consistent and comparable data source at subnational level. In addition, despite the use of national cancer registry data, the availability of data remains limited and sparse leading to potential bias in the estimates. In this regard, we developed a Bayesian Poisson model that allowed us to impute the gaps in the data and improve the estimate where the data were available.

Despite these limitations, our study fills a critical gap in subnational cancer surveillance literature, especially in low- and middle-income settings, where the evidence remains limited. Additionally, it highlights the geographical distribution of underserved populations where enhanced screening programs are essential to support the World Health Organization’s Global Breast Cancer Initiative (GBCI) goal of reducing age-standardized breast cancer mortality rates by 2.5% annually through 2040 [6].

## Conclusions

To the best of our knowledge, this study is the first subnational-level analysis of the RR of breast cancer incidence in Iran that focuses on the district level over time and simultaneously uses several administrative datasets and Bayesian spatiotemporal modelling to obtain district-level estimations between 2000 and 2021 and addresses the incompleteness of the cancer registry. At the same time, with increasing incidence, a lower incidence was observed in the most deprived districts, possibly due to underdiagnosis or late-stage diagnosis. On the other hand, the incidence of breast cancer was much greater in districts with higher levels of female education than in those with the lowest level of female education.

## Supporting information

S1 Fig Map of Iran by district. (PNG)

S2 Fig Estimated RR of breast cancer incidence (left side) versus SMR (right side) at the district level from 2000 to 2010. Cancer registry data were not available in 2006, so the SMR map for 2006 is unavailable.
(PDF)

S3 Fig National average RR of breast cancer incidence categorised by year and YOS quintiles (each point represents a year). (PNG)

S4 Fig RR of breast cancer incidence categorised by wealth index quintiles from 2000 to 2010 (each point represents a district). Light blue shows the lowest WI quintile, and pink shows the highest WI quintile).
(PNG)

S5 Fig Projected RR of breast cancer incidence at the district level from 2011 to 2021. (PDF)

S6 Fig Trend of RR of breast cancer incidence for each district from 2000–2021 in Iran (spatiotemporal model for 2000–2010 and projection for 2011–2021). The grey shading shows the 95% credible intervals.
(PDF)

S7 Fig BGR diagnostics. (PDF)

S1 Table List of provinces and districts in Iran. (DOCX)

S1 Appendix Spatiotemporal model in OpenBUGS. (DOCX)

S2 Appendix Model convergence and goodness of fit. (DOCX)

S2 Table R-hat index for the model-specific parameters to check model convergence. (DOCX)

S3 Table Posterior mean and 95% credible intervals for the RR of breast cancer incidence by district and year (sorted by mean values in 2010). (DOCX)

S4 Table National average RR of breast cancer incidence from 2000 to 2021 (based on all posterior samples). (DOCX)

S5 Table Posterior mean and 95% credible intervals for the RR of breast cancer incidence by district and year (sorted by mean values in 2021). (DOCX)

S6 Table Results of cross-validation for the spatiotemporal model at the district level for RR of breast cancer incidence. (DOCX)

S1 Data Aggregated data. (PDF)
