# Supplementary figures and images for: Spatiotemporal disparity of breast cancer incidence in Iranian female populations at the district level from 2000 to 2021: Bayesian disease mapping
Source: PLoS One. 2025 Sep 11;20(9):e0330017. doi: 10.1371/journal.pone.0330017 (PMC12425319; doi:10.1371/journal.pone.0330017)

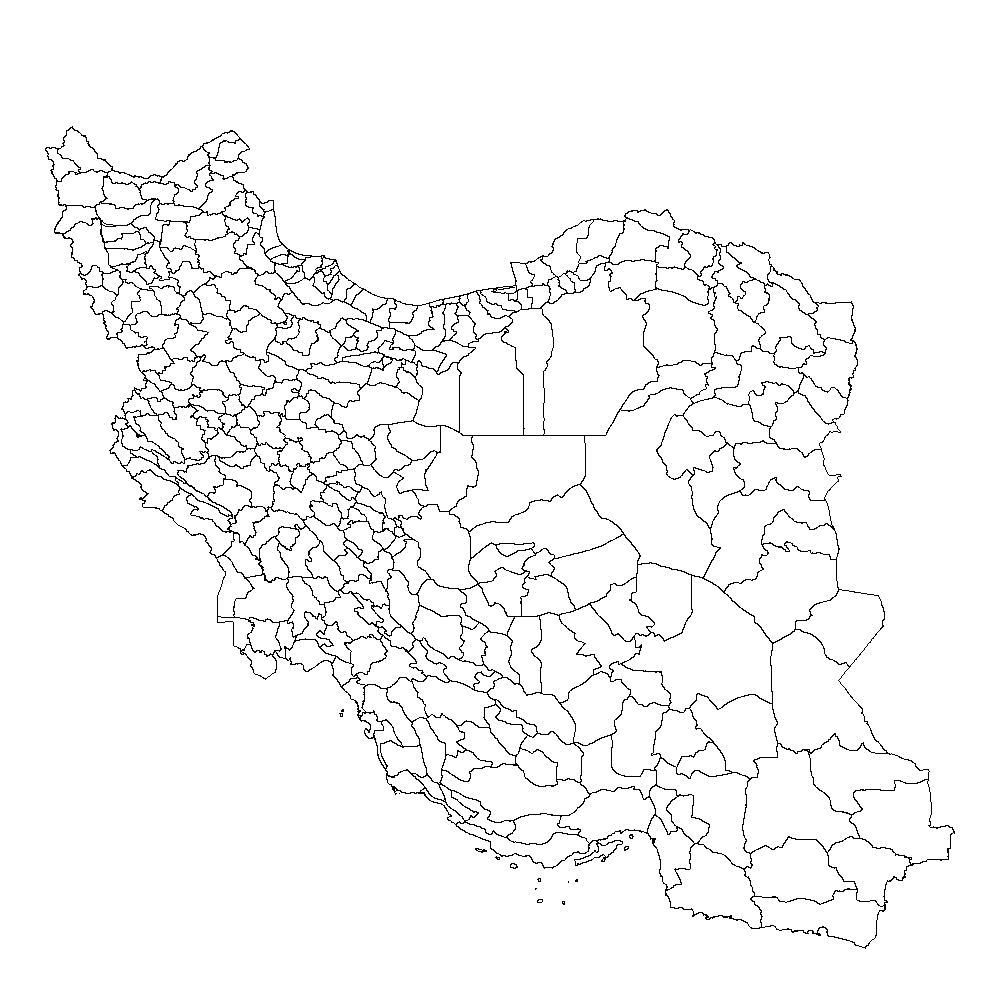

Supplement: S1 Fig — (PNG) [file pone.0330017.s001.png]

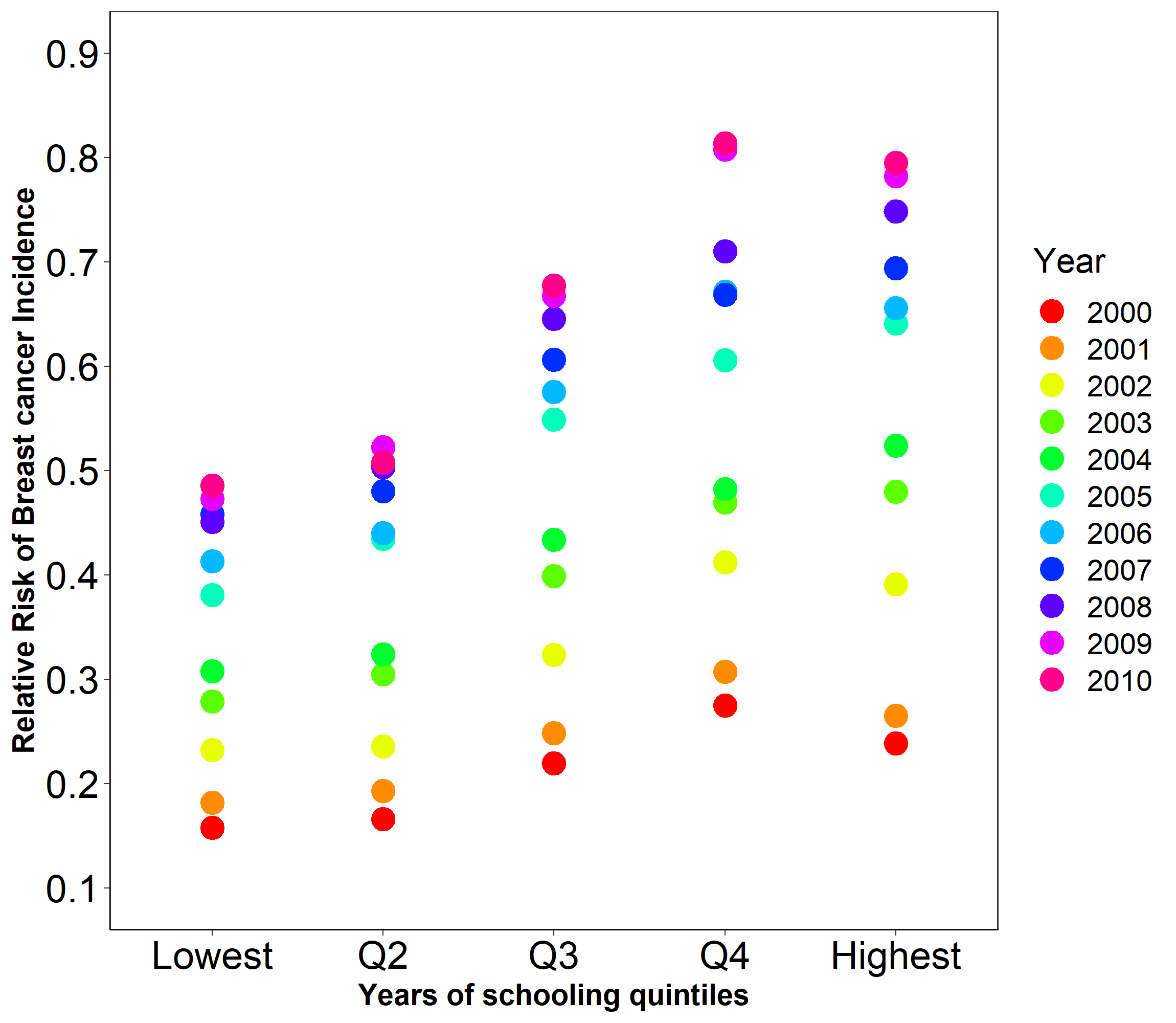

Supplement: S3 Fig — (PNG) [file pone.0330017.s003.png]

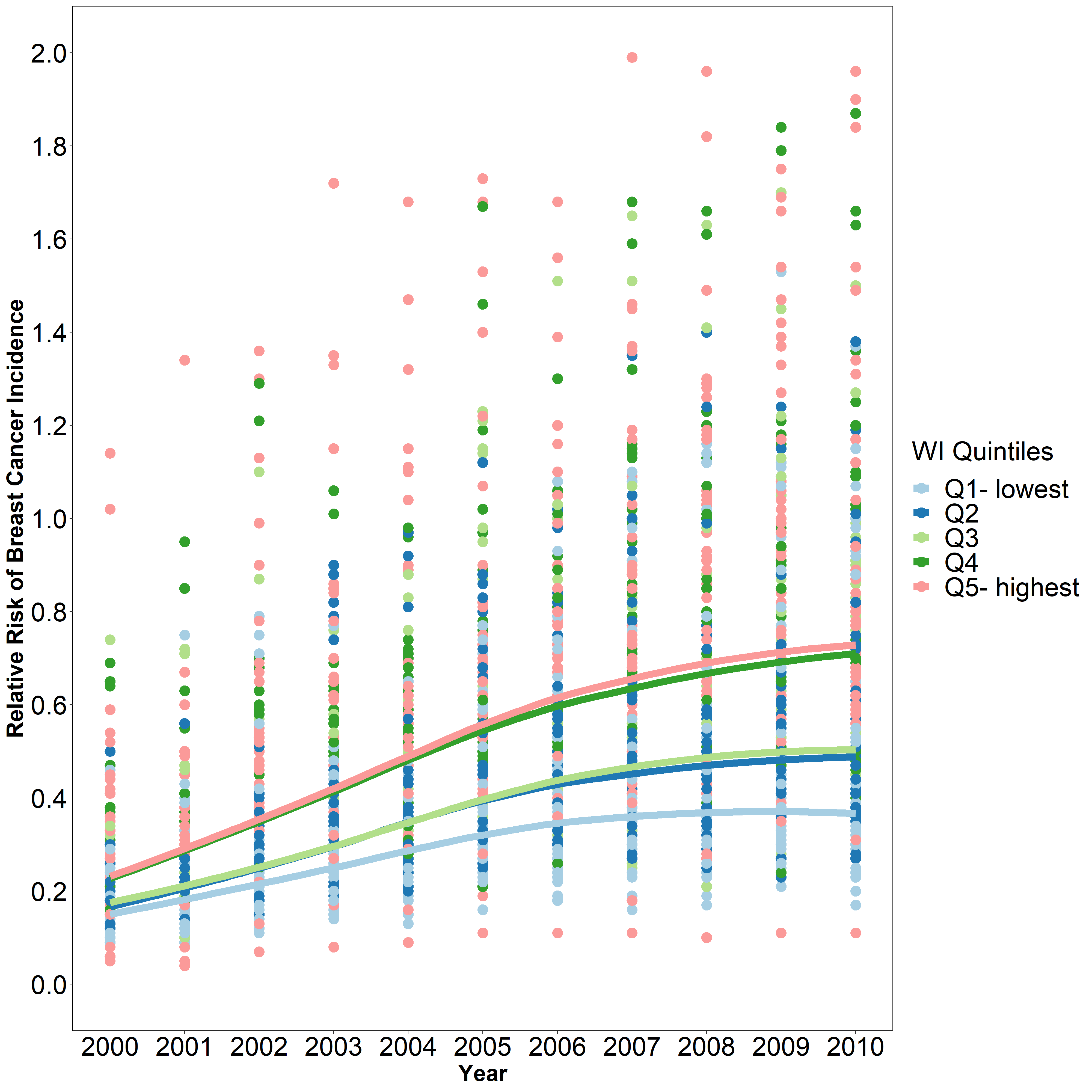

Supplement: S4 Fig — Light blue shows the lowest WI quintile, and pink shows the highest WI quintile). (PNG) [file pone.0330017.s004.png]

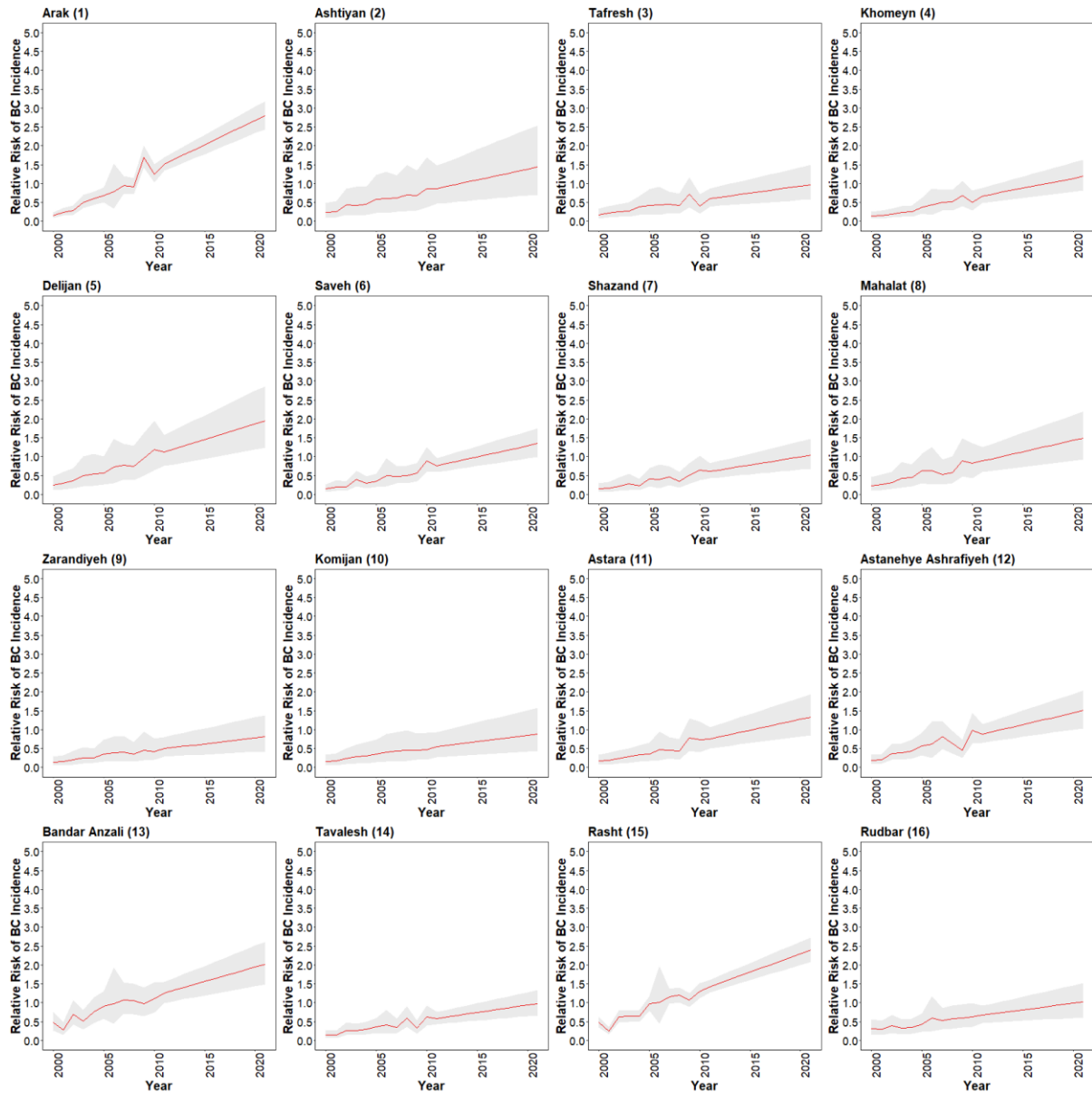

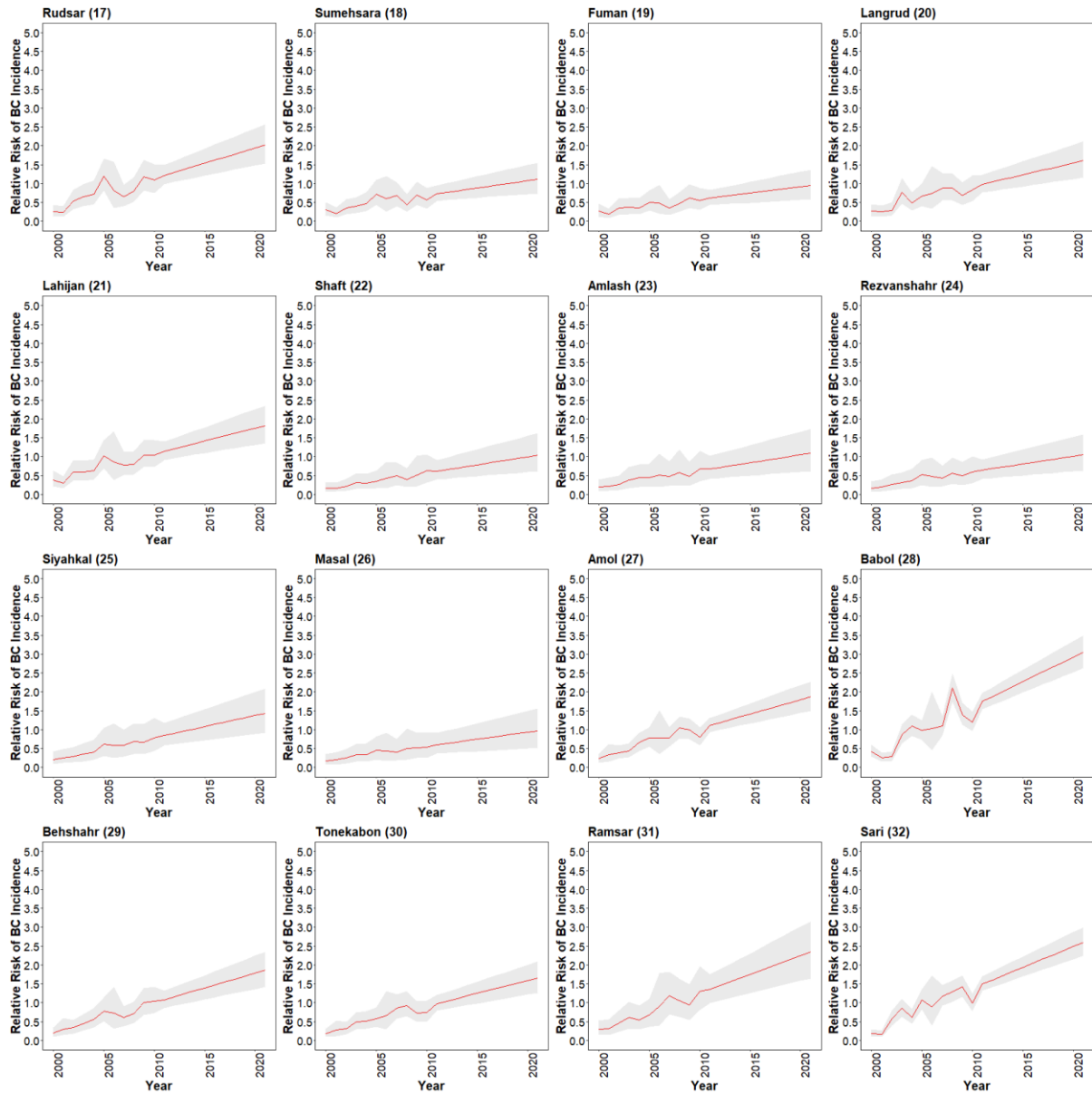

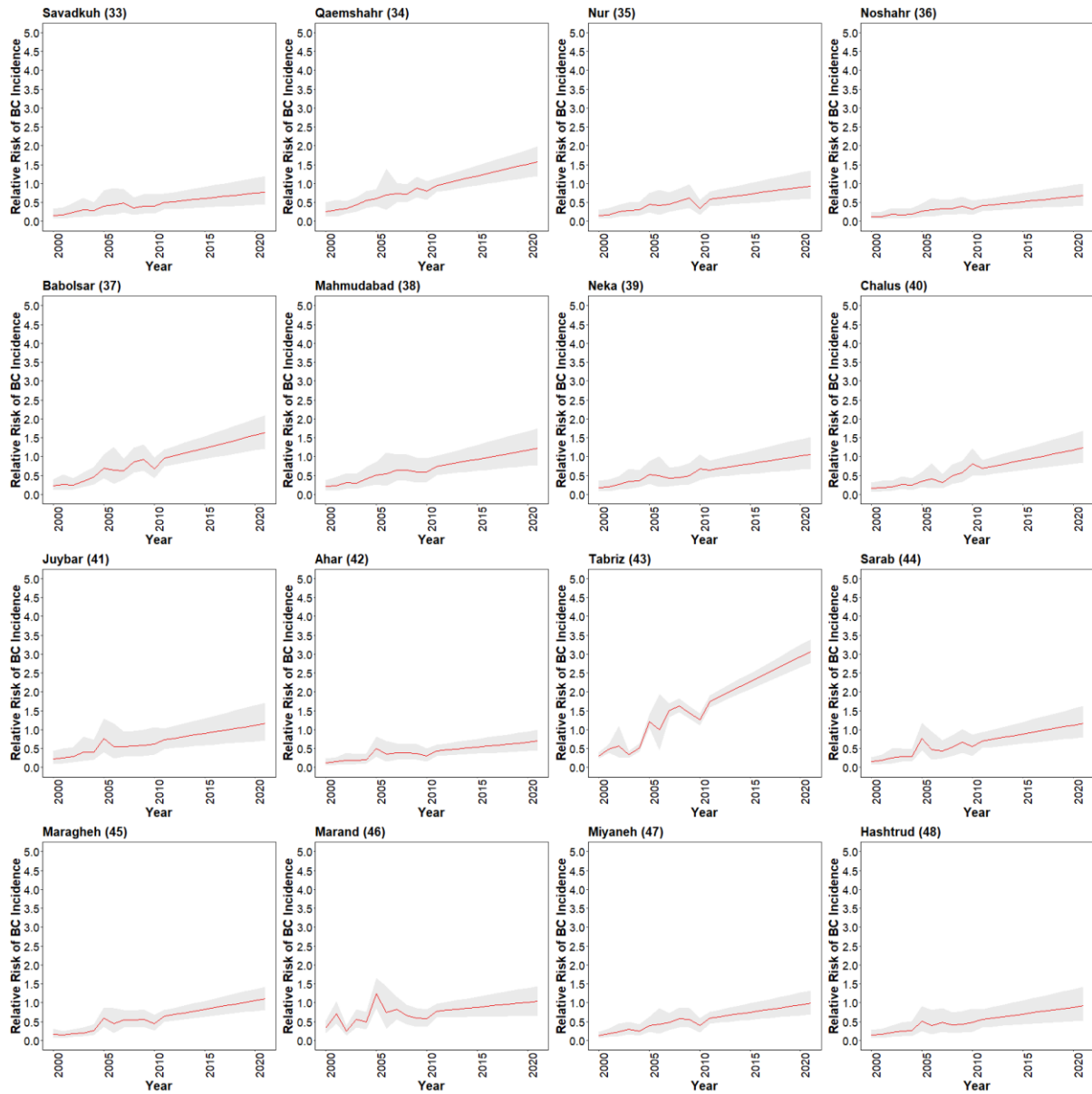

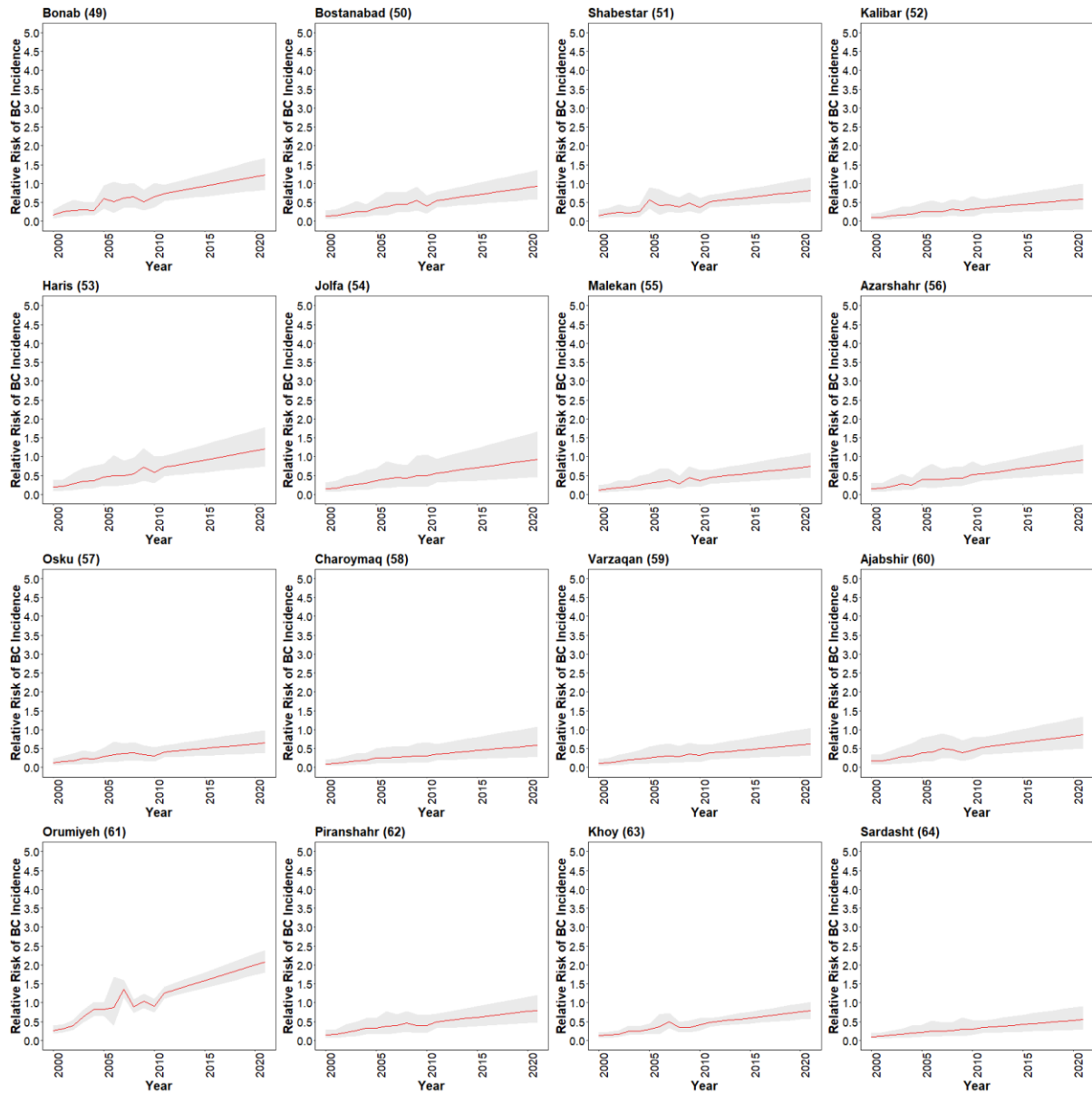

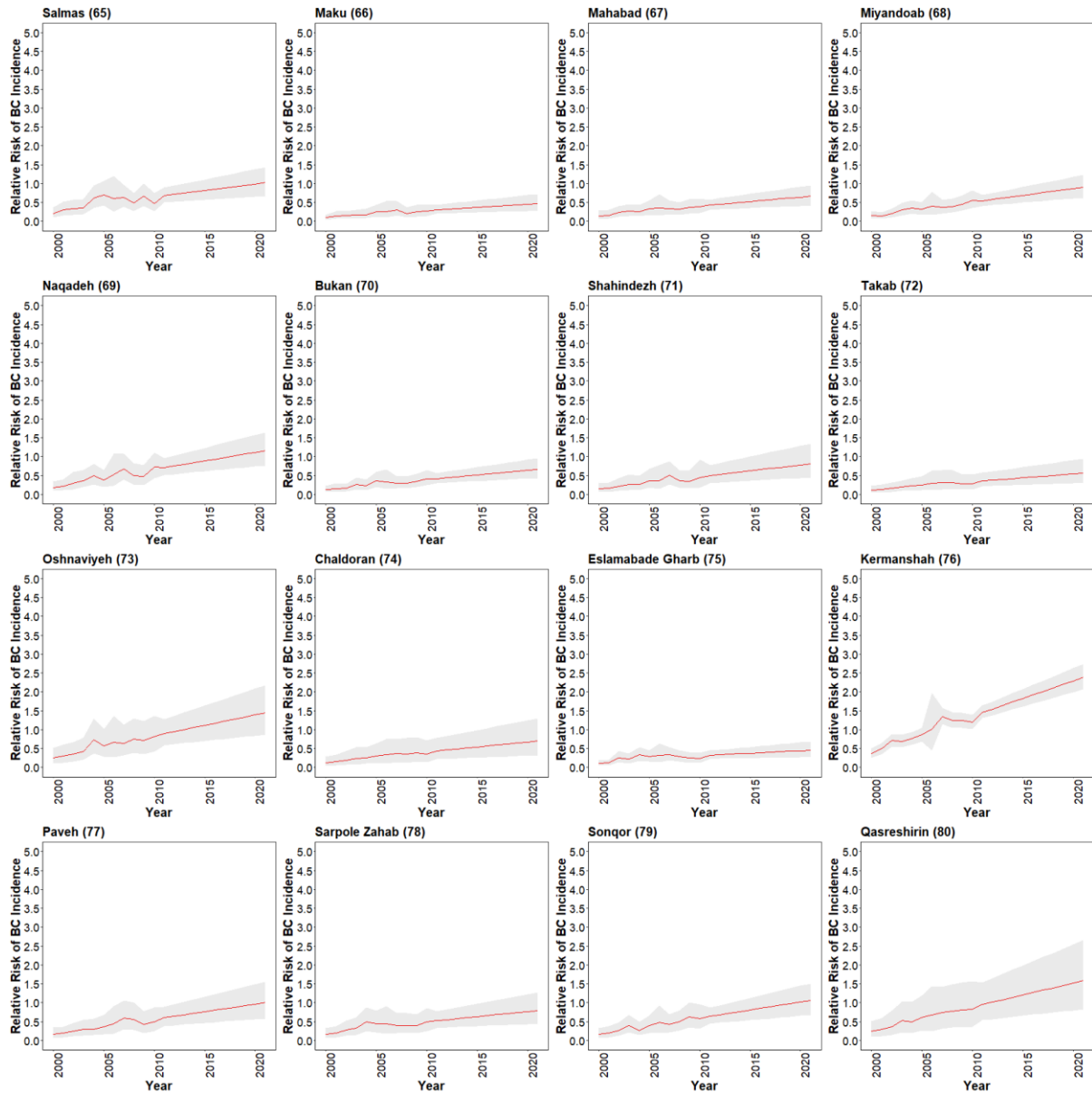

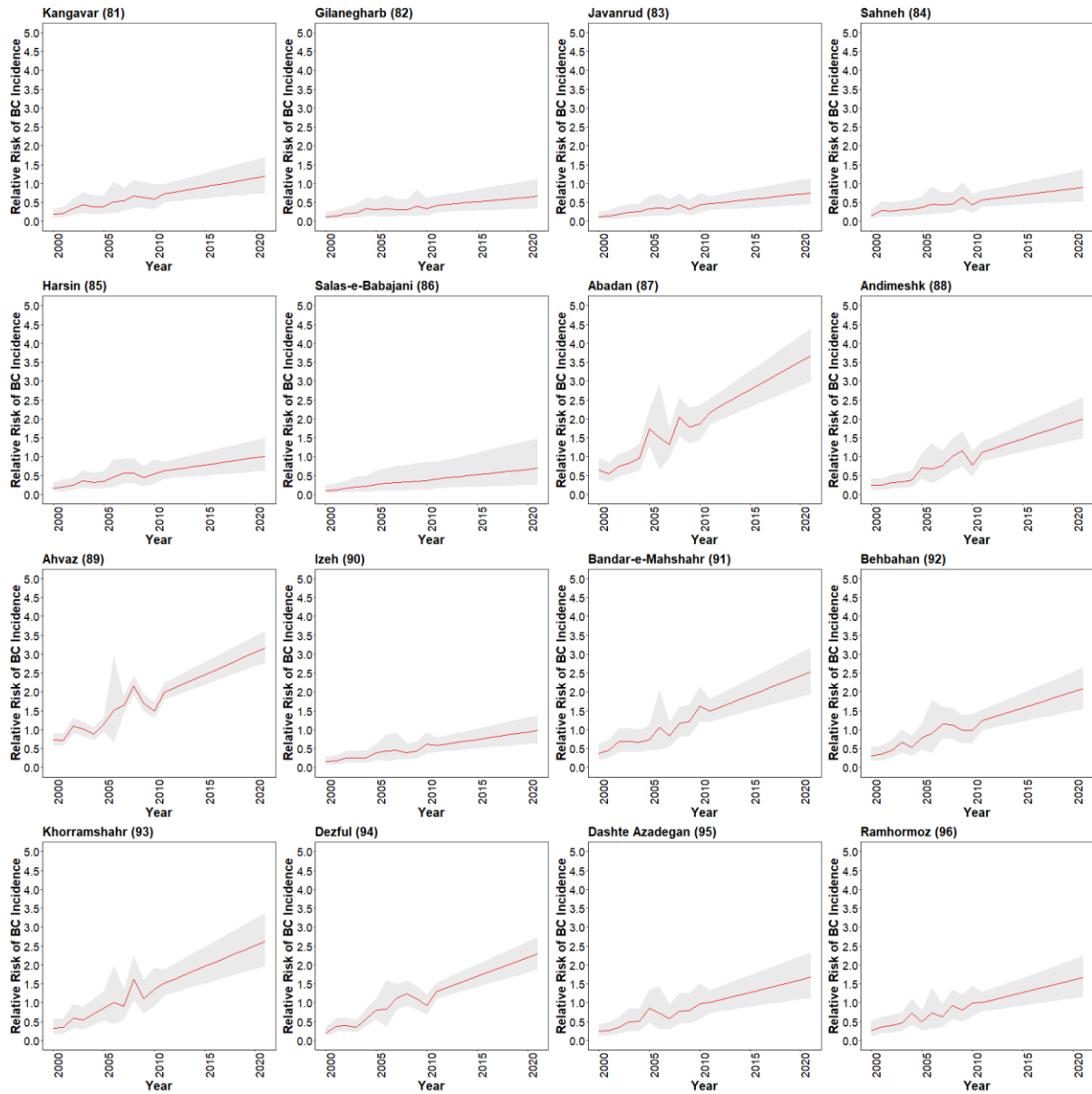

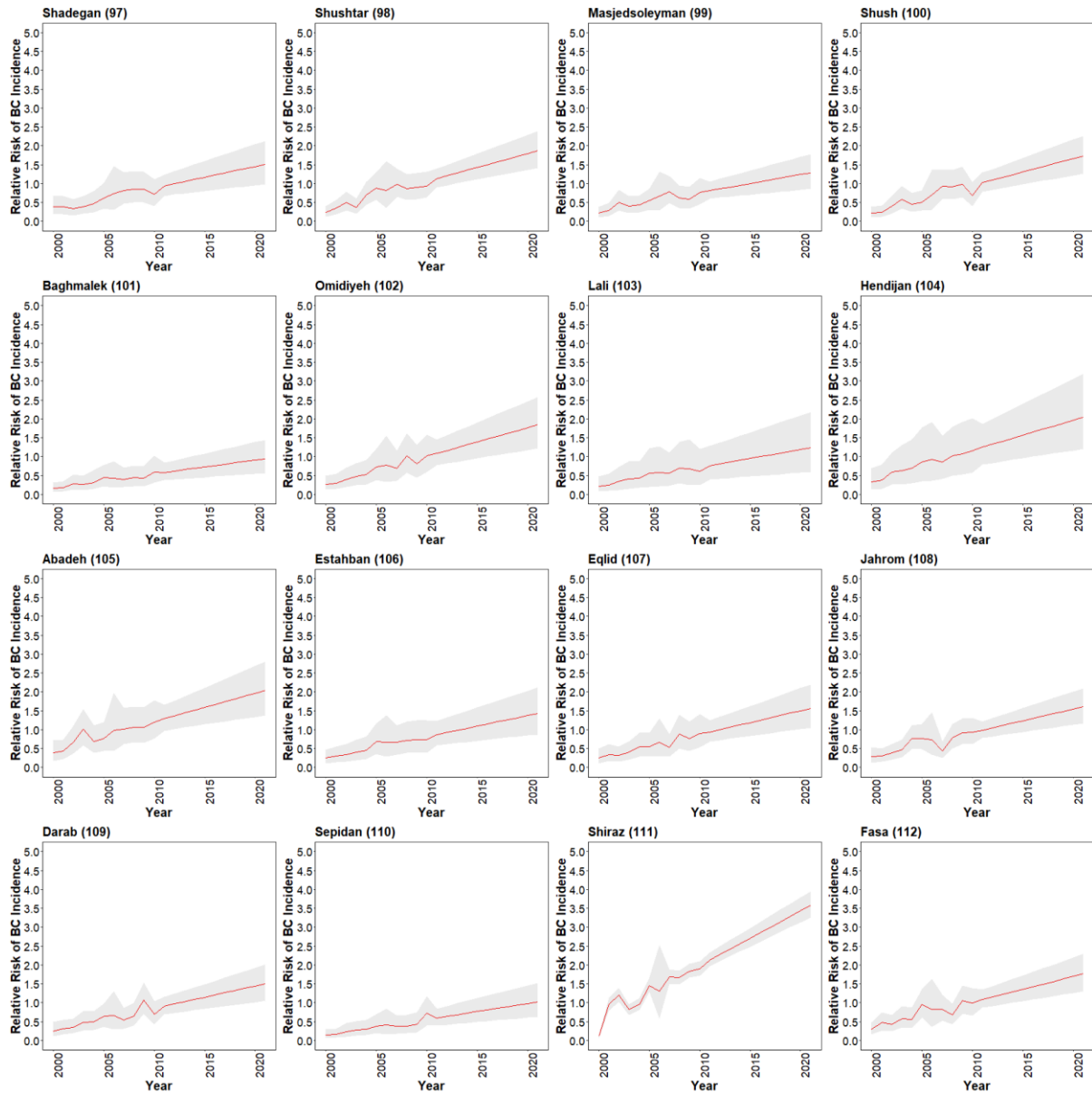

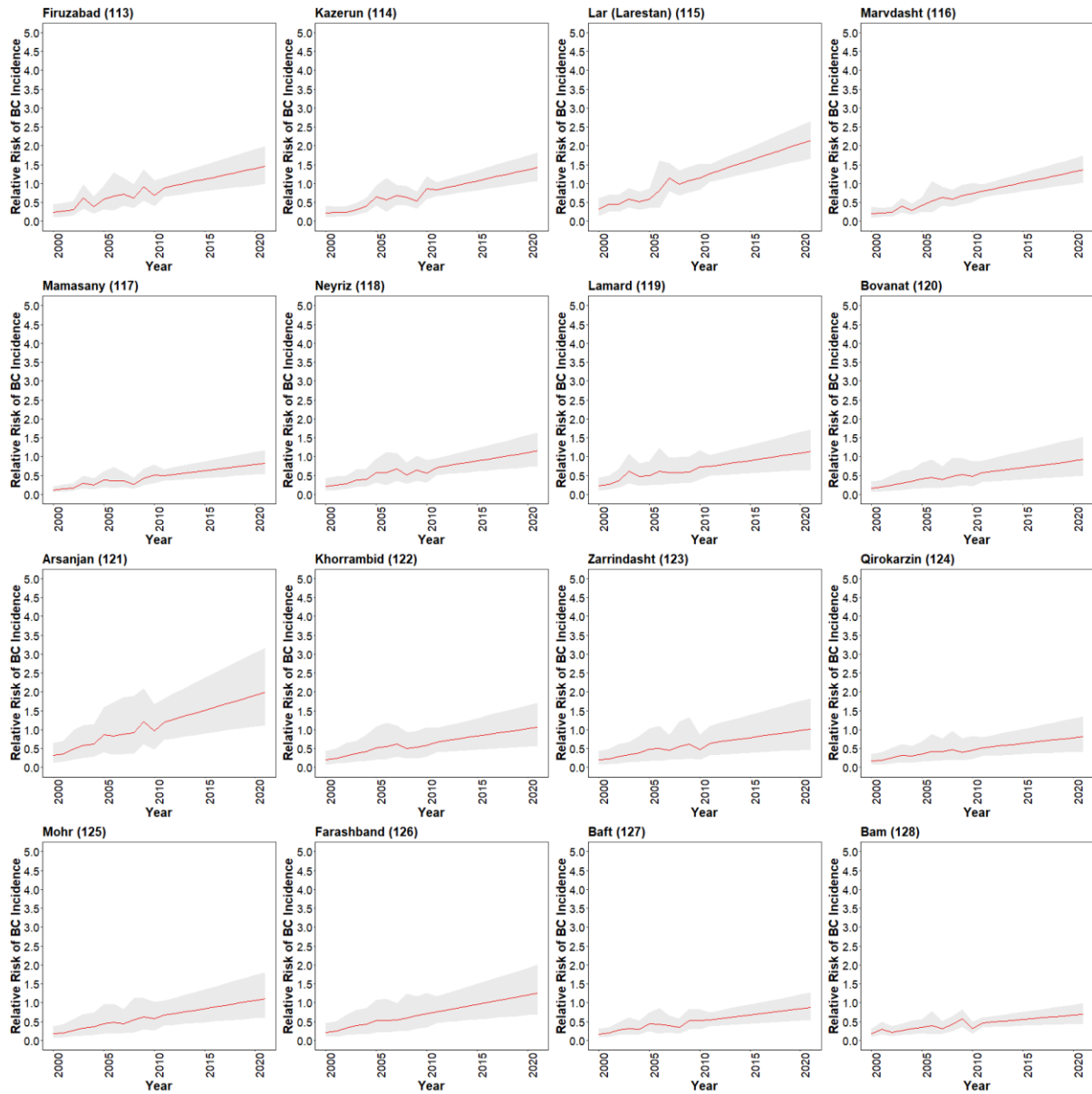

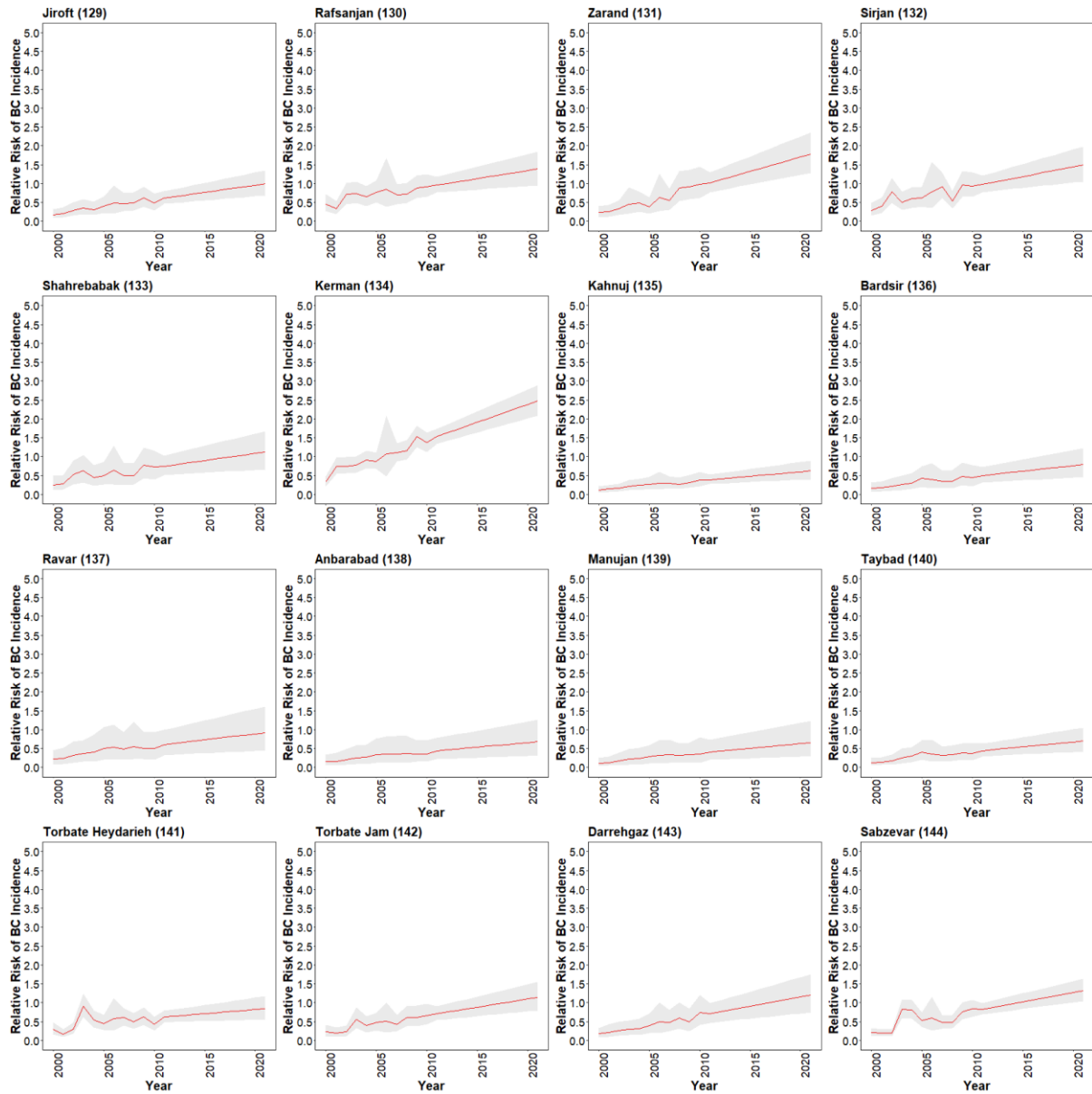

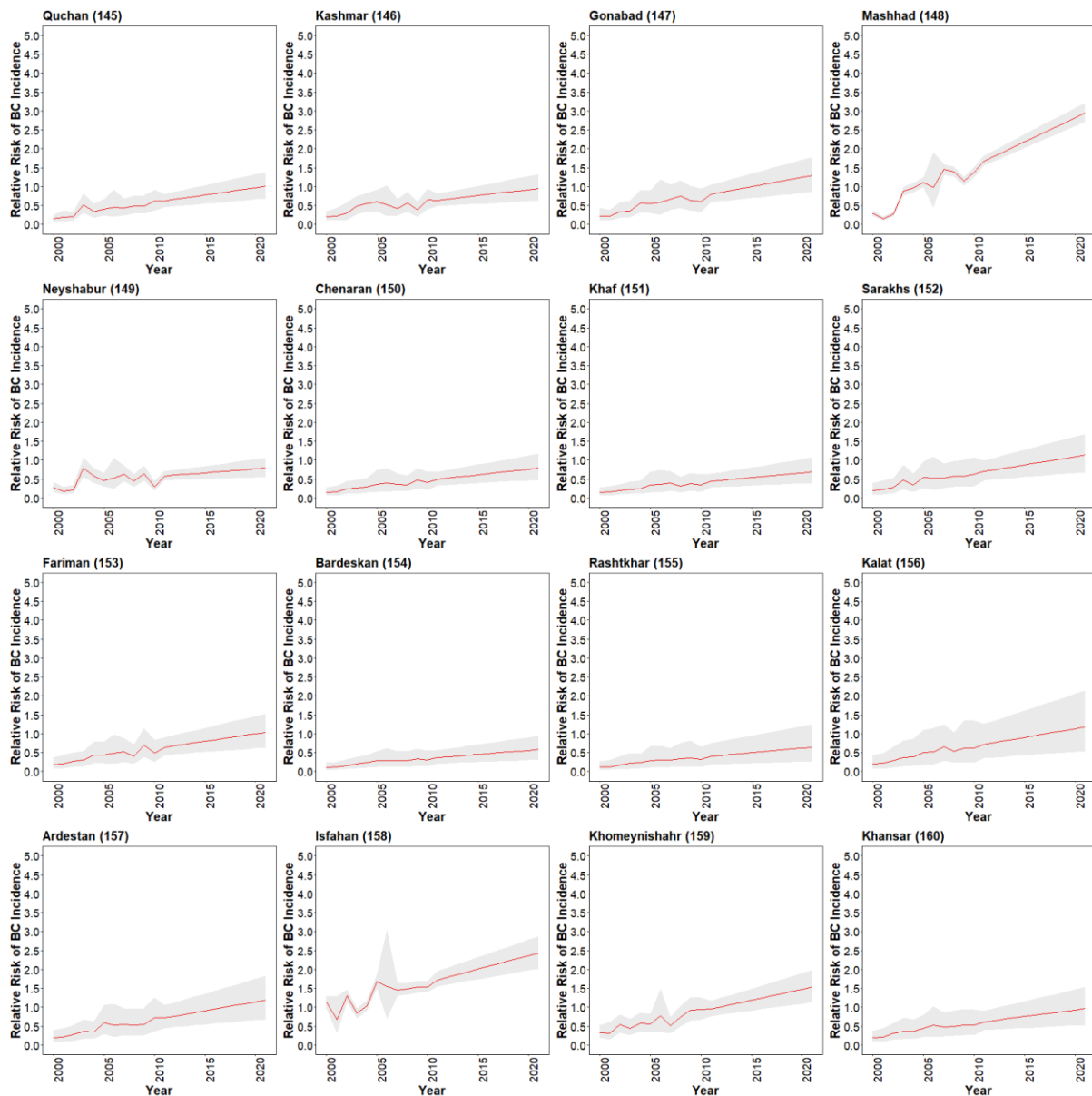

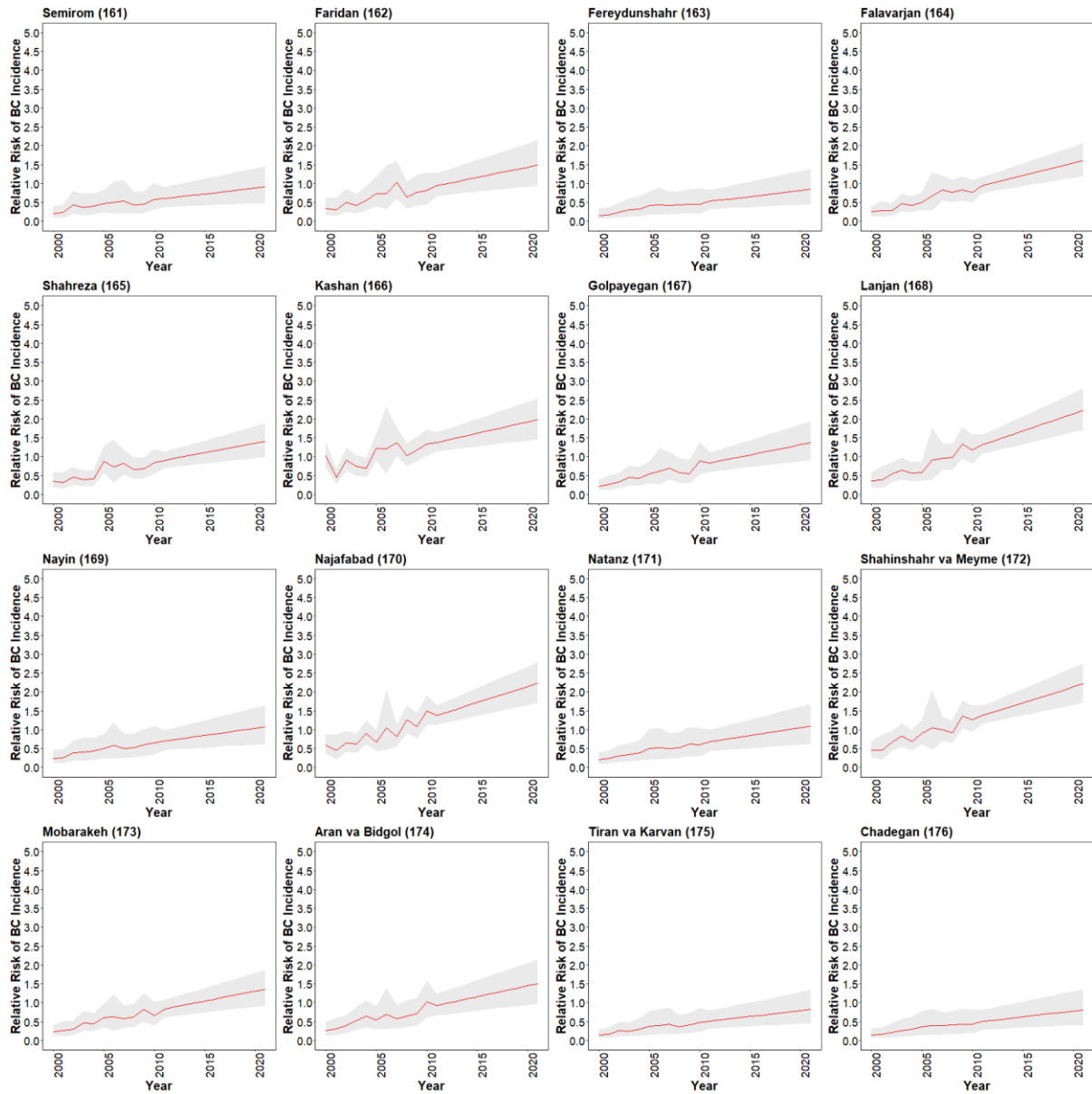

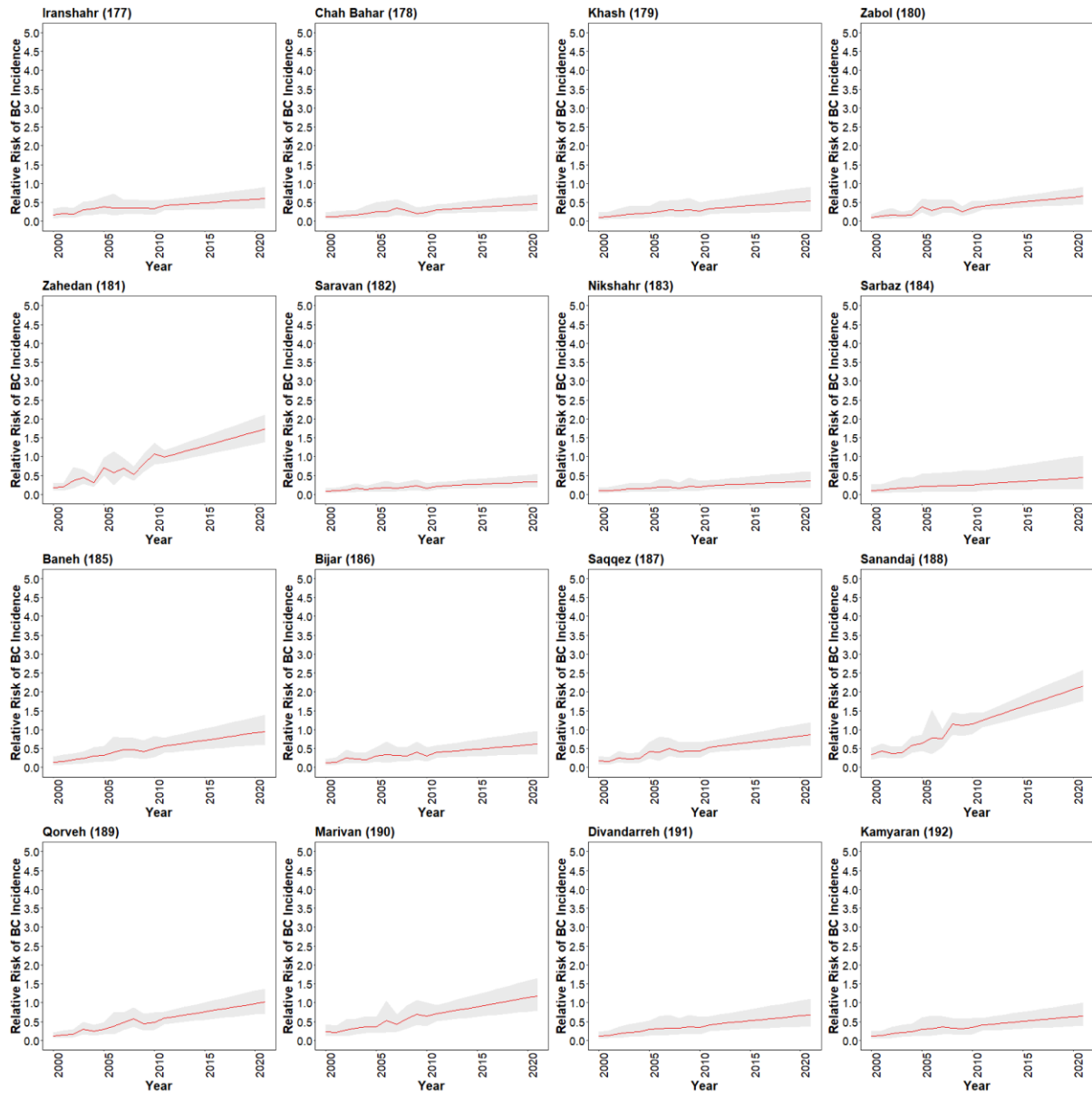

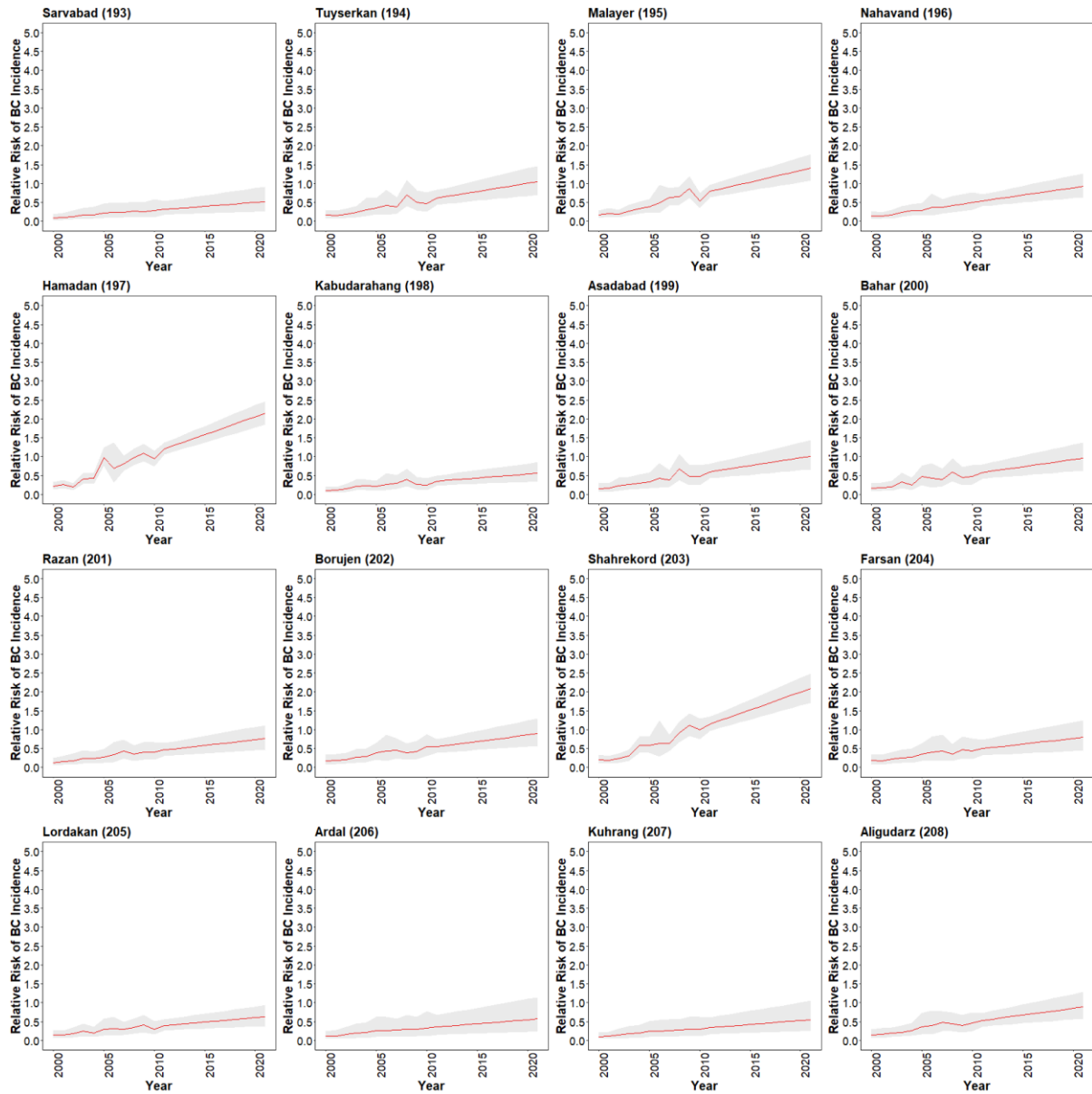

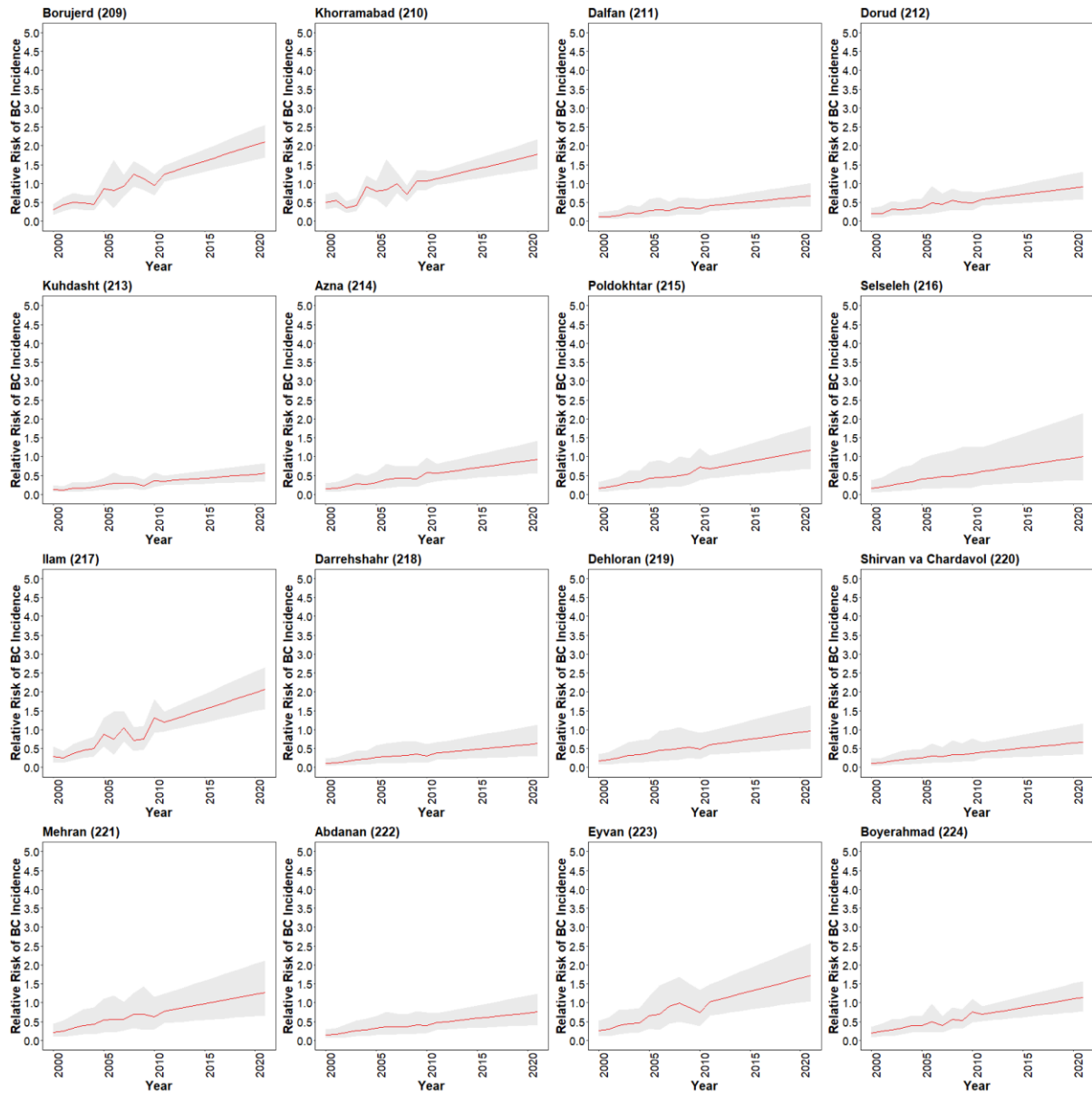

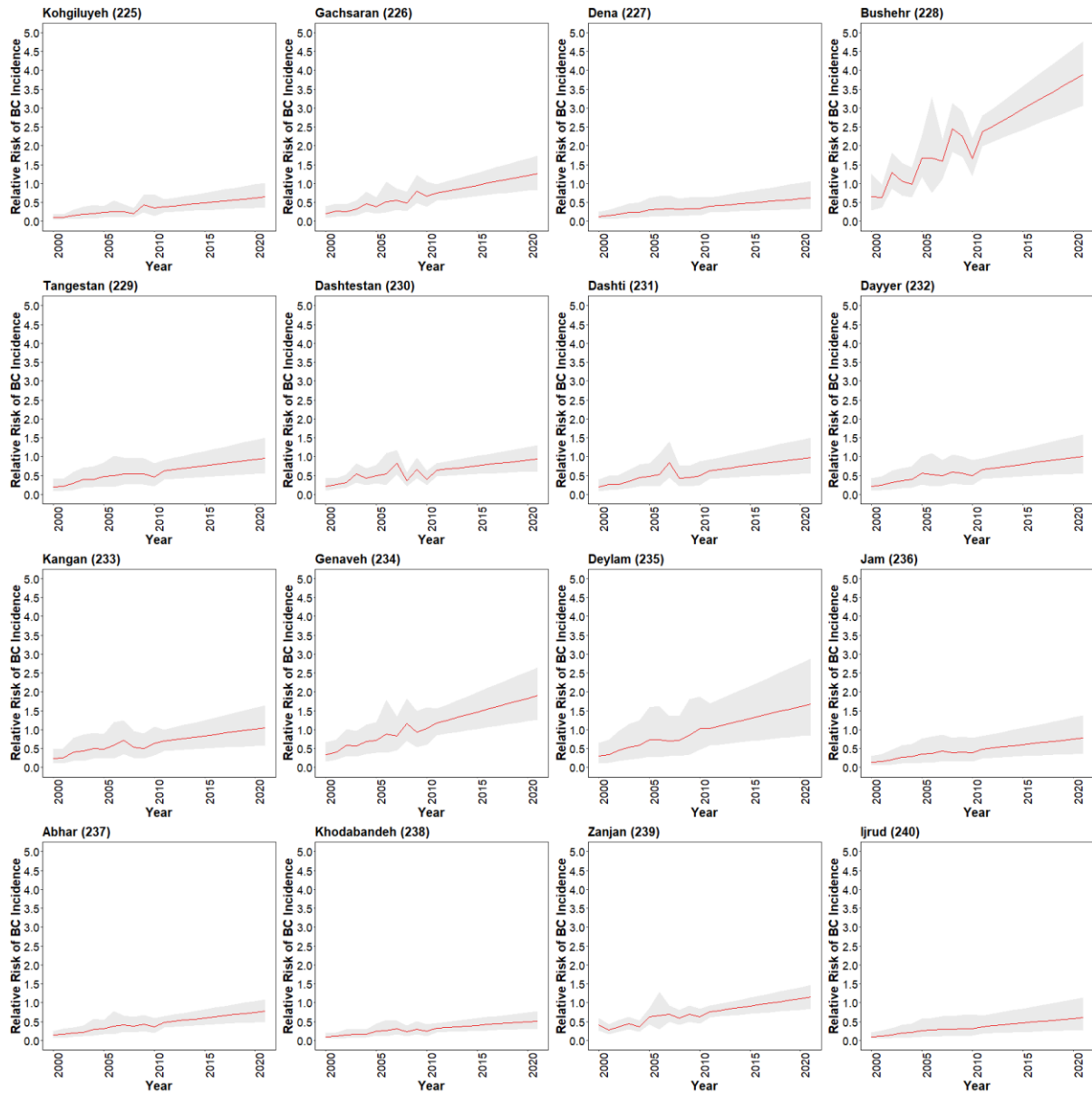

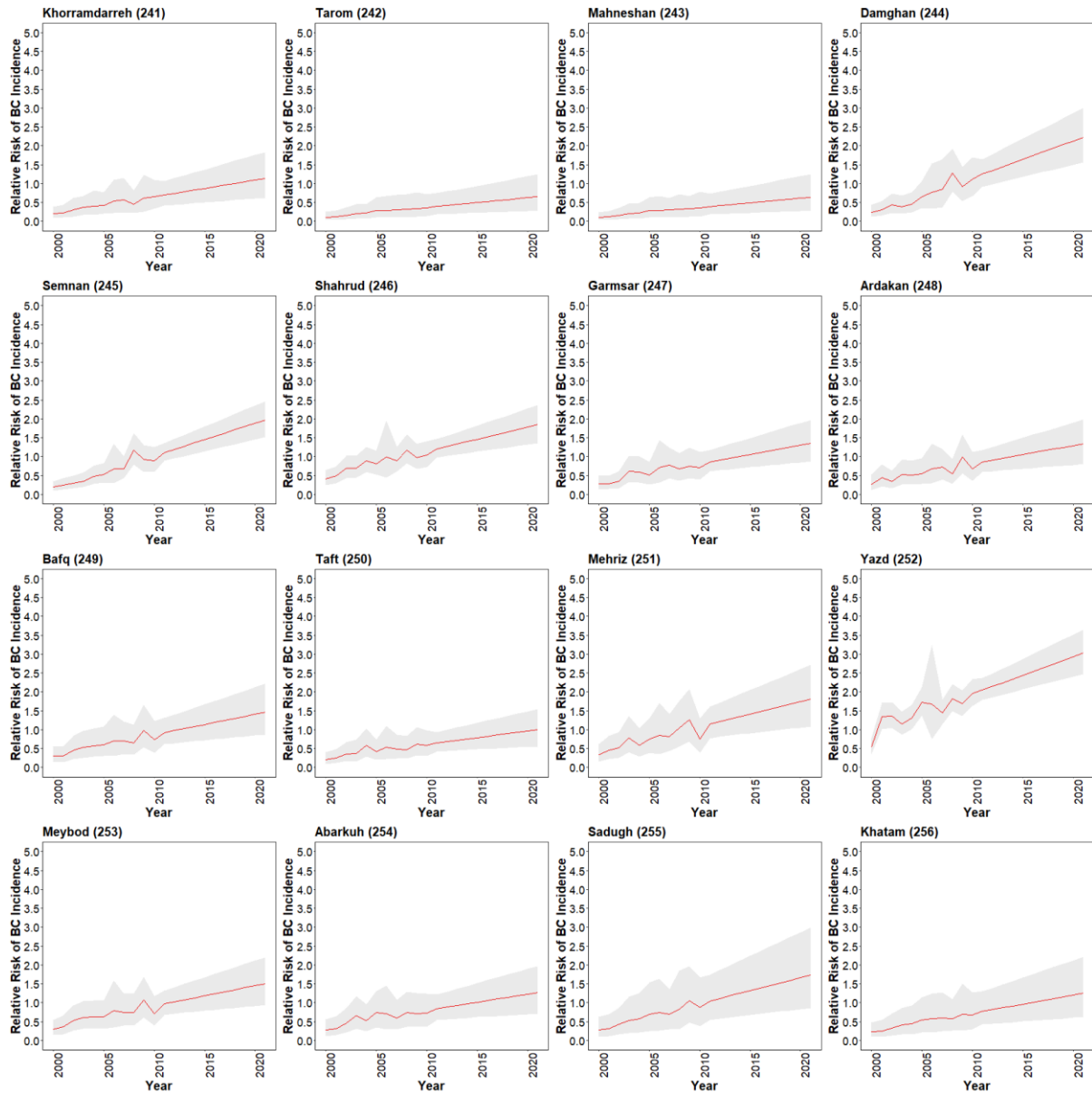

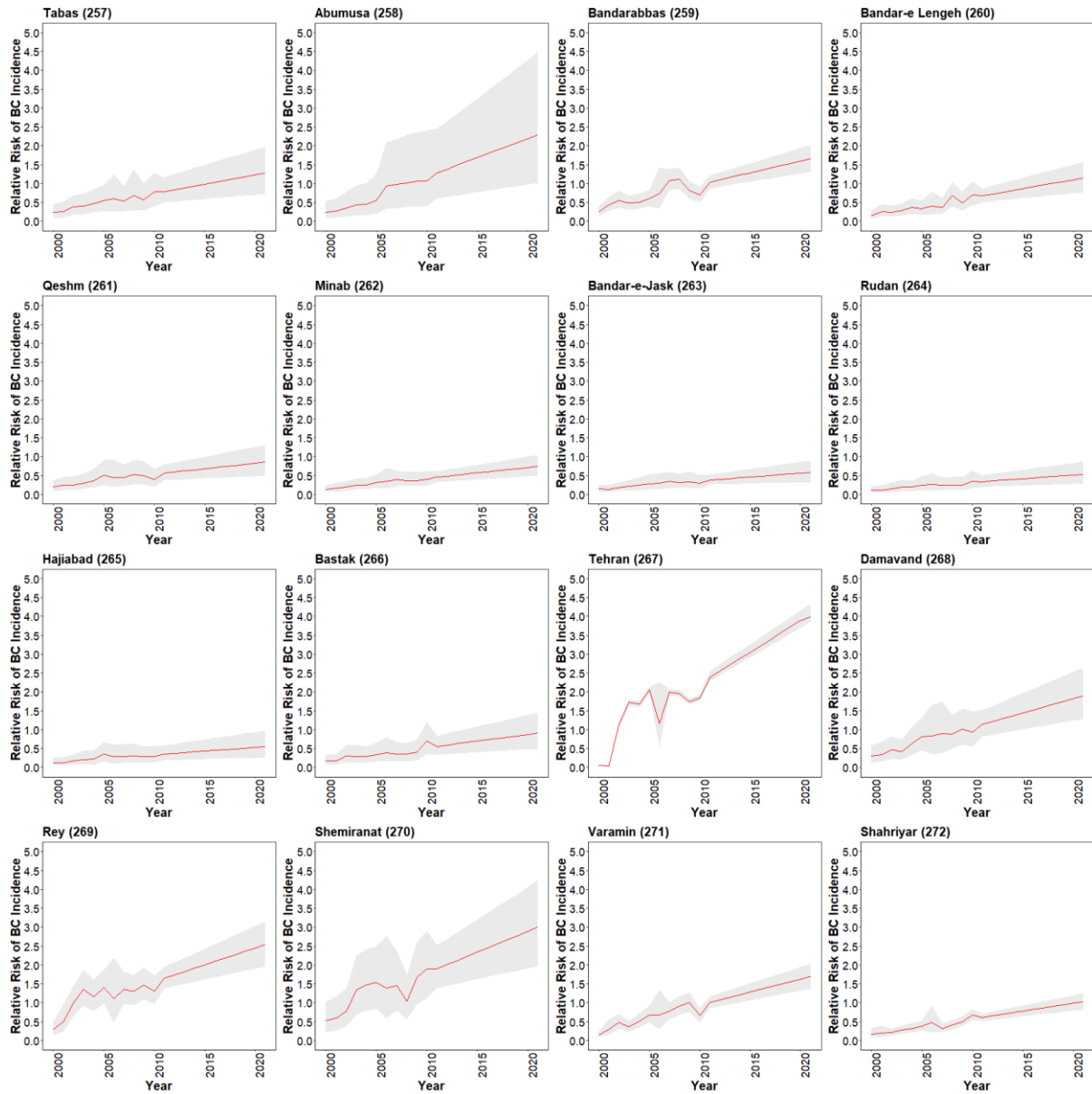

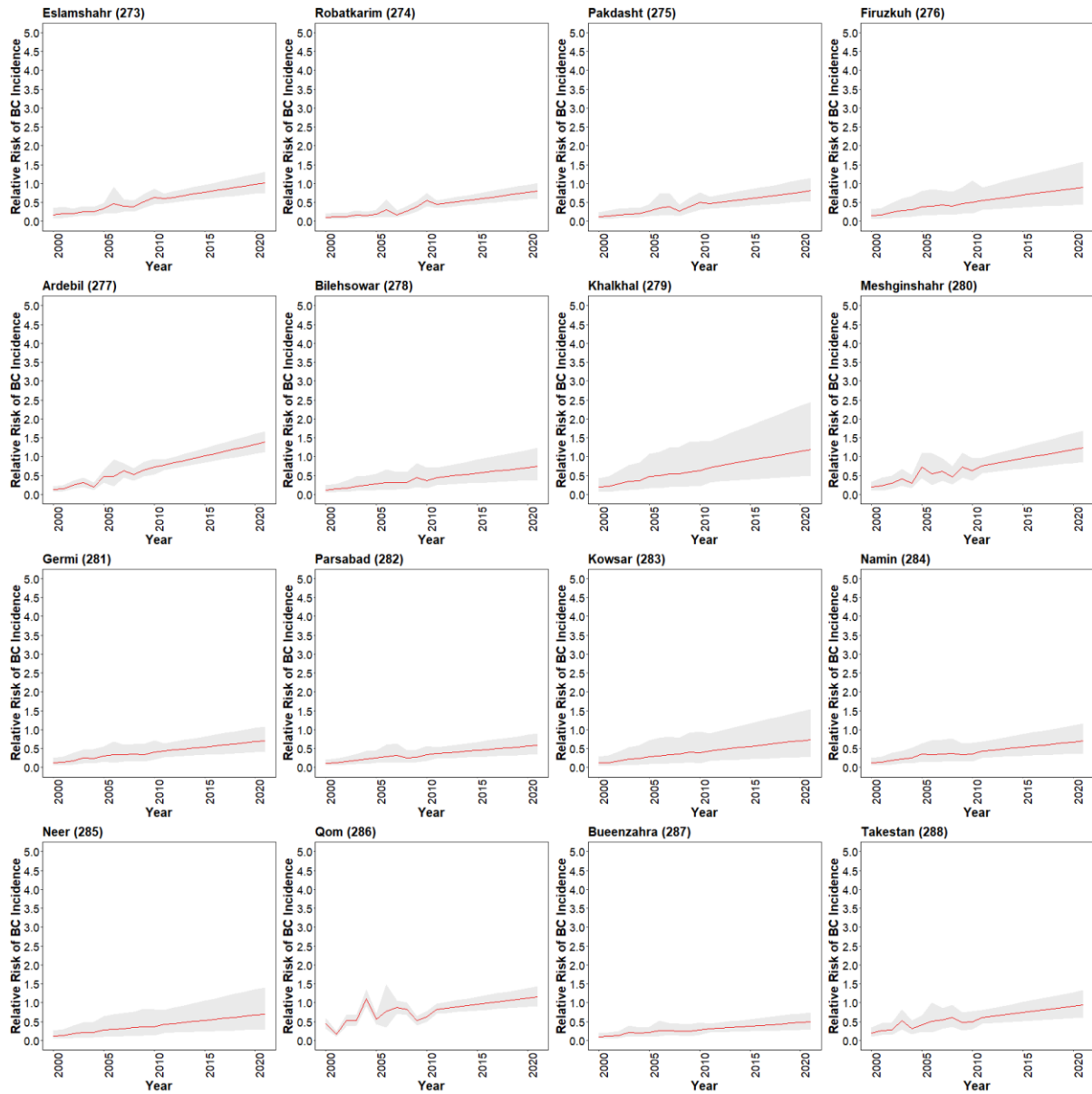

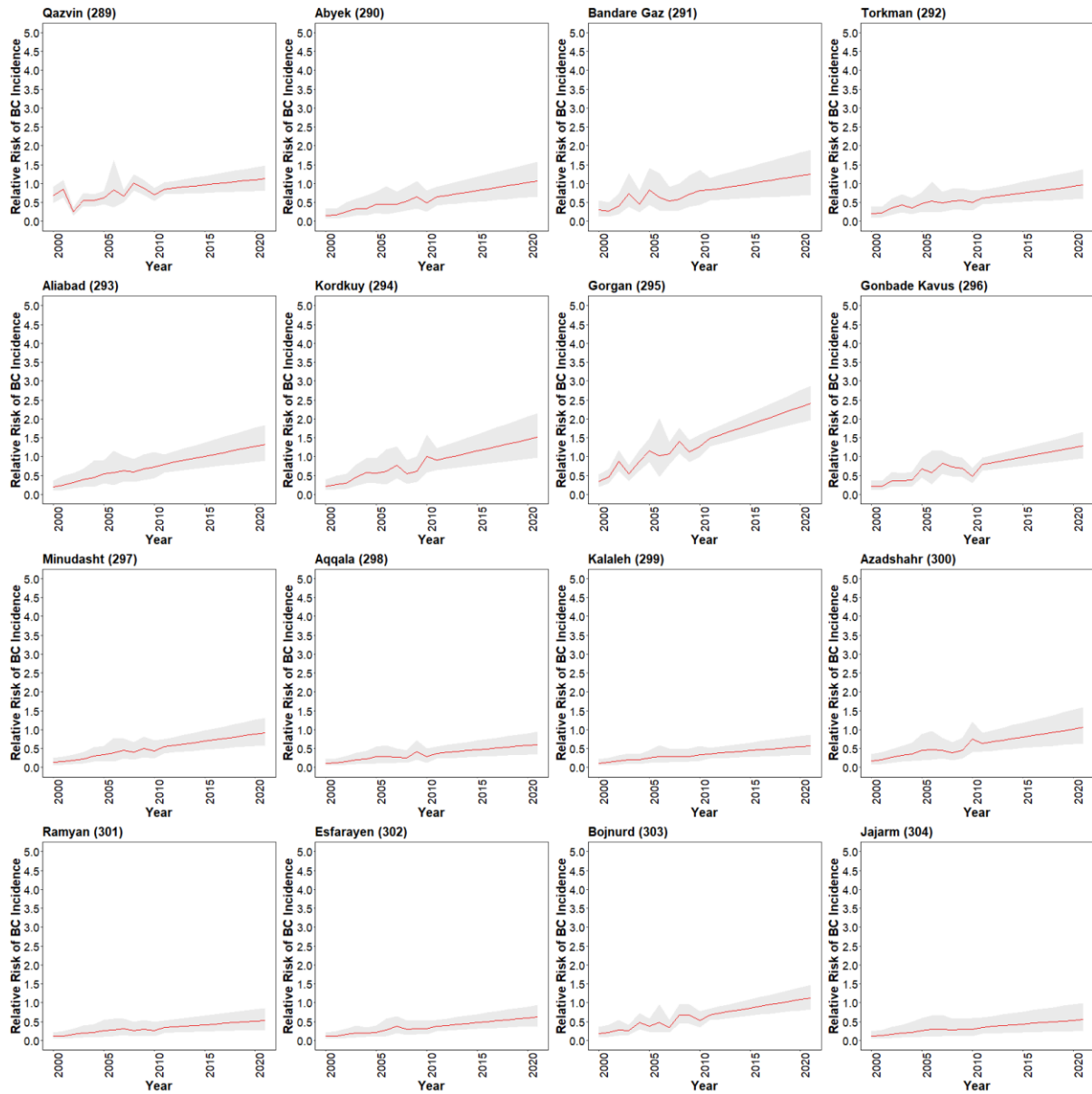

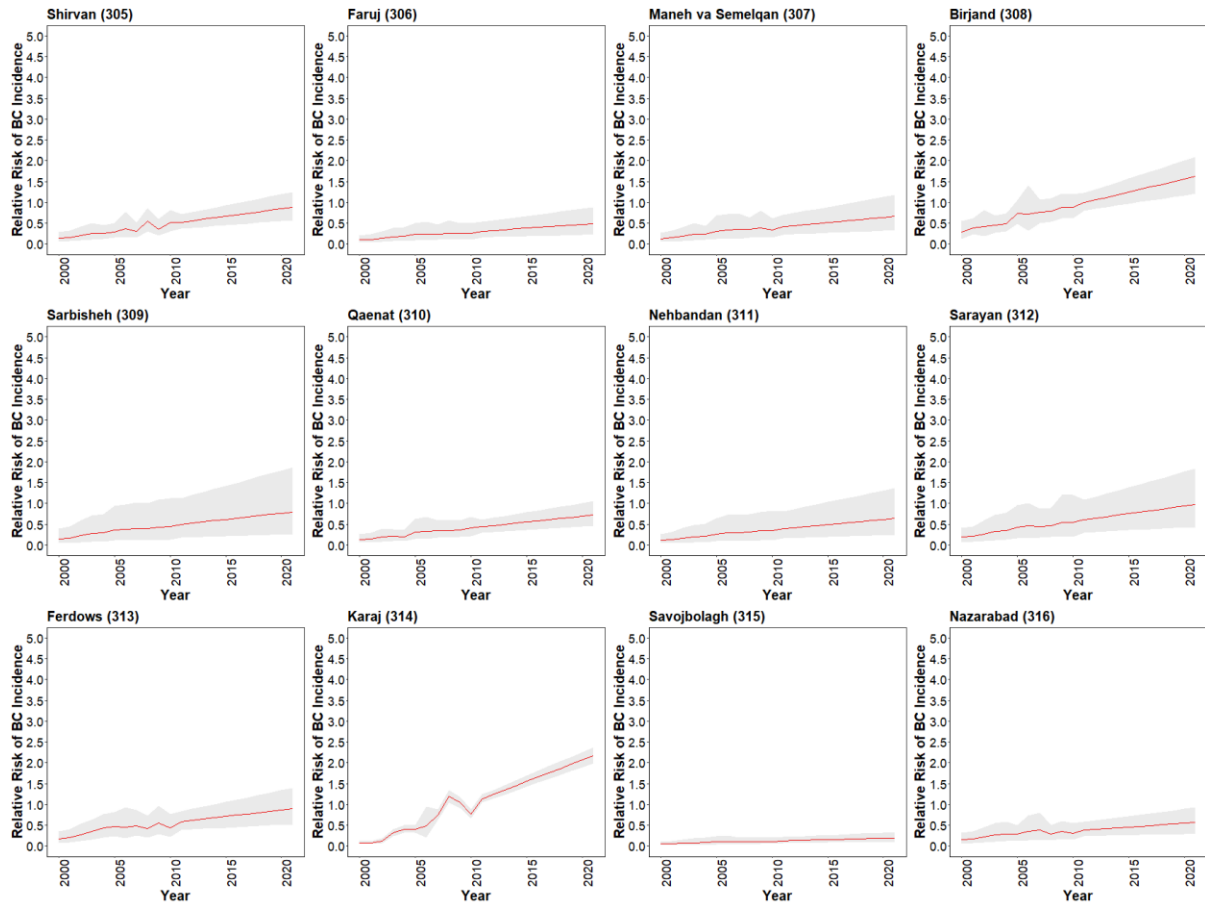

Supplement: S6 Fig — The grey shading shows the 95% credible intervals. (PDF) [file pone.0330017.s006.pdf]

bgr diagnostic

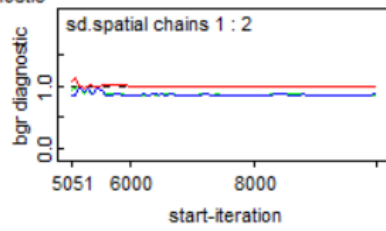

bgr diagnostic

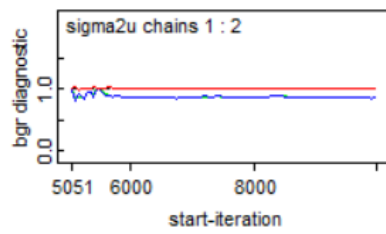

bgr diagnostic

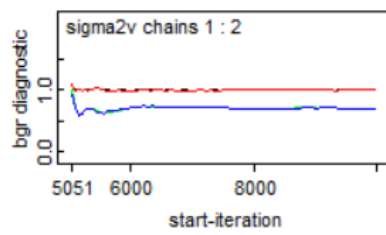

bgr diagnostic

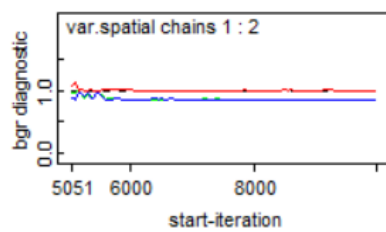

Supplement: S7 Fig — (PDF) [file pone.0330017.s007.pdf]

**S2 Table. R-hat index for the model-specific parameters to check model convergence.**


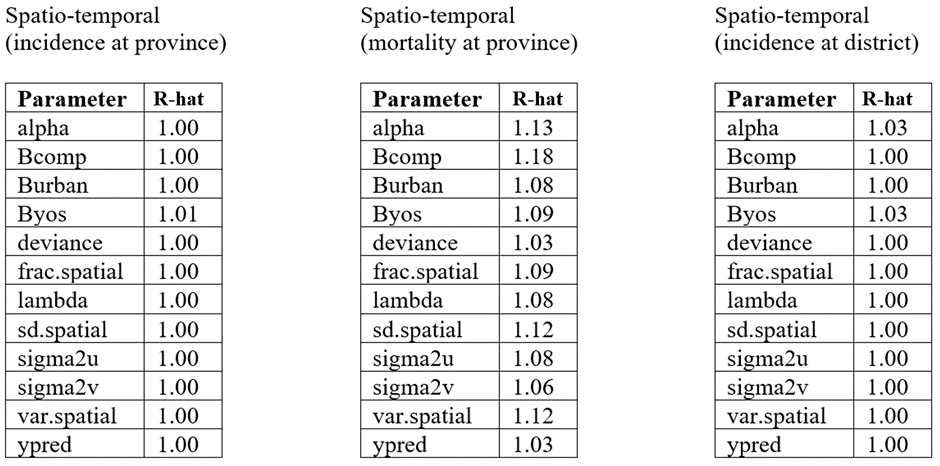

Supplement: S2 Table — (DOCX) [file pone.0330017.s011.docx]
