# Supplementary material for: Spatiotemporal disparity of breast cancer incidence in Iranian female populations at the district level from 2000 to 2021: Bayesian disease mapping
Source: PLoS One. 2025 Sep 11;20(9):e0330017. doi: 10.1371/journal.pone.0330017 (PMC12425319; doi:10.1371/journal.pone.0330017)
Supplement: S2 Fig — Cancer registry data were not available in 2006, so the SMR map for 2006 is unavailable. (PDF) [file pone.0330017.s002.pdf]

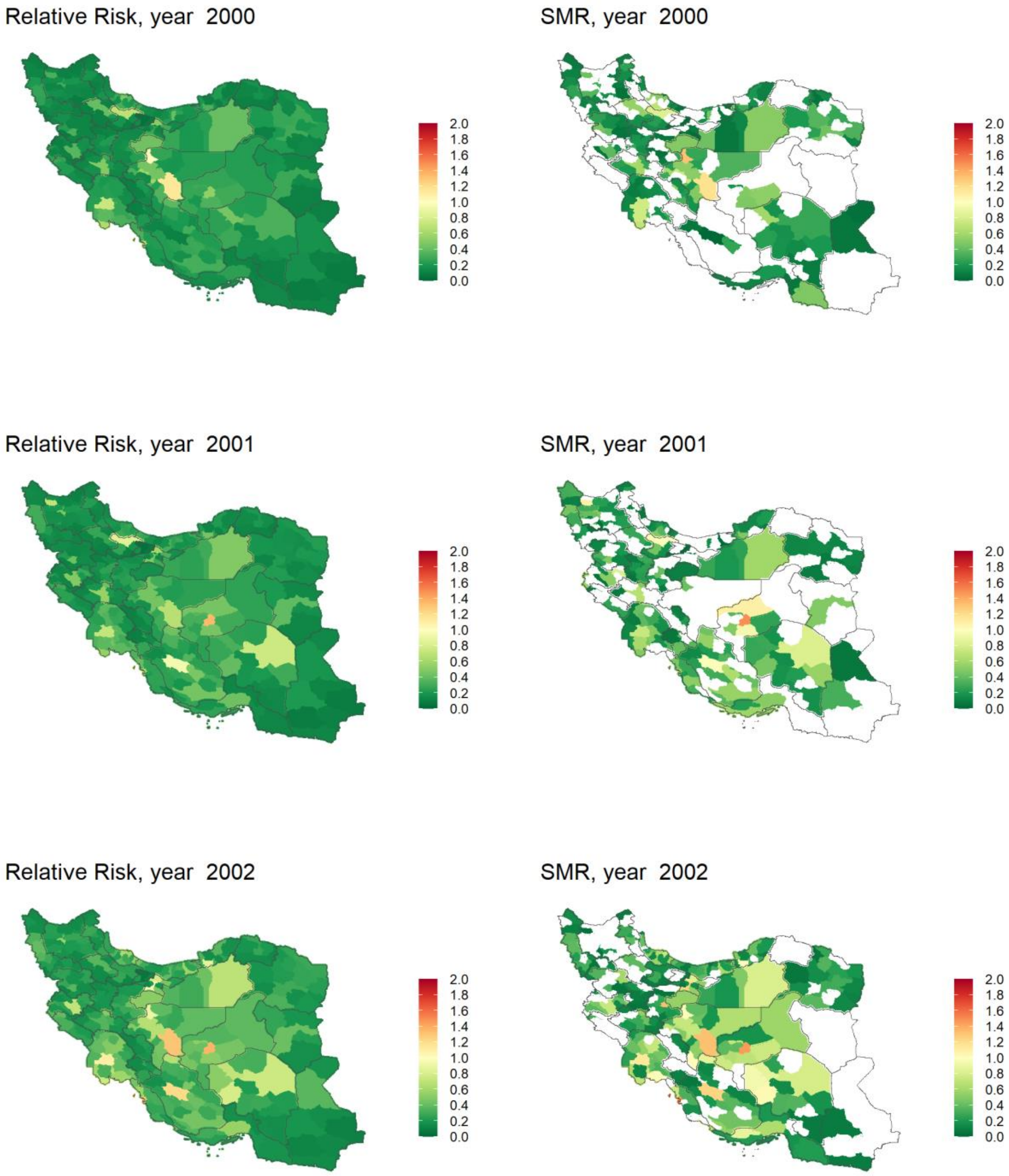
Relative Risk, year 2000
SMR, year 2000
Relative Risk, year 2001
SMR, year 2001
Relative Risk, year 2002
SMR, year 2002
2.0
1.8
1.6
1.4
1.2
1.0
0.8
0.6
0.4
0.2
0.0

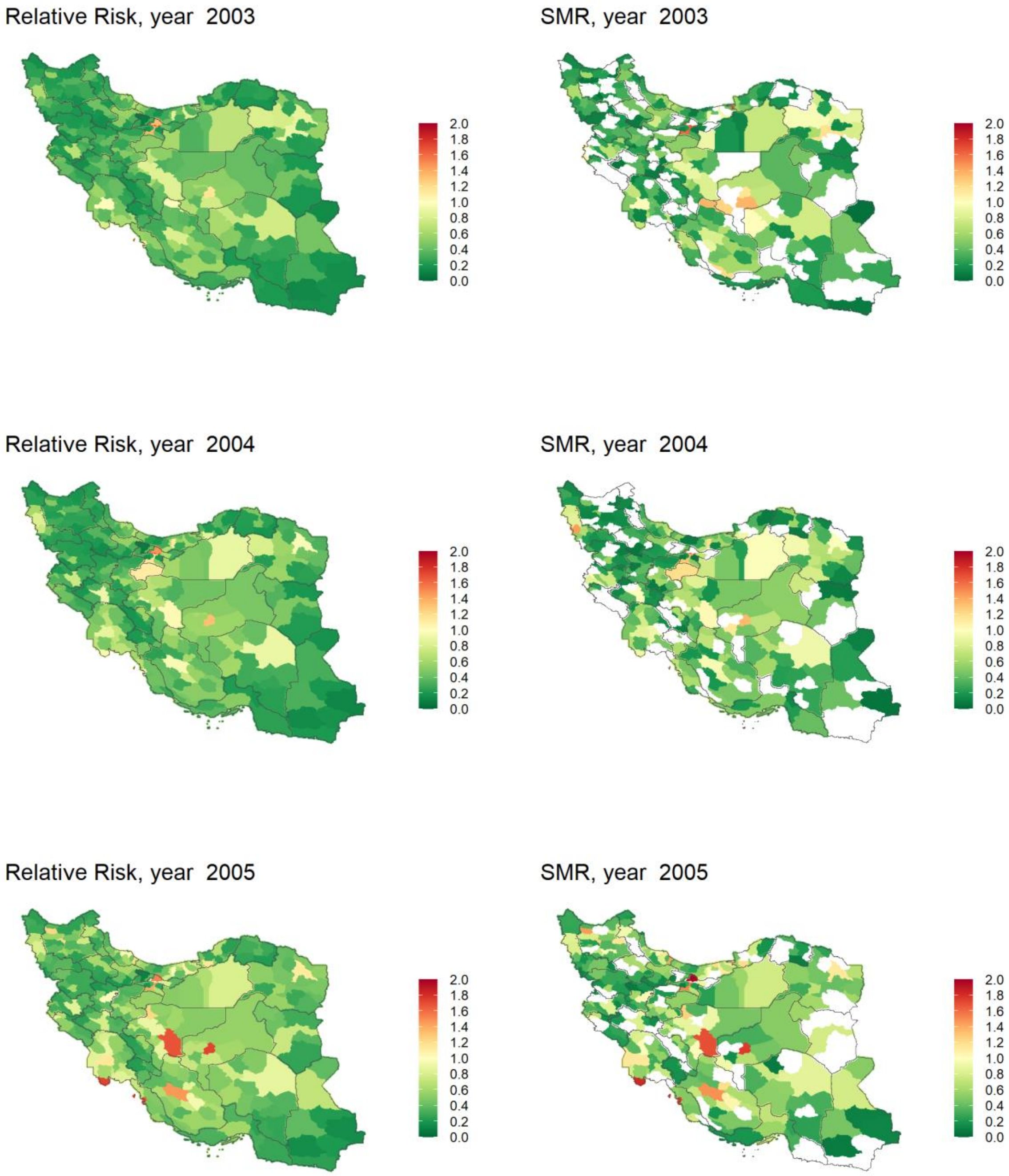
Relative Risk, year 2003
SMR, year 2003
Relative Risk, year 2004
SMR, year 2004
Relative Risk, year 2005
SMR, year 2005
2.0
1.8
1.6
1.4
1.2
1.0
0.8
0.6
0.4
0.2
0.0

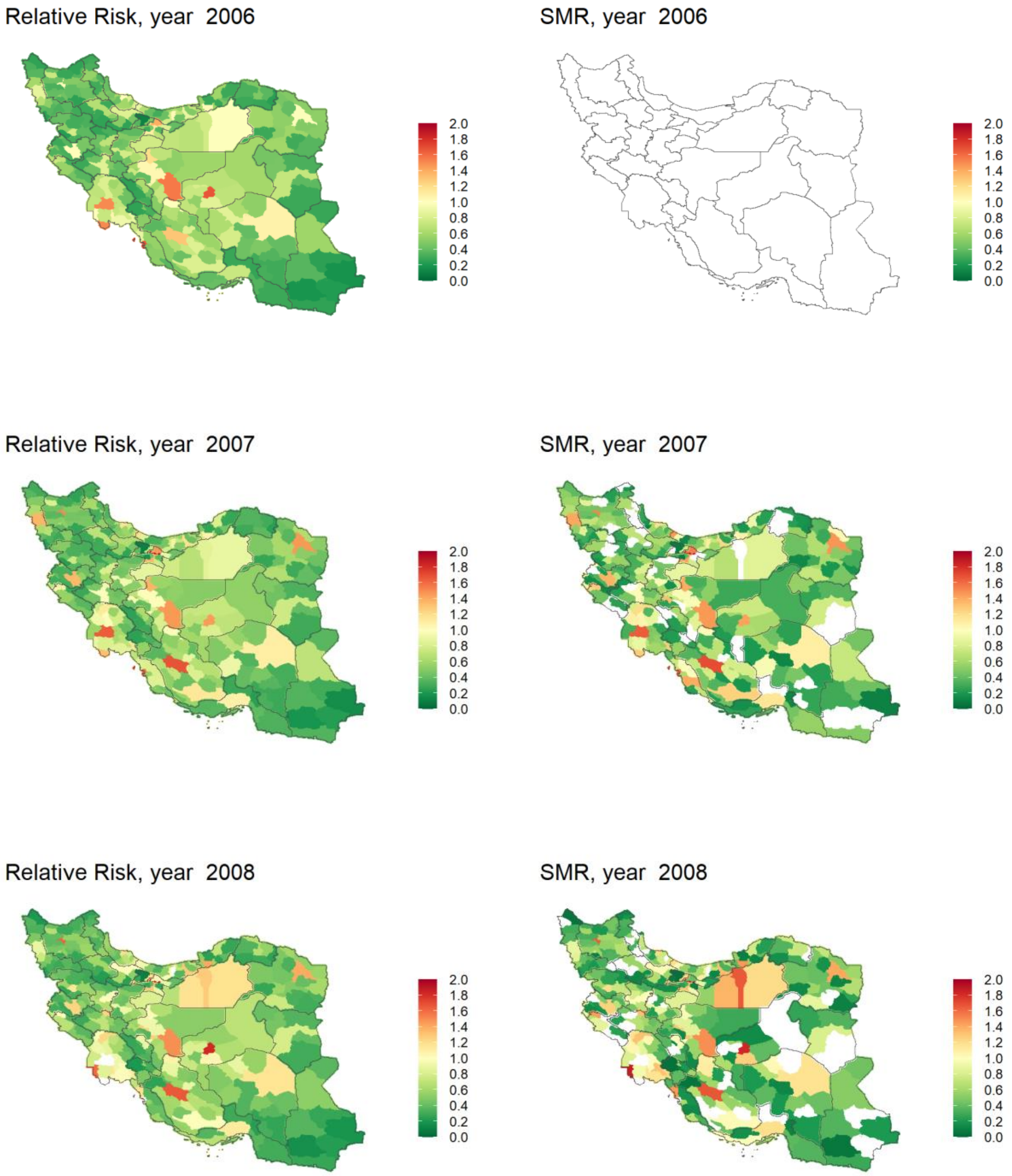
Relative Risk, year 2006
SMR, year 2006
Relative Risk, year 2007
SMR, year 2007
Relative Risk, year 2008
SMR, year 2008
2.0
1.8
1.6
1.4
1.2
1.0
0.8
0.6
0.4
0.2
0.0

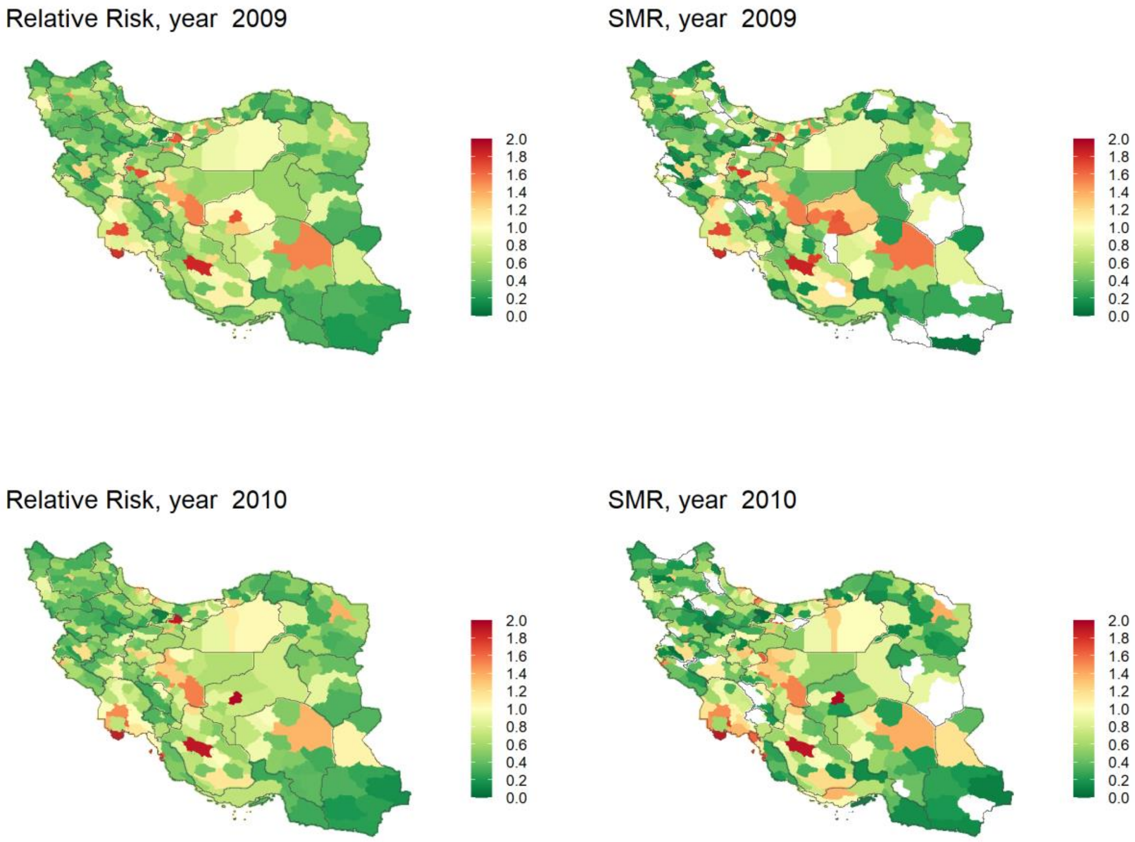
Relative Risk, year 2009
SMR, year 2009
Relative Risk, year 2010
SMR, year 2010
2.0
1.8
1.6
1.4
1.2
1.0
0.8
0.6
0.4
0.2
0.0
