# Supplementary material for: Spatiotemporal disparity of breast cancer incidence in Iranian female populations at the district level from 2000 to 2021: Bayesian disease mapping
Source: PLoS One. 2025 Sep 11;20(9):e0330017. doi: 10.1371/journal.pone.0330017 (PMC12425319; doi:10.1371/journal.pone.0330017)

Relative Risk, year 2011

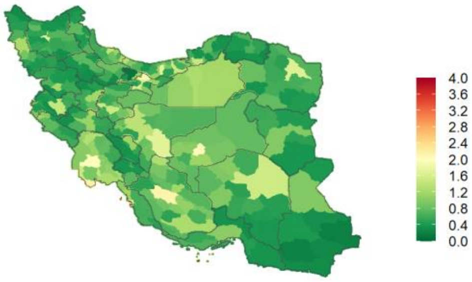

Relative Risk, year 2012

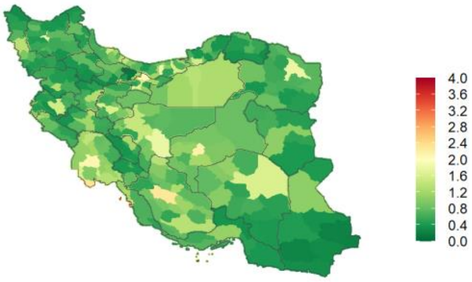

Relative Risk, year 2013

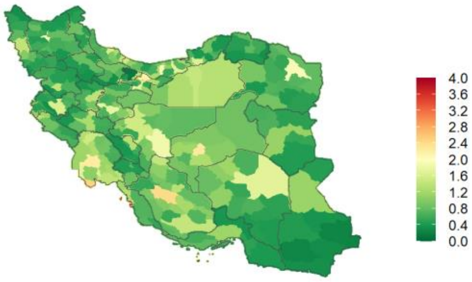

Relative Risk, year 2014

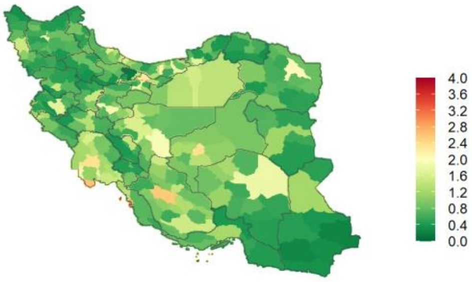

Relative Risk, year 2015

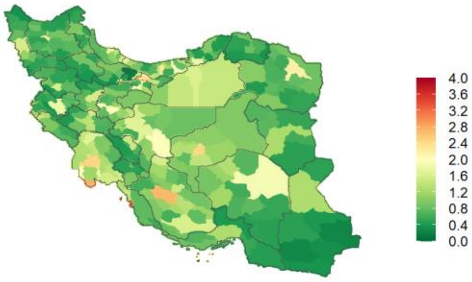

Relative Risk, year 2016

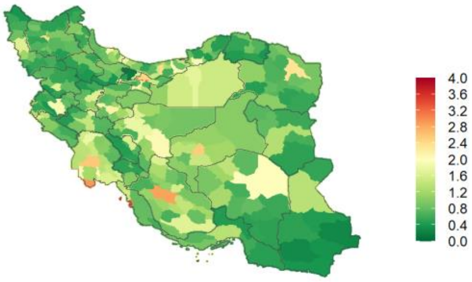

Relative Risk, year 2017

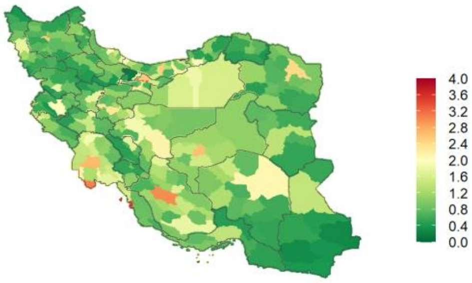

Relative Risk, year 2018

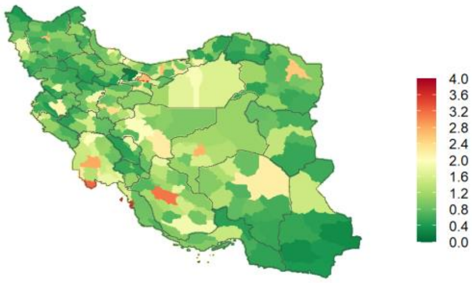

Relative Risk, year 2019

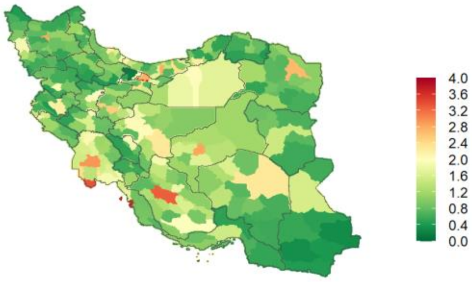

Relative Risk, year 2020

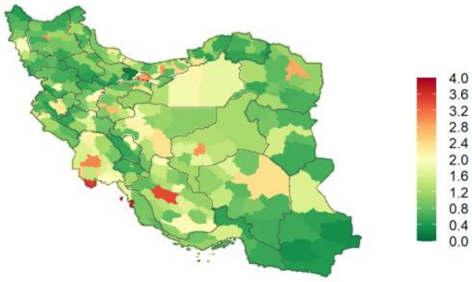

Relative Risk, year 2021

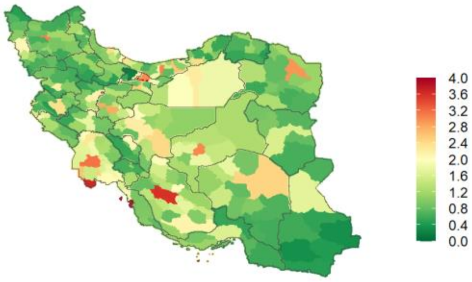

Supplement: S5 Fig — (PDF) [file pone.0330017.s005.pdf]
