# Supplementary material for: Spatiotemporal disparity of breast cancer incidence in Iranian female populations at the district level from 2000 to 2021: Bayesian disease mapping
Source: PLoS One. 2025 Sep 11;20(9):e0330017. doi: 10.1371/journal.pone.0330017 (PMC12425319; doi:10.1371/journal.pone.0330017)
Supplement: S1 Table — (DOCX) [file pone.0330017.s008.docx]

**S1 Table.** **List of provinces and districts in Iran.**

| **Province (total: 31)** | **Districts (total: 316)** |
| --- | --- |
| **Markazi (10) *** | Arak, Ashtiyan, Tafresh, Khomeyn, Delijan, Saveh, Shazand, Mahalat, Zarandiyeh, Komijan |
| **Gilan (16)** | Astara, Astanehye Ashrafiyeh, Bandar Anzali, Tavalesh, Rasht, Rudbar, Rudsar, Sumehsara, Fuman, Langrud, Lahijan, Shaft, Amlash, Rezvanshahr, Siyahkal, Masal |
| **Mazandaran (15)** | Amol, Babol, Behshahr, Tonekabon, Ramsar, Sari, Savadkuh, Qaemshahr, Nur, Noshahr, Babolsar, Mahmudabad, Neka, Chalus, Juybar |
| **Azerbaijan, East (19)** | Ahar, Tabriz, Sarab, Maragheh, Marand, Miyaneh, Hashtrud, Bonab, Bostanabad, Shabestar, Kalibar, Haris, Jolfa, Malekan, Azarshahr, Osku, Charoymaq, Varzaqan, Ajabshir |
| **Azerbaijan, West (14)** | Orumiyeh, Piranshahr, Khoy, Sardasht, Salmas, Maku, Mahabad, Miyandoab, Naqadeh, Bukan, Shahindezh, Takab, Oshnaviyeh, Chaldoran |
| **Kermanshah (12)** | Eslamabade Gharb, Kermanshah, Paveh, Sarpole Zahab, Sonqor, Qasreshirin, Kangavar, Gilanegharb, Javanrud, Sahneh, Harsin, Salas-e-Babajani |
| **Khuzestan (18)** | Abadan, Andimeshk, Ahvaz, Izeh, Bandar-e-Mahshahr, Behbahan, Khorramshahr, Dezful, Dashte Azadegan, Ramhormoz, Shadegan, Shushtar, Masjedsoleyman, Shush, Baghmalek, Omidiyeh, Lali, Hendijan |
| **Fars (22)** | Abadeh, Estahban, Eqlid, Jahrom, Darab , Sepidan, Shiraz, Fasa, Firuzabad, Kazerun, Lar (Larestan), Marvdasht, Mamasany, Neyriz, Lamard, Bovanat, Arsanjan, Khorrambid, Zarrindasht, Qirokarzin, Mohr, Farashband |
| **Kerman (13)** | Baft, Bam, Jiroft, Rafsanjan, Zarand, Sirjan, Shahrebabak, Kerman, Kahnuj, Bardsir, Ravar, Anbarabad, Manujan |
| **Khorasan, Razavi (17)** | Taybad, Torbate Heydarieh, Torbate Jam, Darrehgaz, Sabzevar, Quchan, Kashmar, Gonabad, Mashhad, Neyshabur, Chenaran, Khaf, Sarakhs, Fariman, Bardeskan, Rashtkhar, Kalat |
| **Isfahan (20)** | Ardestan, Isfahan, Khomeynishahr, Khansar, Semirom, Faridan, Fereydunshahr, Falavarjan, Shahreza, Kashan, Golpayegan, Lanjan, Nayin, Najafabad, Natanz, Shahinshahr va Meyme, Mobarakeh, Aran va Bidgol, Tiran va Karvan, Chadegan |
| **Sistan and Baluchistan (8)** | Iranshahr, Chah Bahar, Khash, Zabol, Zahedan, Saravan, Nikshahr, Sarbaz |
| **Kordestan (9)** | Baneh, Bijar, Saqqez, Sanandaj, Qorveh, Marivan, Divandarreh, Kamyaran, Sarvabad |
| **Hamadan (8)** | Tuyserkan, Malayer, Nahavand, Hamadan, Kabudarahang, Asadabad, Bahar, Razan |
| **Chahar Mahaal and Bakhtiari (6)** | Borujen, Shahrekord, Farsan, Lordakan, Ardal, Kuhrang |
| **Lorestan (9)** | Aligudarz, Borujerd, Khorramabad, Dalfan, Dorud, Kuhdasht, Azna, Poldokhtar, Selseleh |
| **Ilam (7)** | Ilam, Darrehshahr, Dehloran, Shirvan va Chardavol, Mehran, Abdanan, Eyvan |
| **Kohgiluyeh and Boyer-Ahmad (4)** | Boyerahmad, Kohgiluyeh, Gachsaran, Dena |
| **Bushehr (9)** | Bushehr, Tangestan, Dashtestan, Dashti, Dayyer, Kangan, Genaveh, Deylam, Jam |
| **Zanjan (7)** | Abhar, Khodabandeh, Zanjan, Ijrud, Khorramdarreh, Tarom, Mahneshan |
| **Semnan (4)** | \| Semnan, \| Damghan, \| Semnan, \| Shahrud \| \| --- \| --- \| --- \| --- \| |
| **Yazd (10)** | Ardakan, Bafq, Taft, Mehriz, Yazd, Meybod, Abarkuh, Sadugh, Khatam, Tabas |
| **Hormozgan (9)** | Abumusa, Bandarabbas, Bandar-e Lengeh, Qeshm, Minab, Bandar-e-Jask, Rudan, Hajiabad, Bastak |
| **Tehran (10)** | Tehran, Damavand, Rey, Shemiranat, Varamin, Shahriyar, Eslamshahr, Robatkarim, Pakdasht, Firuzkuh |
| **Ardabil (9)** | Ardebil, Bilehsowar, Khalkhal, Meshginshahr, Germi, Parsabad, Kowsar, Namin, Neer |
| **Qom (1)** | Qom |
| **Qazvin (4)** | Bueenzahra, Takestan, Qazvin, Abyek |
| **Golestan (11)** | Bandare Gaz, Torkman, Aliabad, Kordkuy, Gorgan, Gonbade Kavus, Minudasht, Aqqala, Kalaleh, Azadshahr, Ramyan |
| **Khorasan, North (6)** | Esfarayen, Bojnurd, Jajarm, Shirvan, Faruj, Maneh va Semelqan |
| **Khorasan, South (6)** | Birjand, Sarbisheh, Qaenat, Nehbandan, Sarayan, Ferdows |
| **Alborz (3)** | Karaj, Savojbolagh, Nazarabad |

*The number in brackets shows the total number of districts in each province
