# Supplementary material for: Spatiotemporal disparity of breast cancer incidence in Iranian female populations at the district level from 2000 to 2021: Bayesian disease mapping
Source: PLoS One. 2025 Sep 11;20(9):e0330017. doi: 10.1371/journal.pone.0330017 (PMC12425319; doi:10.1371/journal.pone.0330017)
Supplement: S1 Appendix — (DOCX) [file pone.0330017.s009.docx]

**S1 Appendix. Spatiotemporal model in OpenBUGS.**

**# Y = Breast cancer count (incidence/mortality)**

**# i = districts (316)**

**# t = year (T=11, from 2000(1)-2010(11))**

**# Y~ Poisson(lambda*Exp)**

**#Ndist = 316/#Nprov = 31**

**model {**

**for (i in 1: Ndist) {**

**for (t in 1: T) {**

**Y [i, t] ~ dpois (mu [i, t])**

**mu [i, t] <- lambda [i, t] *E [i, t]**

**log (lambda [i, t]) <- v [i, t]**

**v [i, t] ~dnorm (mu2[i, t], precv) #precv = tau. v = 1/variance. v**

**mu2 [i, t] <- alpha +u[i] +Byos*predyos [i, t] + Burban*urbanisation [i, t] +Bcomp*median_com [i, t] + xi[t]**

**ypred [i, t] ~ dpois (mu [i, t])**

**}}**

**Byos ~ dnorm (0, 0.0001)**

**Burban ~ dnorm (0, 0.0001)**

**Bcomp ~ dnorm (0, 0.0001)**

**#Put a hyperprior on U**

**u [1: Ndist] ~ car. normal (adj [], weights [], num [], precu) # spatial model**

**for (k in 1: sumNumNeigh) {weights[k]<-1}**

**#Temporal random effects - Random Walk order 1**

**xi [1: T] ~ car. Normal (adj. time [], weights. time [], num. time [], precxi)**

**for (n in 1: sumNumNeigh.time) {weights. time[n] <- 1}**

**# other priors**

**precu ~ dgamma (0.5,0.0005)**

**precv ~ dgamma (0.5,0.0005)**

**precxi ~ dgamma (0.5,0.0005)**

**alpha ~ dflat ()**

**sigma2u<-1/precu**

**sigma2v<-1/precv**

**sigma2xi<-1/precxi**

**sd. spatial<-sd (u [1: Ndist])**

**var. spatial<- sd. spatial*sd. spatial**

**frac. spatial<-var. spatial/(var. spatial+sigma2v)**

**}**
