# Supplementary material for: Spatiotemporal disparity of breast cancer incidence in Iranian female populations at the district level from 2000 to 2021: Bayesian disease mapping
Source: PLoS One. 2025 Sep 11;20(9):e0330017. doi: 10.1371/journal.pone.0330017 (PMC12425319; doi:10.1371/journal.pone.0330017)
Supplement: S2 Appendix — (DOCX) [file pone.0330017.s010.docx]

**S2 Appendix.** **Model convergence and goodness of fit.**

Various metrics were used to show the quality of model convergence and the quality of model fitting. These measurements provided quantitative values that allowed us to check whether the convergence and fitting processes were acceptable.

The model’s convergence was assessed both informally via visual inspection of trace and density plots (**S7 Fig**) and formally using the Brooks-Gelman-Rubin (BGR) diagnostic. The BGR statistic, also known as R-hat, compares the between- and within-chain variances to assess convergence. An R-hat value close to 1.0 indicates satisfactory convergence, with values below 1.1 considered acceptable [[1](https://paperpile.com/c/3Nlql5/muUb9), [2]](https://paperpile.com/c/3Nlql5/RVXOE). We examined the R-hat values and their progression across iterations to ensure stable convergence for all model parameters. The R-hat results are provided below (S2 Table).

The model’s goodness of fit was assessed using the deviation information criterion (DIC), Moran’s I test and cross-validation. The DIC is a hierarchical modelling generalisation of the Akaike information criterion (AIC), a quality estimator for evaluating and comparing statistical models [[3]](https://paperpile.com/c/3Nlql5/osFJU). It is used in Bayesian [model selection](https://en.wikipedia.org/wiki/Model_selection) problems where the [posterior distributions](https://en.wikipedia.org/wiki/Posterior_distribution) of the [models](https://en.wikipedia.org/wiki/Statistical_model) have been obtained by MCMC simulation [[4]](https://paperpile.com/c/3Nlql5/9fywJ). Many other researchers have also successfully used this approach in health monitoring applications [[5,6]](https://paperpile.com/c/3Nlql5/675C8+dteiI). Although the minimum DIC estimates the model with the best predictions, it is difficult to definitively determine what would establish a significant difference in DIC. Differences of more than ten might undoubtedly rule out a model with a higher DIC. Nevertheless, if the difference is less than five and the models provide very different results, it could be misleading to report only the model with the lowest DIC [[7]](https://paperpile.com/c/3Nlql5/Bg5qj). Since the model also borrows strength via covariates, we checked the improvement of model fitting according to the DIC using different covariates. A sensitivity analysis was also performed by fitting a model including the incidence of female obesity. Based on the DIC, the quality of this model fit was worse than that of the model we selected. Including an inappropriately large number of covariates would lead to a model driven by the covariates more than the outcome data themselves, so we included standard covariates in these models. The model residuals were also calculated, and Moran’s I test [[8]](https://paperpile.com/c/3Nlql5/AUyLP) was performed to check for spatial correlation among the residuals. The Moran’s I test computes the fitted line slope between the actual residual for each district and the mean residual calculated, including the neighbouring areas. The obtained Moran’s I coefficient (close to zero) and the associated p value (p > 0.05) indicated that there was no evidence to reject the null hypothesis of no spatial correlation. We then concluded that the model allows for appropriate spatial pattern information.

**Supporting references**

1. Gelman A, Hill J. Data analysis using regression and multilevel/hierarchical models. Cambridge: Cambridge University Press; 2006. doi:10.1017/CBO9780511790942
2. Bürkner P-C. brms: an R package for Bayesian multilevel models using Stan. J Stat Softw. 2017;80:1–28.
3. Cain MK, Zhang Z. Fit for a Bayesian: an evaluation of PPP and DIC for structural equation modeling. Struct Equ Modeling. 2019;26:39–50. doi:10.1080/10705511.2018.1490648
4. Ando T. Bayesian predictive information criterion for the evaluation of hierarchical Bayesian and empirical Bayes models. Biometrika. 2007;94:443–458. doi:10.1093/biomet/asm017
5. Asaria P, Fortunato L, Fecht D, Tzoulaki I, Abellan JJ, Hambly P, et al. Trends and inequalities in cardiovascular disease mortality across 7932 English electoral wards, 1982–2006: Bayesian spatial analysis. Int J Epidemiol. 2012;41:1737–1749.
6. Di Cesare M, Bhatti Z, Soofi SB, Fortunato L, Ezzati M, et al. Geographical and socioeconomic inequalities in women and children’s nutritional status in Pakistan in 2011: an analysis of data from a nationally representative survey. Lancet Glob Health. 2015;3:e229–e239. doi:10.1016/S2214-109X(15)70001-X
7. El-Basyouny K, Sayed T. Collision prediction models using multivariate Poisson-lognormal regression. Accid Anal Prev. 2009;41:820–828.
8. Moran PAP. A test for the serial independence of residuals. Biometrika. 1950;37:178–181.
