# Supplementary material for: Spatiotemporal disparity of breast cancer incidence in Iranian female populations at the district level from 2000 to 2021: Bayesian disease mapping
Source: PLoS One. 2025 Sep 11;20(9):e0330017. doi: 10.1371/journal.pone.0330017 (PMC12425319; doi:10.1371/journal.pone.0330017)
Supplement: S3 Table — (DOCX) [file pone.0330017.s012.docx]

**S3 Table. Posterior mean and 95% credible intervals for the RR of breast cancer incidence by district and year (sorted by mean values in 2010).**

| **District** | **2000** | **2005** | **2010** |
| --- | --- | --- | --- |
| **Yazd** | 0.54 (0.35, 0.76) | 1.73 (1.38, 2.12) | 1.96 (1.63, 2.33) |
| **Shiraz** | 0.11 (0.07, 0.16) | 1.46 (1.29, 1.66) | 1.9 (1.72, 2.09) |
| **Shemiranat** | 0.52 (0.23, 1.03) | 1.53 (0.84, 2.49) | 1.9 (1.12, 2.91) |
| **Abadan** | 0.64 (0.4, 0.95) | 1.73 (1.28, 2.25) | 1.87 (1.44, 2.37) |
| **Tehran** | 0.06 (0.04, 0.08) | 2.05 (1.95, 2.14) | 1.84 (1.76, 1.92) |
| **Bushehr** | 0.65 (0.29, 1.26) | 1.67 (1.18, 2.27) | 1.66 (1.2, 2.2) |
| **Bandar-e-Mahshahr** | 0.37 (0.2, 0.62) | 0.74 (0.46, 1.11) | 1.63 (1.2, 2.14) |
| **Isfahan** | 1.14 (0.98, 1.31) | 1.68 (1.5, 1.86) | 1.54 (1.4, 1.69) |
| **Ahvaz** | 0.74 (0.57, 0.92) | 1.14 (0.95, 1.35) | 1.5 (1.3, 1.72) |
| **Najafabad** | 0.59 (0.37, 0.88) | 0.66 (0.43, 0.96) | 1.49 (1.12, 1.91) |
| **Mashhad** | 0.29 (0.22, 0.37) | 1.12 (0.99, 1.25) | 1.38 (1.25, 1.51) |
| **Kerman** | 0.33 (0.21, 0.47) | 0.88 (0.67, 1.11) | 1.37 (1.12, 1.64) |
| **Khorramshahr** | 0.33 (0.17, 0.57) | 0.86 (0.52, 1.3) | 1.36 (0.91, 1.93) |
| **Kashan** | 1.02 (0.71, 1.4) | 1.22 (0.89, 1.61) | 1.34 (1.02, 1.72) |
| **Rasht** | 0.47 (0.34, 0.62) | 0.97 (0.79, 1.17) | 1.31 (1.12, 1.52) |
| **Ramsar** | 0.29 (0.14, 0.52) | 0.67 (0.37, 1.11) | 1.31 (0.81, 1.97) |
| **Ilam** | 0.29 (0.13, 0.56) | 0.88 (0.56, 1.31) | 1.31 (0.91, 1.81) |
| **Rey** | 0.29 (0.15, 0.49) | 1.4 (0.99, 1.87) | 1.31 (0.96, 1.72) |
| **Tabriz** | 0.31 (0.23, 0.4) | 1.21 (1.05, 1.39) | 1.27 (1.12, 1.43) |
| **Shahinshahr va Meyme** | 0.45 (0.27, 0.7) | 0.9 (0.62, 1.24) | 1.27 (0.95, 1.64) |
| **Gorgan** | 0.34 (0.2, 0.52) | 1.15 (0.86, 1.49) | 1.27 (0.99, 1.58) |
| **Arak** | 0.15 (0.09, 0.24) | 0.69 (0.5, 0.9) | 1.25 (1.03, 1.51) |
| **Babol** | 0.42 (0.28, 0.6) | 0.98 (0.74, 1.25) | 1.2 (0.96, 1.47) |
| **Abadeh** | 0.38 (0.17, 0.72) | 0.76 (0.43, 1.2) | 1.2 (0.76, 1.78) |
| **Delijan** | 0.25 (0.11, 0.48) | 0.56 (0.27, 1.01) | 1.19 (0.64, 1.95) |
| **Kermanshah** | 0.37 (0.26, 0.51) | 0.86 (0.69, 1.05) | 1.19 (1.01, 1.39) |
| **Lanjan** | 0.36 (0.2, 0.58) | 0.58 (0.36, 0.87) | 1.17 (0.82, 1.58) |
| **Hendijan** | 0.33 (0.13, 0.69) | 0.86 (0.35, 1.76) | 1.15 (0.57, 2.01) |
| **Lar (Larestan)** | 0.32 (0.14, 0.61) | 0.58 (0.36, 0.86) | 1.15 (0.83, 1.52) |
| **Sanandaj** | 0.34 (0.21, 0.52) | 0.63 (0.43, 0.87) | 1.15 (0.88, 1.46) |
| **Damghan** | 0.24 (0.12, 0.44) | 0.65 (0.35, 1.06) | 1.12 (0.67, 1.69) |
| **Bandar Anzali** | 0.47 (0.26, 0.76) | 0.9 (0.57, 1.31) | 1.1 (0.75, 1.53) |
| **Rudsar** | 0.26 (0.14, 0.44) | 1.19 (0.81, 1.66) | 1.09 (0.75, 1.51) |
| **Zahedan** | 0.18 (0.1, 0.3) | 0.71 (0.49, 0.98) | 1.07 (0.8, 1.37) |
| **Khorramabad** | 0.5 (0.32, 0.72) | 0.8 (0.59, 1.06) | 1.07 (0.83, 1.35) |
| **Abumusa** | 0.24 (0.09, 0.54) | 0.56 (0.21, 1.24) | 1.07 (0.4, 2.4) |
| **Lahijan** | 0.38 (0.21, 0.62) | 1.02 (0.69, 1.43) | 1.04 (0.72, 1.43) |
| **Behshahr** | 0.2 (0.1, 0.34) | 0.78 (0.51, 1.14) | 1.04 (0.72, 1.42) |
| **Shahrud** | 0.41 (0.24, 0.65) | 0.81 (0.53, 1.15) | 1.04 (0.73, 1.4) |
| **Aran va Bidgol** | 0.27 (0.13, 0.49) | 0.54 (0.29, 0.91) | 1.03 (0.61, 1.58) |
| **Genaveh** | 0.34 (0.15, 0.67) | 0.72 (0.38, 1.19) | 1.03 (0.6, 1.6) |
| **Deylam** | 0.3 (0.11, 0.65) | 0.74 (0.29, 1.6) | 1.03 (0.47, 1.88) |
| **Omidiyeh** | 0.27 (0.13, 0.49) | 0.72 (0.38, 1.2) | 1.02 (0.59, 1.59) |
| **Kordkuy** | 0.22 (0.11, 0.4) | 0.56 (0.29, 0.95) | 1.01 (0.58, 1.58) |
| **Shahrekord** | 0.21 (0.12, 0.34) | 0.59 (0.39, 0.82) | 1 (0.75, 1.29) |
| **Astanehye Ashrafiyeh** | 0.19 (0.09, 0.33) | 0.57 (0.32, 0.91) | 0.99 (0.63, 1.45) |
| **Sari** | 0.18 (0.1, 0.29) | 1.07 (0.82, 1.35) | 0.99 (0.78, 1.23) |
| **Behbahan** | 0.31 (0.17, 0.53) | 0.78 (0.48, 1.16) | 0.99 (0.65, 1.41) |
| **Ramhormoz** | 0.27 (0.12, 0.52) | 0.49 (0.27, 0.79) | 0.99 (0.64, 1.45) |
| **Fasa** | 0.29 (0.16, 0.48) | 0.95 (0.61, 1.36) | 0.99 (0.66, 1.39) |
| **Dashte Azadegan** | 0.24 (0.11, 0.43) | 0.86 (0.48, 1.36) | 0.98 (0.58, 1.51) |
| **Zarand** | 0.23 (0.11, 0.41) | 0.38 (0.21, 0.64) | 0.98 (0.62, 1.44) |
| **Arsanjan** | 0.32 (0.13, 0.65) | 0.87 (0.41, 1.59) | 0.96 (0.47, 1.68) |
| **Borujerd** | 0.3 (0.17, 0.46) | 0.86 (0.61, 1.17) | 0.95 (0.69, 1.24) |
| **Shushtar** | 0.24 (0.12, 0.41) | 0.89 (0.57, 1.3) | 0.94 (0.63, 1.32) |
| **Khomeynishahr** | 0.33 (0.19, 0.53) | 0.56 (0.35, 0.83) | 0.94 (0.67, 1.26) |
| **Hamadan** | 0.22 (0.13, 0.33) | 0.98 (0.76, 1.23) | 0.94 (0.75, 1.16) |
| **Damavand** | 0.3 (0.13, 0.59) | 0.82 (0.45, 1.32) | 0.94 (0.55, 1.47) |
| **Jahrom** | 0.28 (0.12, 0.54) | 0.77 (0.49, 1.14) | 0.93 (0.62, 1.31) |
| **Sirjan** | 0.29 (0.15, 0.48) | 0.61 (0.38, 0.92) | 0.93 (0.65, 1.29) |
| **Dezful** | 0.2 (0.11, 0.34) | 0.81 (0.56, 1.11) | 0.92 (0.67, 1.22) |
| **Rafsanjan** | 0.46 (0.27, 0.71) | 0.77 (0.5, 1.08) | 0.92 (0.65, 1.25) |
| **Orumiyeh** | 0.27 (0.18, 0.39) | 0.82 (0.64, 1.01) | 0.91 (0.74, 1.1) |
| **Eqlid** | 0.26 (0.11, 0.51) | 0.55 (0.3, 0.91) | 0.9 (0.53, 1.41) |
| **Saveh** | 0.15 (0.07, 0.26) | 0.34 (0.19, 0.54) | 0.89 (0.6, 1.25) |
| **Golpayegan** | 0.22 (0.11, 0.4) | 0.54 (0.29, 0.88) | 0.89 (0.52, 1.38) |
| **Semnan** | 0.2 (0.1, 0.35) | 0.53 (0.32, 0.82) | 0.89 (0.59, 1.25) |
| **Birjand** | 0.29 (0.13, 0.56) | 0.74 (0.49, 1.05) | 0.88 (0.61, 1.2) |
| **Ashtiyan** | 0.23 (0.09, 0.49) | 0.58 (0.22, 1.23) | 0.87 (0.37, 1.69) |
| **Sadugh** | 0.28 (0.1, 0.63) | 0.7 (0.25, 1.54) | 0.87 (0.38, 1.66) |
| **Kazerun** | 0.22 (0.1, 0.42) | 0.65 (0.42, 0.95) | 0.86 (0.59, 1.19) |
| **Sabzevar** | 0.21 (0.12, 0.33) | 0.53 (0.36, 0.74) | 0.84 (0.62, 1.08) |
| **Shahreza** | 0.35 (0.19, 0.57) | 0.88 (0.57, 1.28) | 0.84 (0.54, 1.2) |
| **Langrud** | 0.27 (0.14, 0.45) | 0.66 (0.4, 1) | 0.83 (0.53, 1.21) |
| **Mahalat** | 0.23 (0.1, 0.46) | 0.62 (0.3, 1.08) | 0.82 (0.43, 1.36) |
| **Qasreshirin** | 0.25 (0.1, 0.51) | 0.61 (0.26, 1.19) | 0.82 (0.36, 1.57) |
| **Bandare Gaz** | 0.3 (0.14, 0.55) | 0.83 (0.43, 1.41) | 0.82 (0.44, 1.36) |
| **Chalus** | 0.16 (0.07, 0.31) | 0.35 (0.19, 0.6) | 0.81 (0.49, 1.22) |
| **Oshnaviyeh** | 0.26 (0.11, 0.52) | 0.57 (0.28, 1.02) | 0.81 (0.42, 1.36) |
| **Faridan** | 0.34 (0.17, 0.61) | 0.73 (0.4, 1.18) | 0.81 (0.46, 1.3) |
| **Amol** | 0.23 (0.13, 0.36) | 0.78 (0.55, 1.06) | 0.8 (0.58, 1.06) |
| **Qaemshahr** | 0.26 (0.12, 0.51) | 0.6 (0.4, 0.85) | 0.8 (0.57, 1.07) |
| **Siyahkal** | 0.21 (0.09, 0.42) | 0.62 (0.31, 1.05) | 0.79 (0.43, 1.31) |
| **Tabas** | 0.23 (0.09, 0.45) | 0.55 (0.27, 0.97) | 0.78 (0.41, 1.3) |
| **Andimeshk** | 0.24 (0.12, 0.42) | 0.71 (0.42, 1.11) | 0.77 (0.47, 1.15) |
| **Falavarjan** | 0.25 (0.13, 0.41) | 0.5 (0.3, 0.76) | 0.77 (0.51, 1.1) |
| **Karaj** | 0.08 (0.05, 0.12) | 0.41 (0.32, 0.51) | 0.77 (0.66, 0.89) |
| **Masjedsoleyman** | 0.22 (0.11, 0.38) | 0.55 (0.31, 0.87) | 0.76 (0.46, 1.15) |
| **Boyerahmad** | 0.19 (0.08, 0.37) | 0.39 (0.21, 0.65) | 0.76 (0.47, 1.11) |
| **Tonekabon** | 0.18 (0.09, 0.31) | 0.58 (0.37, 0.87) | 0.75 (0.5, 1.05) |
| **Darrehgaz** | 0.18 (0.08, 0.33) | 0.4 (0.2, 0.71) | 0.75 (0.41, 1.2) |
| **Eyvan** | 0.26 (0.11, 0.53) | 0.66 (0.31, 1.18) | 0.75 (0.38, 1.33) |
| **Mehriz** | 0.34 (0.16, 0.62) | 0.75 (0.39, 1.3) | 0.75 (0.38, 1.29) |
| **Azadshahr** | 0.18 (0.08, 0.35) | 0.45 (0.19, 0.89) | 0.75 (0.41, 1.22) |
| **Astara** | 0.17 (0.07, 0.33) | 0.36 (0.17, 0.65) | 0.74 (0.4, 1.21) |
| **Estahban** | 0.25 (0.11, 0.47) | 0.68 (0.35, 1.17) | 0.73 (0.39, 1.25) |
| **Marvdasht** | 0.2 (0.09, 0.39) | 0.42 (0.25, 0.64) | 0.73 (0.5, 1.01) |
| **Poldokhtar** | 0.17 (0.07, 0.33) | 0.42 (0.17, 0.85) | 0.73 (0.38, 1.22) |
| **Bafq** | 0.3 (0.14, 0.56) | 0.6 (0.3, 1.08) | 0.73 (0.38, 1.23) |
| **Aliabad** | 0.2 (0.1, 0.36) | 0.54 (0.29, 0.9) | 0.73 (0.43, 1.13) |
| **Naqadeh** | 0.18 (0.09, 0.34) | 0.38 (0.2, 0.64) | 0.72 (0.42, 1.11) |
| **Sepidan** | 0.15 (0.06, 0.3) | 0.38 (0.19, 0.69) | 0.72 (0.4, 1.18) |
| **Lamard** | 0.23 (0.1, 0.45) | 0.5 (0.25, 0.87) | 0.72 (0.39, 1.18) |
| **Shahrebabak** | 0.25 (0.11, 0.49) | 0.5 (0.26, 0.85) | 0.72 (0.4, 1.16) |
| **Ardestan** | 0.19 (0.08, 0.39) | 0.59 (0.29, 1.06) | 0.72 (0.36, 1.25) |
| **Abarkuh** | 0.28 (0.12, 0.56) | 0.74 (0.35, 1.31) | 0.72 (0.37, 1.24) |
| **Ardebil** | 0.13 (0.07, 0.21) | 0.47 (0.31, 0.65) | 0.72 (0.53, 0.94) |
| **Shadegan** | 0.38 (0.19, 0.66) | 0.62 (0.34, 1.02) | 0.71 (0.41, 1.12) |
| **Farashband** | 0.22 (0.09, 0.46) | 0.52 (0.22, 1.07) | 0.71 (0.34, 1.26) |
| **Garmsar** | 0.28 (0.13, 0.49) | 0.51 (0.27, 0.86) | 0.71 (0.39, 1.13) |
| **Meybod** | 0.3 (0.14, 0.55) | 0.62 (0.32, 1.06) | 0.71 (0.39, 1.18) |
| **Bandarabbas** | 0.25 (0.14, 0.4) | 0.6 (0.41, 0.83) | 0.7 (0.5, 0.92) |
| **Bandar-e Lengeh** | 0.16 (0.08, 0.29) | 0.33 (0.18, 0.56) | 0.7 (0.42, 1.07) |
| **Bastak** | 0.17 (0.07, 0.34) | 0.34 (0.15, 0.65) | 0.7 (0.35, 1.19) |
| **Qazvin** | 0.69 (0.49, 0.91) | 0.61 (0.45, 0.8) | 0.7 (0.54, 0.88) |
| **Darab** | 0.25 (0.11, 0.49) | 0.64 (0.37, 0.98) | 0.69 (0.42, 1.04) |
| **Firuzabad** | 0.24 (0.1, 0.46) | 0.58 (0.32, 0.95) | 0.69 (0.4, 1.08) |
| **Neka** | 0.18 (0.08, 0.37) | 0.53 (0.28, 0.88) | 0.68 (0.38, 1.06) |
| **Shush** | 0.22 (0.11, 0.39) | 0.51 (0.29, 0.82) | 0.68 (0.41, 1.03) |
| **Shahriyar** | 0.17 (0.08, 0.33) | 0.38 (0.27, 0.52) | 0.68 (0.54, 0.83) |
| **Amlash** | 0.19 (0.08, 0.39) | 0.44 (0.21, 0.81) | 0.67 (0.34, 1.15) |
| **Babolsar** | 0.23 (0.12, 0.39) | 0.69 (0.43, 1.05) | 0.67 (0.42, 0.97) |
| **Gachsaran** | 0.21 (0.09, 0.4) | 0.38 (0.2, 0.63) | 0.67 (0.39, 1.04) |
| **Ardakan** | 0.27 (0.12, 0.52) | 0.55 (0.28, 0.96) | 0.67 (0.36, 1.12) |
| **Khatam** | 0.23 (0.09, 0.48) | 0.54 (0.21, 1.14) | 0.67 (0.29, 1.27) |
| **Torbate Jam** | 0.23 (0.12, 0.41) | 0.47 (0.27, 0.73) | 0.66 (0.42, 0.97) |
| **Mobarakeh** | 0.23 (0.11, 0.41) | 0.61 (0.35, 0.97) | 0.66 (0.39, 1.02) |
| **Varamin** | 0.15 (0.09, 0.25) | 0.68 (0.48, 0.92) | 0.66 (0.48, 0.86) |
| **Shazand** | 0.15 (0.06, 0.29) | 0.41 (0.21, 0.69) | 0.65 (0.38, 1.02) |
| **Bonab** | 0.17 (0.08, 0.31) | 0.6 (0.34, 0.95) | 0.65 (0.37, 1.01) |
| **Kashmar** | 0.21 (0.11, 0.36) | 0.6 (0.36, 0.91) | 0.65 (0.41, 0.95) |
| **Nayin** | 0.23 (0.11, 0.45) | 0.51 (0.26, 0.89) | 0.65 (0.33, 1.09) |
| **Khorramdarreh** | 0.2 (0.09, 0.39) | 0.42 (0.2, 0.77) | 0.65 (0.33, 1.1) |
| **Marivan** | 0.23 (0.11, 0.42) | 0.37 (0.19, 0.63) | 0.64 (0.38, 1) |
| **Eslamshahr** | 0.18 (0.08, 0.35) | 0.33 (0.2, 0.49) | 0.64 (0.46, 0.86) |
| **Tavalesh** | 0.14 (0.07, 0.26) | 0.36 (0.2, 0.59) | 0.63 (0.39, 0.93) |
| **Rudbar** | 0.32 (0.16, 0.56) | 0.43 (0.23, 0.71) | 0.63 (0.36, 0.99) |
| **Sarakhs** | 0.19 (0.08, 0.39) | 0.54 (0.27, 0.95) | 0.63 (0.32, 1.07) |
| **Mehran** | 0.22 (0.09, 0.45) | 0.54 (0.22, 1.11) | 0.63 (0.29, 1.15) |
| **Kangan** | 0.23 (0.1, 0.48) | 0.48 (0.23, 0.88) | 0.63 (0.32, 1.08) |
| **Zanjan** | 0.41 (0.26, 0.6) | 0.62 (0.43, 0.85) | 0.63 (0.45, 0.84) |
| **Meshginshahr** | 0.19 (0.09, 0.33) | 0.72 (0.43, 1.1) | 0.63 (0.37, 0.95) |
| **Shaft** | 0.16 (0.07, 0.32) | 0.35 (0.17, 0.63) | 0.62 (0.32, 1.05) |
| **Juybar** | 0.22 (0.09, 0.44) | 0.77 (0.41, 1.29) | 0.62 (0.33, 1.06) |
| **Izeh** | 0.15 (0.07, 0.28) | 0.38 (0.2, 0.62) | 0.62 (0.37, 0.94) |
| **Kalat** | 0.2 (0.07, 0.43) | 0.5 (0.18, 1.09) | 0.62 (0.23, 1.35) |
| **Khalkhal** | 0.19 (0.07, 0.43) | 0.47 (0.17, 1.08) | 0.62 (0.22, 1.41) |
| **Qom** | 0.44 (0.31, 0.59) | 0.56 (0.42, 0.71) | 0.62 (0.49, 0.77) |
| **Lali** | 0.22 (0.08, 0.48) | 0.56 (0.2, 1.22) | 0.61 (0.25, 1.21) |
| **Quchan** | 0.15 (0.07, 0.26) | 0.41 (0.23, 0.66) | 0.61 (0.37, 0.91) |
| **Baghmalek** | 0.16 (0.07, 0.32) | 0.44 (0.22, 0.78) | 0.6 (0.32, 1.02) |
| **Gonabad** | 0.22 (0.1, 0.44) | 0.56 (0.31, 0.9) | 0.6 (0.34, 0.95) |
| **Natanz** | 0.2 (0.09, 0.39) | 0.51 (0.21, 1.01) | 0.6 (0.29, 1.05) |
| **Rezvanshahr** | 0.17 (0.07, 0.35) | 0.52 (0.25, 0.91) | 0.59 (0.3, 1.01) |
| **Mahmudabad** | 0.21 (0.1, 0.38) | 0.51 (0.27, 0.86) | 0.59 (0.33, 0.95) |
| **Kangavar** | 0.19 (0.09, 0.34) | 0.39 (0.2, 0.68) | 0.59 (0.32, 0.98) |
| **Khorrambid** | 0.21 (0.08, 0.43) | 0.52 (0.21, 1.08) | 0.59 (0.28, 1.06) |
| **Semirom** | 0.2 (0.09, 0.38) | 0.47 (0.23, 0.84) | 0.59 (0.3, 1.02) |
| **Marand** | 0.33 (0.19, 0.53) | 1.23 (0.86, 1.65) | 0.58 (0.37, 0.83) |
| **Haris** | 0.19 (0.08, 0.38) | 0.46 (0.23, 0.81) | 0.58 (0.3, 1.01) |
| **Sonqor** | 0.17 (0.07, 0.33) | 0.39 (0.2, 0.66) | 0.58 (0.32, 0.95) |
| **Taft** | 0.2 (0.09, 0.41) | 0.42 (0.2, 0.74) | 0.58 (0.3, 0.99) |
| **Sumehsara** | 0.3 (0.16, 0.51) | 0.72 (0.44, 1.1) | 0.57 (0.34, 0.88) |
| **Mohr** | 0.18 (0.07, 0.38) | 0.45 (0.18, 0.95) | 0.57 (0.27, 1.03) |
| **Azna** | 0.15 (0.06, 0.3) | 0.32 (0.15, 0.59) | 0.57 (0.29, 0.98) |
| **Neyriz** | 0.22 (0.1, 0.42) | 0.57 (0.31, 0.94) | 0.56 (0.31, 0.9) |
| **Robatkarim** | 0.11 (0.05, 0.21) | 0.19 (0.1, 0.3) | 0.56 (0.4, 0.75) |
| **Fuman** | 0.27 (0.13, 0.47) | 0.51 (0.28, 0.82) | 0.55 (0.31, 0.89) |
| **Sarab** | 0.15 (0.07, 0.27) | 0.77 (0.45, 1.18) | 0.55 (0.31, 0.86) |
| **Miyandoab** | 0.15 (0.08, 0.27) | 0.32 (0.18, 0.5) | 0.55 (0.35, 0.81) |
| **Borujen** | 0.17 (0.08, 0.33) | 0.38 (0.2, 0.65) | 0.55 (0.31, 0.88) |
| **Sarayan** | 0.19 (0.07, 0.41) | 0.43 (0.16, 0.95) | 0.55 (0.2, 1.2) |
| **Harsin** | 0.17 (0.08, 0.32) | 0.35 (0.17, 0.61) | 0.54 (0.28, 0.92) |
| **Selseleh** | 0.16 (0.05, 0.38) | 0.41 (0.14, 0.95) | 0.54 (0.18, 1.26) |
| **Masal** | 0.18 (0.07, 0.36) | 0.45 (0.21, 0.81) | 0.53 (0.26, 0.93) |
| **Azarshahr** | 0.15 (0.06, 0.3) | 0.39 (0.2, 0.68) | 0.53 (0.29, 0.87) |
| **Khansar** | 0.2 (0.09, 0.38) | 0.44 (0.21, 0.8) | 0.53 (0.26, 0.94) |
| **Malayer** | 0.17 (0.09, 0.29) | 0.39 (0.24, 0.58) | 0.53 (0.35, 0.75) |
| **Bojnurd** | 0.18 (0.08, 0.36) | 0.38 (0.23, 0.58) | 0.53 (0.35, 0.77) |
| **Baft** | 0.17 (0.08, 0.31) | 0.45 (0.25, 0.74) | 0.52 (0.3, 0.82) |
| **Shirvan** | 0.14 (0.06, 0.28) | 0.29 (0.15, 0.5) | 0.52 (0.3, 0.81) |
| **Khomeyn** | 0.14 (0.07, 0.26) | 0.37 (0.2, 0.61) | 0.51 (0.28, 0.81) |
| **Mamasany** | 0.12 (0.06, 0.22) | 0.38 (0.2, 0.61) | 0.51 (0.3, 0.8) |
| **Baneh** | 0.14 (0.06, 0.28) | 0.32 (0.16, 0.57) | 0.51 (0.27, 0.84) |
| **Pakdasht** | 0.12 (0.06, 0.23) | 0.27 (0.14, 0.46) | 0.51 (0.31, 0.77) |
| **Firuzkuh** | 0.15 (0.06, 0.32) | 0.38 (0.15, 0.8) | 0.51 (0.2, 1.08) |
| **Torkman** | 0.2 (0.09, 0.4) | 0.47 (0.25, 0.77) | 0.51 (0.29, 0.82) |
| **Jolfa** | 0.15 (0.06, 0.31) | 0.37 (0.17, 0.7) | 0.5 (0.2, 1.05) |
| **Paveh** | 0.16 (0.07, 0.34) | 0.36 (0.16, 0.67) | 0.5 (0.24, 0.88) |
| **Ravar** | 0.22 (0.08, 0.46) | 0.5 (0.2, 1.06) | 0.5 (0.21, 0.93) |
| **Nahavand** | 0.14 (0.06, 0.27) | 0.29 (0.16, 0.48) | 0.5 (0.3, 0.77) |
| **Dashti** | 0.2 (0.08, 0.39) | 0.47 (0.23, 0.83) | 0.5 (0.25, 0.87) |
| **Dayyer** | 0.21 (0.09, 0.43) | 0.56 (0.26, 1.01) | 0.5 (0.24, 0.9) |
| **Takestan** | 0.19 (0.09, 0.34) | 0.41 (0.23, 0.66) | 0.5 (0.29, 0.78) |
| **Sarpole Zahab** | 0.16 (0.07, 0.33) | 0.45 (0.22, 0.8) | 0.49 (0.24, 0.85) |
| **Jiroft** | 0.17 (0.09, 0.32) | 0.4 (0.22, 0.65) | 0.49 (0.29, 0.74) |
| **Fariman** | 0.19 (0.08, 0.37) | 0.44 (0.22, 0.78) | 0.49 (0.25, 0.84) |
| **Dorud** | 0.2 (0.1, 0.35) | 0.36 (0.19, 0.6) | 0.49 (0.28, 0.78) |
| **Dehloran** | 0.17 (0.07, 0.35) | 0.38 (0.16, 0.75) | 0.49 (0.22, 0.91) |
| **Tiran va Karvan** | 0.15 (0.06, 0.3) | 0.38 (0.15, 0.78) | 0.48 (0.23, 0.85) |
| **Qorveh** | 0.12 (0.06, 0.22) | 0.29 (0.16, 0.48) | 0.48 (0.28, 0.74) |
| **Bahar** | 0.16 (0.08, 0.3) | 0.47 (0.25, 0.76) | 0.48 (0.26, 0.78) |
| **Abyek** | 0.16 (0.07, 0.33) | 0.45 (0.22, 0.79) | 0.48 (0.25, 0.82) |
| **Komijan** | 0.15 (0.06, 0.34) | 0.36 (0.14, 0.73) | 0.47 (0.2, 0.91) |
| **Hashtrud** | 0.15 (0.06, 0.28) | 0.51 (0.25, 0.91) | 0.47 (0.23, 0.84) |
| **Salmas** | 0.21 (0.1, 0.37) | 0.7 (0.42, 1.06) | 0.47 (0.27, 0.75) |
| **Bovanat** | 0.16 (0.07, 0.34) | 0.41 (0.17, 0.84) | 0.47 (0.22, 0.87) |
| **Zarrindasht** | 0.2 (0.07, 0.43) | 0.48 (0.18, 1.03) | 0.47 (0.21, 0.87) |
| **Tuyserkan** | 0.17 (0.08, 0.31) | 0.36 (0.19, 0.61) | 0.47 (0.26, 0.76) |
| **Asadabad** | 0.15 (0.07, 0.3) | 0.33 (0.17, 0.58) | 0.47 (0.25, 0.77) |
| **Gonbade Kavus** | 0.22 (0.12, 0.36) | 0.68 (0.44, 0.98) | 0.47 (0.3, 0.69) |
| **Qirokarzin** | 0.17 (0.07, 0.35) | 0.36 (0.16, 0.68) | 0.46 (0.22, 0.84) |
| **Fereydunshahr** | 0.16 (0.07, 0.33) | 0.42 (0.19, 0.79) | 0.46 (0.21, 0.88) |
| **Aligudarz** | 0.14 (0.06, 0.29) | 0.36 (0.16, 0.73) | 0.46 (0.25, 0.75) |
| **Tangestan** | 0.2 (0.08, 0.41) | 0.47 (0.22, 0.84) | 0.46 (0.22, 0.82) |
| **Ajabshir** | 0.17 (0.07, 0.33) | 0.39 (0.16, 0.78) | 0.45 (0.22, 0.8) |
| **Maragheh** | 0.16 (0.07, 0.32) | 0.59 (0.37, 0.88) | 0.44 (0.27, 0.67) |
| **Shahindezh** | 0.14 (0.06, 0.29) | 0.37 (0.18, 0.67) | 0.44 (0.18, 0.92) |
| **Javanrud** | 0.13 (0.05, 0.26) | 0.33 (0.14, 0.67) | 0.44 (0.22, 0.75) |
| **Bardsir** | 0.16 (0.07, 0.31) | 0.42 (0.2, 0.74) | 0.44 (0.21, 0.78) |
| **Farsan** | 0.19 (0.09, 0.36) | 0.35 (0.17, 0.64) | 0.44 (0.22, 0.76) |
| **Minudasht** | 0.14 (0.06, 0.26) | 0.33 (0.16, 0.57) | 0.44 (0.23, 0.72) |
| **Sarbisheh** | 0.15 (0.04, 0.4) | 0.37 (0.11, 0.94) | 0.44 (0.13, 1.13) |
| **Ferdows** | 0.18 (0.08, 0.36) | 0.47 (0.23, 0.81) | 0.44 (0.22, 0.77) |
| **Sahneh** | 0.16 (0.07, 0.32) | 0.37 (0.18, 0.66) | 0.43 (0.22, 0.74) |
| **Chadegan** | 0.15 (0.06, 0.32) | 0.37 (0.14, 0.78) | 0.43 (0.18, 0.81) |
| **Saqqez** | 0.17 (0.08, 0.3) | 0.42 (0.24, 0.66) | 0.43 (0.26, 0.66) |
| **Zarandiyeh** | 0.14 (0.06, 0.29) | 0.36 (0.15, 0.74) | 0.42 (0.2, 0.77) |
| **Torbate Heydarieh** | 0.3 (0.17, 0.48) | 0.45 (0.28, 0.67) | 0.42 (0.27, 0.62) |
| **Tafresh** | 0.17 (0.07, 0.34) | 0.42 (0.18, 0.85) | 0.41 (0.2, 0.72) |
| **Savadkuh** | 0.16 (0.07, 0.33) | 0.4 (0.17, 0.82) | 0.41 (0.2, 0.72) |
| **Khoy** | 0.13 (0.06, 0.21) | 0.29 (0.18, 0.45) | 0.41 (0.26, 0.59) |
| **Bukan** | 0.13 (0.06, 0.23) | 0.37 (0.2, 0.6) | 0.41 (0.24, 0.65) |
| **Chenaran** | 0.14 (0.06, 0.28) | 0.37 (0.16, 0.75) | 0.41 (0.22, 0.69) |
| **Germi** | 0.13 (0.06, 0.26) | 0.31 (0.15, 0.56) | 0.41 (0.2, 0.71) |
| **Qaenat** | 0.13 (0.06, 0.26) | 0.31 (0.14, 0.63) | 0.41 (0.22, 0.67) |
| **Bostanabad** | 0.14 (0.06, 0.28) | 0.35 (0.17, 0.61) | 0.4 (0.2, 0.69) |
| **Piranshahr** | 0.14 (0.06, 0.28) | 0.33 (0.16, 0.6) | 0.4 (0.2, 0.68) |
| **Razan** | 0.12 (0.05, 0.25) | 0.27 (0.13, 0.48) | 0.4 (0.21, 0.67) |
| **Abdanan** | 0.14 (0.06, 0.29) | 0.33 (0.15, 0.64) | 0.4 (0.19, 0.75) |
| **Dashtestan** | 0.22 (0.1, 0.43) | 0.5 (0.3, 0.78) | 0.4 (0.24, 0.62) |
| **Miyaneh** | 0.13 (0.06, 0.23) | 0.39 (0.23, 0.62) | 0.39 (0.22, 0.6) |
| **Jam** | 0.14 (0.05, 0.31) | 0.35 (0.13, 0.77) | 0.39 (0.16, 0.78) |
| **Qeshm** | 0.19 (0.08, 0.38) | 0.51 (0.25, 0.9) | 0.39 (0.19, 0.68) |
| **Minab** | 0.13 (0.06, 0.23) | 0.32 (0.18, 0.53) | 0.39 (0.23, 0.61) |
| **Mahabad** | 0.14 (0.06, 0.28) | 0.34 (0.18, 0.55) | 0.38 (0.22, 0.6) |
| **Kahnuj** | 0.11 (0.05, 0.21) | 0.27 (0.14, 0.46) | 0.38 (0.21, 0.59) |
| **Kowsar** | 0.12 (0.04, 0.29) | 0.29 (0.09, 0.71) | 0.38 (0.11, 0.95) |
| **Shabestar** | 0.16 (0.07, 0.31) | 0.57 (0.33, 0.9) | 0.37 (0.2, 0.62) |
| **Malekan** | 0.12 (0.05, 0.24) | 0.3 (0.15, 0.54) | 0.37 (0.19, 0.65) |
| **Salas-e-Babajani** | 0.1 (0.03, 0.25) | 0.27 (0.09, 0.62) | 0.37 (0.12, 0.86) |
| **Taybad** | 0.13 (0.06, 0.25) | 0.41 (0.21, 0.71) | 0.37 (0.19, 0.63) |
| **Shirvan va Chardavol** | 0.11 (0.04, 0.23) | 0.26 (0.11, 0.49) | 0.37 (0.15, 0.77) |
| **Abhar** | 0.14 (0.07, 0.25) | 0.32 (0.16, 0.54) | 0.37 (0.2, 0.6) |
| **Bilehsowar** | 0.12 (0.05, 0.25) | 0.28 (0.12, 0.55) | 0.37 (0.16, 0.71) |
| **Neer** | 0.11 (0.04, 0.26) | 0.28 (0.1, 0.64) | 0.37 (0.13, 0.83) |
| **Chaldoran** | 0.13 (0.04, 0.29) | 0.31 (0.12, 0.63) | 0.36 (0.14, 0.71) |
| **Anbarabad** | 0.15 (0.05, 0.33) | 0.34 (0.12, 0.77) | 0.36 (0.15, 0.71) |
| **Manujan** | 0.11 (0.04, 0.25) | 0.29 (0.11, 0.59) | 0.36 (0.13, 0.8) |
| **Kuhdasht** | 0.13 (0.06, 0.24) | 0.25 (0.13, 0.43) | 0.36 (0.2, 0.58) |
| **Khaf** | 0.14 (0.06, 0.28) | 0.35 (0.15, 0.7) | 0.35 (0.17, 0.62) |
| **Zabol** | 0.11 (0.05, 0.19) | 0.39 (0.24, 0.6) | 0.35 (0.21, 0.55) |
| **Kohgiluyeh** | 0.1 (0.04, 0.21) | 0.23 (0.11, 0.4) | 0.35 (0.14, 0.71) |
| **Tarom** | 0.11 (0.04, 0.26) | 0.28 (0.1, 0.63) | 0.35 (0.14, 0.71) |
| **Mahneshan** | 0.11 (0.04, 0.24) | 0.28 (0.1, 0.63) | 0.35 (0.12, 0.78) |
| **Rudan** | 0.11 (0.04, 0.22) | 0.25 (0.11, 0.47) | 0.35 (0.17, 0.62) |
| **Namin** | 0.12 (0.05, 0.25) | 0.35 (0.16, 0.64) | 0.35 (0.16, 0.65) |
| **Iranshahr** | 0.17 (0.07, 0.33) | 0.39 (0.21, 0.65) | 0.34 (0.18, 0.55) |
| **Divandarreh** | 0.12 (0.05, 0.23) | 0.29 (0.13, 0.53) | 0.34 (0.17, 0.62) |
| **Kamyaran** | 0.12 (0.05, 0.24) | 0.3 (0.12, 0.61) | 0.34 (0.17, 0.6) |
| **Dena** | 0.13 (0.05, 0.26) | 0.31 (0.13, 0.62) | 0.34 (0.15, 0.64) |
| **Parsabad** | 0.11 (0.05, 0.21) | 0.26 (0.13, 0.45) | 0.34 (0.18, 0.57) |
| **Kalaleh** | 0.11 (0.05, 0.22) | 0.25 (0.12, 0.45) | 0.34 (0.17, 0.57) |
| **Nehbandan** | 0.11 (0.04, 0.26) | 0.27 (0.09, 0.65) | 0.34 (0.11, 0.82) |
| **Nur** | 0.16 (0.07, 0.31) | 0.45 (0.24, 0.75) | 0.33 (0.17, 0.57) |
| **Gilanegharb** | 0.12 (0.05, 0.25) | 0.31 (0.14, 0.58) | 0.33 (0.15, 0.62) |
| **Ardal** | 0.11 (0.04, 0.25) | 0.26 (0.09, 0.62) | 0.33 (0.11, 0.77) |
| **Dalfan** | 0.12 (0.05, 0.24) | 0.29 (0.12, 0.58) | 0.33 (0.17, 0.58) |
| **Maneh va Semelqan** | 0.13 (0.05, 0.27) | 0.31 (0.12, 0.68) | 0.33 (0.15, 0.62) |
| **Noshahr** | 0.12 (0.05, 0.23) | 0.27 (0.13, 0.47) | 0.32 (0.17, 0.55) |
| **Kalibar** | 0.1 (0.04, 0.2) | 0.26 (0.12, 0.49) | 0.32 (0.13, 0.67) |
| **Varzaqan** | 0.11 (0.04, 0.22) | 0.26 (0.11, 0.55) | 0.32 (0.14, 0.6) |
| **Rashtkhar** | 0.12 (0.04, 0.27) | 0.29 (0.1, 0.66) | 0.32 (0.13, 0.65) |
| **Ijrud** | 0.1 (0.04, 0.22) | 0.27 (0.1, 0.57) | 0.32 (0.13, 0.7) |
| **Osku** | 0.12 (0.05, 0.24) | 0.29 (0.14, 0.52) | 0.31 (0.15, 0.54) |
| **Charoymaq** | 0.09 (0.04, 0.2) | 0.25 (0.1, 0.51) | 0.31 (0.12, 0.66) |
| **Bam** | 0.18 (0.09, 0.31) | 0.35 (0.19, 0.56) | 0.31 (0.18, 0.49) |
| **Bijar** | 0.12 (0.05, 0.22) | 0.3 (0.15, 0.53) | 0.31 (0.16, 0.54) |
| **Darrehshahr** | 0.11 (0.04, 0.23) | 0.27 (0.1, 0.59) | 0.31 (0.13, 0.62) |
| **Esfarayen** | 0.11 (0.05, 0.22) | 0.22 (0.1, 0.4) | 0.31 (0.16, 0.53) |
| **Nazarabad** | 0.15 (0.06, 0.32) | 0.28 (0.13, 0.5) | 0.31 (0.15, 0.55) |
| **Ahar** | 0.12 (0.06, 0.23) | 0.5 (0.28, 0.82) | 0.3 (0.16, 0.5) |
| **Neyshabur** | 0.28 (0.17, 0.42) | 0.46 (0.31, 0.66) | 0.3 (0.19, 0.44) |
| **Bardeskan** | 0.11 (0.04, 0.23) | 0.28 (0.13, 0.53) | 0.3 (0.14, 0.55) |
| **Lordakan** | 0.14 (0.06, 0.26) | 0.29 (0.13, 0.58) | 0.3 (0.16, 0.51) |
| **Jajarm** | 0.11 (0.04, 0.25) | 0.27 (0.1, 0.6) | 0.3 (0.12, 0.59) |
| **Sardasht** | 0.09 (0.04, 0.19) | 0.22 (0.1, 0.41) | 0.29 (0.14, 0.54) |
| **Kuhrang** | 0.1 (0.04, 0.21) | 0.24 (0.09, 0.51) | 0.29 (0.11, 0.62) |
| **Bandar-e-Jask** | 0.14 (0.07, 0.26) | 0.28 (0.13, 0.52) | 0.29 (0.15, 0.51) |
| **Hajiabad** | 0.12 (0.05, 0.25) | 0.35 (0.15, 0.67) | 0.29 (0.13, 0.57) |
| **Takab** | 0.11 (0.05, 0.23) | 0.25 (0.11, 0.47) | 0.28 (0.13, 0.52) |
| **Sarvabad** | 0.09 (0.03, 0.19) | 0.22 (0.09, 0.47) | 0.28 (0.11, 0.59) |
| **Bueenzahra** | 0.1 (0.05, 0.19) | 0.21 (0.1, 0.37) | 0.28 (0.14, 0.48) |
| **Aqqala** | 0.11 (0.05, 0.22) | 0.28 (0.11, 0.56) | 0.28 (0.13, 0.51) |
| **Maku** | 0.1 (0.04, 0.18) | 0.25 (0.13, 0.44) | 0.27 (0.14, 0.46) |
| **Khash** | 0.11 (0.04, 0.24) | 0.22 (0.1, 0.41) | 0.27 (0.13, 0.5) |
| **Ramyan** | 0.11 (0.05, 0.22) | 0.26 (0.1, 0.54) | 0.27 (0.12, 0.5) |
| **Sarbaz** | 0.1 (0.03, 0.26) | 0.21 (0.06, 0.54) | 0.25 (0.07, 0.64) |
| **Faruj** | 0.1 (0.04, 0.21) | 0.23 (0.09, 0.51) | 0.25 (0.11, 0.5) |
| **Chah Bahar** | 0.12 (0.05, 0.24) | 0.25 (0.11, 0.51) | 0.24 (0.12, 0.41) |
| **Kabudarahang** | 0.1 (0.05, 0.19) | 0.21 (0.1, 0.37) | 0.24 (0.12, 0.43) |
| **Khodabandeh** | 0.1 (0.05, 0.2) | 0.25 (0.13, 0.44) | 0.24 (0.12, 0.42) |
| **Eslamabade Gharb** | 0.11 (0.05, 0.19) | 0.28 (0.15, 0.46) | 0.23 (0.12, 0.39) |
| **Nikshahr** | 0.09 (0.03, 0.18) | 0.16 (0.07, 0.3) | 0.2 (0.09, 0.36) |
| **Saravan** | 0.08 (0.03, 0.16) | 0.16 (0.08, 0.29) | 0.17 (0.08, 0.3) |
| **Savojbolagh** | 0.05 (0.02, 0.11) | 0.11 (0.04, 0.23) | 0.11 (0.05, 0.2) |
