# Supplementary material for: Spatiotemporal disparity of breast cancer incidence in Iranian female populations at the district level from 2000 to 2021: Bayesian disease mapping
Source: PLoS One. 2025 Sep 11;20(9):e0330017. doi: 10.1371/journal.pone.0330017 (PMC12425319; doi:10.1371/journal.pone.0330017)
Supplement: S4 Table — (DOCX) [file pone.0330017.s013.docx]

**S4 Table. National average RR of breast cancer incidence from 2000 to 2021 (based on all posterior samples).**

| **Year** | **National RR (95% CrI)** |
| --- | --- |
| **2000** | 0.21 (0.19, 0.22) |
| **2001** | 0.23 (0.21, 0.25) |
| **2002** | 0.31 (0.30, 0.33) |
| **2003** | 0.38 (0.36, 0.40) |
| **2004** | 0.41 (0.39, 0.43) |
| **2005** | 0.52 (0.49, 0.54) |
| **2006** | 0.55 (0.47, 0.65) |
| **2007** | 0.58 (0.55, 0.60) |
| **2008** | 0.61 (0.58, 0.63) |
| **2009** | 0.65 (0.62, 0.67) |
| **2010** | 0.66 (0.63, 0.68) |
| **2011** | 0.75 (0.73, 0.78) |
| **2012** | 0.80 (0.77, 0.83) |
| **2013** | 0.85 (0.82, 0.88) |
| **2014** | 0.89 (0.86, 0.93) |
| **2015** | 0.94 (0.91, 0.98) |
| **2016** | 0.99 (0.95, 1.03) |
| **2017** | 1.04 (1.00, 1.08) |
| **2018** | 1.09 (1.05, 1.13) |
| **2019** | 1.13 (1.10, 1.18) |
| **2020** | 1.18 (1.14, 1.23) |
| **2021** | 1.23 (1.18, 1.28) |
