# Supplementary material for: Spatiotemporal disparity of breast cancer incidence in Iranian female populations at the district level from 2000 to 2021: Bayesian disease mapping
Source: PLoS One. 2025 Sep 11;20(9):e0330017. doi: 10.1371/journal.pone.0330017 (PMC12425319; doi:10.1371/journal.pone.0330017)
Supplement: S6 Table — (DOCX) [file pone.0330017.s015.docx]

**S6 Table. Results of cross-validation for the spatiotemporal model at the district level for RR of breast cancer incidence.**

**
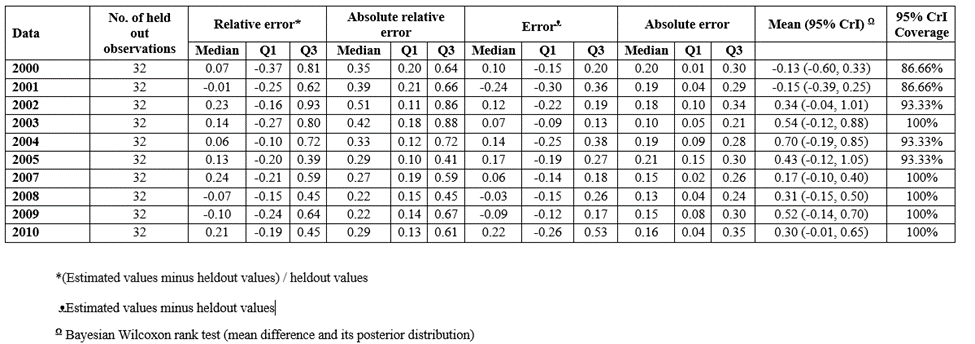
**
