# Supplementary material for: Spatiotemporal disparity of breast cancer incidence in Iranian female populations at the district level from 2000 to 2021: Bayesian disease mapping
Source: PLoS One. 2025 Sep 11;20(9):e0330017. doi: 10.1371/journal.pone.0330017 (PMC12425319; doi:10.1371/journal.pone.0330017)
Supplement: S1 Data — (PDF) [file pone.0330017.s016.pdf]

Year	District	District ID	Female Population	Relative Risk	SMR	Relative Risk lower limit	Relative Risk upper limit	Female Urbanization (%)	Wealth index	Female mean years of schooling	Cancer registery completeness
2000	Arak	1	99475	0.15	0.04	0.09	0.24	75.66	0.21	2.78	0.13
2000	Ashtiyan	2	4151	0.23	NA	0.09	0.49	29.57	0.22	2.71	0.13
2000	Tafresh	3	12993	0.17	NA	0.07	0.34	23.49	0.21	2.74	0.13
2000	Khomeyn	4	22078	0.14	0.09	0.07	0.26	46.27	0.16	2.56	0.13
2000	Delijan	5	7925	0.25	0.25	0.11	0.48	61.59	0.11	2.63	0.13
2000	Saveh	6	32680	0.15	0.06	0.07	0.26	61.73	0.25	2.71	0.13
2000	Shazand	7	22615	0.15	NA	0.06	0.29	26.30	0.15	2.75	0.13
2000	Mahalat	8	9268	0.23	NA	0.1	0.46	76.15	0.24	2.60	0.13
2000	Zarandiyeh	9	10260	0.14	NA	0.06	0.29	52.02	0.20	2.62	0.13
2000	Komijan	10	6977	0.15	NA	0.06	0.34	37.36	0.19	2.78	0.13
2000	Astara	11	12376	0.17	0.17	0.07	0.33	57.46	-0.42	2.94	0.09
2000	Astanehye Ashrafiyeh	12	24086	0.19	0.08	0.09	0.33	40.25	-0.57	3.04	0.09
2000	Bandar Anzali	13	27803	0.47	0.64	0.26	0.76	81.16	-0.45	2.95	0.09
2000	Tavalesh	14	27827	0.14	0.15	0.07	0.26	28.67	-0.58	2.95	0.09
2000	Rasht	15	159215	0.47	0.5	0.34	0.62	64.58	-0.37	3.06	0.09
2000	Rudbar	16	19914	0.32	0.69	0.16	0.56	46.06	-0.58	3.16	0.09
2000	Rudsar	17	32452	0.26	0.18	0.14	0.44	44.57	-0.53	3.06	0.09
2000	Sumehsara	18	27539	0.3	0.5	0.16	0.51	26.24	-0.55	3.20	0.09
2000	Fuman	19	21048	0.27	0.56	0.13	0.47	26.56	-0.49	3.09	0.09
2000	Langrud	20	28640	0.27	0.27	0.14	0.45	56.88	-0.55	2.95	0.09
2000	Lahijan	21	34813	0.38	0.49	0.21	0.62	38.14	-0.50	3.14	0.09
2000	Shaft	22	13795	0.16	NA	0.07	0.32	11.29	-0.42	3.08	0.09
2000	Amlash	23	10002	0.19	NA	0.08	0.39	29.61	-0.52	3.08	0.09
2000	Rezvanshahr	24	11415	0.17	NA	0.07	0.35	25.79	-0.52	2.99	0.09
2000	Siyahkal	25	10436	0.21	NA	0.09	0.42	27.65	-0.42	2.94	0.09
2000	Masal	26	8912	0.18	NA	0.07	0.36	29.96	-0.46	3.09	0.09
2000	Amol	27	61061	0.23	0.17	0.13	0.36	52.55	1.16	3.19	0.23
2000	Babol	28	85993	0.42	0.45	0.28	0.6	44.25	1.19	3.07	0.23
2000	Behshahr	29	34841	0.2	0.06	0.1	0.34	59.11	1.18	3.29	0.23
2000	Tonekabon	30	37571	0.18	0.05	0.09	0.31	45.30	1.09	3.11	0.23
2000	Ramsar	31	14200	0.29	0.14	0.14	0.52	69.65	1.13	3.28	0.23
2000	Sari	32	86056	0.18	0.07	0.1	0.29	47.41	1.13	3.20	0.23
2000	Savadkuh	33	13895	0.16	NA	0.07	0.33	44.40	1.13	3.24	0.23
2000	Qaemshahr	34	54088	0.26	NA	0.12	0.51	55.98	1.12	3.21	0.23
2000	Nur	35	19007	0.16	NA	0.07	0.31	36.73	1.14	3.19	0.23
2000	Noshahr	36	20587	0.12	NA	0.05	0.23	33.25	1.13	3.25	0.23
2000	Babolsar	37	30939	0.23	0.2	0.12	0.39	48.99	1.13	3.21	0.23
2000	Mahmudabad	38	15832	0.21	0.26	0.1	0.38	29.14	1.09	3.30	0.23
2000	Neka	39	17923	0.18	NA	0.08	0.37	32.74	1.10	3.26	0.23
2000	Chalus	40	21867	0.16	NA	0.07	0.31	51.54	1.20	3.22	0.23
2000	Juybar	41	12919	0.22	NA	0.09	0.44	38.32	0.98	3.18	0.23
2000	Ahar	42	25029	0.12	0.08	0.06	0.23	59.71	-0.58	2.06	0.14
2000	Tabriz	43	269339	0.31	0.3	0.23	0.4	93.39	-0.72	1.90	0.14
2000	Sarab	44	23682	0.15	0.08	0.07	0.27	38.83	-0.69	1.81	0.14

2000	Maragheh	45	39689	0.16	NA	0.07	0.32	67.45	-0.59	1.89	0.14
2000	Marand	46	41656	0.33	0.43	0.19	0.53	55.27	-0.66	2.02	0.14
2000	Miyaneh	47	35417	0.13	0.05	0.06	0.23	44.87	-0.64	2.01	0.14
2000	Hashtrud	48	11588	0.15	0.17	0.06	0.28	24.31	-0.69	2.18	0.14
2000	Bonab	49	20390	0.17	0.1	0.08	0.31	59.62	-0.59	1.89	0.14
2000	Bostanabad	50	15909	0.14	NA	0.06	0.28	16.47	-0.63	1.84	0.14
2000	Shabestar	51	25213	0.16	NA	0.07	0.31	45.97	-0.64	2.06	0.14
2000	Kalibar	52	14455	0.1	NA	0.04	0.2	12.71	-0.69	2.02	0.14
2000	Haris	53	11649	0.19	NA	0.08	0.38	43.88	-0.63	1.97	0.14
2000	Jolfa	54	10437	0.15	NA	0.06	0.31	58.16	-0.67	1.93	0.14
2000	Malekan	55	15850	0.12	0.13	0.05	0.24	30.71	-0.57	2.02	0.14
2000	Azarshahr	56	18342	0.15	NA	0.06	0.3	59.01	-0.67	2.06	0.14
2000	Osku	57	14011	0.12	NA	0.05	0.24	47.15	-0.66	1.97	0.14
2000	Charoymaq	58	5364	0.09	NA	0.04	0.2	10.72	-0.66	2.01	0.14
2000	Varzaqan	59	8226	0.11	NA	0.04	0.22	9.61	-0.56	1.99	0.14
2000	Ajabshir	60	11313	0.17	0.36	0.07	0.33	40.51	-0.64	1.99	0.14
2000	Orumiyeh	61	135701	0.27	0.26	0.18	0.39	67.22	-0.49	1.80	0.20
2000	Piranshahr	62	12998	0.14	NA	0.06	0.28	43.99	-0.36	1.78	0.20
2000	Khoy	63	57463	0.13	0.11	0.06	0.21	63.88	-0.42	1.88	0.20
2000	Sardasht	64	14123	0.09	NA	0.04	0.19	42.99	-0.39	1.74	0.20
2000	Salmas	65	26249	0.21	0.16	0.1	0.37	53.00	-0.39	1.91	0.20
2000	Maku	66	25280	0.1	0.08	0.04	0.18	46.33	-0.45	1.96	0.20
2000	Mahabad	67	29614	0.14	NA	0.06	0.28	67.38	-0.37	1.98	0.20
2000	Miyandoab	68	38105	0.15	0.16	0.08	0.27	48.99	-0.42	2.01	0.20
2000	Naqadeh	69	19064	0.18	0.11	0.09	0.34	65.37	-0.45	1.87	0.20
2000	Bukan	70	29129	0.13	0.14	0.06	0.23	70.60	-0.40	1.75	0.20
2000	Shahindezh	71	15030	0.14	NA	0.06	0.29	46.44	-0.46	2.03	0.20
2000	Takab	72	13768	0.11	NA	0.05	0.23	53.09	-0.46	1.82	0.20
2000	Oshnaviyeh	73	8357	0.26	NA	0.11	0.52	53.04	-0.48	1.78	0.20
2000	Chaldoran	74	6325	0.13	NA	0.04	0.29	33.24	-0.41	1.82	0.20
2000	Eslamabade Gharb	75	32512	0.11	0.06	0.05	0.19	49.45	-0.68	1.94	0.10
2000	Kermanshah	76	154854	0.37	0.38	0.26	0.51	83.74	-0.72	2.16	0.10
2000	Paveh	77	8965	0.16	NA	0.07	0.34	50.07	-0.70	1.91	0.10
2000	Sarpole Zahab	78	11884	0.16	NA	0.07	0.33	38.62	-0.71	1.97	0.10
2000	Sonqor	79	18255	0.17	NA	0.07	0.33	44.42	-0.70	1.80	0.10
2000	Qasreshirin	80	3515	0.25	NA	0.1	0.51	67.14	-0.72	1.96	0.10
2000	Kangavar	81	14318	0.19	0.14	0.09	0.34	59.43	-0.58	1.92	0.10
2000	Gilanegharb	82	10537	0.12	NA	0.05	0.25	31.11	-0.73	1.95	0.10
2000	Javanrud	83	14773	0.13	NA	0.05	0.26	49.77	-0.75	2.03	0.10
2000	Sahneh	84	14001	0.16	NA	0.07	0.32	38.71	-0.74	1.90	0.10
2000	Harsin	85	14114	0.17	0.14	0.08	0.32	64.64	-0.68	1.99	0.10
2000	Salas-e-Babajani	86	4169	0.1	NA	0.03	0.25	17.26	-0.66	2.02	0.10
2000	Abadan	87	41547	0.64	0.71	0.4	0.95	84.52	0.90	2.47	0.22
2000	Andimeshk	88	22397	0.24	0.19	0.12	0.42	74.87	0.81	2.56	0.22
2000	Ahvaz	89	181359	0.74	0.77	0.57	0.92	82.03	0.86	2.53	0.22
2000	Izeh	90	24464	0.15	0.17	0.07	0.28	47.84	0.81	2.52	0.22
2000	Bandar-e-Mahshahr	91	31314	0.37	0.34	0.2	0.62	94.14	0.77	2.49	0.22

2000	Behbahan	92	28070	0.31	0.29	0.17	0.53	71.67	0.77	2.42	0.22
2000	Khorramshahr	93	20907	0.33	0.2	0.17	0.57	83.98	0.86	2.60	0.22
2000	Dezful	94	54022	0.2	0.08	0.11	0.34	72.87	0.81	2.39	0.22
2000	Dashte Azadegan	95	16219	0.24	0.13	0.11	0.43	55.18	0.77	2.51	0.22
2000	Ramhormoz	96	23849	0.27	NA	0.12	0.52	51.11	0.79	2.52	0.22
2000	Shadegan	97	16393	0.38	0.77	0.19	0.66	37.99	0.76	2.51	0.22
2000	Shushtar	98	31798	0.24	0.13	0.12	0.41	58.97	0.86	2.44	0.22
2000	Masjedsoleyman	99	27522	0.22	0.15	0.11	0.38	65.90	0.83	2.67	0.22
2000	Shush	100	23853	0.22	0.09	0.11	0.39	45.51	0.86	2.54	0.22
2000	Baghmalek	101	12367	0.16	NA	0.07	0.32	32.68	0.85	2.65	0.22
2000	Omidiyeh	102	12160	0.27	0.17	0.13	0.49	69.25	0.79	2.60	0.22
2000	Lali	103	4541	0.22	NA	0.08	0.48	47.67	0.82	2.44	0.22
2000	Hendijan	104	5559	0.33	NA	0.13	0.69	67.79	0.78	2.37	0.22
2000	Abadeh	105	17483	0.38	NA	0.17	0.72	87.95	0.25	2.45	0.19
2000	Estahban	106	10787	0.25	0.19	0.11	0.47	71.66	0.27	2.56	0.19
2000	Eqlid	107	14319	0.26	NA	0.11	0.51	64.33	0.31	2.64	0.19
2000	Jahrom	108	33123	0.28	NA	0.12	0.54	63.56	0.30	2.44	0.19
2000	Darab	109	24626	0.25	NA	0.11	0.49	42.26	0.21	2.61	0.19
2000	Sepidan	110	12909	0.15	NA	0.06	0.3	17.73	0.22	2.53	0.19
2000	Shiraz	111	266959	0.11	0.01	0.07	0.16	87.37	0.36	2.55	0.19
2000	Fasa	112	30838	0.29	0.27	0.16	0.48	56.59	0.30	2.52	0.19
2000	Firuzabad	113	19099	0.24	NA	0.1	0.46	51.95	0.30	2.54	0.19
2000	Kazerun	114	42569	0.22	NA	0.1	0.42	51.47	0.28	2.57	0.19
2000	Lar (Larestan)	115	39422	0.32	NA	0.14	0.61	57.03	0.33	2.67	0.19
2000	Marvdasht	116	46290	0.2	NA	0.09	0.39	44.13	0.27	2.52	0.19
2000	Mamasany	117	27525	0.12	0.07	0.06	0.22	28.52	0.29	2.53	0.19
2000	Neyriz	118	15792	0.22	NA	0.1	0.42	58.26	0.31	2.53	0.19
2000	Lamard	119	10572	0.23	NA	0.1	0.45	41.83	0.27	2.51	0.19
2000	Bovanat	120	7668	0.16	NA	0.07	0.34	29.09	0.31	2.47	0.19
2000	Arsanjan	121	5737	0.32	NA	0.13	0.65	39.37	0.24	2.58	0.19
2000	Khorrambid	122	6709	0.21	NA	0.08	0.43	76.11	0.28	2.48	0.19
2000	Zarrindasht	123	7981	0.2	NA	0.07	0.43	57.75	0.35	2.50	0.19
2000	Qirokarzin	124	8602	0.17	NA	0.07	0.35	56.82	0.25	2.50	0.19
2000	Mohr	125	6826	0.18	NA	0.07	0.38	35.49	0.37	2.50	0.19
2000	Farashband	126	5918	0.22	NA	0.09	0.46	59.70	0.26	2.59	0.19
2000	Baft	127	22044	0.17	0.18	0.08	0.31	34.02	-0.24	2.47	0.73
2000	Bam	128	31833	0.18	0.2	0.09	0.31	43.61	-0.23	2.73	0.73
2000	Jiroft	129	27109	0.17	0.15	0.09	0.32	38.03	-0.25	2.61	0.73
2000	Rafsanjan	130	41671	0.46	0.6	0.27	0.71	54.06	-0.20	2.88	0.73
2000	Zarand	131	22792	0.23	0.18	0.11	0.41	49.82	-0.21	2.75	0.73
2000	Sirjan	132	34715	0.29	0.24	0.15	0.48	73.72	-0.18	2.71	0.73
2000	Shahrebabak	133	13148	0.25	NA	0.11	0.49	60.24	-0.35	2.71	0.73
2000	Kerman	134	96837	0.33	0.28	0.21	0.47	86.56	-0.22	2.63	0.73
2000	Kahnuj	135	23413	0.11	0.09	0.05	0.21	19.79	-0.24	2.57	0.73
2000	Bardsir	136	12081	0.16	0.17	0.07	0.31	53.55	-0.19	2.65	0.73
2000	Ravar	137	6424	0.22	NA	0.08	0.46	60.08	-0.22	2.65	0.73
2000	Anbarabad	138	7907	0.15	NA	0.05	0.33	34.89	-0.22	2.68	0.73

2000	Manujan	139	6468	0.11	NA	0.04	0.25	19.63	-0.21	2.67	0.73
2000	Taybad	140	17934	0.13	0.12	0.06	0.25	43.21	-0.40	2.37	0.31
2000	Torbate Heydarieh	141	50013	0.3	0.44	0.17	0.48	43.16	-0.41	2.16	0.31
2000	Torbate Jam	142	30544	0.23	0.34	0.12	0.41	41.52	-0.46	2.29	0.31
2000	Darrehgaz	143	13502	0.18	0.15	0.08	0.33	53.56	-0.55	2.16	0.31
2000	Sabzevar	144	75562	0.21	0.21	0.12	0.33	46.12	-0.52	2.20	0.31
2000	Quchan	145	30576	0.15	0.06	0.07	0.26	47.37	-0.50	2.28	0.31
2000	Kashmar	146	32380	0.21	0.25	0.11	0.36	45.88	-0.46	2.17	0.31
2000	Gonabad	147	20398	0.22	NA	0.1	0.44	46.76	-0.42	2.26	0.31
2000	Mashhad	148	413571	0.29	0.28	0.22	0.37	89.55	-0.37	2.18	0.31
2000	Neyshabur	149	71792	0.28	0.36	0.17	0.42	48.53	-0.46	2.24	0.31
2000	Chenaran	150	16087	0.14	NA	0.06	0.28	36.16	-0.47	2.02	0.31
2000	Khaf	151	13601	0.14	NA	0.06	0.28	44.37	-0.43	2.24	0.31
2000	Sarakhs	152	10671	0.19	NA	0.08	0.39	39.31	-0.51	1.98	0.31
2000	Fariman	153	12449	0.19	NA	0.08	0.37	52.89	-0.46	2.11	0.31
2000	Bardeskan	154	11549	0.11	NA	0.04	0.23	32.76	-0.37	2.19	0.31
2000	Rashtkhar	155	8080	0.12	NA	0.04	0.27	17.70	-0.42	2.17	0.31
2000	Kalat	156	6354	0.2	NA	0.07	0.43	23.75	-0.46	2.15	0.31
2000	Ardestan	157	9584	0.19	NA	0.08	0.39	53.08	0.83	2.92	0.12
2000	Isfahan	158	323674	1.14	1.18	0.98	1.31	91.08	0.84	3.09	0.12
2000	Khomeynishahr	159	40990	0.33	0.41	0.19	0.53	93.10	0.88	3.13	0.12
2000	Khansar	160	7467	0.2	0.25	0.09	0.38	60.53	0.79	2.89	0.12
2000	Semirom	161	11135	0.2	0.18	0.09	0.38	51.63	0.83	2.98	0.12
2000	Faridan	162	15656	0.34	0.62	0.17	0.61	38.37	0.84	2.84	0.12
2000	Fereydunshahr	163	6867	0.16	0.29	0.07	0.33	45.03	0.90	2.92	0.12
2000	Falavarjan	164	33937	0.25	0.25	0.13	0.41	60.95	0.85	3.09	0.12
2000	Shahreza	165	31920	0.35	0.5	0.19	0.57	76.93	0.84	2.89	0.12
2000	Kashan	166	51282	1.02	1.31	0.71	1.4	83.76	0.76	3.11	0.12
2000	Golpayegan	167	17014	0.22	0.23	0.11	0.4	71.74	0.85	2.90	0.12
2000	Lanjan	168	35386	0.36	0.42	0.2	0.58	80.59	0.84	2.82	0.12
2000	Nayin	169	11285	0.23	0.34	0.11	0.45	63.99	0.84	3.15	0.12
2000	Najafabad	170	43888	0.59	0.76	0.37	0.88	91.35	0.89	3.02	0.12
2000	Natanz	171	8167	0.2	0.24	0.09	0.39	56.55	0.84	3.08	0.12
2000	Shahinshahr va Meyme	172	43416	0.45	0.52	0.27	0.7	88.16	0.84	2.97	0.12
2000	Mobarakeh	173	20679	0.23	0.2	0.11	0.41	80.57	0.82	3.01	0.12
2000	Aran va Bidgol	174	14747	0.27	0.28	0.13	0.49	85.32	0.79	2.94	0.12
2000	Tiran va Karvan	175	11486	0.15	NA	0.06	0.3	36.21	0.82	2.92	0.12
2000	Chadegan	176	6120	0.15	NA	0.06	0.32	38.50	0.85	3.10	0.12
2000	Iranshahr	177	25383	0.17	NA	0.07	0.33	42.03	-2.33	1.50	0.71
2000	Chah Bahar	178	21709	0.12	NA	0.05	0.24	30.32	-2.37	1.52	0.71
2000	Khash	179	15266	0.11	NA	0.04	0.24	27.95	-2.32	1.71	0.71
2000	Zabol	180	48800	0.11	0.04	0.05	0.19	35.19	-2.36	1.65	0.71
2000	Zahedan	181	62586	0.18	0.07	0.1	0.3	88.08	-2.39	1.56	0.71
2000	Saravan	182	27176	0.08	NA	0.03	0.16	35.15	-2.33	1.51	0.71
2000	Nikshahr	183	20614	0.09	NA	0.03	0.18	21.11	-2.40	1.66	0.71
2000	Sarbaz	184	12908	0.1	NA	0.03	0.26	11.78	-2.41	1.63	0.71
2000	Baneh	185	16292	0.14	NA	0.06	0.28	57.98	-0.89	1.32	0.22

2000	Bijar	186	19047	0.12	0.1	0.05	0.22	49.17	-0.87	1.38	0.22
2000	Saqqez	187	32735	0.17	0.25	0.08	0.3	62.05	-0.82	1.55	0.22
2000	Sanandaj	188	63461	0.34	0.39	0.21	0.52	79.86	-0.91	1.56	0.22
2000	Qorveh	189	31556	0.12	0.06	0.06	0.22	48.60	-0.88	1.46	0.22
2000	Marivan	190	18425	0.23	0.46	0.11	0.42	56.45	-0.86	1.62	0.22
2000	Divandarreh	191	12326	0.12	0.16	0.05	0.23	23.64	-0.85	1.50	0.22
2000	Kamyaran	192	15482	0.12	NA	0.05	0.24	40.52	-0.90	1.60	0.22
2000	Sarvabad	193	9078	0.09	NA	0.03	0.19	4.20	-0.83	1.51	0.22
2000	Tuyserkan	194	21749	0.17	0.27	0.08	0.31	41.91	-0.17	2.16	0.06
2000	Malayer	195	50611	0.17	0.16	0.09	0.29	54.31	-0.21	2.15	0.06
2000	Nahavand	196	31241	0.14	NA	0.06	0.27	47.50	-0.29	2.04	0.06
2000	Hamadan	197	104083	0.22	0.2	0.13	0.33	80.18	-0.23	2.02	0.06
2000	Kabudarahang	198	22216	0.1	0.09	0.05	0.19	15.64	-0.20	2.15	0.06
2000	Asadabad	199	17335	0.15	NA	0.07	0.3	45.17	-0.26	2.07	0.06
2000	Bahar	200	20500	0.16	0.19	0.08	0.3	41.09	-0.23	2.16	0.06
2000	Razan	201	19930	0.12	NA	0.05	0.25	16.69	-0.30	2.05	0.06
2000	Borujen	202	18385	0.17	0.22	0.08	0.33	79.94	-0.61	1.95	0.15
2000	Shahrekord	203	60833	0.21	0.17	0.12	0.34	70.64	-0.51	1.84	0.15
2000	Farsan	204	11835	0.19	0.53	0.09	0.36	61.43	-0.52	1.83	0.15
2000	Lordakan	205	19742	0.14	0.32	0.06	0.26	17.60	-0.52	1.70	0.15
2000	Ardal	206	6864	0.11	NA	0.04	0.25	14.52	-0.58	1.80	0.15
2000	Kuhrang	207	3964	0.1	NA	0.04	0.21	5.93	-0.58	1.94	0.15
2000	Aligudarz	208	19928	0.14	NA	0.06	0.29	55.41	-0.66	2.07	0.14
2000	Borujerd	209	56070	0.3	0.29	0.17	0.46	74.84	-0.67	2.14	0.14
2000	Khorramabad	210	72286	0.5	0.61	0.32	0.72	65.88	-0.68	2.09	0.14
2000	Dalfan	211	16559	0.12	NA	0.05	0.24	39.84	-0.63	2.03	0.14
2000	Dorud	212	22589	0.2	0.28	0.1	0.35	64.86	-0.61	1.92	0.14
2000	Kuhdasht	213	25354	0.13	0.25	0.06	0.24	48.31	-0.61	2.12	0.14
2000	Azna	214	11939	0.15	NA	0.06	0.3	50.48	-0.58	2.13	0.14
2000	Poldokhtar	215	9988	0.17	0.21	0.07	0.33	35.07	-0.67	2.00	0.14
2000	Selseleh	216	9850	0.16	NA	0.05	0.38	37.66	-0.63	1.88	0.14
2000	Ilam	217	26141	0.29	NA	0.13	0.56	80.16	-0.11	1.83	0.23
2000	Darrehshahr	218	7862	0.11	NA	0.04	0.23	35.03	-0.25	1.61	0.23
2000	Dehloran	219	7649	0.17	NA	0.07	0.35	62.21	-0.09	1.63	0.23
2000	Shirvan va Chardavol	220	10591	0.11	NA	0.04	0.23	17.46	-0.10	1.74	0.23
2000	Mehran	221	6825	0.22	NA	0.09	0.45	56.97	-0.09	1.63	0.23
2000	Abdanan	222	6419	0.14	NA	0.06	0.29	57.00	-0.21	1.81	0.23
2000	Eyvan	223	7272	0.26	NA	0.11	0.53	58.90	-0.14	1.68	0.23
2000	Boyerahmad	224	22616	0.19	NA	0.08	0.37	38.09	-1.01	1.77	0.31
2000	Kohgiluyeh	225	27933	0.1	NA	0.04	0.21	32.75	-0.95	1.77	0.31
2000	Gachsaran	226	20023	0.21	NA	0.09	0.4	59.73	-1.02	1.82	0.31
2000	Dena	227	7653	0.13	NA	0.05	0.26	19.29	-1.07	1.91	0.31
2000	Bushehr	228	30444	0.65	NA	0.29	1.26	85.33	0.58	2.35	0.70
2000	Tangestan	229	9890	0.2	NA	0.08	0.41	22.43	0.64	2.41	0.70
2000	Dashtestan	230	34609	0.22	NA	0.1	0.43	60.32	0.56	2.42	0.70
2000	Dashti	231	11356	0.2	NA	0.08	0.39	50.24	0.56	2.31	0.70
2000	Dayyer	232	6398	0.21	NA	0.09	0.43	67.84	0.62	2.24	0.70

2000	Kangan	233	7542	0.23	NA	0.1	0.48	67.43	0.54	2.29	0.70
2000	Genaveh	234	12489	0.34	NA	0.15	0.67	72.84	0.60	2.11	0.70
2000	Deylam	235	4341	0.3	NA	0.11	0.65	71.34	0.61	2.28	0.70
2000	Jam	236	3966	0.14	NA	0.05	0.31	32.28	0.56	2.14	0.70
2000	Abhar	237	26505	0.14	0.15	0.07	0.25	58.98	0.04	1.70	0.13
2000	Khodabandeh	238	24405	0.1	0.17	0.05	0.2	23.07	0.00	1.68	0.13
2000	Zanjan	239	70415	0.41	0.55	0.26	0.6	74.53	0.09	1.73	0.13
2000	Ijrud	240	6530	0.1	NA	0.04	0.22	6.23	0.07	1.81	0.13
2000	Khorramdarreh	241	9415	0.2	0.22	0.09	0.39	77.44	0.04	1.76	0.13
2000	Tarom	242	6979	0.11	NA	0.04	0.26	11.87	-0.01	1.97	0.13
2000	Mahneshan	243	6898	0.11	NA	0.04	0.24	11.10	-0.09	1.74	0.13
2000	Damghan	244	15350	0.24	0.13	0.12	0.44	62.59	1.80	3.73	0.21
2000	Semnan	245	29354	0.2	0.07	0.1	0.35	87.16	1.73	3.79	0.21
2000	Shahrud	246	39749	0.41	0.51	0.24	0.65	59.67	1.73	3.56	0.21
2000	Garmsar	247	14051	0.28	0.43	0.13	0.49	60.96	1.75	3.89	0.21
2000	Ardakan	248	10674	0.27	NA	0.12	0.52	79.51	1.01	2.92	0.30
2000	Bafq	249	7581	0.3	0.53	0.14	0.56	64.26	0.93	2.81	0.30
2000	Taft	250	11098	0.2	NA	0.09	0.41	28.07	0.97	2.99	0.30
2000	Mehriz	251	8092	0.34	0.48	0.16	0.62	50.92	1.05	2.75	0.30
2000	Yazd	252	71919	0.54	0.49	0.35	0.76	95.74	0.95	2.83	0.30
2000	Meybod	253	10537	0.3	0.19	0.14	0.55	90.55	0.98	2.84	0.30
2000	Abarkuh	254	7329	0.28	NA	0.12	0.56	61.83	1.03	2.77	0.30
2000	Sadugh	255	4895	0.28	NA	0.1	0.63	50.32	0.96	2.77	0.30
2000	Khatam	256	4770	0.23	NA	0.09	0.48	54.15	1.00	2.90	0.30
2000	Tabas	257	9969	0.23	NA	0.09	0.45	50.63	1.01	2.74	0.30
2000	Abumusa	258	97	0.24	NA	0.09	0.54	60.00	-0.30	1.68	0.44
2000	Bandarabbas	259	66000	0.25	0.2	0.14	0.4	66.41	-0.28	1.93	0.44
2000	Bandar-e Lengeh	260	17394	0.16	0.12	0.08	0.29	45.70	-0.32	1.75	0.44
2000	Qeshm	261	11126	0.19	NA	0.08	0.38	37.74	-0.21	1.83	0.44
2000	Minab	262	31244	0.13	0.07	0.06	0.23	23.09	-0.32	1.70	0.44
2000	Bandar-e-Jask	263	8558	0.14	0.48	0.07	0.26	15.99	-0.26	1.79	0.44
2000	Rudan	264	12958	0.11	NA	0.04	0.22	30.86	-0.21	1.78	0.44
2000	Hajiabad	265	9667	0.12	NA	0.05	0.25	29.05	-0.27	1.82	0.44
2000	Bastak	266	8659	0.17	NA	0.07	0.34	24.31	-0.26	1.82	0.44
2000	Tehran	267	1499799	0.06	0.04	0.04	0.08	99.63	1.34	3.15	0.27
2000	Damavand	268	14011	0.3	NA	0.13	0.59	66.60	1.41	3.36	0.27
2000	Rey	269	31993	0.29	0.07	0.15	0.49	31.00	1.40	3.18	0.27
2000	Shemiranat	270	6522	0.52	NA	0.23	1.03	53.76	1.34	3.33	0.27
2000	Varamin	271	72084	0.15	0.03	0.09	0.25	76.63	1.41	3.29	0.27
2000	Shahriyar	272	103684	0.17	NA	0.08	0.33	79.04	1.32	3.20	0.27
2000	Eslamshahr	273	63592	0.18	NA	0.08	0.35	87.32	1.33	3.42	0.27
2000	Robatkarim	274	56462	0.11	NA	0.05	0.21	79.25	1.33	3.25	0.27
2000	Pakdasht	275	26112	0.12	0.09	0.06	0.23	67.19	1.40	3.35	0.27
2000	Firuzkuh	276	6842	0.15	NA	0.06	0.32	37.52	1.36	3.17	0.27
2000	Ardebil	277	79882	0.13	0.05	0.07	0.21	80.22	-0.87	1.45	0.12
2000	Bilehsowar	278	8665	0.12	NA	0.05	0.25	33.56	-0.79	1.76	0.12
2000	Khalkhal	279	17544	0.19	NA	0.07	0.43	40.69	-0.76	1.67	0.12

2000	Meshginshahr	280	26017	0.19	0.16	0.09	0.33	36.66	-0.80	1.48	0.12
2000	Germi	281	15262	0.13	0.26	0.06	0.26	27.01	-0.77	1.45	0.12
2000	Parsabad	282	20588	0.11	0.11	0.05	0.21	48.94	-0.78	1.61	0.12
2000	Kowsar	283	5299	0.12	NA	0.04	0.29	22.14	-0.83	1.64	0.12
2000	Namin	284	10368	0.12	NA	0.05	0.25	30.97	-0.80	1.36	0.12
2000	Neer	285	4796	0.11	NA	0.04	0.26	21.11	-0.82	1.51	0.12
2000	Qom	286	147450	0.44	0.5	0.31	0.59	91.30	2.22	2.71	0.16
2000	Bueenzahra	287	24611	0.1	0.16	0.05	0.19	31.18	0.23	2.29	0.24
2000	Takestan	288	25868	0.19	0.23	0.09	0.34	52.65	0.13	2.18	0.24
2000	Qazvin	289	106935	0.69	0.83	0.49	0.91	75.90	0.16	2.30	0.24
2000	Abyek	290	12924	0.16	NA	0.07	0.33	42.95	0.19	2.30	0.24
2000	Bandare Gaz	291	8985	0.3	0.67	0.14	0.55	51.68	0.17	2.29	0.31
2000	Torkman	292	19501	0.2	NA	0.09	0.4	59.08	0.19	2.29	0.31
2000	Aliabad	293	19393	0.2	0.11	0.1	0.36	44.97	0.16	2.29	0.31
2000	Kordkuy	294	12718	0.22	0.16	0.11	0.4	46.57	0.12	2.24	0.31
2000	Gorgan	295	61790	0.34	0.3	0.2	0.52	65.82	0.16	2.42	0.31
2000	Gonbade Kavus	296	41860	0.22	0.2	0.12	0.36	49.32	0.19	2.15	0.31
2000	Minudasht	297	19415	0.14	0.11	0.06	0.26	30.60	0.05	2.32	0.31
2000	Aqqala	298	15513	0.11	NA	0.05	0.22	28.44	0.12	2.18	0.31
2000	Kalaleh	299	19156	0.11	0.11	0.05	0.22	22.40	0.11	2.30	0.31
2000	Azadshahr	300	13240	0.18	NA	0.08	0.35	51.96	0.07	2.15	0.31
2000	Ramyan	301	11973	0.11	0.17	0.05	0.22	38.41	0.04	2.47	0.31
2000	Esfarayen	302	20126	0.11	NA	0.05	0.22	34.05	-0.91	2.39	0.25
2000	Bojnurd	303	45201	0.18	NA	0.08	0.36	54.40	-0.87	2.21	0.25
2000	Jajarm	304	9183	0.11	NA	0.04	0.25	58.44	-0.87	2.36	0.25
2000	Shirvan	305	24710	0.14	NA	0.06	0.28	47.26	-0.81	2.37	0.25
2000	Faruj	306	9776	0.1	0.19	0.04	0.21	22.66	-0.97	2.39	0.25
2000	Maneh va Semelqan	307	12908	0.13	NA	0.05	0.27	21.85	-0.97	2.33	0.25
2000	Birjand	308	42747	0.29	NA	0.13	0.56	51.79	-2.48	2.26	0.24
2000	Sarbisheh	309	8251	0.15	NA	0.04	0.4	22.20	-2.53	2.05	0.24
2000	Qaenat	310	22654	0.13	NA	0.06	0.26	29.39	-0.45	2.36	0.24
2000	Nehbandan	311	7462	0.11	NA	0.04	0.26	22.29	-2.43	1.80	0.24
2000	Sarayan	312	5881	0.19	NA	0.07	0.41	57.27	-2.46	1.96	0.24
2000	Ferdows	313	11827	0.18	NA	0.08	0.36	63.44	-0.37	2.19	0.24
2000	Karaj	314	239926	0.08	0.01	0.05	0.12	95.92	1.32	3.06	0.76
2000	Savojbolagh	315	27127	0.05	NA	0.02	0.11	33.51	1.31	3.31	0.76
2000	Nazarabad	316	16991	0.15	NA	0.06	0.32	75.53	1.32	3.19	0.76
2001	Arak	1	103021	0.24	0.2	0.15	0.36	76.30	0.37	2.76	0.18
2001	Ashtiyan	2	4195	0.25	NA	0.1	0.54	30.05	0.43	2.58	0.18
2001	Tafresh	3	13113	0.22	0.42	0.1	0.41	23.93	0.28	2.68	0.18
2001	Khomeyn	4	22508	0.16	0.09	0.07	0.28	47.36	0.43	2.76	0.18
2001	Delijan	5	8145	0.3	NA	0.13	0.59	62.51	0.44	2.73	0.18
2001	Saveh	6	34273	0.2	NA	0.09	0.38	62.88	0.40	2.80	0.18
2001	Shazand	7	23148	0.17	NA	0.07	0.33	26.79	0.54	2.70	0.18
2001	Mahalat	8	9512	0.26	NA	0.11	0.53	76.77	0.44	2.78	0.18
2001	Zarandiyeh	9	10539	0.16	NA	0.06	0.32	52.64	0.43	2.73	0.18
2001	Komijan	10	7141	0.17	NA	0.06	0.37	37.57	0.42	2.61	0.18

2001	Astara	11	12907	0.19	NA	0.08	0.39	58.03	-0.24	3.17	0.13
2001	Astanehye Ashrafiyeh	12	24614	0.21	0.08	0.1	0.36	40.81	-0.26	3.25	0.13
2001	Bandar Anzali	13	28612	0.28	0.07	0.15	0.47	81.36	-0.22	3.25	0.13
2001	Tavalesh	14	28947	0.15	0.07	0.07	0.27	29.20	-0.25	3.42	0.13
2001	Rasht	15	165455	0.25	0.21	0.17	0.36	65.20	-0.21	3.04	0.13
2001	Rudbar	16	20362	0.3	0.49	0.15	0.52	46.74	-0.26	3.22	0.13
2001	Rudsar	17	33296	0.24	0.06	0.13	0.41	44.13	-0.28	3.29	0.13
2001	Sumehsara	18	28106	0.21	0.14	0.11	0.37	26.84	-0.20	3.08	0.13
2001	Fuman	19	21514	0.19	0.09	0.09	0.34	26.21	-0.24	3.10	0.13
2001	Langrud	20	29414	0.25	0.13	0.13	0.42	57.40	-0.18	3.14	0.13
2001	Lahijan	21	35821	0.29	0.21	0.16	0.48	39.04	-0.27	3.30	0.13
2001	Shaft	22	14050	0.17	0.14	0.08	0.32	11.40	-0.37	3.06	0.13
2001	Amlash	23	10248	0.22	NA	0.09	0.44	29.87	-0.20	3.21	0.13
2001	Rezvanshahr	24	11806	0.19	NA	0.08	0.39	26.17	-0.28	3.13	0.13
2001	Siyahkal	25	10632	0.26	0.36	0.12	0.48	28.18	-0.24	3.33	0.13
2001	Masal	26	9171	0.2	NA	0.08	0.41	29.38	-0.22	3.35	0.13
2001	Amol	27	63351	0.33	NA	0.15	0.62	52.90	1.34	3.41	0.27
2001	Babol	28	88931	0.26	0.16	0.16	0.38	44.58	1.27	3.21	0.27
2001	Behshahr	29	35999	0.3	NA	0.14	0.59	59.37	1.29	3.33	0.27
2001	Tonekabon	30	38803	0.28	NA	0.12	0.53	45.60	1.29	3.35	0.27
2001	Ramsar	31	14581	0.31	0.14	0.15	0.56	69.91	1.34	3.33	0.27
2001	Sari	32	89324	0.17	0.02	0.1	0.27	47.98	1.25	3.21	0.27
2001	Savadkuh	33	14214	0.18	NA	0.07	0.37	44.85	1.20	3.33	0.27
2001	Qaemshahr	34	55933	0.3	NA	0.13	0.57	56.43	1.30	3.32	0.27
2001	Nur	35	19665	0.18	NA	0.08	0.35	37.03	1.25	3.31	0.27
2001	Noshahr	36	21350	0.13	NA	0.05	0.26	33.52	1.24	3.38	0.27
2001	Babolsar	37	32011	0.27	NA	0.12	0.53	49.40	1.32	3.37	0.27
2001	Mahmudabad	38	16463	0.23	NA	0.1	0.46	29.61	1.36	3.52	0.27
2001	Neka	39	18510	0.2	NA	0.08	0.4	33.25	1.30	3.08	0.27
2001	Chalus	40	22606	0.18	NA	0.08	0.36	51.57	1.31	3.39	0.27
2001	Juybar	41	13306	0.25	NA	0.1	0.5	38.39	1.31	3.41	0.27
2001	Ahar	42	25713	0.15	0.16	0.07	0.27	60.14	-0.38	1.94	0.19
2001	Tabriz	43	279683	0.49	0.5	0.39	0.61	93.36	-0.40	1.95	0.19
2001	Sarab	44	24112	0.19	0.16	0.09	0.34	39.37	-0.35	2.09	0.19
2001	Maragheh	45	40940	0.15	0.05	0.07	0.25	67.65	-0.44	1.99	0.19
2001	Marand	46	42912	0.71	1.08	0.45	1.04	55.82	-0.34	2.08	0.19
2001	Miyaneh	47	35984	0.18	0.22	0.09	0.32	45.64	-0.45	2.11	0.19
2001	Hashtrud	48	11660	0.16	0.17	0.07	0.31	25.05	-0.39	2.11	0.19
2001	Bonab	49	21155	0.25	0.39	0.13	0.45	59.84	-0.39	2.15	0.19
2001	Bostanabad	50	16132	0.16	NA	0.07	0.32	16.90	-0.34	2.10	0.19
2001	Shabestar	51	25822	0.2	0.3	0.1	0.35	46.11	-0.39	2.13	0.19
2001	Kalibar	52	14752	0.11	NA	0.04	0.24	12.90	-0.39	2.26	0.19
2001	Haris	53	11865	0.21	0.17	0.09	0.39	44.09	-0.36	2.11	0.19
2001	Jolfa	54	10686	0.17	NA	0.07	0.36	58.56	-0.40	2.13	0.19
2001	Malekan	55	16383	0.14	NA	0.06	0.28	30.91	-0.33	2.00	0.19
2001	Azarshahr	56	18878	0.16	0.11	0.07	0.29	59.13	-0.41	2.16	0.19
2001	Osku	57	14634	0.16	0.41	0.07	0.31	48.02	-0.34	2.00	0.19

2001	Charoymaq	58	5395	0.11	NA	0.04	0.23	11.01	-0.38	2.12	0.19
2001	Varzaqan	59	8366	0.13	0.23	0.05	0.26	9.70	-0.35	2.10	0.19
2001	Ajabshir	60	11674	0.17	NA	0.07	0.35	40.77	-0.39	2.05	0.19
2001	Orumiyeh	61	141088	0.31	0.29	0.21	0.43	67.79	-0.18	2.04	0.25
2001	Piranshahr	62	13615	0.16	0.16	0.07	0.3	45.18	-0.18	1.96	0.25
2001	Khoy	63	59440	0.14	0.1	0.07	0.23	64.20	-0.13	1.96	0.25
2001	Sardasht	64	14587	0.11	NA	0.04	0.22	43.62	-0.15	1.94	0.25
2001	Salmas	65	27151	0.31	0.45	0.16	0.52	53.27	-0.16	1.90	0.25
2001	Maku	66	26102	0.14	0.32	0.07	0.27	46.82	-0.16	1.97	0.25
2001	Mahabad	67	30838	0.16	NA	0.07	0.31	67.66	-0.18	2.01	0.25
2001	Miyandoab	68	39472	0.14	0.05	0.07	0.24	49.46	-0.11	1.86	0.25
2001	Naqadeh	69	19697	0.22	0.21	0.11	0.4	65.80	-0.18	1.96	0.25
2001	Bukan	70	30388	0.14	NA	0.06	0.29	70.98	-0.11	1.92	0.25
2001	Shahindezh	71	15441	0.16	NA	0.06	0.32	47.07	-0.18	1.89	0.25
2001	Takab	72	14065	0.13	NA	0.05	0.26	53.41	-0.15	2.00	0.25
2001	Oshnaviyeh	73	8736	0.3	NA	0.12	0.6	53.21	-0.18	2.03	0.25
2001	Chaldoran	74	6486	0.15	0.31	0.05	0.33	33.50	-0.16	1.95	0.25
2001	Eslamabade Gharb	75	33486	0.12	0.06	0.06	0.21	49.86	-0.43	2.10	0.14
2001	Kermanshah	76	160590	0.5	0.52	0.36	0.65	83.84	-0.45	2.18	0.14
2001	Paveh	77	9271	0.19	0.22	0.08	0.37	50.04	-0.49	2.02	0.14
2001	Sarpole Zahab	78	12380	0.19	NA	0.08	0.38	39.00	-0.55	2.17	0.14
2001	Sonqor	79	18553	0.19	NA	0.08	0.38	44.94	-0.45	2.14	0.14
2001	Qasreshirin	80	3633	0.29	0.56	0.12	0.59	67.58	-0.44	2.17	0.14
2001	Kangavar	81	14591	0.21	0.14	0.1	0.38	59.81	-0.46	2.12	0.14
2001	Gilanegharb	82	10874	0.14	NA	0.06	0.29	31.58	-0.52	2.11	0.14
2001	Javanrud	83	15331	0.14	0.14	0.06	0.28	50.60	-0.51	2.23	0.14
2001	Sahneh	84	14309	0.29	0.83	0.14	0.53	39.40	-0.55	2.11	0.14
2001	Harsin	85	14504	0.2	NA	0.08	0.4	64.63	-0.44	2.11	0.14
2001	Salas-e-Babajani	86	4388	0.12	NA	0.04	0.29	17.96	-0.50	2.30	0.14
2001	Abadan	87	42942	0.55	0.49	0.33	0.84	84.44	0.82	2.82	0.29
2001	Andimeshk	88	23207	0.24	0.09	0.12	0.41	75.40	0.95	2.68	0.29
2001	Ahvaz	89	188454	0.72	0.74	0.57	0.9	82.16	0.88	2.56	0.29
2001	Izeh	90	25344	0.17	0.17	0.08	0.32	48.84	1.00	2.82	0.29
2001	Bandar-e-Mahshahr	91	32700	0.45	0.46	0.25	0.73	94.15	0.89	2.68	0.29
2001	Behbahan	92	28929	0.36	0.35	0.19	0.59	71.74	0.87	2.46	0.29
2001	Khorramshahr	93	21843	0.35	0.19	0.18	0.6	83.89	0.88	2.57	0.29
2001	Dezful	94	56037	0.38	0.42	0.23	0.59	73.05	0.95	2.74	0.29
2001	Dashte Azadegan	95	16622	0.27	0.13	0.13	0.48	55.45	0.87	2.73	0.29
2001	Ramhormoz	96	24621	0.37	0.51	0.19	0.62	51.48	0.94	2.83	0.29
2001	Shadegan	97	16946	0.38	0.62	0.19	0.66	38.18	0.92	2.65	0.29
2001	Shushtar	98	33067	0.35	0.38	0.19	0.57	59.33	0.93	2.49	0.29
2001	Masjedsoleyman	99	27795	0.28	0.29	0.14	0.48	66.10	0.90	2.68	0.29
2001	Shush	100	24752	0.24	0.09	0.12	0.42	45.63	0.89	2.64	0.29
2001	Baghmalek	101	12807	0.18	0.16	0.08	0.34	33.22	0.96	2.69	0.29
2001	Omidiyeh	102	12527	0.3	0.17	0.14	0.54	69.28	0.95	2.66	0.29
2001	Lali	103	4676	0.25	NA	0.09	0.54	47.56	0.90	2.56	0.29
2001	Hendijan	104	5713	0.38	NA	0.15	0.79	68.21	0.94	2.59	0.29

2001	Abadeh	105	17909	0.43	0.44	0.22	0.73	88.07	0.41	2.73	0.24
2001	Estahban	106	11071	0.3	0.36	0.14	0.56	71.55	0.45	2.78	0.24
2001	Eqlid	107	14998	0.34	0.55	0.17	0.62	64.10	0.44	2.79	0.24
2001	Jahrom	108	34004	0.3	0.3	0.16	0.5	63.66	0.49	2.69	0.24
2001	Darab	109	25517	0.31	0.41	0.16	0.54	42.27	0.45	2.65	0.24
2001	Sepidan	110	13386	0.17	0.15	0.08	0.32	18.28	0.46	2.71	0.24
2001	Shiraz	111	277768	0.95	0.99	0.8	1.12	87.48	0.53	2.62	0.24
2001	Fasa	112	31753	0.47	0.64	0.27	0.75	56.86	0.46	2.75	0.24
2001	Firuzabad	113	19544	0.27	0.31	0.13	0.48	52.81	0.54	2.51	0.24
2001	Kazerun	114	43895	0.24	0.23	0.13	0.39	51.46	0.50	2.78	0.24
2001	Lar (Larestan)	115	40695	0.45	0.56	0.26	0.7	57.26	0.43	2.76	0.24
2001	Marvdasht	116	48089	0.22	0.22	0.12	0.36	44.40	0.45	2.67	0.24
2001	Mamasany	117	28401	0.15	0.14	0.07	0.26	29.30	0.44	2.55	0.24
2001	Neyriz	118	16387	0.25	NA	0.11	0.48	58.38	0.53	2.68	0.24
2001	Lamard	119	10957	0.27	0.37	0.13	0.5	42.11	0.47	2.67	0.24
2001	Bovanat	120	7901	0.19	0.25	0.08	0.38	29.26	0.48	2.70	0.24
2001	Arsanjan	121	5974	0.36	0.35	0.15	0.7	39.35	0.48	2.64	0.24
2001	Khorrambid	122	6971	0.24	NA	0.1	0.5	76.39	0.45	2.70	0.24
2001	Zarrindasht	123	8259	0.22	NA	0.09	0.48	57.57	0.42	2.69	0.24
2001	Qirokarzin	124	9003	0.19	NA	0.08	0.4	56.54	0.51	2.72	0.24
2001	Mohr	125	7063	0.2	NA	0.08	0.42	35.61	0.40	2.64	0.24
2001	Farashband	126	6269	0.25	NA	0.1	0.51	58.70	0.58	2.57	0.24
2001	Baft	127	22763	0.19	0.18	0.09	0.34	34.37	-0.18	2.70	0.74
2001	Bam	128	33170	0.3	0.56	0.16	0.5	42.57	-0.04	2.72	0.74
2001	Jiroft	129	28536	0.21	0.22	0.11	0.37	38.59	-0.08	2.82	0.74
2001	Rafsanjan	130	43373	0.33	0.29	0.19	0.53	54.62	-0.06	2.95	0.74
2001	Zarand	131	23449	0.25	0.18	0.12	0.44	50.43	-0.12	2.75	0.74
2001	Sirjan	132	36111	0.4	0.46	0.22	0.63	74.00	-0.12	2.82	0.74
2001	Shahrebabak	133	13943	0.28	0.28	0.13	0.51	60.85	-0.07	2.97	0.74
2001	Kerman	134	100887	0.75	0.82	0.55	0.98	86.79	-0.05	2.91	0.74
2001	Kahnuj	135	25260	0.14	0.17	0.07	0.25	20.13	-0.04	2.88	0.74
2001	Bardsir	136	12610	0.18	0.16	0.08	0.35	53.81	-0.03	2.95	0.74
2001	Ravar	137	6633	0.24	NA	0.09	0.52	59.76	-0.07	2.85	0.74
2001	Anbarabad	138	8439	0.16	NA	0.06	0.38	34.50	-0.07	2.87	0.74
2001	Manujan	139	6799	0.13	NA	0.05	0.29	21.21	-0.01	2.90	0.74
2001	Taybad	140	18558	0.14	0.11	0.07	0.27	43.66	-0.23	2.24	0.36
2001	Torbate Heydarieh	141	51463	0.17	0.08	0.09	0.29	43.76	-0.31	2.27	0.36
2001	Torbate Jam	142	31660	0.2	0.13	0.1	0.34	41.97	-0.21	2.43	0.36
2001	Darrehgaz	143	13805	0.21	NA	0.09	0.43	53.86	-0.23	2.40	0.36
2001	Sabzevar	144	77518	0.19	0.13	0.11	0.3	46.77	-0.28	2.29	0.36
2001	Quchan	145	31336	0.19	NA	0.08	0.37	47.91	-0.26	2.26	0.36
2001	Kashmar	146	33312	0.22	NA	0.1	0.43	46.47	-0.28	2.17	0.36
2001	Gonabad	147	20864	0.22	0.09	0.11	0.39	47.21	-0.30	2.43	0.36
2001	Mashhad	148	432633	0.16	0.12	0.11	0.21	89.72	-0.17	2.30	0.36
2001	Neyshabur	149	73963	0.18	0.14	0.1	0.29	49.21	-0.21	2.22	0.36
2001	Chenaran	150	16674	0.17	NA	0.07	0.33	36.87	-0.22	2.35	0.36
2001	Khaf	151	14111	0.16	NA	0.07	0.31	44.51	-0.25	2.29	0.36

2001	Sarakhs	152	11082	0.23	NA	0.1	0.46	39.54	-0.20	2.31	0.36
2001	Fariman	153	12903	0.21	NA	0.09	0.43	53.06	-0.29	2.44	0.36
2001	Bardeskan	154	11876	0.12	NA	0.05	0.26	33.44	-0.29	2.23	0.36
2001	Rashtkhar	155	8321	0.13	NA	0.05	0.3	17.90	-0.25	2.20	0.36
2001	Kalat	156	6510	0.22	NA	0.08	0.49	23.81	-0.23	2.27	0.36
2001	Ardestan	157	9715	0.22	NA	0.09	0.45	53.49	1.09	3.19	0.16
2001	Isfahan	158	337547	0.67	NA	0.31	1.28	91.18	0.90	3.02	0.16
2001	Khomeynishahr	159	42716	0.32	NA	0.14	0.61	93.95	1.01	3.11	0.16
2001	Khansar	160	7533	0.22	NA	0.1	0.44	60.84	0.98	3.16	0.16
2001	Semirom	161	11581	0.23	NA	0.09	0.45	51.47	1.01	3.12	0.16
2001	Faridan	162	15907	0.31	NA	0.14	0.61	38.93	0.98	3.07	0.16
2001	Fereydunshahr	163	6995	0.18	NA	0.07	0.37	45.49	1.02	3.01	0.16
2001	Falavarjan	164	35246	0.28	NA	0.13	0.54	61.07	1.07	3.11	0.16
2001	Shahreza	165	32908	0.31	NA	0.14	0.6	77.41	1.01	3.19	0.16
2001	Kashan	166	53134	0.45	0.42	0.27	0.67	84.11	0.98	3.02	0.16
2001	Golpayegan	167	17390	0.26	NA	0.11	0.5	72.09	0.97	3.19	0.16
2001	Lanjan	168	36708	0.38	NA	0.17	0.74	81.03	1.07	3.09	0.16
2001	Nayin	169	11500	0.25	NA	0.11	0.49	64.58	1.06	3.20	0.16
2001	Najafabad	170	45623	0.45	NA	0.21	0.86	91.43	1.05	3.09	0.16
2001	Natanz	171	8450	0.23	NA	0.1	0.46	56.34	1.03	3.24	0.16
2001	Shahinshahr va Meyme	172	45424	0.45	NA	0.21	0.86	88.49	0.96	3.11	0.16
2001	Mobarakeh	173	21496	0.27	NA	0.12	0.51	80.80	0.96	3.09	0.16
2001	Aran va Bidgol	174	15299	0.31	NA	0.14	0.59	85.36	1.06	3.21	0.16
2001	Tiran va Karvan	175	11798	0.17	NA	0.07	0.34	36.51	1.04	3.16	0.16
2001	Chadegan	176	6212	0.17	NA	0.06	0.35	38.69	1.09	3.06	0.16
2001	Iranshahr	177	26657	0.21	0.32	0.1	0.38	42.17	-2.10	1.67	0.67
2001	Chah Bahar	178	23182	0.13	NA	0.05	0.27	30.71	-2.13	1.76	0.67
2001	Khash	179	15900	0.12	NA	0.05	0.25	28.58	-2.04	1.57	0.67
2001	Zabol	180	50140	0.14	NA	0.06	0.28	35.70	-2.10	1.80	0.67
2001	Zahedan	181	65614	0.19	0.07	0.1	0.3	87.97	-2.12	1.62	0.67
2001	Saravan	182	28389	0.09	NA	0.04	0.18	35.33	-2.03	1.71	0.67
2001	Nikshahr	183	21443	0.09	NA	0.04	0.19	21.20	-2.07	1.54	0.67
2001	Sarbaz	184	13648	0.11	NA	0.03	0.28	11.64	-2.16	1.66	0.67
2001	Baneh	185	16884	0.16	NA	0.07	0.33	58.65	-0.55	1.70	0.29
2001	Bijar	186	19343	0.14	0.2	0.07	0.27	49.90	-0.55	1.73	0.29
2001	Saqqez	187	33805	0.15	0.12	0.08	0.27	62.53	-0.60	1.64	0.29
2001	Sanandaj	188	66058	0.43	0.5	0.27	0.63	80.21	-0.69	1.63	0.29
2001	Qorveh	189	32603	0.15	0.13	0.07	0.27	49.19	-0.57	1.64	0.29
2001	Marivan	190	19434	0.22	0.33	0.11	0.4	57.51	-0.54	1.41	0.29
2001	Divandarreh	191	12685	0.13	NA	0.06	0.27	24.17	-0.71	1.67	0.29
2001	Kamyaran	192	16014	0.13	NA	0.05	0.27	40.98	-0.64	1.60	0.29
2001	Sarvabad	193	9249	0.11	0.22	0.04	0.22	4.35	-0.57	1.61	0.29
2001	Tuyserkan	194	22115	0.16	0.09	0.07	0.29	42.42	-0.13	2.20	0.09
2001	Malayer	195	51861	0.21	0.23	0.12	0.35	54.90	-0.02	2.11	0.09
2001	Nahavand	196	32019	0.14	0.06	0.07	0.24	47.87	-0.01	2.31	0.09
2001	Hamadan	197	108019	0.26	0.25	0.16	0.38	80.49	-0.10	2.00	0.09
2001	Kabudarahang	198	22586	0.11	0.09	0.05	0.2	15.84	-0.03	2.09	0.09

2001	Asadabad	199	17770	0.16	0.11	0.07	0.3	45.66	-0.03	2.16	0.09
2001	Bahar	200	20986	0.18	0.19	0.09	0.32	41.35	-0.06	2.06	0.09
2001	Razan	201	20285	0.16	0.29	0.07	0.3	17.09	-0.04	2.22	0.09
2001	Borujen	202	19064	0.19	0.22	0.09	0.35	80.16	-0.33	1.96	0.17
2001	Shahrekord	203	63051	0.19	0.1	0.11	0.31	71.05	-0.29	1.99	0.17
2001	Farsan	204	12287	0.17	0.17	0.08	0.34	61.82	-0.37	2.13	0.17
2001	Lordakan	205	20663	0.14	NA	0.06	0.27	17.97	-0.31	2.11	0.17
2001	Ardal	206	7127	0.12	NA	0.04	0.28	14.65	-0.34	1.99	0.17
2001	Kuhrang	207	4149	0.11	NA	0.04	0.23	6.03	-0.41	2.00	0.17
2001	Aligudarz	208	20469	0.16	NA	0.07	0.33	56.02	-0.36	2.29	0.18
2001	Borujerd	209	57556	0.43	0.49	0.27	0.64	74.99	-0.38	2.30	0.18
2001	Khorramabad	210	75456	0.56	0.67	0.37	0.79	66.11	-0.37	2.32	0.18
2001	Dalfan	211	17202	0.13	NA	0.06	0.27	40.13	-0.33	2.16	0.18
2001	Dorud	212	23500	0.21	NA	0.09	0.4	65.08	-0.42	2.21	0.18
2001	Kuhdasht	213	26594	0.12	0.08	0.06	0.22	48.65	-0.41	2.29	0.18
2001	Azna	214	12249	0.17	NA	0.07	0.33	50.98	-0.36	2.07	0.18
2001	Poldokhtar	215	10466	0.19	NA	0.08	0.39	35.47	-0.35	2.22	0.18
2001	Selseleh	216	10238	0.19	NA	0.06	0.45	38.14	-0.32	2.22	0.18
2001	Ilam	217	27430	0.25	0.08	0.12	0.44	80.46	0.09	1.84	0.27
2001	Darrehshahr	218	8204	0.12	NA	0.05	0.27	35.48	0.12	1.90	0.27
2001	Dehloran	219	8007	0.2	0.26	0.08	0.4	62.51	0.10	1.96	0.27
2001	Shirvan va Chardavol	220	10948	0.12	NA	0.05	0.26	18.19	0.06	1.83	0.27
2001	Mehran	221	7183	0.25	NA	0.1	0.52	57.04	0.11	1.89	0.27
2001	Abdanan	222	6672	0.16	NA	0.07	0.33	57.34	-0.01	1.99	0.27
2001	Eyvan	223	7570	0.3	NA	0.12	0.61	59.60	0.11	1.93	0.27
2001	Boyerahmad	224	24013	0.24	0.37	0.12	0.44	38.82	-0.67	1.93	0.31
2001	Kohgiluyeh	225	29137	0.1	0.07	0.05	0.19	33.80	-0.69	1.72	0.31
2001	Gachsaran	226	20729	0.27	0.41	0.13	0.47	60.19	-0.69	2.00	0.31
2001	Dena	227	7875	0.15	NA	0.06	0.3	19.32	-0.73	2.03	0.31
2001	Bushehr	228	31424	0.63	0.56	0.37	0.97	85.37	0.69	2.51	0.73
2001	Tangestan	229	10201	0.21	0.2	0.1	0.41	22.53	0.78	2.29	0.73
2001	Dashtestan	230	35740	0.26	0.28	0.14	0.43	60.57	0.72	2.46	0.73
2001	Dashti	231	11712	0.26	0.52	0.12	0.49	51.14	0.69	2.43	0.73
2001	Dayyer	232	6668	0.25	0.32	0.11	0.48	67.92	0.69	2.57	0.73
2001	Kangan	233	7864	0.26	0.27	0.11	0.51	67.72	0.73	2.48	0.73
2001	Genaveh	234	12996	0.41	0.47	0.2	0.73	73.00	0.75	2.41	0.73
2001	Deylam	235	4511	0.34	NA	0.13	0.73	71.61	0.77	2.52	0.73
2001	Jam	236	4214	0.16	NA	0.06	0.35	32.94	0.78	2.34	0.73
2001	Abhar	237	27425	0.16	NA	0.07	0.31	59.66	0.06	1.83	0.18
2001	Khodabandeh	238	25107	0.11	NA	0.05	0.22	23.55	0.19	1.78	0.18
2001	Zanjan	239	73142	0.28	0.28	0.17	0.42	75.05	0.15	1.99	0.18
2001	Ijrud	240	6658	0.12	NA	0.05	0.26	6.30	0.10	2.07	0.18
2001	Khorramdarreh	241	9811	0.22	0.21	0.1	0.43	77.68	0.08	1.80	0.18
2001	Tarom	242	7213	0.12	NA	0.04	0.28	12.07	0.06	1.77	0.18
2001	Mahneshan	243	7009	0.12	NA	0.04	0.27	11.46	0.03	1.79	0.18
2001	Damghan	244	15719	0.3	0.25	0.15	0.53	63.43	1.84	4.09	0.25
2001	Semnan	245	30583	0.25	0.2	0.13	0.43	87.47	1.89	3.97	0.25

2001	Shahrud	246	40930	0.49	0.59	0.29	0.75	60.27	1.98	3.77	0.25
2001	Garmsar	247	14465	0.28	0.28	0.14	0.5	62.05	1.93	3.96	0.25
2001	Ardakan	248	11001	0.45	1.09	0.22	0.8	79.66	1.08	3.00	0.34
2001	Bafq	249	7869	0.3	0.26	0.14	0.55	64.48	1.03	2.89	0.34
2001	Taft	250	11121	0.26	0.49	0.12	0.49	28.70	1.18	2.96	0.34
2001	Mehriz	251	8219	0.46	0.94	0.22	0.83	51.53	1.14	2.95	0.34
2001	Yazd	252	75287	1.34	1.49	1.01	1.71	95.65	1.13	3.10	0.34
2001	Meybod	253	10914	0.36	0.37	0.17	0.66	90.44	1.12	3.00	0.34
2001	Abarkuh	254	7531	0.32	NA	0.14	0.64	62.01	1.10	3.00	0.34
2001	Sadugh	255	4995	0.32	NA	0.12	0.7	50.78	1.12	2.85	0.34
2001	Khatam	256	4953	0.25	NA	0.1	0.54	54.23	1.11	2.83	0.34
2001	Tabas	257	10290	0.26	NA	0.11	0.52	51.34	1.10	2.91	0.34
2001	Abumusa	258	110	0.27	NA	0.1	0.6	60.00	-0.10	1.90	0.41
2001	Bandarabbas	259	69194	0.43	0.51	0.27	0.65	66.69	-0.12	1.85	0.41
2001	Bandar-e Lengeh	260	18258	0.26	0.58	0.14	0.45	45.90	-0.12	1.93	0.41
2001	Qeshm	261	11665	0.25	0.55	0.11	0.46	37.97	-0.18	1.70	0.41
2001	Minab	262	32523	0.16	NA	0.07	0.32	23.23	-0.05	1.99	0.41
2001	Bandar-e-Jask	263	8869	0.13	NA	0.06	0.25	15.93	-0.13	1.95	0.41
2001	Rudan	264	13520	0.12	NA	0.05	0.25	31.41	-0.06	1.87	0.41
2001	Hajiabad	265	9962	0.13	NA	0.05	0.27	29.29	-0.18	1.81	0.41
2001	Bastak	266	8997	0.18	0.23	0.08	0.36	24.19	-0.07	1.83	0.41
2001	Tehran	267	1551138	0.04	0.01	0.03	0.05	99.63	1.59	3.39	0.29
2001	Damavand	268	14758	0.34	NA	0.15	0.68	67.39	1.56	3.40	0.29
2001	Rey	269	34335	0.5	NA	0.23	0.95	30.21	1.54	3.43	0.29
2001	Shemiranat	270	6782	0.6	NA	0.27	1.17	54.00	1.45	3.61	0.29
2001	Varamin	271	75621	0.3	NA	0.14	0.56	77.17	1.62	3.53	0.29
2001	Shahriyar	272	113377	0.2	NA	0.09	0.39	80.06	1.59	3.60	0.29
2001	Eslamshahr	273	66189	0.2	NA	0.09	0.38	87.67	1.59	3.38	0.29
2001	Robatkarim	274	61950	0.12	NA	0.05	0.24	79.86	1.53	3.42	0.29
2001	Pakdasht	275	27803	0.14	NA	0.06	0.28	68.47	1.57	3.35	0.29
2001	Firuzkuh	276	7092	0.17	NA	0.07	0.36	37.57	1.51	3.49	0.29
2001	Ardebil	277	83318	0.15	0.1	0.08	0.25	80.53	-0.43	1.62	0.15
2001	Bilehsowar	278	8904	0.14	0.23	0.06	0.28	34.04	-0.57	1.71	0.15
2001	Khalkhal	279	17832	0.21	NA	0.07	0.48	41.28	-0.58	1.70	0.15
2001	Meshginshahr	280	26704	0.23	NA	0.1	0.44	37.40	-0.58	1.59	0.15
2001	Germi	281	15533	0.14	NA	0.06	0.28	27.45	-0.54	1.64	0.15
2001	Parsabad	282	21714	0.12	0.1	0.05	0.23	49.46	-0.59	1.59	0.15
2001	Kowsar	283	5354	0.13	NA	0.04	0.32	22.55	-0.59	1.48	0.15
2001	Namin	284	10542	0.14	NA	0.06	0.29	31.50	-0.47	1.74	0.15
2001	Neer	285	4827	0.13	NA	0.05	0.29	21.48	-0.54	1.63	0.15
2001	Qom	286	153786	0.17	0.1	0.1	0.25	91.57	2.25	2.83	0.21
2001	Bueenzahra	287	25322	0.11	0.16	0.05	0.21	31.63	0.41	2.49	0.28
2001	Takestan	288	26768	0.27	0.45	0.14	0.46	53.33	0.36	2.31	0.28
2001	Qazvin	289	111936	0.85	0.99	0.64	1.09	76.41	0.38	2.47	0.28
2001	Abyek	290	13452	0.18	0.16	0.08	0.34	43.94	0.36	2.42	0.28
2001	Bandare Gaz	291	9222	0.27	0.22	0.13	0.5	51.95	0.28	2.28	0.36
2001	Torkman	292	20134	0.22	0.2	0.11	0.39	59.27	0.28	2.27	0.36

2001	Aliabad	293	20021	0.25	NA	0.11	0.49	45.35	0.22	2.27	0.36
2001	Kordkuy	294	13061	0.27	0.31	0.13	0.49	46.83	0.30	2.55	0.36
2001	Gorgan	295	64525	0.46	0.48	0.29	0.68	66.59	0.32	2.39	0.36
2001	Gonbade Kavus	296	43606	0.22	0.14	0.12	0.36	49.23	0.30	2.50	0.36
2001	Minudasht	297	20057	0.15	0.1	0.07	0.29	31.08	0.26	2.36	0.36
2001	Aqqala	298	16104	0.12	0.13	0.05	0.24	28.58	0.30	2.31	0.36
2001	Kalaleh	299	20053	0.14	0.21	0.06	0.26	22.42	0.34	2.37	0.36
2001	Azadshahr	300	13712	0.2	NA	0.09	0.4	52.35	0.26	2.34	0.36
2001	Ramyan	301	12390	0.12	NA	0.05	0.25	38.59	0.25	2.31	0.36
2001	Esfarayen	302	20722	0.12	NA	0.05	0.24	34.74	-0.67	2.36	0.30
2001	Bojnurd	303	46931	0.21	NA	0.09	0.41	54.96	-0.73	2.36	0.30
2001	Jajarm	304	9457	0.13	NA	0.04	0.28	58.87	-0.74	2.49	0.30
2001	Shirvan	305	25544	0.16	NA	0.07	0.32	47.71	-0.76	2.44	0.30
2001	Faruj	306	9901	0.11	NA	0.04	0.24	22.99	-0.80	2.51	0.30
2001	Maneh va Semelqan	307	13397	0.15	NA	0.06	0.32	22.10	-0.72	2.47	0.30
2001	Birjand	308	44208	0.39	0.49	0.23	0.62	52.44	-2.11	2.17	0.26
2001	Sarbisheh	309	8469	0.17	NA	0.05	0.44	22.24	-2.15	2.05	0.26
2001	Qaenat	310	23273	0.14	NA	0.06	0.29	29.95	-0.25	2.27	0.26
2001	Nehbandan	311	7794	0.13	NA	0.04	0.31	22.74	-2.12	2.31	0.26
2001	Sarayan	312	6084	0.21	NA	0.08	0.45	57.32	-2.10	2.13	0.26
2001	Ferdows	313	12135	0.21	NA	0.09	0.41	63.88	-0.22	2.45	0.26
2001	Karaj	314	255193	0.08	0.02	0.05	0.13	96.04	1.50	3.36	0.70
2001	Savojbolagh	315	29203	0.05	NA	0.02	0.12	33.89	1.49	3.43	0.70
2001	Nazarabad	316	17916	0.17	NA	0.07	0.36	76.04	1.57	3.43	0.70
2002	Arak	1	106748	0.29	0.23	0.18	0.42	76.91	0.63	2.95	0.23
2002	Ashtiyan	2	4242	0.43	1.33	0.17	0.86	30.56	0.57	2.96	0.23
2002	Tafresh	3	13241	0.25	0.28	0.12	0.46	24.38	0.59	2.95	0.23
2002	Khomeyn	4	22970	0.19	0.09	0.1	0.34	48.43	0.63	2.69	0.23
2002	Delijan	5	8380	0.37	0.24	0.17	0.69	63.43	0.65	3.02	0.23
2002	Saveh	6	35975	0.2	0.06	0.1	0.34	64.01	0.59	3.01	0.23
2002	Shazand	7	23708	0.22	NA	0.09	0.44	27.27	0.62	2.75	0.23
2002	Mahalat	8	9768	0.32	0.2	0.15	0.6	77.36	0.61	2.95	0.23
2002	Zarandiyeh	9	10830	0.21	NA	0.08	0.43	53.27	0.64	2.81	0.23
2002	Komijan	10	7315	0.23	NA	0.09	0.5	37.77	0.58	2.98	0.23
2002	Astara	11	13469	0.24	0.15	0.11	0.45	58.58	-0.02	3.49	0.16
2002	Astanehye Ashrafiyeh	12	25167	0.37	0.46	0.2	0.62	41.38	0.03	3.31	0.16
2002	Bandar Anzali	13	29457	0.7	0.86	0.42	1.08	81.55	0.01	3.25	0.16
2002	Tavalesh	14	30139	0.27	0.41	0.14	0.47	29.74	-0.01	3.34	0.16
2002	Rasht	15	172038	0.63	0.65	0.48	0.8	65.82	-0.10	3.43	0.16
2002	Rudbar	16	20837	0.39	0.57	0.2	0.67	47.44	0.03	3.38	0.16
2002	Rudsar	17	34180	0.54	0.62	0.32	0.84	43.69	-0.01	3.53	0.16
2002	Sumehsara	18	28711	0.35	0.41	0.19	0.58	27.46	-0.01	3.38	0.16
2002	Fuman	19	22006	0.35	0.53	0.18	0.6	25.87	0.01	3.38	0.16
2002	Langrud	20	30227	0.29	0.13	0.16	0.5	57.91	-0.06	3.30	0.16
2002	Lahijan	21	36884	0.6	0.73	0.37	0.9	39.95	0.00	3.46	0.16
2002	Shaft	22	14321	0.22	0.14	0.1	0.41	11.54	-0.04	3.48	0.16
2002	Amlash	23	10509	0.26	0.18	0.12	0.5	30.13	-0.05	3.16	0.16

2002	Rezvanshahr	24	12215	0.26	NA	0.11	0.53	26.57	-0.05	3.44	0.16
2002	Siyahkal	25	10842	0.29	0.18	0.14	0.54	28.74	0.04	3.35	0.16
2002	Masal	26	9445	0.25	0.21	0.11	0.48	28.80	0.01	3.43	0.16
2002	Amol	27	65751	0.39	0.38	0.24	0.59	53.25	1.45	3.40	0.31
2002	Babol	28	91994	0.28	0.15	0.17	0.42	44.91	1.40	3.33	0.31
2002	Behshahr	29	37211	0.34	0.27	0.18	0.55	59.62	1.41	3.48	0.31
2002	Tonekabon	30	40095	0.31	0.25	0.17	0.5	45.91	1.44	3.48	0.31
2002	Ramsar	31	14975	0.46	0.39	0.23	0.79	70.18	1.40	3.46	0.31
2002	Sari	32	92761	0.58	0.62	0.4	0.79	48.55	1.46	3.58	0.31
2002	Savadkuh	33	14549	0.24	NA	0.1	0.48	45.30	1.47	3.57	0.31
2002	Qaemshahr	34	57872	0.34	0.31	0.21	0.53	56.89	1.47	3.45	0.31
2002	Nur	35	20356	0.25	0.3	0.12	0.44	37.32	1.41	3.55	0.31
2002	Noshahr	36	22152	0.19	0.27	0.09	0.35	33.78	1.44	3.48	0.31
2002	Babolsar	37	33142	0.25	0.06	0.13	0.42	49.81	1.41	3.49	0.31
2002	Mahmudabad	38	17125	0.31	0.36	0.15	0.56	30.07	1.48	3.51	0.31
2002	Neka	39	19126	0.26	0.21	0.13	0.47	33.75	1.49	3.52	0.31
2002	Chalus	40	23378	0.2	0.09	0.1	0.36	51.59	1.34	3.54	0.31
2002	Juybar	41	13709	0.29	0.15	0.14	0.54	38.46	1.45	3.57	0.31
2002	Ahar	42	26430	0.19	NA	0.08	0.38	60.56	-0.12	2.07	0.24
2002	Tabriz	43	290523	0.57	NA	0.27	1.09	93.32	-0.07	2.20	0.24
2002	Sarab	44	24565	0.26	NA	0.11	0.51	39.91	-0.07	2.06	0.24
2002	Maragheh	45	42246	0.18	0.05	0.09	0.3	67.85	-0.09	2.01	0.24
2002	Marand	46	44228	0.24	0.05	0.13	0.39	56.37	-0.11	2.07	0.24
2002	Miyaneh	47	36591	0.23	NA	0.11	0.45	46.41	-0.06	2.24	0.24
2002	Hashtrud	48	11740	0.21	0.17	0.09	0.39	25.79	-0.16	2.15	0.24
2002	Bonab	49	21963	0.29	NA	0.13	0.57	60.07	-0.17	2.16	0.24
2002	Bostanabad	50	16366	0.21	NA	0.09	0.42	17.35	-0.14	2.10	0.24
2002	Shabestar	51	26460	0.23	NA	0.1	0.46	46.25	-0.11	2.15	0.24
2002	Kalibar	52	15075	0.15	NA	0.06	0.3	13.10	-0.16	2.20	0.24
2002	Haris	53	12097	0.28	NA	0.12	0.56	44.31	-0.19	2.08	0.24
2002	Jolfa	54	10947	0.23	NA	0.09	0.47	58.97	-0.17	2.13	0.24
2002	Malekan	55	16941	0.18	NA	0.08	0.38	31.11	-0.20	2.21	0.24
2002	Azarshahr	56	19436	0.22	NA	0.09	0.43	59.25	-0.13	2.02	0.24
2002	Osku	57	15306	0.18	NA	0.08	0.37	48.90	-0.12	2.26	0.24
2002	Charoymaq	58	5426	0.14	NA	0.06	0.3	11.24	-0.14	2.30	0.24
2002	Varzaqan	59	8514	0.16	NA	0.06	0.33	9.81	-0.16	2.22	0.24
2002	Ajabshir	60	12050	0.22	NA	0.09	0.46	41.00	-0.18	2.10	0.24
2002	Orumiyeh	61	146803	0.4	0.38	0.28	0.54	68.35	0.03	1.95	0.29
2002	Piranshahr	62	14281	0.22	0.3	0.1	0.42	46.40	0.10	1.95	0.29
2002	Khoy	63	61511	0.16	0.1	0.09	0.27	64.51	0.09	1.96	0.29
2002	Sardasht	64	15078	0.14	0.14	0.06	0.27	44.23	0.06	1.99	0.29
2002	Salmas	65	28099	0.34	0.36	0.18	0.57	53.54	0.13	2.07	0.29
2002	Maku	66	26967	0.15	0.15	0.07	0.27	47.31	0.09	1.93	0.29
2002	Mahabad	67	32122	0.23	0.32	0.12	0.4	67.94	0.06	1.96	0.29
2002	Miyandoab	68	40914	0.2	0.15	0.1	0.34	49.92	0.13	2.02	0.29
2002	Naqadeh	69	20368	0.3	NA	0.13	0.59	66.23	0.02	1.99	0.29
2002	Bukan	70	31715	0.16	0.07	0.08	0.28	71.36	0.17	1.94	0.29

2002	Shahindezh	71	15870	0.21	NA	0.09	0.43	47.69	0.07	2.02	0.29
2002	Takab	72	14375	0.16	0.14	0.07	0.32	53.70	0.12	1.90	0.29
2002	Oshnaviyeh	73	9140	0.36	0.23	0.16	0.67	53.38	0.17	2.15	0.29
2002	Chaldoran	74	6653	0.19	NA	0.07	0.43	33.80	0.02	2.19	0.29
2002	Eslamabade Gharb	75	34517	0.26	0.47	0.14	0.44	50.27	-0.32	2.31	0.19
2002	Kermanshah	76	166601	0.71	0.74	0.55	0.89	83.94	-0.26	2.14	0.19
2002	Paveh	77	9600	0.24	0.21	0.11	0.46	50.04	-0.18	2.22	0.19
2002	Sarpole Zahab	78	12905	0.28	0.48	0.13	0.52	39.39	-0.26	2.17	0.19
2002	Sonqor	79	18867	0.27	0.31	0.13	0.47	45.50	-0.25	2.20	0.19
2002	Qasreshirin	80	3759	0.37	NA	0.15	0.79	67.95	-0.22	2.26	0.19
2002	Kangavar	81	14877	0.34	0.54	0.17	0.6	60.19	-0.24	2.21	0.19
2002	Gilanegharb	82	11232	0.2	0.37	0.09	0.39	32.03	-0.28	2.23	0.19
2002	Javanrud	83	15934	0.19	NA	0.08	0.4	51.39	-0.22	2.20	0.19
2002	Sahneh	84	14634	0.27	0.41	0.13	0.51	40.11	-0.26	2.19	0.19
2002	Harsin	85	14908	0.24	0.14	0.11	0.44	64.62	-0.15	2.27	0.19
2002	Salas-e-Babajani	86	4626	0.16	NA	0.05	0.37	18.72	-0.19	2.21	0.19
2002	Abadan	87	44401	0.75	0.71	0.48	1.08	84.35	1.08	2.84	0.36
2002	Andimeshk	88	24060	0.31	0.18	0.16	0.53	75.91	1.06	2.60	0.36
2002	Ahvaz	89	195889	1.1	1.13	0.9	1.31	82.27	1.08	2.83	0.36
2002	Izeh	90	26286	0.25	0.32	0.13	0.44	49.84	1.10	2.68	0.36
2002	Bandar-e-Mahshahr	91	34153	0.68	0.76	0.41	1.03	94.15	1.02	2.81	0.36
2002	Behbahan	92	29822	0.45	0.41	0.25	0.73	71.81	1.07	2.67	0.36
2002	Khorramshahr	93	22826	0.6	0.64	0.33	0.97	83.82	1.14	2.74	0.36
2002	Dezful	94	58153	0.4	0.36	0.24	0.61	73.22	1.10	2.68	0.36
2002	Dashte Azadegan	95	17037	0.35	0.25	0.18	0.62	55.73	1.03	2.80	0.36
2002	Ramhormoz	96	25439	0.4	0.41	0.22	0.67	51.87	1.08	2.89	0.36
2002	Shadegan	97	17526	0.33	0.12	0.16	0.58	38.37	0.99	2.74	0.36
2002	Shushtar	98	34397	0.5	0.55	0.28	0.79	59.68	1.04	2.89	0.36
2002	Masjedsoleyman	99	28094	0.51	0.73	0.29	0.83	66.31	1.11	2.82	0.36
2002	Shush	100	25688	0.4	0.42	0.21	0.66	45.73	1.12	2.86	0.36
2002	Baghmalek	101	13277	0.28	0.46	0.13	0.52	33.74	1.09	2.85	0.36
2002	Omidiyeh	102	12917	0.4	0.33	0.2	0.71	69.28	1.14	2.73	0.36
2002	Lali	103	4820	0.34	NA	0.12	0.73	47.44	1.05	2.85	0.36
2002	Hendijan	104	5877	0.59	1.02	0.27	1.11	68.64	1.10	2.87	0.36
2002	Abadeh	105	18353	0.67	0.87	0.37	1.1	88.19	0.71	2.72	0.29
2002	Estahban	106	11368	0.34	0.18	0.16	0.61	71.44	0.58	2.78	0.29
2002	Eqlid	107	15720	0.32	0.13	0.16	0.56	63.88	0.58	2.93	0.29
2002	Jahrom	108	34929	0.38	0.35	0.21	0.61	63.76	0.60	2.79	0.29
2002	Darab	109	26459	0.35	0.31	0.19	0.58	42.27	0.69	2.91	0.29
2002	Sepidan	110	13894	0.23	NA	0.1	0.46	18.86	0.60	2.79	0.29
2002	Shiraz	111	289147	1.21	1.24	1.03	1.39	87.58	0.64	2.88	0.29
2002	Fasa	112	32718	0.42	0.37	0.24	0.67	57.12	0.66	2.80	0.29
2002	Firuzabad	113	20014	0.31	0.2	0.16	0.54	53.67	0.66	2.85	0.29
2002	Kazerun	114	45280	0.24	0.14	0.13	0.4	51.45	0.68	2.82	0.29
2002	Lar (Larestan)	115	42030	0.45	0.44	0.26	0.7	57.47	0.68	2.91	0.29
2002	Marvdasht	116	49989	0.24	0.17	0.13	0.39	44.67	0.66	2.80	0.29
2002	Mamasany	117	29342	0.17	0.07	0.09	0.3	30.09	0.62	2.75	0.29

2002	Neyriz	118	17017	0.28	0.12	0.14	0.49	58.51	0.61	2.80	0.29
2002	Lamard	119	11364	0.37	0.54	0.18	0.68	42.38	0.73	2.80	0.29
2002	Bovanat	120	8144	0.24	NA	0.1	0.51	29.43	0.63	2.76	0.29
2002	Arsanjan	121	6226	0.48	NA	0.2	0.98	39.35	0.70	2.82	0.29
2002	Khorrambid	122	7248	0.31	NA	0.13	0.65	76.64	0.69	2.78	0.29
2002	Zarrindasht	123	8558	0.29	NA	0.11	0.62	57.38	0.58	2.71	0.29
2002	Qirokarzin	124	9434	0.25	NA	0.1	0.52	56.23	0.64	2.91	0.29
2002	Mohr	125	7313	0.27	NA	0.11	0.56	35.77	0.61	2.71	0.29
2002	Farashband	126	6650	0.33	NA	0.14	0.67	57.73	0.75	2.81	0.29
2002	Baft	127	23529	0.28	0.34	0.14	0.48	34.69	0.14	3.09	0.71
2002	Bam	128	34607	0.22	0.18	0.12	0.38	41.56	0.04	3.04	0.71
2002	Jiroft	129	30053	0.29	0.35	0.15	0.5	39.13	0.05	3.04	0.71
2002	Rafsanjan	130	45165	0.71	0.87	0.46	1.02	55.19	0.02	3.00	0.71
2002	Zarand	131	24140	0.33	0.25	0.17	0.56	51.02	0.09	2.98	0.71
2002	Sirjan	132	37581	0.79	1.04	0.49	1.15	74.27	0.12	3.03	0.71
2002	Shahrebabak	133	14797	0.52	0.94	0.26	0.89	61.47	0.08	2.99	0.71
2002	Kerman	134	105147	0.75	0.79	0.56	0.99	87.01	0.09	3.19	0.71
2002	Kahnuj	135	27281	0.16	0.08	0.08	0.28	20.51	0.04	3.03	0.71
2002	Bardsir	136	13166	0.22	0.15	0.1	0.42	54.07	0.02	3.03	0.71
2002	Ravar	137	6855	0.32	NA	0.13	0.69	59.44	0.07	3.12	0.71
2002	Anbarabad	138	9012	0.21	NA	0.07	0.49	34.12	0.18	2.91	0.71
2002	Manujan	139	7163	0.17	NA	0.06	0.39	22.92	0.16	2.93	0.71
2002	Taybad	140	19210	0.18	0.11	0.08	0.34	44.13	0.01	2.43	0.40
2002	Torbate Heydarieh	141	52985	0.3	0.3	0.18	0.48	44.38	-0.06	2.51	0.40
2002	Torbate Jam	142	32830	0.23	0.13	0.12	0.4	42.40	-0.03	2.31	0.40
2002	Darrehgaz	143	14126	0.27	0.28	0.13	0.5	54.16	0.04	2.42	0.40
2002	Sabzevar	144	79576	0.19	0.07	0.11	0.3	47.41	-0.05	2.39	0.40
2002	Quchan	145	32133	0.2	0.06	0.1	0.34	48.45	-0.03	2.59	0.40
2002	Kashmar	146	34291	0.3	NA	0.13	0.58	47.05	-0.02	2.45	0.40
2002	Gonabad	147	21354	0.34	0.37	0.17	0.58	47.66	-0.06	2.49	0.40
2002	Mashhad	148	452684	0.27	0.24	0.2	0.33	89.88	0.04	2.52	0.40
2002	Neyshabur	149	76237	0.22	0.16	0.13	0.34	49.89	-0.06	2.53	0.40
2002	Chenaran	150	17290	0.24	0.35	0.12	0.44	37.57	-0.07	2.43	0.40
2002	Khaf	151	14643	0.2	0.14	0.09	0.36	44.66	-0.07	2.46	0.40
2002	Sarakhs	152	11517	0.28	0.19	0.13	0.53	39.77	0.12	2.52	0.40
2002	Fariman	153	13375	0.27	0.31	0.13	0.51	53.23	-0.02	2.26	0.40
2002	Bardeskan	154	12225	0.16	0.16	0.07	0.32	34.09	-0.08	2.30	0.40
2002	Rashtkhar	155	8576	0.17	NA	0.06	0.4	18.12	0.03	2.37	0.40
2002	Kalat	156	6675	0.29	NA	0.11	0.65	23.88	-0.01	2.38	0.40
2002	Ardestan	157	9854	0.28	0.19	0.12	0.53	53.90	1.17	3.32	0.19
2002	Isfahan	158	352098	1.3	1.32	1.14	1.47	91.27	1.15	3.19	0.19
2002	Khomeynishahr	159	44579	0.54	0.66	0.33	0.81	94.71	1.16	3.09	0.19
2002	Khansar	160	7604	0.32	0.49	0.15	0.58	61.15	1.22	3.25	0.19
2002	Semirom	161	12044	0.43	1.01	0.21	0.8	51.34	1.19	3.16	0.19
2002	Faridan	162	16174	0.5	0.72	0.26	0.86	39.49	1.22	3.23	0.19
2002	Fereydunshahr	163	7125	0.24	NA	0.1	0.49	45.96	1.28	3.21	0.19
2002	Falavarjan	164	36623	0.28	0.17	0.15	0.47	61.19	1.22	3.05	0.19

2002	Shahreza	165	33945	0.46	0.53	0.26	0.72	77.88	1.19	3.17	0.19
2002	Kashan	166	55078	0.9	0.99	0.62	1.23	84.46	1.15	3.25	0.19
2002	Golpayegan	167	17780	0.33	0.32	0.16	0.56	72.44	1.16	3.20	0.19
2002	Lanjan	168	38091	0.55	0.6	0.33	0.84	81.44	1.20	3.23	0.19
2002	Nayin	169	11724	0.39	0.65	0.19	0.7	65.15	1.16	3.36	0.19
2002	Najafabad	170	47438	0.65	0.7	0.42	0.96	91.51	1.16	3.33	0.19
2002	Natanz	171	8743	0.31	0.44	0.14	0.59	56.12	1.13	3.04	0.19
2002	Shahinshahr va Meyme	172	47550	0.67	0.73	0.43	0.97	88.83	1.19	3.20	0.19
2002	Mobarakeh	173	22353	0.3	0.19	0.15	0.52	81.02	1.21	3.20	0.19
2002	Aran va Bidgol	174	15879	0.39	0.39	0.2	0.68	85.41	1.14	3.21	0.19
2002	Tiran va Karvan	175	12125	0.26	0.49	0.12	0.49	36.81	1.16	3.20	0.19
2002	Chadegan	176	6309	0.21	NA	0.08	0.46	38.91	1.22	3.05	0.19
2002	Iranshahr	177	28006	0.19	0.08	0.09	0.35	42.31	-1.80	1.72	0.63
2002	Chah Bahar	178	24780	0.15	0.09	0.07	0.29	31.12	-1.83	1.82	0.63
2002	Khash	179	16568	0.16	NA	0.06	0.33	29.23	-1.83	1.70	0.63
2002	Zabol	180	51551	0.18	NA	0.08	0.36	36.22	-1.85	1.80	0.63
2002	Zahedan	181	68814	0.37	NA	0.17	0.72	87.85	-1.79	1.62	0.63
2002	Saravan	182	29664	0.11	NA	0.05	0.23	35.53	-1.80	1.58	0.63
2002	Nikshahr	183	22313	0.12	NA	0.05	0.25	21.30	-1.91	1.49	0.63
2002	Sarbaz	184	14436	0.14	NA	0.04	0.37	11.49	-1.77	1.82	0.63
2002	Baneh	185	17510	0.2	0.12	0.09	0.37	59.32	-0.37	1.77	0.36
2002	Bijar	186	19663	0.26	0.6	0.12	0.47	50.63	-0.34	1.66	0.36
2002	Saqqez	187	34921	0.26	0.35	0.14	0.44	63.01	-0.39	1.75	0.36
2002	Sanandaj	188	68797	0.37	0.33	0.23	0.55	80.55	-0.38	1.68	0.36
2002	Qorveh	189	33701	0.16	0.06	0.08	0.29	49.78	-0.33	1.45	0.36
2002	Marivan	190	20528	0.28	NA	0.12	0.56	58.56	-0.32	1.79	0.36
2002	Divandarreh	191	13065	0.18	NA	0.07	0.36	24.71	-0.28	1.78	0.36
2002	Kamyaran	192	16583	0.18	NA	0.07	0.37	41.45	-0.29	1.78	0.36
2002	Sarvabad	193	9429	0.13	NA	0.05	0.28	4.54	-0.37	1.64	0.36
2002	Tuyserkan	194	22499	0.19	0.09	0.09	0.34	42.92	0.16	2.23	0.13
2002	Malayer	195	53165	0.19	0.07	0.1	0.3	55.49	0.27	2.20	0.13
2002	Nahavand	196	32822	0.17	0.06	0.08	0.3	48.25	0.16	2.29	0.13
2002	Hamadan	197	112143	0.19	0.09	0.12	0.29	80.79	0.21	2.23	0.13
2002	Kabudarahang	198	22977	0.15	0.18	0.07	0.28	16.05	0.15	2.37	0.13
2002	Asadabad	199	18226	0.23	NA	0.1	0.45	46.13	0.27	2.27	0.13
2002	Bahar	200	21490	0.2	0.09	0.1	0.36	41.61	0.14	2.19	0.13
2002	Razan	201	20661	0.18	NA	0.08	0.37	17.49	0.15	2.35	0.13
2002	Borujen	202	19769	0.21	0.1	0.1	0.39	80.39	-0.25	1.97	0.20
2002	Shahrekord	203	65374	0.23	0.13	0.13	0.37	71.45	-0.15	2.06	0.20
2002	Farsan	204	12764	0.22	0.16	0.1	0.41	62.19	-0.15	2.08	0.20
2002	Lordakan	205	21643	0.18	NA	0.08	0.35	18.33	-0.19	2.17	0.20
2002	Ardal	206	7401	0.16	NA	0.05	0.37	14.73	-0.22	2.01	0.20
2002	Kuhrang	207	4347	0.14	NA	0.05	0.31	6.17	-0.12	2.10	0.20
2002	Aligudarz	208	21038	0.19	0.1	0.09	0.35	56.64	-0.04	2.26	0.21
2002	Borujerd	209	59100	0.51	0.54	0.33	0.75	75.14	-0.09	2.35	0.21
2002	Khorramabad	210	78797	0.36	0.29	0.22	0.53	66.33	-0.03	2.32	0.21
2002	Dalfan	211	17884	0.16	0.12	0.08	0.31	40.43	-0.02	2.33	0.21

2002	Dorud	212	24455	0.32	0.43	0.16	0.54	65.29	-0.16	2.50	0.21
2002	Kuhdasht	213	27903	0.17	NA	0.07	0.33	48.99	-0.07	2.28	0.21
2002	Azna	214	12571	0.21	0.16	0.1	0.41	51.48	-0.08	2.41	0.21
2002	Poldokhtar	215	10970	0.24	0.19	0.11	0.46	35.84	-0.08	2.35	0.21
2002	Selseleh	216	10651	0.25	NA	0.08	0.59	38.58	-0.06	2.22	0.21
2002	Ilam	217	28792	0.37	0.3	0.19	0.62	80.73	0.25	1.91	0.29
2002	Darrehshahr	218	8564	0.16	NA	0.06	0.35	35.97	0.30	2.04	0.29
2002	Dehloran	219	8388	0.26	NA	0.1	0.54	62.81	0.29	2.10	0.29
2002	Shirvan va Chardavol	220	11336	0.17	NA	0.07	0.35	18.97	0.22	2.04	0.29
2002	Mehran	221	7574	0.33	NA	0.13	0.68	57.17	0.25	2.08	0.29
2002	Abdanan	222	6937	0.21	NA	0.08	0.42	57.65	0.21	1.90	0.29
2002	Eyvan	223	7886	0.4	NA	0.17	0.81	60.29	0.29	2.21	0.29
2002	Boyerahmad	224	25519	0.28	NA	0.12	0.56	39.56	-0.38	2.16	0.31
2002	Kohgiluyeh	225	30436	0.15	NA	0.06	0.31	34.87	-0.38	2.19	0.31
2002	Gachsaran	226	21476	0.25	0.1	0.12	0.45	60.64	-0.46	2.09	0.31
2002	Dena	227	8110	0.19	NA	0.08	0.38	19.38	-0.43	2.10	0.31
2002	Bushehr	228	32456	1.29	1.48	0.86	1.83	85.42	0.90	2.59	0.74
2002	Tangestan	229	10529	0.29	NA	0.12	0.59	22.65	0.88	2.54	0.74
2002	Dashtestan	230	36922	0.32	0.33	0.18	0.52	60.81	0.83	2.61	0.74
2002	Dashti	231	12095	0.27	0.17	0.13	0.51	52.02	0.90	2.61	0.74
2002	Dayyer	232	6952	0.31	NA	0.13	0.63	68.05	0.90	2.48	0.74
2002	Kangan	233	8206	0.41	0.78	0.18	0.78	68.00	1.00	2.68	0.74
2002	Genaveh	234	13531	0.58	0.76	0.3	1.01	73.15	0.98	2.61	0.74
2002	Deylam	235	4690	0.45	NA	0.17	0.96	71.91	0.87	2.61	0.74
2002	Jam	236	4480	0.21	NA	0.08	0.47	33.62	0.91	2.53	0.74
2002	Abhar	237	28388	0.19	0.14	0.09	0.34	60.33	0.24	1.92	0.23
2002	Khodabandeh	238	25848	0.15	NA	0.06	0.29	24.03	0.18	1.88	0.23
2002	Zanjan	239	76014	0.37	0.38	0.23	0.54	75.55	0.21	2.06	0.23
2002	Ijrud	240	6794	0.15	NA	0.06	0.33	6.36	0.23	1.94	0.23
2002	Khorramdarreh	241	10228	0.3	NA	0.13	0.61	77.92	0.19	2.03	0.23
2002	Tarom	242	7468	0.16	NA	0.06	0.37	12.30	0.29	1.98	0.23
2002	Mahneshan	243	7131	0.16	NA	0.06	0.36	11.87	0.17	1.78	0.23
2002	Damghan	244	16108	0.43	0.49	0.22	0.74	64.25	2.07	3.95	0.30
2002	Semnan	245	31879	0.3	0.19	0.16	0.5	87.77	2.06	4.19	0.30
2002	Shahrud	246	42161	0.69	0.81	0.43	1.02	60.87	2.01	4.12	0.30
2002	Garmsar	247	14908	0.35	0.27	0.17	0.61	63.11	2.09	4.06	0.30
2002	Ardakan	248	11341	0.35	0.18	0.17	0.62	79.82	1.26	3.11	0.36
2002	Bafq	249	8172	0.46	0.75	0.22	0.84	64.76	1.36	3.10	0.36
2002	Taft	250	11154	0.36	0.66	0.17	0.66	29.34	1.23	3.05	0.36
2002	Mehriz	251	8352	0.52	0.7	0.25	0.93	52.13	1.24	3.14	0.36
2002	Yazd	252	78835	1.36	1.45	1.04	1.73	95.56	1.29	3.02	0.36
2002	Meybod	253	11309	0.53	0.72	0.26	0.93	90.30	1.23	3.12	0.36
2002	Abarkuh	254	7747	0.46	0.76	0.21	0.85	62.17	1.25	2.91	0.36
2002	Sadugh	255	5101	0.42	0.38	0.17	0.86	51.25	1.24	3.17	0.36
2002	Khatam	256	5144	0.33	NA	0.13	0.71	54.26	1.22	3.13	0.36
2002	Tabas	257	10632	0.38	0.58	0.18	0.69	52.05	1.34	3.05	0.36
2002	Abumusa	258	124	0.35	NA	0.13	0.78	60.00	0.01	1.96	0.40

2002	Bandarabbas	259	72592	0.56	0.64	0.37	0.81	66.97	0.01	2.05	0.40
2002	Bandar-e Lengeh	260	19186	0.24	0.22	0.13	0.42	46.09	0.01	2.01	0.40
2002	Qeshm	261	12239	0.25	0.17	0.12	0.47	38.22	0.02	1.98	0.40
2002	Minab	262	33874	0.2	0.18	0.1	0.35	23.37	0.03	2.07	0.40
2002	Bandar-e-Jask	263	9204	0.18	0.22	0.09	0.32	15.87	0.01	2.08	0.40
2002	Rudan	264	14111	0.15	NA	0.06	0.32	31.94	0.00	1.95	0.40
2002	Hajiabad	265	10273	0.17	0.2	0.07	0.34	29.51	0.07	2.05	0.40
2002	Bastak	266	9359	0.31	0.88	0.14	0.59	24.06	0.04	1.94	0.40
2002	Tehran	267	1604547	1.13	1.13	1.06	1.2	99.63	1.74	3.61	0.31
2002	Damavand	268	15555	0.47	0.53	0.24	0.81	68.14	1.72	3.70	0.31
2002	Rey	269	36873	0.99	1.23	0.64	1.43	29.43	1.68	3.74	0.31
2002	Shemiranat	270	7056	0.78	0.84	0.39	1.37	54.26	1.67	3.57	0.31
2002	Varamin	271	79361	0.48	0.54	0.32	0.69	77.69	1.67	3.68	0.31
2002	Shahriyar	272	124152	0.21	0.18	0.13	0.32	81.03	1.70	3.62	0.31
2002	Eslamshahr	273	68933	0.21	0.16	0.12	0.33	88.01	1.63	3.70	0.31
2002	Robatkarim	274	68027	0.13	0.07	0.07	0.22	80.45	1.69	3.53	0.31
2002	Pakdasht	275	29645	0.18	0.16	0.09	0.33	69.72	1.75	3.70	0.31
2002	Firuzkuh	276	7356	0.23	NA	0.09	0.49	37.63	1.65	3.58	0.31
2002	Ardebil	277	86950	0.25	0.24	0.15	0.37	80.84	-0.29	1.72	0.19
2002	Bilehsowar	278	9156	0.17	NA	0.07	0.37	34.53	-0.23	1.74	0.19
2002	Khalkhal	279	18144	0.28	NA	0.1	0.63	41.87	-0.26	1.83	0.19
2002	Meshginshahr	280	27431	0.29	0.29	0.15	0.49	38.13	-0.26	1.58	0.19
2002	Germi	281	15818	0.18	NA	0.08	0.38	27.90	-0.25	1.78	0.19
2002	Parsabad	282	22913	0.15	0.09	0.07	0.28	49.96	-0.23	1.78	0.19
2002	Kowsar	283	5415	0.17	NA	0.05	0.42	22.96	-0.24	1.57	0.19
2002	Namin	284	10726	0.19	NA	0.08	0.39	32.06	-0.29	1.93	0.19
2002	Neer	285	4859	0.18	0.39	0.07	0.39	21.84	-0.31	1.67	0.19
2002	Qom	286	160441	0.52	0.55	0.38	0.68	91.84	2.27	3.04	0.27
2002	Bueenzahra	287	26063	0.13	0.08	0.06	0.24	32.09	0.60	2.46	0.31
2002	Takestan	288	27715	0.28	0.29	0.15	0.48	54.01	0.61	2.56	0.31
2002	Qazvin	289	117217	0.25	0.16	0.16	0.37	76.91	0.65	2.67	0.31
2002	Abyek	290	14010	0.25	NA	0.11	0.51	44.95	0.63	2.67	0.31
2002	Bandare Gaz	291	9473	0.41	0.63	0.19	0.73	52.21	0.40	2.37	0.40
2002	Torkman	292	20799	0.35	0.49	0.18	0.6	59.46	0.42	2.34	0.40
2002	Aliabad	293	20676	0.32	0.3	0.16	0.56	45.74	0.44	2.43	0.40
2002	Kordkuy	294	13421	0.3	0.15	0.15	0.54	47.10	0.53	2.44	0.40
2002	Gorgan	295	67415	0.87	0.99	0.62	1.18	67.34	0.49	2.37	0.40
2002	Gonbade Kavus	296	45433	0.37	0.42	0.21	0.59	49.15	0.47	2.45	0.40
2002	Minudasht	297	20733	0.19	0.1	0.09	0.34	31.52	0.50	2.25	0.40
2002	Aqqala	298	16729	0.16	0.13	0.07	0.3	28.75	0.48	2.53	0.40
2002	Kalaleh	299	21007	0.17	0.2	0.08	0.32	22.46	0.51	2.45	0.40
2002	Azadshahr	300	14206	0.27	0.29	0.13	0.49	52.75	0.43	2.37	0.40
2002	Ramyan	301	12828	0.16	NA	0.06	0.32	38.79	0.46	2.41	0.40
2002	Esfarayen	302	21353	0.16	NA	0.07	0.32	35.44	-0.50	2.45	0.35
2002	Bojnurd	303	48746	0.28	NA	0.13	0.55	55.53	-0.46	2.55	0.35
2002	Jajarm	304	9740	0.16	NA	0.06	0.36	59.30	-0.45	2.46	0.35
2002	Shirvan	305	26420	0.21	NA	0.09	0.42	48.17	-0.53	2.59	0.35

2002	Faruj	306	10035	0.14	NA	0.05	0.31	23.34	-0.50	2.40	0.35
2002	Maneh va Semelqan	307	13914	0.19	NA	0.07	0.41	22.38	-0.47	2.47	0.35
2002	Birjand	308	45750	0.42	NA	0.19	0.81	53.10	-1.77	2.25	0.26
2002	Sarbisheh	309	8701	0.23	NA	0.07	0.59	22.35	-1.83	2.38	0.26
2002	Qaenat	310	23923	0.19	NA	0.08	0.39	30.51	-0.03	2.56	0.26
2002	Nehbandan	311	8150	0.17	NA	0.05	0.41	23.21	-1.79	2.29	0.26
2002	Sarayan	312	6297	0.27	NA	0.1	0.6	57.40	-1.75	2.28	0.26
2002	Ferdows	313	12458	0.27	NA	0.12	0.54	64.32	-0.01	2.61	0.26
2002	Karaj	314	271497	0.13	0.08	0.08	0.19	96.15	1.74	3.55	0.63
2002	Savojbolagh	315	31454	0.07	NA	0.02	0.15	34.29	1.74	3.65	0.63
2002	Nazarabad	316	18901	0.22	NA	0.09	0.46	76.52	1.67	3.63	0.63
2003	Arak	1	110662	0.51	0.52	0.36	0.7	77.51	0.88	2.96	0.28
2003	Ashtiyan	2	4291	0.42	NA	0.16	0.91	31.06	0.89	3.00	0.28
2003	Tafresh	3	13381	0.27	0.14	0.13	0.5	24.84	0.90	3.15	0.28
2003	Khomeyn	4	23464	0.23	0.08	0.12	0.41	49.52	0.81	2.96	0.28
2003	Delijan	5	8633	0.5	NA	0.22	1.01	64.34	0.83	3.08	0.28
2003	Saveh	6	37794	0.39	0.5	0.22	0.62	65.11	0.84	2.79	0.28
2003	Shazand	7	24295	0.28	NA	0.12	0.55	27.76	0.90	3.00	0.28
2003	Mahalat	8	10038	0.42	NA	0.18	0.85	77.96	0.82	2.88	0.28
2003	Zarandiyeh	9	11137	0.26	NA	0.11	0.54	53.89	0.88	2.94	0.28
2003	Komijan	10	7496	0.28	NA	0.11	0.6	38.00	0.93	3.02	0.28
2003	Astara	11	14067	0.28	0.15	0.13	0.51	59.14	0.21	3.42	0.21
2003	Astanehye Ashrafiyeh	12	25749	0.39	0.38	0.21	0.64	41.95	0.24	3.49	0.21
2003	Bandar Anzali	13	30341	0.51	0.38	0.3	0.81	81.73	0.18	3.42	0.21
2003	Tavalesh	14	31405	0.27	0.26	0.14	0.45	30.30	0.22	3.55	0.21
2003	Rasht	15	178991	0.64	0.63	0.49	0.8	66.42	0.16	3.40	0.21
2003	Rudbar	16	21334	0.33	0.19	0.17	0.56	48.14	0.26	3.44	0.21
2003	Rudsar	17	35109	0.65	0.72	0.4	0.98	43.27	0.22	3.60	0.21
2003	Sumehsara	18	29354	0.4	0.4	0.22	0.64	28.09	0.12	3.40	0.21
2003	Fuman	19	22531	0.37	0.43	0.19	0.62	25.54	0.24	3.36	0.21
2003	Langrud	20	31078	0.76	1.01	0.47	1.15	58.43	0.21	3.42	0.21
2003	Lahijan	21	38006	0.59	0.61	0.36	0.89	40.87	0.18	3.34	0.21
2003	Shaft	22	14609	0.31	0.4	0.15	0.56	11.67	0.28	3.28	0.21
2003	Amlash	23	10783	0.38	0.54	0.17	0.72	30.39	0.13	3.38	0.21
2003	Rezvanshahr	24	12648	0.32	0.32	0.15	0.58	26.97	0.27	3.49	0.21
2003	Siyahkal	25	11068	0.35	0.17	0.16	0.63	29.31	0.17	3.43	0.21
2003	Masal	26	9738	0.33	0.41	0.15	0.61	28.26	0.26	3.45	0.21
2003	Amol	27	68267	0.43	0.39	0.27	0.64	53.60	1.52	3.46	0.33
2003	Babol	28	95191	0.86	0.91	0.64	1.13	45.24	1.67	3.52	0.33
2003	Behshahr	29	38482	0.45	0.42	0.26	0.69	59.88	1.63	3.64	0.33
2003	Tonekabon	30	41441	0.49	0.53	0.29	0.75	46.22	1.57	3.66	0.33
2003	Ramsar	31	15386	0.61	0.64	0.32	1.02	70.43	1.69	3.74	0.33
2003	Sari	32	96377	0.85	0.93	0.63	1.11	49.12	1.67	3.46	0.33
2003	Savadkuh	33	14898	0.3	NA	0.12	0.61	45.75	1.58	3.72	0.33
2003	Qaemshahr	34	59907	0.43	0.4	0.26	0.64	57.34	1.54	3.56	0.33
2003	Nur	35	21079	0.29	0.29	0.14	0.5	37.62	1.67	3.70	0.33
2003	Noshahr	36	22986	0.18	0.09	0.08	0.33	34.05	1.67	3.38	0.33

2003	Babolsar	37	34335	0.35	0.24	0.19	0.57	50.23	1.60	3.70	0.33
2003	Mahmudabad	38	17826	0.3	0.12	0.15	0.54	30.53	1.61	3.65	0.33
2003	Neka	39	19778	0.34	NA	0.14	0.68	34.28	1.67	3.56	0.33
2003	Chalus	40	24183	0.27	0.25	0.14	0.48	51.60	1.64	3.56	0.33
2003	Juybar	41	14134	0.4	NA	0.17	0.81	38.52	1.53	3.61	0.33
2003	Ahar	42	27184	0.19	0.07	0.09	0.35	60.97	0.13	2.22	0.30
2003	Tabriz	43	301882	0.34	0.3	0.25	0.43	93.28	0.07	2.21	0.30
2003	Sarab	44	25043	0.29	0.24	0.15	0.5	40.45	0.06	2.26	0.30
2003	Maragheh	45	43611	0.2	0.05	0.11	0.34	68.04	0.10	2.40	0.30
2003	Marand	46	45604	0.56	0.62	0.34	0.83	56.92	0.11	2.22	0.30
2003	Miyaneh	47	37234	0.29	0.31	0.16	0.49	47.18	0.19	2.29	0.30
2003	Hashtrud	48	11831	0.25	0.17	0.12	0.47	26.55	0.11	2.32	0.30
2003	Bonab	49	22811	0.3	0.18	0.15	0.52	60.30	0.00	2.34	0.30
2003	Bostanabad	50	16611	0.26	NA	0.11	0.53	17.80	0.10	2.36	0.30
2003	Shabestar	51	27120	0.22	0.07	0.11	0.39	46.39	0.17	2.34	0.30
2003	Kalibar	52	15419	0.18	NA	0.07	0.38	13.32	0.09	2.20	0.30
2003	Haris	53	12345	0.35	NA	0.15	0.69	44.53	0.18	2.25	0.30
2003	Jolfa	54	11220	0.26	0.18	0.11	0.52	59.37	0.17	2.28	0.30
2003	Malekan	55	17523	0.2	0.12	0.09	0.37	31.33	0.09	2.15	0.30
2003	Azarshahr	56	20021	0.28	NA	0.12	0.55	59.38	0.17	2.37	0.30
2003	Osku	57	16024	0.23	NA	0.09	0.45	49.77	0.18	2.23	0.30
2003	Charoymaq	58	5464	0.17	NA	0.07	0.37	11.58	0.21	2.17	0.30
2003	Varzaqan	59	8674	0.2	0.23	0.09	0.39	9.96	0.10	2.36	0.30
2003	Ajabshir	60	12449	0.28	NA	0.11	0.56	41.27	0.20	2.12	0.30
2003	Orumiyeh	61	152872	0.63	0.64	0.48	0.81	68.90	0.40	2.12	0.33
2003	Piranshahr	62	15000	0.27	0.28	0.12	0.49	47.61	0.34	2.25	0.33
2003	Khoy	63	63680	0.25	0.25	0.14	0.39	64.83	0.33	2.04	0.33
2003	Sardasht	64	15593	0.16	0.13	0.07	0.31	44.84	0.40	1.98	0.33
2003	Salmas	65	29100	0.35	0.28	0.19	0.58	53.82	0.36	2.10	0.33
2003	Maku	66	27871	0.17	0.15	0.08	0.31	47.81	0.41	2.11	0.33
2003	Mahabad	67	33473	0.27	0.31	0.14	0.46	68.23	0.37	2.25	0.33
2003	Miyandoab	68	42429	0.3	0.34	0.16	0.48	50.39	0.39	2.03	0.33
2003	Naqadeh	69	21074	0.37	0.39	0.19	0.64	66.66	0.39	2.02	0.33
2003	Bukan	70	33111	0.26	0.31	0.13	0.44	71.75	0.36	2.15	0.33
2003	Shahindezh	71	16322	0.26	NA	0.11	0.53	48.33	0.35	2.19	0.33
2003	Takab	72	14702	0.2	0.13	0.09	0.37	53.99	0.40	2.15	0.33
2003	Oshnaviyeh	73	9568	0.42	0.22	0.2	0.78	53.56	0.40	2.13	0.33
2003	Chaldoran	74	6825	0.24	NA	0.08	0.53	34.06	0.36	2.20	0.33
2003	Eslamabade Gharb	75	35613	0.22	0.23	0.11	0.37	50.67	0.04	2.33	0.25
2003	Kermanshah	76	172902	0.69	0.69	0.53	0.86	84.04	-0.08	2.33	0.25
2003	Paveh	77	9952	0.29	0.2	0.13	0.55	50.02	-0.07	2.42	0.25
2003	Sarpole Zahab	78	13461	0.33	0.46	0.15	0.61	39.78	-0.07	2.35	0.25
2003	Sonqor	79	19201	0.4	0.62	0.21	0.68	46.06	-0.01	2.31	0.25
2003	Qasreshirin	80	3889	0.52	1.05	0.22	1.03	68.27	-0.09	2.40	0.25
2003	Kangavar	81	15175	0.44	0.66	0.22	0.76	60.56	-0.07	2.37	0.25
2003	Gilanegharb	82	11612	0.22	NA	0.09	0.46	32.50	-0.08	2.25	0.25
2003	Javanrud	83	16585	0.24	0.25	0.11	0.45	52.20	-0.04	2.33	0.25

2003	Sahneh	84	14975	0.3	0.26	0.14	0.53	40.82	-0.02	2.31	0.25
2003	Harsin	85	15326	0.36	0.53	0.18	0.65	64.60	-0.10	2.31	0.25
2003	Salas-e-Babajani	86	4884	0.2	NA	0.06	0.47	19.44	-0.01	2.35	0.25
2003	Abadan	87	45924	0.82	0.73	0.54	1.16	84.25	1.21	3.00	0.42
2003	Andimeshk	88	24959	0.33	0.09	0.17	0.57	76.42	1.26	2.80	0.42
2003	Ahvaz	89	203685	1.01	1.01	0.83	1.21	82.39	1.24	2.95	0.42
2003	Izeh	90	27290	0.25	0.15	0.13	0.43	50.83	1.09	2.99	0.42
2003	Bandar-e-Mahshahr	91	35678	0.69	0.67	0.42	1.05	94.16	1.12	2.95	0.42
2003	Behbahan	92	30754	0.66	0.72	0.4	1.02	71.87	1.27	2.76	0.42
2003	Khorramshahr	93	23855	0.54	0.35	0.3	0.89	83.75	1.22	2.93	0.42
2003	Dezful	94	60375	0.35	0.21	0.21	0.54	73.39	1.21	2.88	0.42
2003	Dashte Azadegan	95	17469	0.49	0.48	0.25	0.84	56.00	1.21	2.89	0.42
2003	Ramhormoz	96	26302	0.46	0.4	0.25	0.75	52.24	1.13	2.98	0.42
2003	Shadegan	97	18130	0.38	0.12	0.2	0.67	38.58	1.18	2.96	0.42
2003	Shushtar	98	35798	0.37	0.18	0.21	0.6	60.03	1.18	2.80	0.42
2003	Masjedsoleyman	99	28417	0.41	0.36	0.22	0.67	66.50	1.18	2.87	0.42
2003	Shush	100	26667	0.58	0.72	0.33	0.93	45.84	1.20	2.91	0.42
2003	Baghmalek	101	13784	0.27	0.15	0.12	0.49	34.29	1.23	2.82	0.42
2003	Omidiyeh	102	13331	0.47	0.31	0.24	0.82	69.24	1.17	2.89	0.42
2003	Lali	103	4970	0.41	NA	0.15	0.88	47.30	1.20	2.81	0.42
2003	Hendijan	104	6048	0.63	NA	0.26	1.3	69.06	1.26	2.78	0.42
2003	Abadeh	105	18810	1.01	1.37	0.59	1.55	88.30	0.86	2.91	0.33
2003	Estahban	106	11677	0.4	0.17	0.19	0.73	71.31	0.88	2.94	0.33
2003	Eqlid	107	16485	0.4	0.25	0.2	0.69	63.64	0.83	3.00	0.33
2003	Jahrom	108	35898	0.47	0.45	0.27	0.74	63.86	0.78	2.96	0.33
2003	Darab	109	27460	0.48	0.52	0.27	0.78	42.28	0.77	2.87	0.33
2003	Sepidan	110	14434	0.28	0.28	0.13	0.51	19.45	0.82	2.79	0.33
2003	Shiraz	111	301141	0.82	0.81	0.68	0.96	87.67	0.79	2.88	0.33
2003	Fasa	112	33738	0.58	0.6	0.35	0.9	57.40	0.78	2.97	0.33
2003	Firuzabad	113	20514	0.61	0.89	0.33	0.98	54.53	0.75	2.92	0.33
2003	Kazerun	114	46729	0.31	0.22	0.17	0.49	51.44	0.77	2.91	0.33
2003	Lar (Larestan)	115	43442	0.59	0.61	0.37	0.88	57.69	0.88	2.86	0.33
2003	Marvdasht	116	51982	0.41	0.44	0.24	0.62	44.93	0.83	2.98	0.33
2003	Mamasany	117	30342	0.3	0.4	0.16	0.5	30.91	0.86	2.89	0.33
2003	Neyriz	118	17681	0.38	0.35	0.2	0.66	58.63	0.83	2.87	0.33
2003	Lamard	119	11790	0.61	1.21	0.3	1.07	42.66	0.89	2.81	0.33
2003	Bovanat	120	8398	0.3	NA	0.12	0.62	29.60	0.81	2.83	0.33
2003	Arsanjan	121	6495	0.58	0.65	0.26	1.11	39.40	0.82	2.79	0.33
2003	Khorrambid	122	7543	0.37	0.27	0.16	0.7	76.86	0.80	2.92	0.33
2003	Zarrindasht	123	8875	0.34	0.23	0.14	0.67	57.15	0.85	2.96	0.33
2003	Qirokarzin	124	9893	0.32	0.42	0.14	0.61	55.96	0.90	2.98	0.33
2003	Mohr	125	7578	0.33	NA	0.13	0.69	35.93	0.74	2.90	0.33
2003	Farashband	126	7065	0.4	NA	0.16	0.81	56.70	0.84	2.93	0.33
2003	Baft	127	24345	0.31	NA	0.14	0.61	35.03	0.23	3.09	0.70
2003	Bam	128	36150	0.27	0.23	0.14	0.45	40.54	0.29	3.05	0.70
2003	Jiroft	129	31669	0.35	0.39	0.19	0.58	39.68	0.24	3.15	0.70
2003	Rafsanjan	130	47057	0.74	0.83	0.48	1.05	55.76	0.24	3.08	0.70

2003	Zarand	131	24865	0.46	NA	0.2	0.9	51.61	0.21	3.14	0.70
2003	Sirjan	132	39133	0.51	0.47	0.31	0.79	74.55	0.22	3.16	0.70
2003	Shahrebabak	133	15716	0.62	1.01	0.32	1.04	62.08	0.24	3.02	0.70
2003	Kerman	134	109629	0.77	0.77	0.57	1	87.22	0.21	3.19	0.70
2003	Kahnuj	135	29492	0.21	0.22	0.11	0.38	20.89	0.25	3.19	0.70
2003	Bardsir	136	13755	0.27	0.15	0.12	0.5	54.35	0.27	3.20	0.70
2003	Ravar	137	7089	0.37	0.29	0.15	0.72	59.08	0.24	3.22	0.70
2003	Anbarabad	138	9629	0.26	NA	0.09	0.58	33.73	0.22	3.16	0.70
2003	Manujan	139	7566	0.22	NA	0.08	0.48	24.76	0.07	3.20	0.70
2003	Taybad	140	19896	0.25	NA	0.11	0.51	44.59	0.15	2.57	0.43
2003	Torbate Heydarieh	141	54572	0.9	1.17	0.62	1.23	44.99	0.19	2.48	0.43
2003	Torbate Jam	142	34053	0.56	0.8	0.33	0.87	42.84	0.14	2.60	0.43
2003	Darrehgaz	143	14462	0.29	0.14	0.14	0.52	54.46	0.22	2.46	0.43
2003	Sabzevar	144	81740	0.82	0.97	0.59	1.08	48.05	0.21	2.52	0.43
2003	Quchan	145	32968	0.52	0.78	0.3	0.81	48.99	0.21	2.64	0.43
2003	Kashmar	146	35324	0.48	0.63	0.27	0.75	47.62	0.16	2.55	0.43
2003	Gonabad	147	21868	0.36	0.27	0.19	0.61	48.10	0.31	2.54	0.43
2003	Mashhad	148	473787	0.88	0.89	0.76	1	90.03	0.21	2.64	0.43
2003	Neyshabur	149	78622	0.79	0.97	0.56	1.06	50.57	0.07	2.51	0.43
2003	Chenaran	150	17939	0.26	0.22	0.12	0.47	38.28	0.13	2.52	0.43
2003	Khaf	151	15201	0.23	0.14	0.11	0.43	44.81	0.13	2.55	0.43
2003	Sarakhs	152	11974	0.48	0.91	0.23	0.87	40.03	0.21	2.64	0.43
2003	Fariman	153	13869	0.3	0.15	0.14	0.54	53.39	0.16	2.52	0.43
2003	Bardeskan	154	12595	0.2	0.16	0.09	0.39	34.77	0.20	2.67	0.43
2003	Rashtkhar	155	8844	0.22	NA	0.08	0.49	18.32	0.12	2.59	0.43
2003	Kalat	156	6852	0.37	NA	0.14	0.81	23.98	0.21	2.57	0.43
2003	Ardestan	157	10002	0.37	0.37	0.16	0.68	54.31	1.37	3.54	0.23
2003	Isfahan	158	367358	0.84	0.83	0.71	0.97	91.37	1.28	3.28	0.23
2003	Khomeynishahr	159	46587	0.45	0.4	0.27	0.7	95.38	1.36	3.47	0.23
2003	Khansar	160	7677	0.36	NA	0.16	0.72	61.45	1.38	3.33	0.23
2003	Semirom	161	12527	0.37	NA	0.15	0.74	51.21	1.37	3.43	0.23
2003	Faridan	162	16451	0.42	0.24	0.21	0.72	40.05	1.40	3.44	0.23
2003	Fereydunshahr	163	7267	0.3	NA	0.12	0.6	46.47	1.40	3.38	0.23
2003	Falavarjan	164	38067	0.47	0.49	0.27	0.73	61.30	1.35	3.26	0.23
2003	Shahreza	165	35027	0.39	0.29	0.22	0.63	78.35	1.42	3.26	0.23
2003	Kashan	166	57116	0.74	0.71	0.49	1.04	84.79	1.38	3.22	0.23
2003	Golpayegan	167	18186	0.45	0.53	0.23	0.76	72.79	1.29	3.42	0.23
2003	Lanjan	168	39539	0.65	0.68	0.4	0.97	81.86	1.29	3.42	0.23
2003	Nayin	169	11956	0.4	NA	0.17	0.78	65.72	1.40	3.27	0.23
2003	Najafabad	170	49341	0.62	0.59	0.39	0.9	91.58	1.27	3.11	0.23
2003	Natanz	171	9049	0.34	0.21	0.16	0.63	55.92	1.35	3.30	0.23
2003	Shahinshahr va Meyme	172	49812	0.84	0.9	0.56	1.18	89.14	1.35	3.25	0.23
2003	Mobarakeh	173	23254	0.46	0.54	0.25	0.77	81.22	1.37	3.34	0.23
2003	Aran va Bidgol	174	16490	0.53	0.63	0.28	0.89	85.43	1.33	3.27	0.23
2003	Tiran va Karvan	175	12469	0.25	0.16	0.11	0.47	37.13	1.37	3.36	0.23
2003	Chadegan	176	6408	0.27	NA	0.1	0.57	39.14	1.35	3.32	0.23
2003	Iranshahr	177	29432	0.31	0.36	0.16	0.52	42.43	-1.66	1.82	0.59

2003	Chah Bahar	178	26500	0.17	0.08	0.08	0.31	31.53	-1.63	1.47	0.59
2003	Khash	179	17274	0.19	NA	0.08	0.4	29.90	-1.57	1.64	0.59
2003	Zabol	180	53027	0.15	0.04	0.08	0.26	36.74	-1.59	1.73	0.59
2003	Zahedan	181	72196	0.45	0.46	0.28	0.66	87.72	-1.56	1.62	0.59
2003	Saravan	182	31011	0.16	0.27	0.07	0.29	35.71	-1.61	1.65	0.59
2003	Nikshahr	183	23226	0.14	NA	0.06	0.29	21.40	-1.51	1.57	0.59
2003	Sarbaz	184	15279	0.17	NA	0.05	0.44	11.37	-1.58	1.89	0.59
2003	Baneh	185	18168	0.24	0.11	0.11	0.42	59.99	-0.08	1.87	0.42
2003	Bijar	186	20007	0.22	0.2	0.1	0.39	51.36	-0.08	1.83	0.42
2003	Saqqez	187	36087	0.22	0.11	0.11	0.37	63.49	-0.12	1.68	0.42
2003	Sanandaj	188	71685	0.38	0.29	0.24	0.55	80.88	-0.14	1.82	0.42
2003	Qorveh	189	34865	0.29	0.35	0.16	0.49	50.37	-0.08	1.98	0.42
2003	Marivan	190	21710	0.33	0.3	0.17	0.57	59.60	-0.09	1.83	0.42
2003	Divandarreh	191	13465	0.21	NA	0.09	0.43	25.27	-0.16	1.70	0.42
2003	Kamyaran	192	17182	0.22	0.24	0.1	0.41	41.93	-0.15	1.65	0.42
2003	Sarvabad	193	9618	0.17	NA	0.06	0.35	4.69	-0.16	1.79	0.42
2003	Tuyserkan	194	22897	0.23	0.08	0.11	0.4	43.43	0.40	2.31	0.19
2003	Malayer	195	54520	0.27	0.22	0.16	0.43	56.07	0.39	2.45	0.19
2003	Nahavand	196	33657	0.24	0.24	0.13	0.41	48.61	0.37	2.38	0.19
2003	Hamadan	197	116460	0.41	0.39	0.28	0.56	81.08	0.38	2.45	0.19
2003	Kabudarahang	198	23389	0.22	0.35	0.11	0.39	16.27	0.39	2.41	0.19
2003	Asadabad	199	18697	0.26	0.22	0.13	0.46	46.61	0.41	2.28	0.19
2003	Bahar	200	22014	0.33	0.45	0.17	0.57	41.87	0.39	2.23	0.19
2003	Razan	201	21055	0.23	NA	0.1	0.46	17.89	0.34	2.54	0.19
2003	Borujen	202	20510	0.27	0.2	0.13	0.49	80.60	0.06	2.19	0.22
2003	Shahrekord	203	67801	0.3	0.21	0.18	0.47	71.85	0.01	2.22	0.22
2003	Farsan	204	13267	0.26	0.16	0.12	0.47	62.59	-0.03	2.19	0.22
2003	Lordakan	205	22689	0.25	0.37	0.12	0.44	18.71	0.06	2.20	0.22
2003	Ardal	206	7693	0.2	NA	0.07	0.45	14.83	0.08	2.18	0.22
2003	Kuhrang	207	4558	0.18	NA	0.07	0.38	6.33	0.02	2.24	0.22
2003	Aligudarz	208	21633	0.22	0.09	0.11	0.39	57.23	0.16	2.26	0.25
2003	Borujerd	209	60705	0.48	0.43	0.3	0.7	75.29	0.15	2.52	0.25
2003	Khorramabad	210	82321	0.42	0.36	0.27	0.61	66.53	0.15	2.32	0.25
2003	Dalfan	211	18602	0.22	NA	0.09	0.44	40.74	0.21	2.48	0.25
2003	Dorud	212	25457	0.3	0.25	0.16	0.51	65.51	0.21	2.33	0.25
2003	Kuhdasht	213	29288	0.17	0.07	0.08	0.31	49.34	0.16	2.30	0.25
2003	Azna	214	12907	0.28	NA	0.12	0.56	51.97	0.29	2.51	0.25
2003	Poldokhtar	215	11504	0.31	NA	0.13	0.63	36.25	0.23	2.42	0.25
2003	Selseleh	216	11091	0.3	NA	0.1	0.72	39.03	0.23	2.32	0.25
2003	Ilam	217	30232	0.45	0.35	0.25	0.72	81.00	0.49	2.26	0.31
2003	Darrehshahr	218	8943	0.2	NA	0.07	0.43	36.47	0.45	2.15	0.31
2003	Dehloran	219	8790	0.32	NA	0.12	0.67	63.10	0.56	2.27	0.31
2003	Shirvan va Chardavol	220	11758	0.21	NA	0.08	0.44	19.77	0.44	2.18	0.31
2003	Mehran	221	7996	0.4	NA	0.16	0.82	57.24	0.42	2.19	0.31
2003	Abdanan	222	7215	0.26	NA	0.11	0.53	57.96	0.39	2.29	0.31
2003	Eyvan	223	8222	0.44	0.25	0.2	0.82	60.96	0.53	2.26	0.31
2003	Boyerahmad	224	27142	0.33	0.33	0.17	0.57	40.29	-0.15	2.29	0.29

2003	Kohgiluyeh	225	31844	0.19	NA	0.08	0.38	35.96	-0.12	2.23	0.29
2003	Gachsaran	226	22257	0.32	0.19	0.16	0.56	61.09	-0.14	2.42	0.29
2003	Dena	227	8359	0.23	NA	0.09	0.47	19.41	-0.10	2.32	0.29
2003	Bushehr	228	33546	1.06	1.04	0.69	1.54	85.45	1.14	2.65	0.75
2003	Tangestan	229	10873	0.39	0.55	0.18	0.71	22.75	1.05	2.66	0.75
2003	Dashtestan	230	38162	0.54	0.69	0.32	0.83	61.05	1.05	2.75	0.75
2003	Dashti	231	12504	0.35	0.32	0.17	0.64	52.92	1.07	2.68	0.75
2003	Dayyer	232	7256	0.37	0.29	0.16	0.69	68.18	1.00	2.74	0.75
2003	Kangan	233	8566	0.43	NA	0.18	0.87	68.32	1.06	2.65	0.75
2003	Genaveh	234	14094	0.57	0.44	0.29	0.97	73.31	1.05	2.71	0.75
2003	Deylam	235	4881	0.54	NA	0.21	1.15	72.13	1.06	2.59	0.75
2003	Jam	236	4767	0.27	NA	0.1	0.59	34.33	1.06	2.92	0.75
2003	Abhar	237	29404	0.22	0.14	0.11	0.39	61.00	0.30	2.09	0.29
2003	Khodabandeh	238	26634	0.16	0.08	0.07	0.29	24.53	0.28	2.05	0.29
2003	Zanjan	239	79044	0.44	0.44	0.29	0.63	76.04	0.34	2.20	0.29
2003	Ijrud	240	6939	0.19	NA	0.07	0.41	6.46	0.30	2.05	0.29
2003	Khorramdarreh	241	10665	0.37	0.39	0.17	0.67	78.13	0.26	1.98	0.29
2003	Tarom	242	7741	0.2	NA	0.07	0.46	12.51	0.22	2.08	0.29
2003	Mahneshan	243	7261	0.2	NA	0.07	0.47	12.25	0.30	2.12	0.29
2003	Damghan	244	16517	0.39	0.12	0.2	0.68	65.05	2.22	4.27	0.33
2003	Semnan	245	33240	0.34	0.19	0.19	0.57	88.05	2.18	4.32	0.33
2003	Shahrud	246	43446	0.7	0.73	0.45	1.02	61.47	2.06	4.22	0.33
2003	Garmsar	247	15380	0.61	0.91	0.33	1.02	64.16	2.15	4.31	0.33
2003	Ardakan	248	11695	0.53	0.68	0.27	0.92	79.99	1.49	3.21	0.37
2003	Bafq	249	8490	0.54	0.72	0.26	0.96	64.99	1.36	3.26	0.37
2003	Taft	250	11196	0.37	NA	0.16	0.73	30.00	1.44	3.14	0.37
2003	Mehriz	251	8491	0.78	1.37	0.4	1.37	52.75	1.27	3.14	0.37
2003	Yazd	252	82573	1.15	1.16	0.86	1.48	95.48	1.41	3.13	0.37
2003	Meybod	253	11725	0.61	0.69	0.31	1.04	90.17	1.44	3.25	0.37
2003	Abarkuh	254	7977	0.66	1.24	0.32	1.17	62.32	1.39	3.25	0.37
2003	Sadugh	255	5214	0.52	NA	0.19	1.16	51.70	1.49	3.12	0.37
2003	Khatam	256	5348	0.41	NA	0.16	0.86	54.31	1.49	3.20	0.37
2003	Tabas	257	10996	0.41	0.37	0.19	0.74	52.75	1.44	3.21	0.37
2003	Abumusa	258	142	0.43	NA	0.16	0.95	60.00	0.19	2.08	0.38
2003	Bandarabbas	259	76208	0.48	0.46	0.31	0.69	67.25	0.27	2.11	0.38
2003	Bandar-e Lengeh	260	20177	0.28	0.21	0.15	0.47	46.29	0.14	2.16	0.38
2003	Qeshm	261	12853	0.3	0.17	0.14	0.55	38.47	0.18	2.10	0.38
2003	Minab	262	35301	0.24	0.23	0.13	0.41	23.51	0.21	2.15	0.38
2003	Bandar-e-Jask	263	9560	0.21	0.21	0.1	0.38	15.82	0.27	1.96	0.38
2003	Rudan	264	14733	0.19	NA	0.08	0.39	32.52	0.18	2.08	0.38
2003	Hajiabad	265	10601	0.21	NA	0.08	0.44	29.75	0.20	2.13	0.38
2003	Bastak	266	9749	0.29	NA	0.12	0.6	23.93	0.15	2.27	0.38
2003	Tehran	267	1660123	1.72	1.73	1.63	1.81	99.62	1.95	3.78	0.32
2003	Damavand	268	16400	0.42	0.12	0.21	0.73	68.89	1.87	3.71	0.32
2003	Rey	269	39627	1.35	1.62	0.93	1.87	28.67	1.92	3.80	0.32
2003	Shemiranat	270	7344	1.33	2.14	0.7	2.24	54.51	1.97	3.70	0.32
2003	Varamin	271	83318	0.36	0.31	0.23	0.53	78.20	1.85	3.76	0.32

2003	Shahriyar	272	136131	0.28	0.27	0.18	0.4	81.99	1.83	3.76	0.32
2003	Eslamshahr	273	71834	0.26	0.21	0.15	0.41	88.33	1.90	3.80	0.32
2003	Robatkarim	274	74755	0.17	0.13	0.09	0.28	81.04	1.94	3.72	0.32
2003	Pakdasht	275	31646	0.19	0.07	0.09	0.35	70.97	1.84	3.66	0.32
2003	Firuzkuh	276	7631	0.28	NA	0.11	0.6	37.66	1.84	3.89	0.32
2003	Ardebil	277	90786	0.31	0.3	0.19	0.45	81.13	-0.06	1.82	0.24
2003	Bilehsowar	278	9425	0.22	NA	0.09	0.47	35.04	-0.02	2.03	0.24
2003	Khalkhal	279	18477	0.34	NA	0.12	0.77	42.45	0.00	1.91	0.24
2003	Meshginshahr	280	28209	0.41	0.5	0.23	0.67	38.87	0.02	1.72	0.24
2003	Germi	281	16119	0.25	0.37	0.11	0.47	28.35	-0.01	1.96	0.24
2003	Parsabad	282	24194	0.19	0.18	0.09	0.35	50.47	-0.02	1.68	0.24
2003	Kowsar	283	5482	0.22	NA	0.06	0.54	23.35	0.03	1.91	0.24
2003	Namin	284	10923	0.22	0.18	0.1	0.43	32.61	-0.01	1.88	0.24
2003	Neer	285	4898	0.21	NA	0.07	0.48	22.19	-0.01	2.01	0.24
2003	Qom	286	167430	0.52	0.53	0.39	0.67	92.10	2.52	3.00	0.32
2003	Bueenzahra	287	26830	0.22	0.37	0.11	0.39	32.55	0.90	2.63	0.33
2003	Takestan	288	28708	0.52	0.78	0.3	0.82	54.69	0.86	2.85	0.33
2003	Qazvin	289	122793	0.56	0.56	0.4	0.75	77.41	0.91	2.73	0.33
2003	Abyek	290	14598	0.33	0.43	0.15	0.6	45.95	0.87	2.60	0.33
2003	Bandare Gaz	291	9733	0.74	1.63	0.38	1.28	52.48	0.66	2.54	0.43
2003	Torkman	292	21495	0.43	0.57	0.23	0.72	59.66	0.65	2.49	0.43
2003	Aliabad	293	21360	0.39	0.39	0.2	0.67	46.12	0.65	2.46	0.43
2003	Kordkuy	294	13794	0.46	0.58	0.23	0.8	47.34	0.67	2.41	0.43
2003	Gorgan	295	70473	0.54	0.47	0.35	0.77	68.07	0.61	2.54	0.43
2003	Gonbade Kavus	296	47350	0.36	0.31	0.2	0.56	49.06	0.68	2.55	0.43
2003	Minudasht	297	21441	0.22	0.1	0.11	0.4	32.01	0.58	2.47	0.43
2003	Aqqala	298	17385	0.21	0.24	0.09	0.39	28.93	0.69	2.61	0.43
2003	Kalaleh	299	22019	0.2	0.19	0.1	0.37	22.49	0.70	2.57	0.43
2003	Azadshahr	300	14726	0.32	0.28	0.15	0.57	53.13	0.61	2.54	0.43
2003	Ramyan	301	13284	0.19	NA	0.08	0.39	38.98	0.59	2.40	0.43
2003	Esfarayen	302	22023	0.2	NA	0.08	0.39	36.14	-0.36	2.41	0.38
2003	Bojnurd	303	50657	0.26	0.16	0.14	0.41	56.09	-0.26	2.64	0.38
2003	Jajarm	304	10036	0.2	0.2	0.08	0.41	59.71	-0.33	2.51	0.38
2003	Shirvan	305	27341	0.25	NA	0.11	0.51	48.62	-0.27	2.39	0.38
2003	Faruj	306	10180	0.18	NA	0.07	0.38	23.68	-0.37	2.68	0.38
2003	Maneh va Semelqan	307	14453	0.23	NA	0.09	0.5	22.64	-0.26	2.61	0.38
2003	Birjand	308	47381	0.45	0.42	0.27	0.68	53.75	-1.48	2.27	0.25
2003	Sarbisheh	309	8941	0.28	NA	0.08	0.71	22.43	-1.48	2.44	0.25
2003	Qaenat	310	24606	0.21	0.16	0.1	0.38	31.07	0.21	2.49	0.25
2003	Nehbandan	311	8528	0.2	NA	0.06	0.48	23.70	-1.39	2.16	0.25
2003	Sarayan	312	6518	0.33	NA	0.12	0.72	57.49	-1.33	2.36	0.25
2003	Ferdows	313	12801	0.35	0.46	0.16	0.63	64.78	0.22	2.56	0.25
2003	Karaj	314	288912	0.32	0.31	0.24	0.41	96.26	1.87	3.94	0.56
2003	Savojbolagh	315	33904	0.08	NA	0.03	0.19	34.68	1.85	3.77	0.56
2003	Nazarabad	316	19946	0.27	NA	0.11	0.55	77.00	1.89	3.74	0.56
2004	Arak	1	114779	0.6	0.61	0.43	0.79	78.10	1.09	3.27	0.32
2004	Ashtiyan	2	4343	0.45	0.43	0.18	0.91	31.61	1.07	3.08	0.32

2004	Tafresh	3	13527	0.38	0.55	0.18	0.67	25.30	1.03	3.22	0.32
2004	Khomeyn	4	23995	0.25	0.08	0.13	0.42	50.59	1.05	3.10	0.32
2004	Delijan	5	8904	0.54	NA	0.23	1.07	65.22	1.09	3.14	0.32
2004	Saveh	6	39739	0.29	0.21	0.16	0.48	66.20	1.08	2.97	0.32
2004	Shazand	7	24913	0.23	0.08	0.11	0.41	28.25	1.05	2.97	0.32
2004	Mahalat	8	10321	0.45	0.38	0.21	0.8	78.53	1.10	3.19	0.32
2004	Zarandiyeh	9	11460	0.26	0.17	0.12	0.51	54.50	1.15	3.20	0.32
2004	Komijan	10	7688	0.31	NA	0.11	0.67	38.21	0.98	3.11	0.32
2004	Astara	11	14702	0.33	0.28	0.15	0.59	59.69	0.50	3.58	0.24
2004	Astanehye Ashrafiyeh	12	26359	0.44	0.44	0.24	0.73	42.52	0.48	3.74	0.24
2004	Bandar Anzali	13	31267	0.76	0.8	0.46	1.14	81.91	0.50	3.42	0.24
2004	Tavalesh	14	32758	0.3	0.31	0.16	0.5	30.87	0.53	3.55	0.24
2004	Rasht	15	186335	0.65	0.64	0.5	0.81	67.01	0.45	3.64	0.24
2004	Rudbar	16	21858	0.34	0.18	0.18	0.58	48.84	0.49	3.45	0.24
2004	Rudsar	17	36084	0.72	0.8	0.45	1.08	42.84	0.55	3.70	0.24
2004	Sumehsara	18	30037	0.47	0.52	0.27	0.76	28.74	0.50	3.65	0.24
2004	Fuman	19	23082	0.36	0.34	0.19	0.61	25.22	0.49	3.62	0.24
2004	Langrud	20	31965	0.49	0.43	0.28	0.76	58.93	0.49	3.71	0.24
2004	Lahijan	21	39190	0.63	0.64	0.39	0.93	41.79	0.51	3.60	0.24
2004	Shaft	22	14912	0.3	0.26	0.15	0.55	11.81	0.47	3.68	0.24
2004	Amlash	23	11067	0.44	0.7	0.2	0.8	30.65	0.45	3.51	0.24
2004	Rezvanshahr	24	13109	0.36	0.46	0.17	0.67	27.41	0.45	3.55	0.24
2004	Siyahkal	25	11312	0.41	0.34	0.2	0.73	29.88	0.56	3.64	0.24
2004	Masal	26	10052	0.34	0.39	0.16	0.64	27.77	0.46	3.52	0.24
2004	Amol	27	70904	0.66	0.7	0.44	0.92	53.95	1.85	3.74	0.36
2004	Babol	28	98526	1.1	1.17	0.84	1.39	45.57	1.76	3.77	0.36
2004	Behshahr	29	39821	0.56	0.6	0.34	0.85	60.14	1.75	3.74	0.36
2004	Tonekabon	30	42850	0.51	0.56	0.31	0.78	46.53	1.76	3.62	0.36
2004	Ramsar	31	15813	0.55	0.37	0.29	0.92	70.68	1.75	3.75	0.36
2004	Sari	32	100184	0.61	0.61	0.44	0.82	49.70	1.82	3.83	0.36
2004	Savadkuh	33	15266	0.28	0.13	0.13	0.51	46.22	1.85	3.87	0.36
2004	Qaemshahr	34	62045	0.56	0.58	0.36	0.8	57.79	1.75	3.75	0.36
2004	Nur	35	21832	0.3	0.28	0.15	0.52	37.92	1.79	3.74	0.36
2004	Noshahr	36	23859	0.19	0.08	0.09	0.35	34.33	1.78	3.80	0.36
2004	Babolsar	37	35594	0.46	0.46	0.27	0.73	50.64	1.78	3.81	0.36
2004	Mahmudabad	38	18565	0.41	0.44	0.21	0.72	31.02	1.78	3.84	0.36
2004	Neka	39	20465	0.37	0.39	0.19	0.65	34.79	1.84	3.76	0.36
2004	Chalus	40	25024	0.24	0.08	0.12	0.43	51.63	1.76	3.80	0.36
2004	Juybar	41	14578	0.41	0.41	0.2	0.73	38.60	1.80	3.61	0.36
2004	Ahar	42	27973	0.21	0.07	0.1	0.37	61.39	0.42	2.51	0.34
2004	Tabriz	43	313798	0.52	0.51	0.42	0.64	93.24	0.52	2.44	0.34
2004	Sarab	44	25550	0.28	0.16	0.15	0.49	41.00	0.42	2.30	0.34
2004	Maragheh	45	45038	0.26	0.18	0.14	0.43	68.23	0.40	2.48	0.34
2004	Marand	46	47046	0.5	0.47	0.3	0.76	57.47	0.31	2.50	0.34
2004	Miyaneh	47	37922	0.25	0.15	0.13	0.42	47.94	0.47	2.41	0.34
2004	Hashtrud	48	11935	0.27	0.17	0.12	0.51	27.34	0.44	2.38	0.34
2004	Bonab	49	23705	0.29	0.09	0.15	0.51	60.52	0.45	2.57	0.34

2004	Bostanabad	50	16871	0.25	0.12	0.12	0.46	18.28	0.38	2.44	0.34
2004	Shabestar	51	27809	0.25	0.14	0.13	0.44	46.51	0.43	2.40	0.34
2004	Kalibar	52	15795	0.19	NA	0.08	0.4	13.57	0.46	2.29	0.34
2004	Haris	53	12615	0.37	NA	0.16	0.76	44.77	0.38	2.32	0.34
2004	Jolfa	54	11506	0.3	NA	0.12	0.64	59.74	0.36	2.35	0.34
2004	Malekan	55	18135	0.25	NA	0.1	0.5	31.54	0.35	2.49	0.34
2004	Azarshahr	56	20633	0.24	0.1	0.12	0.44	59.50	0.42	2.45	0.34
2004	Osku	57	16797	0.22	0.12	0.1	0.4	50.64	0.44	2.28	0.34
2004	Charoymaq	58	5505	0.19	NA	0.07	0.39	11.89	0.40	2.39	0.34
2004	Varzaqan	59	8841	0.22	NA	0.09	0.45	10.10	0.37	2.48	0.34
2004	Ajabshir	60	12867	0.31	NA	0.13	0.63	41.53	0.43	2.39	0.34
2004	Orumiyeh	61	159321	0.83	0.86	0.64	1.03	69.44	0.65	2.26	0.35
2004	Piranshahr	62	15779	0.33	0.54	0.16	0.6	48.82	0.70	1.99	0.35
2004	Khoy	63	65957	0.24	0.21	0.14	0.38	65.15	0.60	2.15	0.35
2004	Sardasht	64	16136	0.19	NA	0.08	0.4	45.45	0.64	2.41	0.35
2004	Salmas	65	30150	0.61	0.81	0.36	0.95	54.09	0.68	2.18	0.35
2004	Maku	66	28825	0.18	0.14	0.09	0.34	48.32	0.68	2.21	0.35
2004	Mahabad	67	34902	0.25	0.23	0.14	0.43	68.51	0.61	2.08	0.35
2004	Miyandoab	68	44026	0.35	0.42	0.2	0.56	50.84	0.62	2.23	0.35
2004	Naqadeh	69	21826	0.49	0.66	0.26	0.81	67.07	0.64	2.34	0.35
2004	Bukan	70	34585	0.23	0.18	0.12	0.39	72.12	0.57	2.34	0.35
2004	Shahindezh	71	16797	0.26	0.24	0.12	0.49	48.97	0.60	2.08	0.35
2004	Takab	72	15045	0.23	0.26	0.1	0.43	54.27	0.56	2.28	0.35
2004	Oshnaviyeh	73	10020	0.74	1.44	0.37	1.29	53.71	0.67	2.16	0.35
2004	Chaldoran	74	7004	0.25	0.29	0.1	0.53	34.38	0.61	2.26	0.35
2004	Eslamabade Gharb	75	36774	0.33	0.5	0.18	0.53	51.07	0.31	2.58	0.30
2004	Kermanshah	76	179512	0.76	0.76	0.6	0.94	84.14	0.27	2.38	0.30
2004	Paveh	77	10331	0.3	0.19	0.14	0.57	49.98	0.22	2.43	0.30
2004	Sarpole Zahab	78	14053	0.49	1.03	0.24	0.87	40.14	0.22	2.44	0.30
2004	Sonqor	79	19550	0.27	0.1	0.14	0.49	46.63	0.28	2.30	0.30
2004	Qasreshirin	80	4026	0.49	NA	0.2	1.03	68.64	0.19	2.29	0.30
2004	Kangavar	81	15488	0.39	0.39	0.19	0.68	60.91	0.16	2.37	0.30
2004	Gilanegharb	82	12017	0.34	0.85	0.15	0.64	32.99	0.21	2.36	0.30
2004	Javanrud	83	17288	0.26	0.24	0.12	0.47	53.00	0.19	2.43	0.30
2004	Sahneh	84	15333	0.32	0.26	0.15	0.57	41.54	0.25	2.43	0.30
2004	Harsin	85	15757	0.32	0.26	0.15	0.58	64.59	0.24	2.48	0.30
2004	Salas-e-Babajani	86	5168	0.21	NA	0.07	0.5	20.27	0.19	2.41	0.30
2004	Abadan	87	47517	0.96	0.92	0.65	1.33	84.15	1.33	2.86	0.47
2004	Andimeshk	88	25906	0.38	0.16	0.2	0.63	76.91	1.26	2.99	0.47
2004	Ahvaz	89	211856	0.88	0.86	0.71	1.06	82.50	1.37	3.00	0.47
2004	Izeh	90	28365	0.26	0.15	0.13	0.45	51.82	1.24	2.99	0.47
2004	Bandar-e-Mahshahr	91	37284	0.66	0.58	0.41	1	94.16	1.31	3.04	0.47
2004	Behbahan	92	31726	0.54	0.45	0.31	0.84	71.91	1.33	2.79	0.47
2004	Khorramshahr	93	24936	0.71	0.67	0.41	1.1	83.68	1.35	2.93	0.47
2004	Dezful	94	62709	0.58	0.57	0.38	0.84	73.56	1.30	3.06	0.47
2004	Dashte Azadegan	95	17916	0.51	0.47	0.27	0.86	56.27	1.29	3.13	0.47
2004	Ramhormoz	96	27217	0.72	0.92	0.42	1.12	52.62	1.30	3.12	0.47

2004	Shadegan	97	18764	0.47	0.34	0.24	0.8	38.81	1.36	2.95	0.47
2004	Shushtar	98	37277	0.7	0.79	0.43	1.05	60.36	1.38	2.94	0.47
2004	Masjedsoleyman	99	28770	0.43	0.36	0.23	0.69	66.69	1.33	2.96	0.47
2004	Shush	100	27694	0.46	0.38	0.25	0.75	45.96	1.34	3.04	0.47
2004	Baghmalek	101	14323	0.32	NA	0.14	0.65	34.83	1.36	2.87	0.47
2004	Omidiyeh	102	13769	0.53	0.46	0.27	0.91	69.17	1.31	3.02	0.47
2004	Lali	103	5128	0.43	0.4	0.18	0.88	47.20	1.31	3.04	0.47
2004	Hendijan	104	6229	0.7	NA	0.29	1.45	69.43	1.32	3.12	0.47
2004	Abadeh	105	19286	0.68	0.62	0.39	1.11	88.41	0.98	3.01	0.36
2004	Estahban	106	12005	0.46	0.33	0.22	0.82	71.16	1.03	3.10	0.36
2004	Eqlid	107	17297	0.56	0.71	0.29	0.92	63.41	0.98	2.85	0.36
2004	Jahrom	108	36915	0.76	0.92	0.48	1.12	63.96	1.03	3.09	0.36
2004	Darab	109	28524	0.49	0.5	0.28	0.78	42.29	0.94	3.02	0.36
2004	Sepidan	110	15008	0.3	0.27	0.14	0.54	20.06	0.93	3.01	0.36
2004	Shiraz	111	313786	0.96	0.96	0.81	1.12	87.75	0.96	3.13	0.36
2004	Fasa	112	34819	0.56	0.52	0.33	0.87	57.66	1.04	2.93	0.36
2004	Firuzabad	113	21043	0.38	0.19	0.2	0.65	55.37	0.95	3.10	0.36
2004	Kazerun	114	48242	0.4	0.38	0.24	0.61	51.43	0.96	2.99	0.36
2004	Lar (Larestan)	115	44940	0.52	0.46	0.31	0.78	57.89	0.92	2.95	0.36
2004	Marvdasht	116	54087	0.29	0.19	0.17	0.47	45.20	0.93	2.96	0.36
2004	Mamasany	117	31413	0.25	0.19	0.13	0.42	31.72	1.05	2.94	0.36
2004	Neyriz	118	18380	0.4	0.33	0.21	0.68	58.75	0.98	3.07	0.36
2004	Lamard	119	12242	0.47	0.5	0.23	0.83	42.96	0.97	3.16	0.36
2004	Bovanat	120	8667	0.34	0.46	0.15	0.66	29.79	0.91	3.01	0.36
2004	Arsanjan	121	6781	0.62	0.62	0.28	1.14	39.46	1.02	3.00	0.36
2004	Khorrambid	122	7856	0.42	NA	0.17	0.87	77.11	0.97	2.99	0.36
2004	Zarrindasht	123	9216	0.38	NA	0.15	0.83	56.93	1.00	2.92	0.36
2004	Qirokarzin	124	10385	0.3	0.2	0.13	0.57	55.67	1.01	2.94	0.36
2004	Mohr	125	7854	0.36	NA	0.14	0.75	36.05	1.02	3.01	0.36
2004	Farashband	126	7514	0.42	NA	0.17	0.86	55.73	1.03	2.94	0.36
2004	Baft	127	25214	0.3	0.23	0.16	0.52	35.36	0.38	3.45	0.67
2004	Bam	128	37806	0.32	0.33	0.17	0.52	39.57	0.38	3.38	0.67
2004	Jiroft	129	33390	0.31	0.25	0.17	0.52	40.24	0.40	3.39	0.67
2004	Rafsanjan	130	49052	0.65	0.67	0.41	0.94	56.33	0.45	3.20	0.67
2004	Zarand	131	25627	0.48	0.48	0.26	0.79	52.20	0.39	3.31	0.67
2004	Sirjan	132	40772	0.6	0.6	0.36	0.9	74.82	0.33	3.20	0.67
2004	Shahrebabak	133	16708	0.44	0.35	0.23	0.77	62.69	0.37	3.40	0.67
2004	Kerman	134	114351	0.9	0.92	0.69	1.15	87.44	0.34	3.35	0.67
2004	Kahnuj	135	31913	0.24	0.27	0.12	0.41	21.28	0.35	3.25	0.67
2004	Bardsir	136	14375	0.3	0.28	0.14	0.56	54.63	0.35	3.35	0.67
2004	Ravar	137	7336	0.41	NA	0.16	0.87	58.74	0.39	3.23	0.67
2004	Anbarabad	138	10297	0.27	NA	0.09	0.6	33.35	0.40	3.13	0.67
2004	Manujan	139	8003	0.24	NA	0.08	0.52	26.67	0.38	3.37	0.67
2004	Taybad	140	20616	0.3	0.4	0.14	0.53	45.05	0.48	2.58	0.46
2004	Torbate Heydarieh	141	56244	0.55	0.64	0.35	0.8	45.61	0.34	2.75	0.46
2004	Torbate Jam	142	35337	0.4	0.41	0.22	0.64	43.28	0.33	2.69	0.46
2004	Darrehgaz	143	14820	0.32	0.13	0.15	0.57	54.75	0.41	2.74	0.46

2004	Sabzevar	144	84016	0.81	0.94	0.58	1.08	48.68	0.40	2.71	0.46
2004	Quchan	145	33843	0.34	0.35	0.18	0.56	49.54	0.38	2.59	0.46
2004	Kashmar	146	36411	0.55	0.72	0.33	0.84	48.20	0.34	2.75	0.46
2004	Gonabad	147	22405	0.57	0.79	0.32	0.92	48.55	0.37	2.63	0.46
2004	Mashhad	148	495998	0.97	0.98	0.85	1.1	90.18	0.37	2.71	0.46
2004	Neyshabur	149	81124	0.58	0.66	0.39	0.79	51.24	0.45	2.49	0.46
2004	Chenaran	150	18627	0.3	0.32	0.14	0.53	38.99	0.32	2.65	0.46
2004	Khaf	151	15784	0.25	0.13	0.12	0.46	44.93	0.46	2.76	0.46
2004	Sarakhs	152	12456	0.34	0.17	0.16	0.64	40.28	0.38	2.49	0.46
2004	Fariman	153	14386	0.44	0.71	0.22	0.79	53.56	0.30	2.52	0.46
2004	Bardeskan	154	12988	0.23	0.31	0.1	0.44	35.46	0.38	2.56	0.46
2004	Rashtkhar	155	9125	0.23	0.22	0.09	0.47	18.55	0.34	2.62	0.46
2004	Kalat	156	7037	0.39	NA	0.15	0.87	24.05	0.39	2.61	0.46
2004	Ardestan	157	10160	0.34	0.18	0.16	0.63	54.72	1.49	3.25	0.27
2004	Isfahan	158	383372	1.04	1.04	0.91	1.19	91.46	1.46	3.41	0.27
2004	Khomeynishahr	159	48744	0.58	0.6	0.36	0.86	95.97	1.53	3.35	0.27
2004	Khansar	160	7756	0.36	0.24	0.17	0.67	61.75	1.60	3.41	0.27
2004	Semirom	161	13039	0.4	0.47	0.19	0.73	51.06	1.53	3.36	0.27
2004	Faridan	162	16745	0.55	0.58	0.29	0.93	40.60	1.56	3.46	0.27
2004	Fereydunshahr	163	7411	0.32	NA	0.13	0.66	46.97	1.45	3.36	0.27
2004	Falavarjan	164	39584	0.42	0.37	0.24	0.67	61.42	1.54	3.38	0.27
2004	Shahreza	165	36154	0.41	0.28	0.23	0.65	78.82	1.60	3.50	0.27
2004	Kashan	166	59256	0.69	0.61	0.46	0.97	85.11	1.53	3.46	0.27
2004	Golpayegan	167	18608	0.43	0.41	0.23	0.73	73.12	1.58	3.46	0.27
2004	Lanjan	168	41060	0.56	0.51	0.34	0.86	82.26	1.47	3.23	0.27
2004	Nayin	169	12196	0.44	0.47	0.21	0.78	66.27	1.59	3.43	0.27
2004	Najafabad	170	51334	0.9	0.96	0.61	1.24	91.64	1.53	3.24	0.27
2004	Natanz	171	9368	0.39	0.41	0.19	0.72	55.70	1.55	3.35	0.27
2004	Shahinshahr va Meyme	172	52214	0.69	0.66	0.45	0.99	89.45	1.54	3.33	0.27
2004	Mobarakeh	173	24196	0.44	0.43	0.24	0.73	81.42	1.58	3.42	0.27
2004	Aran va Bidgol	174	17130	0.64	0.84	0.35	1.05	85.48	1.58	3.50	0.27
2004	Tiran va Karvan	175	12826	0.29	NA	0.12	0.6	37.44	1.57	3.38	0.27
2004	Chadegan	176	6512	0.3	NA	0.12	0.63	39.37	1.59	3.46	0.27
2004	Iranshahr	177	30941	0.33	0.42	0.18	0.56	42.58	-1.40	1.82	0.52
2004	Chah Bahar	178	28361	0.21	NA	0.09	0.42	31.95	-1.28	1.72	0.52
2004	Khash	179	18019	0.2	NA	0.08	0.42	30.59	-1.32	1.64	0.52
2004	Zabol	180	54575	0.18	0.11	0.09	0.3	37.26	-1.32	1.63	0.52
2004	Zahedan	181	75767	0.32	0.23	0.19	0.48	87.58	-1.37	1.67	0.52
2004	Saravan	182	32428	0.13	0.06	0.06	0.23	35.88	-1.37	1.83	0.52
2004	Nikshahr	183	24187	0.15	NA	0.06	0.31	21.51	-1.35	1.76	0.52
2004	Sarbaz	184	16176	0.18	NA	0.05	0.45	11.24	-1.32	1.75	0.52
2004	Baneh	185	18864	0.3	0.33	0.14	0.53	60.64	0.02	1.80	0.48
2004	Bijar	186	20380	0.21	0.1	0.1	0.4	52.09	0.03	1.99	0.48
2004	Saqqez	187	37301	0.24	0.16	0.13	0.41	63.97	0.19	1.77	0.48
2004	Sanandaj	188	74736	0.58	0.58	0.39	0.81	81.21	0.09	1.76	0.48
2004	Qorveh	189	36086	0.24	0.17	0.13	0.41	50.97	0.09	1.80	0.48
2004	Marivan	190	22988	0.37	0.37	0.19	0.64	60.61	0.12	1.85	0.48

2004	Divandarreh	191	13889	0.23	NA	0.1	0.48	25.86	0.14	1.80	0.48
2004	Kamyaran	192	17819	0.23	0.23	0.11	0.44	42.43	0.12	1.91	0.48
2004	Sarvabad	193	9814	0.18	NA	0.07	0.38	4.85	0.19	1.90	0.48
2004	Tuyserkan	194	23311	0.31	NA	0.14	0.61	43.93	0.60	2.47	0.23
2004	Malayer	195	55934	0.34	0.32	0.2	0.52	56.65	0.65	2.47	0.23
2004	Nahavand	196	34525	0.28	0.29	0.15	0.46	48.99	0.60	2.50	0.23
2004	Hamadan	197	120988	0.42	0.39	0.28	0.57	81.37	0.63	2.49	0.23
2004	Kabudarahang	198	23823	0.23	0.34	0.11	0.4	16.49	0.56	2.43	0.23
2004	Asadabad	199	19185	0.3	0.32	0.15	0.53	47.10	0.74	2.56	0.23
2004	Bahar	200	22558	0.25	0.09	0.12	0.44	42.15	0.61	2.47	0.23
2004	Razan	201	21473	0.23	0.18	0.11	0.42	18.32	0.71	2.46	0.23
2004	Borujen	202	21288	0.28	0.19	0.14	0.5	80.80	0.29	2.27	0.23
2004	Shahrekord	203	70349	0.58	0.64	0.38	0.82	72.25	0.26	2.24	0.23
2004	Farsan	204	13797	0.27	0.15	0.13	0.5	62.95	0.24	2.19	0.23
2004	Lordakan	205	23806	0.2	0.09	0.1	0.36	19.09	0.17	2.35	0.23
2004	Ardal	206	8004	0.21	NA	0.07	0.47	14.96	0.24	2.10	0.23
2004	Kuhrang	207	4780	0.19	NA	0.07	0.41	6.48	0.16	2.22	0.23
2004	Aligudarz	208	22259	0.26	0.18	0.13	0.46	57.83	0.56	2.54	0.26
2004	Borujerd	209	62377	0.46	0.38	0.29	0.68	75.43	0.49	2.52	0.26
2004	Khorramabad	210	86036	0.92	1	0.67	1.21	66.74	0.42	2.39	0.26
2004	Dalfan	211	19362	0.2	0.11	0.09	0.38	41.04	0.49	2.45	0.26
2004	Dorud	212	26513	0.34	0.31	0.18	0.58	65.71	0.35	2.61	0.26
2004	Kuhdasht	213	30752	0.2	0.14	0.1	0.35	49.69	0.48	2.66	0.26
2004	Azna	214	13258	0.27	0.15	0.13	0.49	52.47	0.39	2.56	0.26
2004	Poldokhtar	215	12068	0.33	0.35	0.15	0.62	36.62	0.47	2.38	0.26
2004	Selseleh	216	11558	0.33	NA	0.11	0.78	39.47	0.49	2.54	0.26
2004	Ilam	217	31754	0.51	0.47	0.29	0.81	81.28	0.70	2.26	0.31
2004	Darrehshahr	218	9342	0.22	0.23	0.08	0.44	36.97	0.72	2.36	0.31
2004	Dehloran	219	9217	0.34	NA	0.13	0.72	63.40	0.60	2.41	0.31
2004	Shirvan va Chardavol	220	12216	0.23	NA	0.09	0.47	20.62	0.68	2.41	0.31
2004	Mehran	221	8456	0.43	NA	0.17	0.88	57.31	0.63	2.25	0.31
2004	Abdanan	222	7509	0.28	NA	0.11	0.57	58.34	0.63	2.25	0.31
2004	Eyvan	223	8581	0.46	0.24	0.21	0.86	61.58	0.71	2.34	0.31
2004	Boyerahmad	224	28895	0.4	0.46	0.22	0.66	41.04	0.26	2.45	0.26
2004	Kohgiluyeh	225	33373	0.21	NA	0.08	0.43	37.07	0.14	2.56	0.26
2004	Gachsaran	226	23084	0.47	0.63	0.25	0.78	61.53	0.23	2.14	0.26
2004	Dena	227	8621	0.24	NA	0.1	0.5	19.45	0.19	2.34	0.26
2004	Bushehr	228	34697	0.98	0.87	0.63	1.43	85.47	1.18	2.69	0.74
2004	Tangestan	229	11236	0.4	0.54	0.19	0.74	22.86	1.22	2.82	0.74
2004	Dashtestan	230	39457	0.43	0.46	0.25	0.69	61.28	1.22	2.74	0.74
2004	Dashti	231	12947	0.44	0.63	0.22	0.79	53.83	1.22	2.82	0.74
2004	Dayyer	232	7579	0.39	0.28	0.18	0.74	68.27	1.13	2.89	0.74
2004	Kangan	233	8947	0.51	0.72	0.24	0.92	68.60	1.33	2.92	0.74
2004	Genaveh	234	14687	0.68	0.7	0.36	1.13	73.45	1.26	2.88	0.74
2004	Deylam	235	5082	0.59	NA	0.23	1.25	72.37	1.19	2.81	0.74
2004	Jam	236	5080	0.28	NA	0.1	0.62	35.03	1.25	2.76	0.74
2004	Abhar	237	30476	0.29	NA	0.13	0.57	61.66	0.42	2.15	0.32

2004	Khodabandeh	238	27467	0.16	0.07	0.08	0.3	25.02	0.46	2.04	0.32
2004	Zanjan	239	82241	0.36	0.3	0.23	0.53	76.52	0.47	2.09	0.32
2004	Ijrud	240	7091	0.21	NA	0.08	0.45	6.57	0.53	2.11	0.32
2004	Khorramdarreh	241	11125	0.4	NA	0.17	0.81	78.35	0.36	2.23	0.32
2004	Tarom	242	8040	0.22	0.25	0.08	0.46	12.71	0.35	2.16	0.32
2004	Mahneshan	243	7401	0.22	NA	0.08	0.49	12.70	0.46	2.23	0.32
2004	Damghan	244	16948	0.45	0.23	0.23	0.77	65.84	2.36	4.50	0.35
2004	Semnan	245	34672	0.48	0.47	0.28	0.76	88.32	2.34	4.46	0.35
2004	Shahrud	246	44790	0.89	0.98	0.59	1.26	62.06	2.37	4.39	0.35
2004	Garmsar	247	15881	0.6	0.76	0.32	1	65.20	2.31	4.60	0.35
2004	Ardakan	248	12068	0.51	0.49	0.26	0.89	80.14	1.59	3.39	0.37
2004	Bafq	249	8824	0.57	0.7	0.28	1.03	65.18	1.57	3.48	0.37
2004	Taft	250	11248	0.58	1.14	0.29	1.03	30.65	1.55	3.34	0.37
2004	Mehriz	251	8639	0.58	0.45	0.29	1.03	53.35	1.58	3.23	0.37
2004	Yazd	252	86510	1.32	1.34	1.02	1.66	95.39	1.63	3.24	0.37
2004	Meybod	253	12157	0.62	0.66	0.32	1.06	90.04	1.57	3.25	0.37
2004	Abarkuh	254	8220	0.53	0.48	0.25	0.96	62.44	1.59	3.29	0.37
2004	Sadugh	255	5332	0.57	NA	0.21	1.25	52.14	1.59	3.38	0.37
2004	Khatam	256	5564	0.44	NA	0.17	0.93	54.41	1.58	3.35	0.37
2004	Tabas	257	11385	0.47	0.53	0.23	0.85	53.45	1.64	3.35	0.37
2004	Abumusa	258	163	0.45	NA	0.17	1.01	60.00	0.35	2.16	0.34
2004	Bandarabbas	259	80059	0.51	0.49	0.34	0.73	67.52	0.32	2.35	0.34
2004	Bandar-e Lengeh	260	21241	0.37	0.5	0.2	0.62	46.49	0.35	2.21	0.34
2004	Qeshm	261	13507	0.37	0.47	0.18	0.67	38.72	0.39	2.26	0.34
2004	Minab	262	36813	0.25	0.22	0.13	0.42	23.65	0.35	2.15	0.34
2004	Bandar-e-Jask	263	9940	0.25	0.41	0.12	0.44	15.77	0.35	2.28	0.34
2004	Rudan	264	15395	0.19	0.13	0.08	0.36	33.10	0.26	2.30	0.34
2004	Hajiabad	265	10945	0.22	NA	0.08	0.46	29.99	0.28	2.22	0.34
2004	Bastak	266	10174	0.29	0.2	0.13	0.55	23.81	0.35	2.27	0.34
2004	Tehran	267	1717971	1.68	1.68	1.59	1.76	99.62	2.07	3.89	0.34
2004	Damavand	268	17299	0.64	0.71	0.34	1.07	69.63	2.11	3.98	0.34
2004	Rey	269	42613	1.15	1.3	0.78	1.6	27.91	2.07	4.05	0.34
2004	Shemiranat	270	7644	1.47	2.31	0.79	2.41	54.77	2.10	3.87	0.34
2004	Varamin	271	87507	0.51	0.51	0.34	0.71	78.70	2.09	3.84	0.34
2004	Shahriyar	272	149470	0.32	0.32	0.22	0.46	82.91	2.11	3.97	0.34
2004	Eslamshahr	273	74903	0.25	0.17	0.14	0.38	88.65	1.96	3.93	0.34
2004	Robatkarim	274	82214	0.16	0.08	0.09	0.26	81.61	2.10	3.98	0.34
2004	Pakdasht	275	33825	0.2	0.07	0.1	0.36	72.17	2.06	3.90	0.34
2004	Firuzkuh	276	7919	0.31	NA	0.12	0.66	37.68	2.10	4.03	0.34
2004	Ardebil	277	94846	0.2	0.09	0.11	0.31	81.41	0.21	1.93	0.26
2004	Bilehsowar	278	9709	0.24	NA	0.09	0.49	35.54	0.25	2.02	0.26
2004	Khalkhal	279	18843	0.37	NA	0.13	0.84	43.04	0.35	1.84	0.26
2004	Meshginshahr	280	29037	0.3	0.14	0.16	0.51	39.62	0.24	1.97	0.26
2004	Germi	281	16437	0.24	NA	0.1	0.49	28.80	0.24	1.93	0.26
2004	Parsabad	282	25568	0.22	NA	0.09	0.43	50.98	0.19	1.99	0.26
2004	Kowsar	283	5556	0.23	NA	0.07	0.58	23.77	0.37	1.90	0.26
2004	Namin	284	11130	0.25	NA	0.1	0.52	33.15	0.27	1.99	0.26

2004	Neer	285	4940	0.22	NA	0.08	0.5	22.57	0.26	1.79	0.26
2004	Qom	286	174772	1.11	1.19	0.91	1.35	92.35	2.50	3.24	0.37
2004	Bueenzahra	287	27632	0.2	0.22	0.09	0.35	33.02	1.05	2.95	0.35
2004	Takestan	288	29751	0.31	0.2	0.16	0.52	55.36	1.12	2.81	0.35
2004	Qazvin	289	128689	0.55	0.53	0.39	0.72	77.90	1.08	2.84	0.35
2004	Abyek	290	15224	0.34	NA	0.15	0.69	46.99	1.11	2.98	0.35
2004	Bandare Gaz	291	10009	0.46	0.39	0.23	0.82	52.74	0.77	2.58	0.46
2004	Torkman	292	22226	0.36	0.27	0.19	0.61	59.84	0.84	2.69	0.46
2004	Aliabad	293	22073	0.44	NA	0.19	0.89	46.50	0.76	2.69	0.46
2004	Kordkuy	294	14180	0.57	0.84	0.3	0.96	47.60	0.88	2.72	0.46
2004	Gorgan	295	73711	0.88	0.92	0.63	1.18	68.79	0.76	2.60	0.46
2004	Gonbade Kavus	296	49354	0.38	0.34	0.22	0.6	48.97	0.78	2.68	0.46
2004	Minudasht	297	22186	0.3	0.37	0.15	0.53	32.49	0.80	2.58	0.46
2004	Aqqala	298	18072	0.22	NA	0.09	0.43	29.12	0.84	2.62	0.46
2004	Kalaleh	299	23089	0.2	0.09	0.09	0.36	22.53	0.74	2.74	0.46
2004	Azadshahr	300	15269	0.36	0.41	0.17	0.65	53.49	0.82	2.64	0.46
2004	Ramyan	301	13764	0.21	NA	0.08	0.43	39.19	0.75	2.62	0.46
2004	Esfarayen	302	22734	0.19	0.09	0.09	0.34	36.85	-0.04	2.63	0.40
2004	Bojnurd	303	52666	0.48	0.58	0.29	0.73	56.65	-0.04	2.66	0.40
2004	Jajarm	304	10349	0.22	NA	0.08	0.49	60.09	-0.06	2.67	0.40
2004	Shirvan	305	28307	0.25	0.21	0.13	0.45	49.08	-0.11	2.72	0.40
2004	Faruj	306	10334	0.19	NA	0.07	0.4	23.99	-0.11	2.61	0.40
2004	Maneh va Semelqan	307	15022	0.23	0.14	0.1	0.44	22.91	-0.05	2.59	0.40
2004	Birjand	308	49101	0.48	0.44	0.3	0.73	54.41	-1.14	2.69	0.24
2004	Sarbisheh	309	9192	0.29	NA	0.08	0.74	22.52	-1.07	2.39	0.24
2004	Qaenat	310	25322	0.2	0.08	0.1	0.36	31.62	0.30	2.63	0.24
2004	Nehbandan	311	8930	0.22	NA	0.07	0.51	24.19	-1.04	2.61	0.24
2004	Sarayan	312	6753	0.35	NA	0.13	0.77	57.56	-1.15	2.40	0.24
2004	Ferdows	313	13162	0.43	0.75	0.21	0.77	65.21	0.42	2.75	0.24
2004	Karaj	314	307522	0.41	0.41	0.32	0.51	96.37	2.03	4.06	0.47
2004	Savojbolagh	315	36567	0.09	NA	0.03	0.2	35.09	2.05	4.10	0.47
2004	Nazarabad	316	21052	0.29	NA	0.12	0.58	77.48	2.11	3.95	0.47
2005	Arak	1	119107	0.69	0.69	0.5	0.9	78.67	1.29	3.18	0.35
2005	Ashtiyan	2	4401	0.58	NA	0.22	1.23	32.12	1.33	3.26	0.35
2005	Tafresh	3	13686	0.42	NA	0.18	0.85	25.79	1.35	3.29	0.35
2005	Khomeyn	4	24561	0.37	0.32	0.2	0.61	51.67	1.28	3.33	0.35
2005	Delijan	5	9197	0.56	0.22	0.27	1.01	66.09	1.24	3.26	0.35
2005	Saveh	6	41820	0.34	0.2	0.19	0.54	67.26	1.27	3.46	0.35
2005	Shazand	7	25563	0.41	0.47	0.21	0.69	28.75	1.34	3.37	0.35
2005	Mahalat	8	10622	0.62	0.74	0.3	1.08	79.10	1.29	3.20	0.35
2005	Zarandiyeh	9	11801	0.36	NA	0.15	0.74	55.12	1.33	3.21	0.35
2005	Komijan	10	7892	0.36	0.25	0.14	0.73	38.43	1.31	3.23	0.35
2005	Astara	11	15375	0.36	0.13	0.17	0.65	60.24	0.67	3.79	0.28
2005	Astanehye Ashrafiyeh	12	26999	0.57	0.58	0.32	0.91	43.09	0.76	3.83	0.28
2005	Bandar Anzali	13	32236	0.9	0.89	0.57	1.31	82.07	0.73	3.81	0.28
2005	Tavalesh	14	34207	0.36	0.36	0.2	0.59	31.47	0.86	3.70	0.28
2005	Rasht	15	194099	0.97	0.97	0.79	1.17	67.59	0.69	3.90	0.28

2005	Rudbar	16	22405	0.43	0.26	0.23	0.71	49.55	0.73	3.74	0.28
2005	Rudsar	17	37107	1.19	1.41	0.81	1.66	42.43	0.74	3.80	0.28
2005	Sumehsara	18	30763	0.72	0.89	0.44	1.1	29.39	0.81	3.59	0.28
2005	Fuman	19	23670	0.51	0.58	0.28	0.82	24.90	0.70	3.84	0.28
2005	Langrud	20	32895	0.66	0.65	0.4	1	59.44	0.74	3.72	0.28
2005	Lahijan	21	40443	1.02	1.14	0.69	1.43	42.72	0.73	3.74	0.28
2005	Shaft	22	15236	0.35	0.25	0.17	0.63	11.95	0.71	3.64	0.28
2005	Amlash	23	11366	0.44	0.34	0.21	0.81	30.91	0.74	3.68	0.28
2005	Rezvanshahr	24	13594	0.52	0.74	0.25	0.91	27.84	0.78	3.82	0.28
2005	Siyahkal	25	11576	0.62	0.83	0.31	1.05	30.45	0.71	3.83	0.28
2005	Masal	26	10386	0.45	0.57	0.21	0.81	27.27	0.76	3.67	0.28
2005	Amol	27	73670	0.78	0.81	0.55	1.06	54.29	1.93	3.87	0.38
2005	Babol	28	102010	0.98	0.99	0.74	1.25	45.91	1.88	4.03	0.38
2005	Behshahr	29	41227	0.78	0.87	0.51	1.14	60.39	1.99	3.88	0.38
2005	Tonekabon	30	44319	0.58	0.58	0.37	0.87	46.84	1.91	3.94	0.38
2005	Ramsar	31	16262	0.67	0.48	0.37	1.11	70.92	1.92	4.01	0.38
2005	Sari	32	104194	1.07	1.12	0.82	1.35	50.27	1.91	4.05	0.38
2005	Savadkuh	33	15650	0.4	NA	0.17	0.82	46.68	1.87	3.86	0.38
2005	Qaemshahr	34	64290	0.6	0.59	0.4	0.85	58.23	1.89	3.74	0.38
2005	Nur	35	22619	0.45	0.54	0.24	0.75	38.23	1.92	4.01	0.38
2005	Noshahr	36	24771	0.27	0.24	0.13	0.47	34.60	1.91	3.85	0.38
2005	Babolsar	37	36922	0.69	0.77	0.43	1.05	51.03	1.98	3.88	0.38
2005	Mahmudabad	38	19343	0.51	0.53	0.27	0.86	31.49	1.97	3.86	0.38
2005	Neka	39	21190	0.53	0.66	0.28	0.88	35.32	1.97	3.97	0.38
2005	Chalus	40	25898	0.35	0.31	0.19	0.6	51.65	1.95	3.86	0.38
2005	Juybar	41	15047	0.77	1.2	0.41	1.29	38.66	1.94	3.97	0.38
2005	Ahar	42	28804	0.5	0.77	0.28	0.82	61.80	0.66	2.56	0.39
2005	Tabriz	43	326297	1.21	1.23	1.05	1.39	93.20	0.65	2.48	0.39
2005	Sarab	44	26086	0.77	1.15	0.45	1.18	41.54	0.72	2.53	0.39
2005	Maragheh	45	46530	0.59	0.74	0.37	0.88	68.42	0.65	2.44	0.39
2005	Marand	46	48559	1.23	1.45	0.86	1.65	58.01	0.64	2.48	0.39
2005	Miyaneh	47	38659	0.39	0.4	0.23	0.62	48.71	0.68	2.54	0.39
2005	Hashtrud	48	12049	0.51	0.99	0.25	0.91	28.13	0.69	2.66	0.39
2005	Bonab	49	24648	0.6	0.76	0.34	0.95	60.73	0.63	2.58	0.39
2005	Bostanabad	50	17145	0.35	0.35	0.17	0.61	18.75	0.68	2.44	0.39
2005	Shabestar	51	28525	0.57	0.81	0.33	0.9	46.64	0.77	2.40	0.39
2005	Kalibar	52	16199	0.26	0.36	0.12	0.49	13.82	0.65	2.42	0.39
2005	Haris	53	12906	0.46	0.47	0.23	0.81	45.01	0.66	2.47	0.39
2005	Jolfa	54	11804	0.37	0.34	0.17	0.7	60.14	0.62	2.66	0.39
2005	Malekan	55	18778	0.3	0.33	0.15	0.54	31.76	0.63	2.41	0.39
2005	Azarshahr	56	21276	0.39	0.47	0.2	0.68	59.63	0.63	2.42	0.39
2005	Osku	57	17627	0.29	0.23	0.14	0.52	51.51	0.65	2.50	0.39
2005	Charoymaq	58	5549	0.25	0.37	0.1	0.51	12.21	0.66	2.72	0.39
2005	Varzaqan	59	9017	0.26	NA	0.11	0.55	10.25	0.63	2.34	0.39
2005	Ajabshir	60	13307	0.39	NA	0.16	0.78	41.78	0.66	2.58	0.39
2005	Orumiyeh	61	166185	0.82	0.82	0.64	1.01	69.97	0.97	2.24	0.37
2005	Piranshahr	62	16620	0.33	0.26	0.16	0.6	50.02	0.87	2.45	0.37

2005	Khoy	63	68351	0.29	0.27	0.18	0.45	65.47	0.89	2.26	0.37
2005	Sardasht	64	16706	0.22	0.12	0.1	0.41	46.05	0.99	2.41	0.37
2005	Salmas	65	31255	0.7	0.85	0.42	1.06	54.35	0.99	2.19	0.37
2005	Maku	66	29826	0.25	0.28	0.13	0.44	48.83	0.90	2.33	0.37
2005	Mahabad	67	36405	0.34	0.34	0.18	0.55	68.79	0.93	2.41	0.37
2005	Miyandoab	68	45708	0.32	0.27	0.18	0.5	51.31	0.94	2.31	0.37
2005	Naqadeh	69	22625	0.38	0.18	0.2	0.64	67.49	0.93	2.31	0.37
2005	Bukan	70	36141	0.37	0.46	0.2	0.6	72.48	0.90	2.24	0.37
2005	Shahindezh	71	17301	0.37	0.47	0.18	0.67	49.61	0.92	2.26	0.37
2005	Takab	72	15408	0.25	0.13	0.11	0.47	54.53	0.93	2.36	0.37
2005	Oshnaviyeh	73	10504	0.57	0.39	0.28	1.02	53.83	0.87	2.39	0.37
2005	Chaldoran	74	7193	0.31	0.28	0.12	0.63	34.65	0.82	2.19	0.37
2005	Eslamabade Gharb	75	38004	0.28	0.27	0.15	0.46	51.47	0.40	2.49	0.35
2005	Kermanshah	76	186449	0.86	0.85	0.69	1.05	84.23	0.51	2.53	0.35
2005	Paveh	77	10735	0.36	0.19	0.16	0.67	49.98	0.42	2.37	0.35
2005	Sarpole Zahab	78	14687	0.45	0.56	0.22	0.8	40.51	0.46	2.56	0.35
2005	Sonqor	79	19919	0.39	0.29	0.2	0.66	47.22	0.40	2.49	0.35
2005	Qasreshirin	80	4169	0.61	0.49	0.26	1.19	69.01	0.44	2.61	0.35
2005	Kangavar	81	15813	0.39	0.13	0.2	0.68	61.28	0.41	2.55	0.35
2005	Gilanegharb	82	12445	0.31	0.33	0.14	0.58	33.47	0.40	2.50	0.35
2005	Javanrud	83	18045	0.33	NA	0.14	0.67	53.76	0.43	2.48	0.35
2005	Sahneh	84	15713	0.37	0.25	0.18	0.66	42.29	0.42	2.37	0.35
2005	Harsin	85	16207	0.35	0.13	0.17	0.61	64.56	0.46	2.49	0.35
2005	Salas-e-Babajani	86	5478	0.27	NA	0.09	0.62	21.11	0.41	2.30	0.35
2005	Abadan	87	49182	1.73	1.82	1.28	2.25	84.04	1.42	3.06	0.50
2005	Andimeshk	88	26904	0.71	0.79	0.42	1.11	77.40	1.45	3.08	0.50
2005	Ahvaz	89	220422	1.14	1.13	0.95	1.35	82.60	1.49	3.09	0.50
2005	Izeh	90	29514	0.38	0.35	0.2	0.62	52.79	1.49	3.07	0.50
2005	Bandar-e-Mahshahr	91	38971	0.74	0.61	0.46	1.11	94.14	1.44	3.13	0.50
2005	Behbahan	92	32739	0.78	0.74	0.48	1.16	71.96	1.42	3.25	0.50
2005	Khorramshahr	93	26069	0.86	0.8	0.52	1.3	83.60	1.45	3.08	0.50
2005	Dezful	94	65160	0.81	0.83	0.56	1.11	73.73	1.47	3.13	0.50
2005	Dashte Azadegan	95	18378	0.86	1.14	0.48	1.36	56.55	1.49	3.08	0.50
2005	Ramhormoz	96	28186	0.49	0.3	0.27	0.79	52.97	1.51	2.99	0.50
2005	Shadegan	97	19426	0.62	0.54	0.34	1.02	39.07	1.53	3.12	0.50
2005	Shushtar	98	38829	0.89	0.98	0.57	1.3	60.68	1.46	3.06	0.50
2005	Masjedsoleyman	99	29148	0.55	0.49	0.31	0.87	66.87	1.37	3.02	0.50
2005	Shush	100	28765	0.51	0.37	0.29	0.82	46.06	1.37	3.20	0.50
2005	Baghmalek	101	14902	0.44	0.55	0.22	0.78	35.37	1.48	3.18	0.50
2005	Omidiyeh	102	14233	0.72	0.73	0.38	1.2	69.11	1.43	3.22	0.50
2005	Lali	103	5293	0.56	NA	0.2	1.22	47.12	1.35	3.14	0.50
2005	Hendijan	104	6415	0.86	NA	0.35	1.76	69.81	1.48	3.02	0.50
2005	Abadeh	105	19783	0.76	0.6	0.43	1.2	88.51	1.18	3.13	0.38
2005	Estahban	106	12345	0.68	0.81	0.35	1.17	71.01	1.15	3.16	0.38
2005	Eqlid	107	18161	0.55	0.45	0.3	0.91	63.18	1.15	3.08	0.38
2005	Jahrom	108	37981	0.77	0.84	0.49	1.14	64.05	1.18	3.14	0.38
2005	Darab	109	29651	0.64	0.69	0.37	0.98	42.31	1.18	2.96	0.38

2005	Sepidan	110	15619	0.38	0.39	0.19	0.69	20.67	1.17	3.19	0.38
2005	Shiraz	111	327126	1.46	1.48	1.29	1.66	87.83	1.17	3.16	0.38
2005	Fasa	112	35961	0.95	1.06	0.61	1.36	57.93	1.18	2.96	0.38
2005	Firuzabad	113	21601	0.58	0.57	0.32	0.95	56.21	1.11	3.12	0.38
2005	Kazerun	114	49823	0.65	0.73	0.42	0.95	51.42	1.17	3.09	0.38
2005	Lar (Larestan)	115	46526	0.58	0.48	0.36	0.86	58.09	1.14	3.13	0.38
2005	Marvdasht	116	56307	0.42	0.37	0.25	0.64	45.48	1.18	3.11	0.38
2005	Mamasany	117	32557	0.38	0.44	0.2	0.61	32.57	1.19	3.03	0.38
2005	Neyriz	118	19121	0.57	0.64	0.31	0.94	58.86	1.17	3.20	0.38
2005	Lamard	119	12715	0.5	0.32	0.25	0.87	43.27	1.18	3.24	0.38
2005	Bovanat	120	8949	0.41	NA	0.17	0.84	29.97	1.07	3.15	0.38
2005	Arsanjan	121	7086	0.87	1.19	0.41	1.59	39.49	1.18	3.05	0.38
2005	Khorrambid	122	8188	0.52	NA	0.21	1.08	77.32	1.17	3.08	0.38
2005	Zarrindasht	123	9585	0.48	NA	0.18	1.03	56.70	1.18	3.15	0.38
2005	Qirokarzin	124	10911	0.36	0.19	0.16	0.68	55.38	1.15	3.06	0.38
2005	Mohr	125	8149	0.45	NA	0.18	0.95	36.17	1.20	3.17	0.38
2005	Farashband	126	8004	0.52	NA	0.22	1.07	54.69	1.18	3.15	0.38
2005	Baft	127	26141	0.45	0.53	0.25	0.74	35.70	0.55	3.47	0.62
2005	Bam	128	39587	0.35	0.32	0.19	0.56	38.62	0.52	3.47	0.62
2005	Jiroft	129	35225	0.4	0.35	0.22	0.65	40.79	0.52	3.53	0.62
2005	Rafsanjan	130	51156	0.77	0.76	0.5	1.08	56.90	0.57	3.49	0.62
2005	Zarand	131	26426	0.38	0.08	0.21	0.64	52.78	0.51	3.59	0.62
2005	Sirjan	132	42506	0.61	0.53	0.38	0.92	75.10	0.50	3.57	0.62
2005	Shahrebabak	133	17775	0.5	0.33	0.26	0.85	63.29	0.48	3.53	0.62
2005	Kerman	134	119322	0.88	0.86	0.67	1.11	87.65	0.51	3.54	0.62
2005	Kahnuj	135	34569	0.27	0.25	0.14	0.46	21.69	0.54	3.53	0.62
2005	Bardsir	136	15030	0.42	0.54	0.2	0.74	54.92	0.54	3.51	0.62
2005	Ravar	137	7599	0.5	NA	0.2	1.06	58.39	0.51	3.45	0.62
2005	Anbarabad	138	11018	0.34	NA	0.12	0.77	32.99	0.51	3.58	0.62
2005	Manujan	139	8486	0.29	0.25	0.11	0.59	28.69	0.57	3.48	0.62
2005	Taybad	140	21371	0.41	0.59	0.21	0.71	45.51	0.65	2.78	0.49
2005	Torbate Heydarieh	141	57992	0.45	0.41	0.28	0.67	46.22	0.68	2.73	0.49
2005	Torbate Jam	142	36683	0.47	0.45	0.27	0.73	43.72	0.60	2.84	0.49
2005	Darrehgaz	143	15194	0.4	0.26	0.2	0.71	55.05	0.49	2.73	0.49
2005	Sabzevar	144	86414	0.53	0.53	0.36	0.74	49.31	0.56	2.75	0.49
2005	Quchan	145	34760	0.41	0.4	0.23	0.66	50.09	0.59	2.74	0.49
2005	Kashmar	146	37558	0.6	0.69	0.36	0.91	48.78	0.66	2.82	0.49
2005	Gonabad	147	22969	0.56	0.6	0.31	0.9	48.99	0.62	2.66	0.49
2005	Mashhad	148	519381	1.12	1.13	0.99	1.25	90.33	0.54	2.79	0.49
2005	Neyshabur	149	83748	0.46	0.45	0.31	0.66	51.92	0.64	2.87	0.49
2005	Chenaran	150	19350	0.37	NA	0.16	0.75	39.72	0.58	2.81	0.49
2005	Khaf	151	16395	0.35	NA	0.15	0.7	45.06	0.65	2.85	0.49
2005	Sarakhs	152	12964	0.54	0.67	0.27	0.95	40.55	0.61	2.90	0.49
2005	Fariman	153	14929	0.44	0.41	0.22	0.78	53.71	0.67	2.82	0.49
2005	Bardeskan	154	13410	0.28	0.3	0.13	0.53	36.17	0.60	2.76	0.49
2005	Rashtkhar	155	9419	0.29	NA	0.1	0.66	18.81	0.55	2.85	0.49
2005	Kalat	156	7238	0.5	NA	0.18	1.09	24.15	0.57	2.82	0.49

2005	Ardestan	157	10329	0.59	0.91	0.29	1.06	55.12	1.70	3.66	0.31
2005	Isfahan	158	400176	1.68	1.69	1.5	1.86	91.54	1.71	3.55	0.31
2005	Khomeynishahr	159	51056	0.56	0.49	0.35	0.83	96.49	1.74	3.48	0.31
2005	Khansar	160	7836	0.44	0.24	0.21	0.8	62.06	1.71	3.52	0.31
2005	Semirom	161	13571	0.47	0.45	0.23	0.84	50.91	1.78	3.50	0.31
2005	Faridan	162	17056	0.73	0.8	0.4	1.18	41.16	1.74	3.64	0.31
2005	Fereydunshahr	163	7563	0.42	0.52	0.19	0.79	47.48	1.70	3.51	0.31
2005	Falavarjan	164	41176	0.5	0.41	0.3	0.76	61.54	1.70	3.69	0.31
2005	Shahreza	165	37331	0.88	1.02	0.57	1.28	79.27	1.67	3.59	0.31
2005	Kashan	166	61501	1.22	1.25	0.89	1.61	85.42	1.70	3.63	0.31
2005	Golpayegan	167	19049	0.54	0.5	0.29	0.88	73.46	1.72	3.69	0.31
2005	Lanjan	168	42656	0.58	0.44	0.36	0.87	82.66	1.74	3.46	0.31
2005	Nayin	169	12447	0.51	0.46	0.26	0.89	66.79	1.72	3.43	0.31
2005	Najafabad	170	53420	0.66	0.54	0.43	0.96	91.71	1.80	3.63	0.31
2005	Natanz	171	9707	0.51	NA	0.21	1.01	55.48	1.72	3.72	0.31
2005	Shahinshahr va Meyme	172	54770	0.9	0.88	0.62	1.24	89.76	1.72	3.64	0.31
2005	Mobarakeh	173	25184	0.61	0.66	0.35	0.97	81.62	1.76	3.61	0.31
2005	Aran va Bidgol	174	17804	0.54	0.35	0.29	0.91	85.51	1.80	3.61	0.31
2005	Tiran va Karvan	175	13201	0.38	NA	0.15	0.78	37.77	1.80	3.60	0.31
2005	Chadegan	176	6624	0.37	NA	0.14	0.78	39.60	1.73	3.52	0.31
2005	Iranshahr	177	32541	0.39	0.46	0.21	0.65	42.72	-1.06	1.83	0.44
2005	Chah Bahar	178	30375	0.25	NA	0.11	0.51	32.40	-1.19	1.71	0.44
2005	Khash	179	18807	0.22	0.11	0.1	0.41	31.31	-1.12	1.70	0.44
2005	Zabol	180	56211	0.39	0.51	0.24	0.6	37.79	-1.16	1.78	0.44
2005	Zahedan	181	79537	0.71	0.78	0.49	0.98	87.45	-1.14	1.84	0.44
2005	Saravan	182	33919	0.16	0.12	0.08	0.29	36.07	-1.08	1.95	0.44
2005	Nikshahr	183	25195	0.16	0.08	0.07	0.3	21.63	-1.13	1.89	0.44
2005	Sarbaz	184	17137	0.21	NA	0.06	0.54	11.11	-1.13	1.80	0.44
2005	Baneh	185	19600	0.32	0.21	0.16	0.57	61.30	0.37	1.93	0.53
2005	Bijar	186	20777	0.3	0.28	0.15	0.53	52.83	0.28	1.84	0.53
2005	Saqqez	187	38564	0.42	0.47	0.24	0.66	64.44	0.34	2.01	0.53
2005	Sanandaj	188	77957	0.63	0.61	0.43	0.87	81.53	0.32	1.79	0.53
2005	Qorveh	189	37383	0.29	0.22	0.16	0.48	51.57	0.40	1.79	0.53
2005	Marivan	190	24375	0.37	0.18	0.19	0.63	61.61	0.37	1.91	0.53
2005	Divandarreh	191	14336	0.29	0.28	0.13	0.53	26.46	0.40	1.90	0.53
2005	Kamyaran	192	18494	0.3	NA	0.12	0.61	42.92	0.35	1.79	0.53
2005	Sarvabad	193	10020	0.22	NA	0.09	0.47	5.05	0.33	1.85	0.53
2005	Tuyserkan	194	23741	0.36	0.32	0.19	0.61	44.44	0.80	2.54	0.29
2005	Malayer	195	57408	0.39	0.35	0.24	0.58	57.23	0.89	2.47	0.29
2005	Nahavand	196	35423	0.29	0.22	0.16	0.48	49.36	0.92	2.54	0.29
2005	Hamadan	197	125732	0.98	1.04	0.76	1.23	81.66	0.86	2.72	0.29
2005	Kabudarahang	198	24279	0.21	0.08	0.1	0.37	16.71	0.78	2.66	0.29
2005	Asadabad	199	19691	0.33	0.2	0.17	0.58	47.57	0.78	2.65	0.29
2005	Bahar	200	23123	0.47	0.6	0.25	0.76	42.42	0.91	2.50	0.29
2005	Razan	201	21914	0.27	0.18	0.13	0.48	18.74	0.88	2.64	0.29
2005	Borujen	202	22101	0.38	0.37	0.2	0.65	80.99	0.42	2.21	0.25
2005	Shahrekord	203	73013	0.59	0.59	0.39	0.82	72.64	0.37	2.44	0.25

2005	Farsan	204	14351	0.35	0.29	0.17	0.64	63.32	0.36	2.36	0.25
2005	Lordakan	205	24993	0.29	NA	0.13	0.58	19.47	0.44	2.32	0.25
2005	Ardal	206	8333	0.26	NA	0.09	0.62	15.08	0.37	2.38	0.25
2005	Kuhrang	207	5015	0.24	NA	0.09	0.51	6.65	0.45	2.31	0.25
2005	Aligudarz	208	22917	0.36	NA	0.16	0.73	58.42	0.72	2.68	0.28
2005	Borujerd	209	64119	0.86	0.9	0.61	1.17	75.56	0.71	2.57	0.28
2005	Khorramabad	210	89962	0.8	0.82	0.59	1.06	66.93	0.70	2.59	0.28
2005	Dalfan	211	20163	0.29	NA	0.12	0.58	41.35	0.72	2.64	0.28
2005	Dorud	212	27622	0.36	0.23	0.19	0.6	65.92	0.62	2.59	0.28
2005	Kuhdasht	213	32303	0.25	0.2	0.13	0.43	50.04	0.68	2.72	0.28
2005	Azna	214	13626	0.32	0.15	0.15	0.59	52.97	0.71	2.80	0.28
2005	Poldokhtar	215	12666	0.42	NA	0.17	0.85	37.02	0.67	2.61	0.28
2005	Selseleh	216	12058	0.41	NA	0.14	0.95	39.89	0.72	2.51	0.28
2005	Ilam	217	33365	0.88	1.02	0.56	1.31	81.54	0.82	2.54	0.30
2005	Darrehshahr	218	9764	0.27	NA	0.1	0.59	37.44	0.78	2.50	0.30
2005	Dehloran	219	9670	0.38	0.22	0.16	0.75	63.71	0.79	2.47	0.30
2005	Shirvan va Chardavol	220	12714	0.26	0.16	0.11	0.49	21.49	0.80	2.41	0.30
2005	Mehran	221	8954	0.54	NA	0.22	1.11	57.35	0.84	2.45	0.30
2005	Abdanan	222	7821	0.33	0.27	0.15	0.64	58.69	0.82	2.36	0.30
2005	Eyvan	223	8959	0.66	0.69	0.31	1.18	62.23	0.94	2.51	0.30
2005	Boyerahmad	224	30789	0.39	0.29	0.21	0.65	41.80	0.44	2.44	0.23
2005	Kohgiluyeh	225	35032	0.23	0.18	0.11	0.4	38.20	0.48	2.57	0.23
2005	Gachsaran	226	23957	0.38	0.17	0.2	0.63	61.95	0.48	2.57	0.23
2005	Dena	227	8897	0.31	NA	0.13	0.62	19.48	0.38	2.63	0.23
2005	Bushehr	228	35913	1.67	1.73	1.18	2.27	85.48	1.44	3.04	0.72
2005	Tangestan	229	11618	0.47	0.52	0.22	0.84	22.96	1.36	2.92	0.72
2005	Dashtestan	230	40814	0.5	0.5	0.3	0.78	61.51	1.46	2.91	0.72
2005	Dashti	231	13420	0.47	0.45	0.23	0.83	54.72	1.38	2.91	0.72
2005	Dayyer	232	7920	0.56	0.79	0.26	1.01	68.41	1.32	2.97	0.72
2005	Kangan	233	9352	0.48	0.23	0.23	0.88	68.88	1.44	2.90	0.72
2005	Genaveh	234	15310	0.72	0.54	0.38	1.19	73.58	1.39	3.07	0.72
2005	Deylam	235	5294	0.74	NA	0.29	1.6	72.59	1.39	3.12	0.72
2005	Jam	236	5416	0.35	NA	0.13	0.77	35.74	1.40	2.89	0.72
2005	Abhar	237	31608	0.32	0.26	0.16	0.54	62.32	0.64	2.27	0.35
2005	Khodabandeh	238	28349	0.25	0.28	0.13	0.44	25.50	0.56	2.24	0.35
2005	Zanjan	239	85620	0.62	0.62	0.43	0.85	76.99	0.56	2.33	0.35
2005	Ijrud	240	7253	0.27	NA	0.1	0.57	6.70	0.60	2.45	0.35
2005	Khorramdarreh	241	11610	0.42	0.18	0.2	0.77	78.54	0.56	2.30	0.35
2005	Tarom	242	8365	0.28	NA	0.1	0.63	12.94	0.57	2.35	0.35
2005	Mahneshan	243	7554	0.28	NA	0.1	0.63	13.12	0.50	2.29	0.35
2005	Damghan	244	17397	0.65	0.57	0.35	1.06	66.61	2.44	4.63	0.37
2005	Semnan	245	36178	0.53	0.45	0.32	0.82	88.58	2.54	4.65	0.37
2005	Shahrud	246	46192	0.81	0.77	0.53	1.15	62.65	2.52	4.69	0.37
2005	Garmsar	247	16414	0.51	0.24	0.27	0.86	66.22	2.48	4.70	0.37
2005	Ardakan	248	12460	0.55	0.32	0.28	0.96	80.29	1.77	3.67	0.36
2005	Bafq	249	9181	0.6	0.45	0.3	1.08	65.42	1.71	3.53	0.36
2005	Taft	250	11312	0.42	0.16	0.2	0.74	31.32	1.72	3.54	0.36

2005	Mehriz	251	8790	0.75	0.67	0.39	1.3	53.96	1.63	3.48	0.36
2005	Yazd	252	90658	1.73	1.76	1.38	2.12	95.30	1.74	3.51	0.36
2005	Meybod	253	12613	0.62	0.32	0.32	1.06	89.91	1.72	3.62	0.36
2005	Abarkuh	254	8476	0.74	0.93	0.35	1.31	62.58	1.74	3.46	0.36
2005	Sadugh	255	5458	0.7	NA	0.25	1.54	52.59	1.78	3.42	0.36
2005	Khatam	256	5791	0.54	NA	0.21	1.14	54.50	1.66	3.50	0.36
2005	Tabas	257	11797	0.55	0.51	0.27	0.97	54.15	1.76	3.58	0.36
2005	Abumusa	258	186	0.56	NA	0.21	1.24	60.00	0.39	2.30	0.30
2005	Bandarabbas	259	84160	0.6	0.57	0.41	0.83	67.78	0.43	2.41	0.30
2005	Bandar-e Lengeh	260	22371	0.33	0.19	0.18	0.56	46.68	0.41	2.42	0.30
2005	Qeshm	261	14206	0.51	0.75	0.25	0.9	38.98	0.42	2.39	0.30
2005	Minab	262	38408	0.32	0.32	0.18	0.53	23.78	0.36	2.45	0.30
2005	Bandar-e-Jask	263	10348	0.28	NA	0.13	0.52	15.76	0.58	2.54	0.30
2005	Rudan	264	16090	0.25	0.26	0.11	0.47	33.68	0.45	2.36	0.30
2005	Hajiabad	265	11311	0.35	0.71	0.15	0.67	30.28	0.42	2.59	0.30
2005	Bastak	266	10630	0.34	0.19	0.15	0.65	23.68	0.44	2.46	0.30
2005	Tehran	267	1778194	2.05	2.05	1.95	2.14	99.62	2.23	4.19	0.34
2005	Damavand	268	18258	0.82	0.9	0.45	1.32	70.35	2.23	4.21	0.34
2005	Rey	269	45856	1.4	1.53	0.99	1.87	27.18	2.23	4.14	0.34
2005	Shemiranat	270	7962	1.53	1.97	0.84	2.49	55.04	2.17	3.95	0.34
2005	Varamin	271	91939	0.68	0.7	0.48	0.92	79.20	2.27	4.16	0.34
2005	Shahriyar	272	164340	0.38	0.37	0.27	0.52	83.79	2.24	4.17	0.34
2005	Eslamshahr	273	78153	0.33	0.27	0.2	0.49	88.96	2.22	4.27	0.34
2005	Robatkarim	274	90490	0.19	0.1	0.1	0.3	82.17	2.21	4.13	0.34
2005	Pakdasht	275	36201	0.27	0.19	0.14	0.46	73.33	2.18	4.05	0.34
2005	Firuzkuh	276	8221	0.38	NA	0.15	0.8	37.72	2.25	4.07	0.34
2005	Ardebil	277	99145	0.47	0.48	0.31	0.65	81.68	0.56	2.09	0.30
2005	Bilehsowar	278	10010	0.28	0.21	0.12	0.55	36.09	0.52	2.12	0.30
2005	Khalkhal	279	19239	0.47	NA	0.17	1.08	43.64	0.58	2.17	0.30
2005	Meshginshahr	280	29918	0.72	0.94	0.43	1.1	40.38	0.47	2.12	0.30
2005	Germi	281	16774	0.31	0.36	0.15	0.56	29.27	0.57	1.93	0.30
2005	Parsabad	282	27033	0.26	0.24	0.13	0.45	51.49	0.54	1.92	0.30
2005	Kowsar	283	5637	0.29	NA	0.09	0.71	24.19	0.50	1.92	0.30
2005	Namin	284	11351	0.35	0.51	0.16	0.64	33.70	0.51	2.02	0.30
2005	Neer	285	4988	0.28	NA	0.1	0.64	22.92	0.46	2.10	0.30
2005	Qom	286	182491	0.56	0.54	0.42	0.71	92.60	2.62	3.38	0.40
2005	Bueenzahra	287	28471	0.21	0.14	0.1	0.37	33.49	1.26	3.02	0.36
2005	Takestan	288	30848	0.41	0.33	0.23	0.66	56.04	1.36	3.07	0.36
2005	Qazvin	289	134927	0.61	0.59	0.45	0.8	78.38	1.27	3.05	0.36
2005	Abyek	290	15892	0.45	0.53	0.22	0.79	48.01	1.30	2.91	0.36
2005	Bandare Gaz	291	10297	0.83	1.34	0.43	1.41	53.00	1.01	2.87	0.48
2005	Torkman	292	22998	0.47	0.44	0.25	0.77	60.04	0.96	2.68	0.48
2005	Aliabad	293	22820	0.54	0.54	0.29	0.9	46.87	0.96	2.73	0.48
2005	Kordkuy	294	14582	0.56	0.54	0.29	0.95	47.83	1.02	2.78	0.48
2005	Gorgan	295	77137	1.15	1.2	0.86	1.49	69.49	1.00	2.79	0.48
2005	Gonbade Kavus	296	51456	0.68	0.77	0.44	0.98	48.88	1.00	2.65	0.48
2005	Minudasht	297	22968	0.33	0.27	0.16	0.57	33.00	1.02	2.71	0.48

2005	Aqqala	298	18795	0.28	NA	0.11	0.56	29.32	0.92	2.82	0.48
2005	Kalaleh	299	24229	0.25	0.18	0.12	0.45	22.60	0.97	2.75	0.48
2005	Azadshahr	300	15843	0.45	NA	0.19	0.89	53.87	1.06	2.82	0.48
2005	Ramyan	301	14265	0.26	NA	0.1	0.54	39.41	1.00	2.80	0.48
2005	Esfarayen	302	23486	0.22	0.09	0.1	0.4	37.57	0.11	2.76	0.42
2005	Bojnurd	303	54775	0.38	0.34	0.23	0.58	57.21	0.18	2.63	0.42
2005	Jajarm	304	10680	0.27	NA	0.1	0.6	60.46	0.01	2.65	0.42
2005	Shirvan	305	29323	0.29	0.21	0.15	0.5	49.54	0.15	2.64	0.42
2005	Faruj	306	10503	0.23	NA	0.09	0.51	24.34	0.18	2.79	0.42
2005	Maneh va Semelqan	307	15616	0.31	NA	0.12	0.68	23.18	0.07	2.73	0.42
2005	Birjand	308	50926	0.74	0.78	0.49	1.05	55.07	-0.74	2.57	0.23
2005	Sarbisheh	309	9455	0.37	NA	0.11	0.94	22.58	-0.78	2.72	0.23
2005	Qaenat	310	26077	0.31	NA	0.14	0.63	32.18	0.59	2.82	0.23
2005	Nehbandan	311	9362	0.27	NA	0.09	0.65	24.71	-0.78	2.50	0.23
2005	Sarayan	312	6998	0.43	NA	0.16	0.95	57.63	-0.78	2.43	0.23
2005	Ferdows	313	13542	0.47	0.58	0.23	0.81	65.64	0.63	2.76	0.23
2005	Karaj	314	327412	0.41	0.41	0.32	0.51	96.47	2.16	4.13	0.38
2005	Savojbolagh	315	39464	0.11	NA	0.04	0.23	35.49	2.22	4.17	0.38
2005	Nazarabad	316	22228	0.28	0.1	0.13	0.5	77.96	2.28	4.17	0.38
2006	Arak	1	123656	0.78	NA	0.34	1.52	79.22	1.53	3.38	0.38
2006	Ashtiyan	2	4469	0.6	NA	0.22	1.32	32.62	1.53	3.25	0.38
2006	Tafresh	3	13853	0.44	NA	0.18	0.92	26.33	1.58	3.31	0.38
2006	Khomeyn	4	25155	0.43	NA	0.18	0.87	52.75	1.54	3.34	0.38
2006	Delijan	5	9523	0.72	NA	0.3	1.47	66.95	1.45	3.33	0.38
2006	Saveh	6	44053	0.49	NA	0.21	0.96	68.28	1.48	3.28	0.38
2006	Shazand	7	26249	0.39	NA	0.16	0.79	29.25	1.55	3.21	0.38
2006	Mahalat	8	10944	0.62	NA	0.26	1.25	79.60	1.54	3.31	0.38
2006	Zarandiyeh	9	12149	0.38	NA	0.15	0.81	55.67	1.47	3.46	0.38
2006	Komijan	10	8104	0.41	NA	0.15	0.89	38.79	1.52	3.37	0.38
2006	Astara	11	16095	0.47	NA	0.19	0.95	60.82	1.09	3.77	0.31
2006	Astanehye Ashrafiyeh	12	27675	0.61	NA	0.26	1.22	43.63	0.95	3.87	0.31
2006	Bandar Anzali	13	33259	0.98	NA	0.44	1.93	82.20	1.01	3.89	0.31
2006	Tavalesh	14	35760	0.41	NA	0.18	0.81	32.12	0.94	3.92	0.31
2006	Rasht	15	202320	1	NA	0.45	1.96	68.16	0.99	3.83	0.31
2006	Rudbar	16	22983	0.6	NA	0.25	1.18	50.29	1.14	4.00	0.31
2006	Rudsar	17	38169	0.81	NA	0.36	1.58	42.01	1.00	3.93	0.31
2006	Sumehsara	18	31531	0.6	NA	0.26	1.2	30.04	0.96	3.94	0.31
2006	Fuman	19	24279	0.49	NA	0.21	0.97	24.58	0.91	3.85	0.31
2006	Langrud	20	33865	0.74	NA	0.33	1.46	59.96	1.09	3.95	0.31
2006	Lahijan	21	41764	0.85	NA	0.38	1.67	43.66	1.04	3.67	0.31
2006	Shaft	22	15566	0.42	NA	0.17	0.86	11.99	1.03	3.82	0.31
2006	Amlash	23	11684	0.51	NA	0.2	1.07	31.14	1.03	3.88	0.31
2006	Rezvanshahr	24	14115	0.47	NA	0.19	0.98	28.30	1.02	3.84	0.31
2006	Siyahkal	25	11861	0.59	NA	0.25	1.17	31.05	0.96	3.90	0.31
2006	Masal	26	10735	0.44	NA	0.18	0.92	26.79	0.98	3.83	0.31
2006	Amol	27	76570	0.78	NA	0.35	1.51	54.62	2.02	4.16	0.39
2006	Babol	28	105641	1.03	NA	0.46	2	46.24	2.06	4.15	0.39

2006	Behshahr	29	42709	0.72	NA	0.31	1.42	60.66	2.10	4.22	0.39
2006	Tonekabon	30	45860	0.66	NA	0.29	1.3	47.14	2.09	4.17	0.39
2006	Ramsar	31	16730	0.9	NA	0.39	1.79	71.13	2.16	4.30	0.39
2006	Sari	32	108433	0.89	NA	0.4	1.72	50.84	2.06	4.21	0.39
2006	Savadkuh	33	16059	0.43	NA	0.17	0.88	47.12	2.09	4.07	0.39
2006	Qaemshahr	34	66655	0.7	NA	0.31	1.39	58.68	2.05	3.95	0.39
2006	Nur	35	23451	0.42	NA	0.18	0.84	38.57	2.09	3.98	0.39
2006	Noshahr	36	25732	0.31	NA	0.13	0.62	34.85	2.11	4.14	0.39
2006	Babolsar	37	38325	0.64	NA	0.28	1.26	51.44	2.10	3.99	0.39
2006	Mahmudabad	38	20175	0.55	NA	0.23	1.11	31.96	2.05	4.15	0.39
2006	Neka	39	21961	0.49	NA	0.2	1.01	35.85	2.08	4.03	0.39
2006	Chalus	40	26818	0.41	NA	0.17	0.82	51.66	2.04	4.03	0.39
2006	Juybar	41	15538	0.56	NA	0.23	1.16	38.77	2.16	3.95	0.39
2006	Ahar	42	29668	0.35	NA	0.15	0.7	62.27	0.94	2.59	0.42
2006	Tabriz	43	339422	1	NA	0.45	1.94	93.15	0.92	2.60	0.42
2006	Sarab	44	26654	0.47	NA	0.2	0.95	42.10	1.00	2.66	0.42
2006	Maragheh	45	48111	0.44	NA	0.19	0.86	68.57	0.93	2.61	0.42
2006	Marand	46	50151	0.74	NA	0.32	1.44	58.54	0.96	2.62	0.42
2006	Miyaneh	47	39441	0.43	NA	0.18	0.85	49.46	0.96	2.68	0.42
2006	Hashtrud	48	12172	0.39	NA	0.16	0.81	28.96	0.95	2.64	0.42
2006	Bonab	49	25656	0.52	NA	0.22	1.04	60.96	0.92	2.65	0.42
2006	Bostanabad	50	17443	0.39	NA	0.16	0.79	19.24	0.95	2.81	0.42
2006	Shabestar	51	29270	0.42	NA	0.18	0.85	46.74	0.83	2.74	0.42
2006	Kalibar	52	16660	0.26	NA	0.1	0.56	14.22	0.97	2.62	0.42
2006	Haris	53	13214	0.5	NA	0.21	1.04	45.31	0.99	2.70	0.42
2006	Jolfa	54	12109	0.41	NA	0.16	0.87	60.44	0.94	2.65	0.42
2006	Malekan	55	19441	0.33	NA	0.13	0.68	31.95	0.93	2.76	0.42
2006	Azarshahr	56	21943	0.39	NA	0.16	0.81	59.76	0.92	2.68	0.42
2006	Osku	57	18524	0.33	NA	0.14	0.69	52.32	0.97	2.69	0.42
2006	Charoymaq	58	5594	0.25	NA	0.1	0.53	12.68	0.92	2.50	0.42
2006	Varzaqan	59	9216	0.28	NA	0.11	0.6	10.45	1.00	2.63	0.42
2006	Ajabshir	60	13773	0.4	NA	0.16	0.84	41.99	0.97	2.53	0.42
2006	Orumiyeh	61	173497	0.87	NA	0.39	1.68	70.48	1.14	2.46	0.38
2006	Piranshahr	62	17528	0.38	NA	0.16	0.78	51.28	1.15	2.31	0.38
2006	Khoy	63	70858	0.36	NA	0.16	0.7	65.81	1.22	2.42	0.38
2006	Sardasht	64	17306	0.25	NA	0.1	0.53	46.68	1.17	2.51	0.38
2006	Salmas	65	32427	0.6	NA	0.26	1.2	54.64	1.18	2.54	0.38
2006	Maku	66	30896	0.26	NA	0.11	0.53	49.33	1.16	2.38	0.38
2006	Mahabad	67	37990	0.36	NA	0.16	0.72	69.09	1.14	2.39	0.38
2006	Miyandoab	68	47485	0.4	NA	0.18	0.79	51.79	1.09	2.43	0.38
2006	Naqadeh	69	23463	0.53	NA	0.23	1.07	67.87	1.21	2.47	0.38
2006	Bukan	70	37787	0.33	NA	0.14	0.66	72.83	1.08	2.37	0.38
2006	Shahindezh	71	17829	0.37	NA	0.15	0.77	50.27	1.17	2.36	0.38
2006	Takab	72	15803	0.3	NA	0.12	0.63	54.81	1.12	2.48	0.38
2006	Oshnaviyeh	73	11024	0.67	NA	0.27	1.36	54.00	1.19	2.37	0.38
2006	Chaldoran	74	7392	0.33	NA	0.11	0.75	34.88	1.24	2.42	0.38
2006	Eslamabade Gharb	75	39314	0.32	NA	0.14	0.64	51.87	0.72	2.83	0.39

2006	Kermanshah	76	193732	1.02	NA	0.46	1.98	84.32	0.66	2.65	0.39
2006	Paveh	77	11165	0.44	NA	0.18	0.91	49.98	0.62	2.63	0.39
2006	Sarpole Zahab	78	15355	0.44	NA	0.18	0.91	40.86	0.68	2.61	0.39
2006	Sonqor	79	20309	0.47	NA	0.2	0.93	47.83	0.66	2.64	0.39
2006	Qasreshirin	80	4327	0.67	NA	0.25	1.44	69.14	0.72	2.63	0.39
2006	Kangavar	81	16158	0.52	NA	0.22	1.05	61.63	0.78	2.52	0.39
2006	Gilanegharb	82	12897	0.34	NA	0.13	0.7	33.93	0.66	2.75	0.39
2006	Javanrud	83	18855	0.36	NA	0.14	0.73	54.58	0.70	2.61	0.39
2006	Sahneh	84	16106	0.46	NA	0.19	0.92	43.04	0.65	2.62	0.39
2006	Harsin	85	16666	0.46	NA	0.19	0.93	64.60	0.69	2.59	0.39
2006	Salas-e-Babajani	86	5811	0.29	NA	0.09	0.7	21.96	0.66	2.71	0.39
2006	Abadan	87	50911	1.51	NA	0.66	2.93	83.95	1.65	3.24	0.52
2006	Andimeshk	88	27960	0.68	NA	0.3	1.36	77.85	1.54	3.07	0.52
2006	Ahvaz	89	229415	1.51	NA	0.67	2.93	82.70	1.54	3.09	0.52
2006	Izeh	90	30756	0.43	NA	0.18	0.87	53.78	1.59	3.25	0.52
2006	Bandar-e-Mahshahr	91	40753	1.06	NA	0.47	2.08	94.14	1.54	3.17	0.52
2006	Behbahan	92	33790	0.9	NA	0.39	1.79	72.00	1.57	3.28	0.52
2006	Khorramshahr	93	27272	1.01	NA	0.44	1.99	83.53	1.61	3.17	0.52
2006	Dezful	94	67746	0.82	NA	0.36	1.6	73.90	1.65	3.11	0.52
2006	Dashte Azadegan	95	18855	0.72	NA	0.31	1.44	56.74	1.57	3.17	0.52
2006	Ramhormoz	96	29247	0.72	NA	0.31	1.41	53.34	1.57	3.20	0.52
2006	Shadegan	97	20126	0.73	NA	0.31	1.46	39.29	1.62	3.26	0.52
2006	Shushtar	98	40460	0.82	NA	0.36	1.6	61.01	1.58	3.24	0.52
2006	Masjedsoleyman	99	29560	0.66	NA	0.28	1.31	67.07	1.54	3.30	0.52
2006	Shush	100	29886	0.7	NA	0.31	1.37	46.20	1.61	3.27	0.52
2006	Baghmalek	101	15517	0.43	NA	0.18	0.88	35.96	1.59	3.18	0.52
2006	Omidiyeh	102	14724	0.77	NA	0.33	1.56	69.07	1.56	3.35	0.52
2006	Lali	103	5479	0.58	NA	0.21	1.27	47.26	1.51	3.28	0.52
2006	Hendijan	104	6606	0.92	NA	0.36	1.92	70.24	1.59	3.25	0.52
2006	Abadeh	105	20308	0.99	NA	0.43	1.98	88.54	1.31	3.27	0.38
2006	Estahban	106	12703	0.67	NA	0.28	1.38	70.85	1.30	3.39	0.38
2006	Eqlid	107	19082	0.66	NA	0.28	1.3	62.99	1.30	3.31	0.38
2006	Jahrom	108	39104	0.74	NA	0.33	1.46	64.14	1.35	3.42	0.38
2006	Darab	109	30846	0.66	NA	0.29	1.31	42.30	1.34	3.47	0.38
2006	Sepidan	110	16276	0.41	NA	0.17	0.84	21.30	1.39	3.24	0.38
2006	Shiraz	111	341210	1.3	NA	0.58	2.53	87.90	1.34	3.24	0.38
2006	Fasa	112	37180	0.83	NA	0.36	1.63	58.20	1.30	3.35	0.38
2006	Firuzabad	113	22199	0.66	NA	0.29	1.3	57.07	1.33	3.35	0.38
2006	Kazerun	114	51497	0.57	NA	0.25	1.14	51.42	1.39	3.43	0.38
2006	Lar (Larestan)	115	48205	0.82	NA	0.36	1.61	58.27	1.33	3.19	0.38
2006	Marvdasht	116	58653	0.54	NA	0.24	1.06	45.77	1.34	3.39	0.38
2006	Mamasany	117	33781	0.37	NA	0.16	0.72	33.45	1.30	3.20	0.38
2006	Neyriz	118	19898	0.57	NA	0.25	1.13	58.99	1.31	3.28	0.38
2006	Lamard	119	13221	0.61	NA	0.25	1.23	43.62	1.35	3.29	0.38
2006	Bovanat	120	9239	0.44	NA	0.17	0.93	30.12	1.30	3.45	0.38
2006	Arsanjan	121	7410	0.83	NA	0.33	1.73	39.76	1.47	3.34	0.38
2006	Khorrambid	122	8538	0.56	NA	0.22	1.18	77.43	1.34	3.34	0.38

2006	Zarrindasht	123	9977	0.5	NA	0.18	1.09	56.40	1.29	3.21	0.38
2006	Qirokarzin	124	11478	0.42	NA	0.17	0.89	55.12	1.35	3.27	0.38
2006	Mohr	125	8467	0.47	NA	0.18	0.98	36.43	1.32	3.13	0.38
2006	Farashband	126	8533	0.53	NA	0.22	1.11	53.58	1.34	3.24	0.38
2006	Baft	127	27160	0.43	NA	0.18	0.86	36.13	0.70	3.57	0.56
2006	Bam	128	41536	0.4	NA	0.17	0.78	37.80	0.69	3.68	0.56
2006	Jiroft	129	37175	0.48	NA	0.21	0.95	41.37	0.67	3.84	0.56
2006	Rafsanjan	130	53382	0.85	NA	0.38	1.67	57.44	0.65	3.78	0.56
2006	Zarand	131	27280	0.63	NA	0.27	1.27	53.34	0.72	3.53	0.56
2006	Sirjan	132	44331	0.79	NA	0.35	1.57	75.39	0.67	3.54	0.56
2006	Shahrebabak	133	18922	0.64	NA	0.27	1.28	63.90	0.61	3.64	0.56
2006	Kerman	134	124560	1.08	NA	0.48	2.08	87.87	0.70	3.59	0.56
2006	Kahnuj	135	37504	0.3	NA	0.13	0.6	22.39	0.69	3.53	0.56
2006	Bardsir	136	15711	0.4	NA	0.16	0.83	55.21	0.65	3.54	0.56
2006	Ravar	137	7870	0.53	NA	0.2	1.13	58.01	0.70	3.73	0.56
2006	Anbarabad	138	11793	0.35	NA	0.12	0.81	32.61	0.75	3.67	0.56
2006	Manujan	139	9003	0.32	NA	0.11	0.72	30.64	0.69	3.74	0.56
2006	Taybad	140	22166	0.36	NA	0.15	0.74	46.03	0.84	2.96	0.50
2006	Torbate Heydarieh	141	59821	0.58	NA	0.26	1.12	46.85	0.76	3.01	0.50
2006	Torbate Jam	142	38086	0.51	NA	0.22	1.01	44.14	0.79	2.72	0.50
2006	Darrehgaz	143	15594	0.49	NA	0.2	1	55.35	0.82	2.87	0.50
2006	Sabzevar	144	88929	0.59	NA	0.26	1.15	49.92	0.82	2.81	0.50
2006	Quchan	145	35727	0.46	NA	0.2	0.92	50.64	0.77	2.92	0.50
2006	Kashmar	146	38782	0.52	NA	0.22	1.03	49.32	0.73	2.76	0.50
2006	Gonabad	147	23546	0.59	NA	0.25	1.19	49.43	0.87	2.93	0.50
2006	Mashhad	148	544020	0.98	NA	0.44	1.91	90.47	0.79	2.91	0.50
2006	Neyshabur	149	86504	0.53	NA	0.24	1.05	52.58	0.81	2.76	0.50
2006	Chenaran	150	20118	0.4	NA	0.17	0.8	40.42	0.86	2.98	0.50
2006	Khaf	151	17042	0.36	NA	0.15	0.74	45.22	0.86	2.94	0.50
2006	Sarakhs	152	13500	0.53	NA	0.22	1.09	40.83	0.71	2.96	0.50
2006	Fariman	153	15507	0.49	NA	0.21	0.99	53.89	0.83	2.90	0.50
2006	Bardeskan	154	13871	0.29	NA	0.12	0.62	36.87	0.77	2.75	0.50
2006	Rashtkhar	155	9722	0.3	NA	0.1	0.69	19.02	0.83	2.76	0.50
2006	Kalat	156	7466	0.52	NA	0.19	1.15	24.45	0.79	2.96	0.50
2006	Ardestan	157	10528	0.53	NA	0.21	1.09	55.52	1.92	3.78	0.33
2006	Isfahan	158	417819	1.56	NA	0.7	3.05	91.63	1.90	3.62	0.33
2006	Khomeynishahr	159	53531	0.77	NA	0.34	1.49	96.94	1.95	3.77	0.33
2006	Khansar	160	7914	0.52	NA	0.22	1.03	62.36	1.97	3.62	0.33
2006	Semirom	161	14125	0.51	NA	0.2	1.05	50.80	1.95	3.57	0.33
2006	Faridan	162	17389	0.74	NA	0.32	1.47	41.71	1.91	3.77	0.33
2006	Fereydunshahr	163	7719	0.43	NA	0.17	0.9	47.89	1.98	3.65	0.33
2006	Falavarjan	164	42844	0.67	NA	0.29	1.3	61.66	1.85	3.84	0.33
2006	Shahreza	165	38572	0.73	NA	0.32	1.46	79.69	1.92	3.65	0.33
2006	Kashan	166	63860	1.2	NA	0.54	2.33	85.73	1.88	3.59	0.33
2006	Golpayegan	167	19502	0.61	NA	0.26	1.22	73.82	1.92	3.76	0.33
2006	Lanjan	168	44331	0.9	NA	0.4	1.77	83.02	1.90	3.65	0.33
2006	Nayin	169	12730	0.58	NA	0.24	1.19	67.40	1.94	3.66	0.33

2006	Najafabad	170	55608	1.05	NA	0.47	2.06	91.78	1.91	3.67	0.33
2006	Natanz	171	10059	0.52	NA	0.22	1.05	55.30	1.82	3.63	0.33
2006	Shahinshahr va Meyme	172	57486	1.05	NA	0.47	2.06	90.06	1.97	3.64	0.33
2006	Mobarakeh	173	26224	0.62	NA	0.26	1.22	81.83	1.99	3.61	0.33
2006	Aran va Bidgol	174	18530	0.7	NA	0.31	1.39	85.49	1.99	3.64	0.33
2006	Tiran va Karvan	175	13601	0.4	NA	0.16	0.83	38.11	1.89	3.83	0.33
2006	Chadegan	176	6739	0.39	NA	0.15	0.84	39.76	1.92	3.69	0.33
2006	Iranshahr	177	34255	0.36	NA	0.15	0.73	42.98	-0.90	1.70	0.37
2006	Chah Bahar	178	32553	0.26	NA	0.11	0.53	32.90	-0.93	1.78	0.37
2006	Khash	179	19626	0.25	NA	0.1	0.53	32.04	-0.78	1.87	0.37
2006	Zabol	180	57936	0.28	NA	0.12	0.57	38.38	-0.87	1.78	0.37
2006	Zahedan	181	83510	0.58	NA	0.25	1.14	87.32	-0.87	1.95	0.37
2006	Saravan	182	35484	0.18	NA	0.07	0.37	36.29	-0.92	1.87	0.37
2006	Nikshahr	183	26276	0.19	NA	0.07	0.4	21.82	-0.84	1.80	0.37
2006	Sarbaz	184	18149	0.22	NA	0.06	0.56	10.96	-1.00	1.91	0.37
2006	Baneh	185	20382	0.4	NA	0.17	0.82	61.94	0.46	2.20	0.56
2006	Bijar	186	21202	0.33	NA	0.13	0.68	53.54	0.60	2.05	0.56
2006	Saqqez	187	39884	0.41	NA	0.17	0.81	64.94	0.54	2.16	0.56
2006	Sanandaj	188	81370	0.79	NA	0.35	1.53	81.83	0.66	1.98	0.56
2006	Qorveh	189	38754	0.38	NA	0.16	0.76	52.19	0.51	1.95	0.56
2006	Marivan	190	25872	0.52	NA	0.22	1.06	62.57	0.57	2.11	0.56
2006	Divandarreh	191	14802	0.31	NA	0.13	0.64	27.08	0.48	2.03	0.56
2006	Kamyaran	192	19207	0.32	NA	0.13	0.66	43.40	0.51	2.09	0.56
2006	Sarvabad	193	10238	0.23	NA	0.09	0.5	5.45	0.55	1.95	0.56
2006	Tuyserkan	194	24197	0.42	NA	0.18	0.84	44.95	1.08	2.88	0.33
2006	Malayer	195	58946	0.49	NA	0.22	0.96	57.80	1.05	2.68	0.33
2006	Nahavand	196	36357	0.37	NA	0.16	0.73	49.74	1.05	2.65	0.33
2006	Hamadan	197	130725	0.7	NA	0.31	1.37	81.94	1.08	2.77	0.33
2006	Kabudarahang	198	24752	0.27	NA	0.12	0.56	16.92	1.07	3.01	0.33
2006	Asadabad	199	20224	0.42	NA	0.18	0.82	48.05	1.05	2.83	0.33
2006	Bahar	200	23718	0.42	NA	0.18	0.83	42.75	1.04	2.61	0.33
2006	Razan	201	22376	0.33	NA	0.14	0.67	19.16	1.06	2.56	0.33
2006	Borujen	202	22953	0.43	NA	0.18	0.86	81.15	0.66	2.56	0.25
2006	Shahrekord	203	75820	0.63	NA	0.28	1.24	73.02	0.61	2.58	0.25
2006	Farsan	204	14941	0.4	NA	0.17	0.81	63.77	0.65	2.50	0.25
2006	Lordakan	205	26264	0.31	NA	0.13	0.62	19.85	0.62	2.46	0.25
2006	Ardal	206	8681	0.27	NA	0.09	0.63	15.29	0.56	2.35	0.25
2006	Kuhrang	207	5274	0.25	NA	0.09	0.55	6.79	0.61	2.63	0.25
2006	Aligudarz	208	23610	0.39	NA	0.16	0.79	59.05	1.02	2.92	0.27
2006	Borujerd	209	65934	0.82	NA	0.36	1.62	75.69	0.91	2.79	0.27
2006	Khorramabad	210	94150	0.84	NA	0.37	1.64	67.15	0.96	2.72	0.27
2006	Dalfan	211	20992	0.31	NA	0.13	0.63	41.73	1.06	2.87	0.27
2006	Dorud	212	28793	0.48	NA	0.2	0.94	66.11	0.94	2.89	0.27
2006	Kuhdasht	213	33943	0.29	NA	0.12	0.58	50.42	0.94	2.80	0.27
2006	Azna	214	14013	0.39	NA	0.16	0.81	53.46	0.94	2.73	0.27
2006	Poldokhtar	215	13306	0.44	NA	0.17	0.9	37.46	0.99	2.67	0.27
2006	Selseleh	216	12603	0.43	NA	0.14	1.04	40.35	0.95	2.66	0.27

2006	Ilam	217	35066	0.75	NA	0.33	1.47	81.85	1.07	2.62	0.28
2006	Darrehshahr	218	10204	0.28	NA	0.1	0.63	37.91	1.05	2.69	0.28
2006	Dehloran	219	10151	0.45	NA	0.17	0.96	63.99	1.08	2.72	0.28
2006	Shirvan va Chardavol	220	13261	0.3	NA	0.12	0.63	22.53	1.10	2.56	0.28
2006	Mehran	221	9501	0.56	NA	0.22	1.19	57.49	1.07	2.68	0.28
2006	Abdanan	222	8151	0.36	NA	0.15	0.76	59.07	1.02	2.58	0.28
2006	Eyvan	223	9369	0.7	NA	0.28	1.46	62.82	1.09	2.75	0.28
2006	Boyerahmad	224	32836	0.49	NA	0.21	0.98	42.55	0.72	2.65	0.19
2006	Kohgiluyeh	225	36864	0.26	NA	0.11	0.55	39.39	0.75	2.57	0.19
2006	Gachsaran	226	24896	0.52	NA	0.23	1.04	62.39	0.74	2.68	0.19
2006	Dena	227	9186	0.32	NA	0.12	0.66	19.41	0.71	2.79	0.19
2006	Bushehr	228	37207	1.68	NA	0.75	3.3	85.47	1.58	3.40	0.68
2006	Tangestan	229	12015	0.49	NA	0.2	1.02	23.01	1.66	3.12	0.68
2006	Dashtestan	230	42237	0.55	NA	0.24	1.09	61.74	1.49	3.04	0.68
2006	Dashti	231	13930	0.52	NA	0.21	1.08	55.60	1.56	3.14	0.68
2006	Dayyer	232	8275	0.53	NA	0.22	1.09	68.58	1.52	3.07	0.68
2006	Kangan	233	9792	0.59	NA	0.24	1.2	68.98	1.55	3.02	0.68
2006	Genaveh	234	15969	0.89	NA	0.38	1.79	73.71	1.59	3.19	0.68
2006	Deylam	235	5527	0.74	NA	0.27	1.62	72.75	1.54	2.95	0.68
2006	Jam	236	5781	0.37	NA	0.13	0.82	36.57	1.53	3.03	0.68
2006	Abhar	237	32795	0.38	NA	0.16	0.77	62.96	0.64	2.35	0.36
2006	Khodabandeh	238	29294	0.26	NA	0.11	0.54	25.97	0.68	2.33	0.36
2006	Zanjan	239	89188	0.66	NA	0.29	1.28	77.44	0.68	2.35	0.36
2006	Ijrud	240	7428	0.28	NA	0.1	0.6	6.98	0.76	2.53	0.36
2006	Khorramdarreh	241	12113	0.53	NA	0.22	1.09	78.75	0.64	2.39	0.36
2006	Tarom	242	8716	0.29	NA	0.1	0.66	13.08	0.65	2.37	0.36
2006	Mahneshan	243	7725	0.29	NA	0.1	0.66	13.78	0.74	2.54	0.36
2006	Damghan	244	17872	0.77	NA	0.33	1.52	67.35	2.64	4.87	0.37
2006	Semnan	245	37767	0.68	NA	0.3	1.33	88.83	2.71	4.92	0.37
2006	Shahrud	246	47662	0.99	NA	0.44	1.95	63.24	2.59	4.99	0.37
2006	Garmsar	247	16989	0.71	NA	0.31	1.44	67.21	2.60	4.93	0.37
2006	Ardakan	248	12852	0.68	NA	0.3	1.34	80.51	1.92	3.52	0.34
2006	Bafq	249	9543	0.7	NA	0.3	1.4	65.67	1.94	3.67	0.34
2006	Taft	250	11394	0.54	NA	0.22	1.1	32.06	1.90	3.78	0.34
2006	Mehriz	251	8940	0.85	NA	0.36	1.71	54.57	1.86	3.64	0.34
2006	Yazd	252	95026	1.68	NA	0.75	3.25	95.20	1.99	3.64	0.34
2006	Meybod	253	13097	0.8	NA	0.35	1.59	89.73	1.90	3.60	0.34
2006	Abarkuh	254	8738	0.71	NA	0.29	1.46	62.66	1.91	3.65	0.34
2006	Sadugh	255	5594	0.74	NA	0.27	1.64	52.96	1.85	3.61	0.34
2006	Khatam	256	6021	0.57	NA	0.22	1.24	54.60	1.91	3.65	0.34
2006	Tabas	257	12238	0.6	NA	0.25	1.24	54.87	1.88	3.60	0.34
2006	Abumusa	258	253	0.93	NA	0.33	2.09	98.33	0.46	2.44	0.26
2006	Bandarabbas	259	88529	0.74	NA	0.33	1.42	68.04	0.48	2.54	0.26
2006	Bandar-e Lengeh	260	23591	0.41	NA	0.19	0.78	46.88	0.41	2.42	0.26
2006	Qeshm	261	14936	0.45	NA	0.19	0.93	39.31	0.38	2.47	0.26
2006	Minab	262	40073	0.35	NA	0.15	0.7	23.92	0.44	2.58	0.26
2006	Bandar-e-Jask	263	10785	0.29	NA	0.13	0.56	15.84	0.46	2.56	0.26

2006	Rudan	264	16829	0.27	NA	0.1	0.56	34.27	0.41	2.63	0.26
2006	Hajiabad	265	11698	0.28	NA	0.11	0.6	30.54	0.47	2.48	0.26
2006	Bastak	266	11132	0.39	NA	0.16	0.8	23.49	0.58	2.48	0.26
2006	Tehran	267	1840903	1.16	NA	0.53	2.25	99.62	2.37	4.45	0.35
2006	Damavand	268	19271	0.83	NA	0.35	1.66	71.07	2.34	4.35	0.35
2006	Rey	269	49379	1.1	NA	0.48	2.19	26.45	2.37	4.38	0.35
2006	Shemiranat	270	8296	1.39	NA	0.59	2.77	55.34	2.36	4.33	0.35
2006	Varamin	271	96633	0.68	NA	0.3	1.34	79.67	2.44	4.16	0.35
2006	Shahriyar	272	180933	0.48	NA	0.21	0.93	84.66	2.37	4.32	0.35
2006	Eslamshahr	273	81593	0.47	NA	0.21	0.92	89.28	2.34	4.42	0.35
2006	Robatkarim	274	99671	0.3	NA	0.13	0.59	82.70	2.39	4.37	0.35
2006	Pakdasht	275	38772	0.36	NA	0.15	0.73	74.48	2.44	4.30	0.35
2006	Firuzkuh	276	8532	0.4	NA	0.15	0.85	37.64	2.37	4.26	0.35
2006	Ardebil	277	103690	0.47	NA	0.21	0.93	81.94	0.73	2.22	0.31
2006	Bilehsowar	278	10326	0.31	NA	0.12	0.66	36.61	0.77	2.02	0.31
2006	Khalkhal	279	19678	0.5	NA	0.17	1.13	44.27	0.73	2.24	0.31
2006	Meshginshahr	280	30861	0.55	NA	0.24	1.09	41.13	0.75	2.32	0.31
2006	Germi	281	17131	0.33	NA	0.13	0.68	29.78	0.79	2.24	0.31
2006	Parsabad	282	28599	0.29	NA	0.12	0.6	51.98	0.82	2.30	0.31
2006	Kowsar	283	5727	0.31	NA	0.09	0.78	24.65	0.77	2.25	0.31
2006	Namin	284	11582	0.34	NA	0.14	0.71	34.20	0.74	2.35	0.31
2006	Neer	285	5045	0.3	NA	0.1	0.69	23.30	0.76	2.26	0.31
2006	Qom	286	190607	0.77	NA	0.34	1.49	92.84	2.78	3.70	0.42
2006	Bueenzahra	287	29334	0.26	NA	0.11	0.53	33.97	1.56	3.24	0.37
2006	Takestan	288	31993	0.51	NA	0.22	1.01	56.73	1.58	3.24	0.37
2006	Qazvin	289	141522	0.83	NA	0.37	1.62	78.85	1.51	3.11	0.37
2006	Abyek	290	16599	0.46	NA	0.19	0.93	48.99	1.55	3.18	0.37
2006	Bandare Gaz	291	10607	0.64	NA	0.28	1.28	53.35	1.20	2.84	0.49
2006	Torkman	292	23816	0.54	NA	0.23	1.05	60.29	1.14	3.05	0.49
2006	Aliabad	293	23594	0.57	NA	0.24	1.15	47.22	1.16	2.71	0.49
2006	Kordkuy	294	15001	0.61	NA	0.27	1.19	48.10	1.13	2.87	0.49
2006	Gorgan	295	80758	1.03	NA	0.46	2.01	70.19	1.06	2.79	0.49
2006	Gonbade Kavus	296	53654	0.58	NA	0.26	1.15	48.79	1.15	2.81	0.49
2006	Minudasht	297	23790	0.39	NA	0.16	0.79	33.54	1.14	2.90	0.49
2006	Aqqala	298	19550	0.28	NA	0.11	0.59	29.55	1.12	2.80	0.49
2006	Kalaleh	299	25453	0.29	NA	0.12	0.59	22.77	1.21	2.84	0.49
2006	Azadshahr	300	16449	0.47	NA	0.2	0.96	54.20	1.18	2.89	0.49
2006	Ramyan	301	14784	0.28	NA	0.11	0.58	39.64	1.17	2.98	0.49
2006	Esfarayen	302	24282	0.28	NA	0.11	0.57	38.31	0.33	2.69	0.42
2006	Bojnurd	303	56989	0.48	NA	0.21	0.96	57.75	0.30	2.71	0.42
2006	Jajarm	304	11035	0.29	NA	0.1	0.66	60.80	0.25	2.90	0.42
2006	Shirvan	305	30398	0.37	NA	0.16	0.76	50.02	0.32	2.99	0.42
2006	Faruj	306	10682	0.24	NA	0.09	0.54	24.68	0.29	2.84	0.42
2006	Maneh va Semelqan	307	16235	0.33	NA	0.12	0.72	23.46	0.25	2.78	0.42
2006	Birjand	308	52853	0.72	NA	0.32	1.41	55.70	-0.38	2.70	0.20
2006	Sarbisheh	309	9733	0.38	NA	0.11	0.97	22.79	-0.50	2.68	0.20
2006	Qaenat	310	26870	0.33	NA	0.14	0.67	32.76	0.73	2.99	0.20

2006	Nehbandan	311	9820	0.29	NA	0.09	0.72	25.34	-0.39	2.85	0.20
2006	Sarayan	312	7260	0.46	NA	0.16	1.01	57.74	-0.61	2.73	0.20
2006	Ferdows	313	13933	0.45	NA	0.19	0.93	66.18	0.80	2.94	0.20
2006	Karaj	314	348685	0.49	NA	0.21	0.95	96.57	2.28	4.39	0.30
2006	Savojbolagh	315	42624	0.11	NA	0.04	0.26	35.95	2.40	4.37	0.30
2006	Nazarabad	316	23484	0.36	NA	0.14	0.74	78.41	2.38	4.20	0.30
2007	Arak	1	129511	0.95	0.97	0.74	1.19	79.75	1.71	3.49	0.52
2007	Ashtiyan	2	4464	0.62	0.42	0.26	1.22	33.99	1.70	3.61	0.52
2007	Tafresh	3	14028	0.45	0.4	0.22	0.79	27.07	1.67	3.54	0.52
2007	Khomeyn	4	25689	0.51	0.6	0.28	0.83	53.89	1.78	3.39	0.52
2007	Delijan	5	9834	0.77	0.81	0.39	1.33	68.04	1.75	3.44	0.52
2007	Saveh	6	45952	0.48	0.46	0.29	0.74	69.12	1.64	3.57	0.52
2007	Shazand	7	26800	0.46	0.52	0.25	0.75	29.64	1.70	3.37	0.52
2007	Mahalat	8	11319	0.52	0.17	0.26	0.92	80.34	1.73	3.49	0.52
2007	Zarandiyeh	9	12348	0.4	NA	0.16	0.83	56.29	1.69	3.41	0.52
2007	Komijan	10	8340	0.43	NA	0.16	0.94	39.10	1.74	3.48	0.52
2007	Astara	11	16767	0.46	0.36	0.23	0.8	62.08	1.29	4.03	0.43
2007	Astanehye Ashrafiyeh	12	28049	0.81	0.96	0.49	1.23	44.32	1.31	4.05	0.43
2007	Bandar Anzali	13	34403	1.07	1.11	0.71	1.53	82.56	1.29	4.00	0.43
2007	Tavalesh	14	37111	0.35	0.27	0.2	0.58	33.12	1.31	3.99	0.43
2007	Rasht	15	210254	1.16	1.17	0.97	1.37	69.24	1.29	4.05	0.43
2007	Rudbar	16	23405	0.53	0.42	0.29	0.86	50.82	1.26	4.24	0.43
2007	Rudsar	17	38906	0.65	0.54	0.41	0.96	42.59	1.27	4.14	0.43
2007	Sumehsara	18	32093	0.68	0.73	0.41	1.03	30.77	1.19	4.09	0.43
2007	Fuman	19	24707	0.35	0.08	0.18	0.58	25.11	1.20	4.00	0.43
2007	Langrud	20	34732	0.88	0.95	0.56	1.28	60.49	1.33	4.04	0.43
2007	Lahijan	21	42859	0.78	0.71	0.51	1.12	45.60	1.24	4.14	0.43
2007	Shaft	22	15807	0.49	0.61	0.25	0.84	12.21	1.25	3.94	0.43
2007	Amlash	23	11858	0.48	0.32	0.23	0.88	31.86	1.21	3.98	0.43
2007	Rezvanshahr	24	14542	0.43	0.27	0.21	0.76	28.83	1.34	4.04	0.43
2007	Siyahkal	25	12074	0.58	0.47	0.29	1	32.16	1.23	4.15	0.43
2007	Masal	26	11143	0.41	0.18	0.19	0.75	27.61	1.29	4.00	0.43
2007	Amol	27	79591	0.79	0.79	0.56	1.06	55.29	2.29	4.15	0.51
2007	Babol	28	109088	1.09	1.1	0.85	1.37	46.83	2.25	4.18	0.51
2007	Behshahr	29	43713	0.61	0.54	0.38	0.91	61.42	2.29	4.34	0.51
2007	Tonekabon	30	47173	0.86	0.96	0.58	1.22	47.22	2.21	4.27	0.51
2007	Ramsar	31	17139	1.19	1.46	0.7	1.81	71.67	2.26	4.19	0.51
2007	Sari	32	113049	1.17	1.22	0.92	1.47	51.65	2.18	4.15	0.51
2007	Savadkuh	33	16186	0.49	0.59	0.24	0.85	47.03	2.29	4.25	0.51
2007	Qaemshahr	34	69403	0.74	0.74	0.51	1.01	59.35	2.18	4.11	0.51
2007	Nur	35	24158	0.46	0.5	0.25	0.76	38.94	2.25	4.20	0.51
2007	Noshahr	36	26822	0.33	0.37	0.17	0.57	34.87	2.19	4.13	0.51
2007	Babolsar	37	39445	0.63	0.61	0.39	0.94	51.79	2.25	4.26	0.51
2007	Mahmudabad	38	20797	0.65	0.78	0.36	1.05	32.49	2.22	4.07	0.51
2007	Neka	39	22727	0.42	0.26	0.22	0.71	36.35	2.27	4.27	0.51
2007	Chalus	40	27643	0.32	0.14	0.17	0.55	51.66	2.23	4.23	0.51
2007	Juybar	41	15956	0.56	0.5	0.29	0.95	38.94	2.25	4.19	0.51

2007	Ahar	42	30466	0.38	0.39	0.2	0.63	62.71	1.16	2.65	0.56
2007	Tabriz	43	353928	1.51	1.52	1.33	1.69	93.11	1.17	2.94	0.56
2007	Sarab	44	27356	0.44	0.37	0.24	0.72	42.69	1.28	2.75	0.56
2007	Maragheh	45	49963	0.54	0.6	0.34	0.81	68.83	1.13	2.84	0.56
2007	Marand	46	51724	0.83	0.86	0.56	1.16	58.88	1.26	2.84	0.56
2007	Miyaneh	47	40168	0.47	0.49	0.28	0.73	50.26	1.18	2.88	0.56
2007	Hashtrud	48	12366	0.48	0.64	0.23	0.86	29.65	1.22	2.96	0.56
2007	Bonab	49	26476	0.62	0.7	0.35	0.98	61.06	1.14	3.01	0.56
2007	Bostanabad	50	17932	0.45	0.56	0.23	0.77	19.46	1.31	2.77	0.56
2007	Shabestar	51	29907	0.44	0.45	0.25	0.71	46.81	1.14	2.83	0.56
2007	Kalibar	52	17031	0.26	0.23	0.12	0.48	14.31	1.21	2.66	0.56
2007	Haris	53	13549	0.5	0.45	0.25	0.89	45.71	1.22	2.74	0.56
2007	Jolfa	54	12410	0.44	0.48	0.2	0.81	60.94	1.23	2.82	0.56
2007	Malekan	55	20155	0.38	0.51	0.19	0.67	31.82	1.14	2.63	0.56
2007	Azarshahr	56	22658	0.39	0.35	0.19	0.67	59.70	1.25	2.69	0.56
2007	Osku	57	19369	0.37	0.42	0.18	0.64	53.09	1.19	2.76	0.56
2007	Charoymaq	58	5701	0.27	NA	0.11	0.57	13.06	1.14	2.76	0.56
2007	Varzaqan	59	9388	0.3	NA	0.12	0.63	10.80	1.14	2.76	0.56
2007	Ajabshir	60	14035	0.51	0.71	0.25	0.9	42.14	1.21	2.77	0.56
2007	Orumiyeh	61	181090	1.36	1.41	1.14	1.6	71.20	1.40	2.58	0.47
2007	Piranshahr	62	18394	0.39	0.35	0.19	0.69	52.01	1.39	2.66	0.47
2007	Khoy	63	73625	0.5	0.57	0.33	0.72	66.11	1.44	2.54	0.47
2007	Sardasht	64	18013	0.25	0.23	0.12	0.47	47.71	1.32	2.48	0.47
2007	Salmas	65	33612	0.63	0.66	0.38	0.97	54.66	1.40	2.52	0.47
2007	Maku	66	31971	0.31	0.39	0.16	0.53	49.45	1.36	2.50	0.47
2007	Mahabad	67	39462	0.34	0.31	0.19	0.56	69.30	1.30	2.65	0.47
2007	Miyandoab	68	49300	0.37	0.33	0.21	0.57	51.94	1.37	2.53	0.47
2007	Naqadeh	69	24094	0.68	0.85	0.39	1.07	68.06	1.30	2.54	0.47
2007	Bukan	70	39630	0.29	0.21	0.16	0.48	73.50	1.39	2.66	0.47
2007	Shahindezh	71	18384	0.51	0.76	0.26	0.87	50.81	1.35	2.70	0.47
2007	Takab	72	16171	0.31	NA	0.13	0.64	55.19	1.40	2.50	0.47
2007	Oshnaviyeh	73	11518	0.64	0.54	0.32	1.13	53.94	1.40	2.55	0.47
2007	Chaldoran	74	7636	0.37	0.53	0.14	0.75	34.87	1.39	2.44	0.47
2007	Eslamabade Gharb	75	40349	0.33	0.35	0.19	0.54	52.48	0.88	2.66	0.53
2007	Kermanshah	76	201782	1.35	1.37	1.14	1.58	84.45	0.87	2.82	0.53
2007	Paveh	77	11451	0.6	1.04	0.29	1.06	50.46	0.89	2.61	0.53
2007	Sarpole Zahab	78	15840	0.4	0.26	0.2	0.73	40.91	0.98	2.75	0.53
2007	Sonqor	79	20643	0.42	0.28	0.21	0.7	48.31	0.88	2.76	0.53
2007	Qasreshirin	80	4444	0.74	0.92	0.32	1.42	70.09	0.88	2.75	0.53
2007	Kangavar	81	16639	0.53	0.48	0.27	0.89	62.12	0.92	2.75	0.53
2007	Gilanegharb	82	13222	0.31	0.15	0.14	0.58	34.40	0.89	2.75	0.53
2007	Javanrud	83	19647	0.33	0.21	0.16	0.59	55.57	0.89	2.73	0.53
2007	Sahneh	84	16526	0.44	0.36	0.22	0.76	43.64	0.96	2.75	0.53
2007	Harsin	85	17040	0.56	0.71	0.29	0.94	64.94	0.82	2.72	0.53
2007	Salas-e-Babajani	86	5941	0.32	NA	0.1	0.76	23.14	0.93	2.93	0.53
2007	Abadan	87	52057	1.32	1.27	0.95	1.75	84.34	1.74	3.29	0.64
2007	Andimeshk	88	29126	0.76	0.8	0.45	1.16	78.35	1.74	3.32	0.64

2007	Ahvaz	89	241197	1.65	1.65	1.43	1.89	83.00	1.71	3.30	0.64
2007	Izeh	90	32001	0.46	NA	0.2	0.91	54.71	1.69	3.25	0.64
2007	Bandar-e-Mahshahr	91	42972	0.83	0.7	0.53	1.2	94.01	1.73	3.38	0.64
2007	Behbahan	92	34942	1.15	1.27	0.76	1.61	72.20	1.69	3.27	0.64
2007	Khorramshahr	93	28160	0.9	0.82	0.55	1.36	83.43	1.71	3.23	0.64
2007	Dezful	94	70541	1.13	1.21	0.83	1.48	73.93	1.70	3.41	0.64
2007	Dashte Azadegan	95	19542	0.57	0.32	0.3	0.95	56.39	1.77	3.35	0.64
2007	Ramhormoz	96	30072	0.62	0.49	0.36	0.95	54.87	1.74	3.37	0.64
2007	Shadegan	97	21192	0.82	0.9	0.47	1.29	39.56	1.72	3.33	0.64
2007	Shushtar	98	41906	0.99	1.06	0.65	1.4	62.34	1.72	3.30	0.64
2007	Masjedsoleyman	99	29993	0.79	0.88	0.48	1.19	67.25	1.67	3.42	0.64
2007	Shush	100	31209	0.93	1.09	0.58	1.37	46.32	1.73	3.27	0.64
2007	Baghmalek	101	16108	0.4	0.26	0.2	0.71	36.44	1.72	3.45	0.64
2007	Omidiyeh	102	15280	0.69	0.54	0.37	1.15	69.11	1.64	3.37	0.64
2007	Lali	103	5649	0.56	0.36	0.23	1.1	47.77	1.69	3.21	0.64
2007	Hendijan	104	6834	0.86	0.6	0.41	1.56	70.49	1.69	3.27	0.64
2007	Abadeh	105	20987	1.02	1.03	0.62	1.57	88.37	1.49	3.30	0.51
2007	Estahban	106	13039	0.66	0.61	0.34	1.11	70.70	1.50	3.29	0.51
2007	Eqlid	107	19558	0.53	0.31	0.28	0.88	63.08	1.51	3.36	0.51
2007	Jahrom	108	40567	0.43	0.2	0.25	0.68	64.32	1.58	3.36	0.51
2007	Darab	109	32351	0.55	0.44	0.32	0.86	42.59	1.50	3.46	0.51
2007	Sepidan	110	16773	0.38	0.24	0.18	0.66	21.31	1.52	3.42	0.51
2007	Shiraz	111	357632	1.68	1.7	1.5	1.88	87.88	1.60	3.46	0.51
2007	Fasa	112	38627	0.83	0.83	0.53	1.21	58.72	1.52	3.31	0.51
2007	Firuzabad	113	23148	0.72	0.79	0.42	1.14	57.69	1.49	3.23	0.51
2007	Kazerun	114	52838	0.68	0.73	0.44	0.96	51.80	1.56	3.35	0.51
2007	Lar (Larestan)	115	50597	1.14	1.25	0.8	1.54	58.27	1.53	3.30	0.51
2007	Marvdasht	116	61095	0.64	0.68	0.42	0.92	46.23	1.52	3.46	0.51
2007	Mamasany	117	34714	0.37	0.35	0.2	0.6	34.30	1.56	3.32	0.51
2007	Neyriz	118	20685	0.67	0.79	0.37	1.09	59.25	1.53	3.35	0.51
2007	Lamard	119	13927	0.57	0.44	0.28	0.98	43.90	1.56	3.37	0.51
2007	Bovanat	120	9528	0.4	0.21	0.18	0.75	30.52	1.49	3.31	0.51
2007	Arsanjan	121	7712	0.89	NA	0.36	1.85	39.86	1.51	3.46	0.51
2007	Khorrambid	122	8876	0.61	0.69	0.28	1.11	77.88	1.45	3.46	0.51
2007	Zarrindasht	123	10490	0.45	0.19	0.2	0.86	56.80	1.52	3.32	0.51
2007	Qirokarzin	124	11920	0.42	0.35	0.19	0.77	55.52	1.47	3.29	0.51
2007	Mohr	125	8987	0.44	0.23	0.2	0.83	37.27	1.47	3.26	0.51
2007	Farashband	126	8737	0.54	0.47	0.25	0.99	54.62	1.51	3.31	0.51
2007	Baft	127	28076	0.4	0.35	0.21	0.66	35.56	0.81	3.65	0.64
2007	Bam	128	45592	0.32	0.23	0.18	0.51	38.21	0.86	3.60	0.64
2007	Jiroft	129	39787	0.47	0.47	0.27	0.75	41.18	0.74	3.83	0.64
2007	Rafsanjan	130	55658	0.7	0.62	0.45	0.99	57.37	0.76	3.91	0.64
2007	Zarand	131	28262	0.55	0.43	0.31	0.87	53.70	0.85	3.77	0.64
2007	Sirjan	132	46364	0.91	0.96	0.61	1.28	75.72	0.81	3.68	0.64
2007	Shahrebabak	133	19018	0.49	0.21	0.25	0.83	64.67	0.80	3.85	0.64
2007	Kerman	134	129751	1.1	1.1	0.87	1.36	87.76	0.77	3.74	0.64
2007	Kahnuj	135	39635	0.29	0.27	0.16	0.48	22.48	0.82	3.65	0.64

2007	Bardsir	136	15671	0.35	0.13	0.17	0.62	55.63	0.86	3.87	0.64
2007	Ravar	137	8071	0.48	0.25	0.21	0.93	57.44	0.80	3.70	0.64
2007	Anbarabad	138	12346	0.36	NA	0.13	0.83	32.09	0.73	3.70	0.64
2007	Manujan	139	9487	0.33	NA	0.12	0.73	30.42	0.75	3.71	0.64
2007	Taybad	140	23229	0.32	0.18	0.16	0.56	46.08	1.02	3.06	0.62
2007	Torbate Heydarieh	141	62048	0.61	0.62	0.4	0.86	47.16	1.08	3.09	0.62
2007	Torbate Jam	142	39719	0.43	0.32	0.24	0.67	44.49	0.98	3.12	0.62
2007	Darrehgaz	143	15893	0.47	0.37	0.24	0.81	55.73	1.06	3.11	0.62
2007	Sabzevar	144	91966	0.48	0.43	0.32	0.66	50.84	1.02	3.00	0.62
2007	Quchan	145	36531	0.43	0.38	0.24	0.68	51.48	1.07	3.06	0.62
2007	Kashmar	146	40153	0.42	0.3	0.24	0.66	49.73	1.06	3.20	0.62
2007	Gonabad	147	24049	0.66	0.73	0.38	1.05	49.61	1.08	3.00	0.62
2007	Mashhad	148	569084	1.46	1.47	1.32	1.6	90.49	1.05	2.94	0.62
2007	Neyshabur	149	89706	0.62	0.65	0.44	0.85	53.13	1.01	3.15	0.62
2007	Chenaran	150	21088	0.37	0.29	0.19	0.64	41.20	1.05	3.02	0.62
2007	Khaf	151	17768	0.4	0.47	0.2	0.71	45.30	1.01	3.14	0.62
2007	Sarakhs	152	13970	0.52	0.46	0.26	0.9	41.06	1.07	3.02	0.62
2007	Fariman	153	16084	0.52	0.51	0.26	0.89	54.37	1.04	3.18	0.62
2007	Bardeskan	154	14267	0.28	0.14	0.13	0.53	37.39	0.96	3.03	0.62
2007	Rashtkhar	155	10097	0.3	0.2	0.12	0.62	19.16	1.10	2.96	0.62
2007	Kalat	156	7576	0.65	1.04	0.28	1.24	24.77	1.01	3.19	0.62
2007	Ardestan	157	10585	0.54	0.53	0.27	0.97	56.48	2.10	3.85	0.48
2007	Isfahan	158	438054	1.46	1.46	1.31	1.63	91.77	2.12	3.84	0.48
2007	Khomeynishahr	159	56097	0.51	0.37	0.32	0.75	96.97	2.11	3.81	0.48
2007	Khansar	160	8039	0.48	0.23	0.23	0.86	62.49	2.10	3.69	0.48
2007	Semirom	161	14324	0.54	NA	0.22	1.09	51.06	2.20	3.63	0.48
2007	Faridan	162	17736	1.03	1.32	0.6	1.59	42.20	2.12	3.82	0.48
2007	Fereydunshahr	163	7911	0.42	0.25	0.19	0.79	48.07	2.10	3.82	0.48
2007	Falavarjan	164	44541	0.84	0.93	0.55	1.21	61.61	2.15	3.76	0.48
2007	Shahreza	165	39648	0.82	0.85	0.52	1.19	80.11	2.13	3.86	0.48
2007	Kashan	166	66007	1.37	1.4	1.03	1.77	86.09	2.05	3.78	0.48
2007	Golpayegan	167	19988	0.69	0.77	0.39	1.12	74.33	2.17	3.85	0.48
2007	Lanjan	168	46340	0.96	0.98	0.65	1.35	83.38	2.11	3.67	0.48
2007	Nayin	169	12990	0.51	0.29	0.26	0.89	67.87	2.18	3.75	0.48
2007	Najafabad	170	57726	0.82	0.75	0.56	1.14	91.82	2.13	3.80	0.48
2007	Natanz	171	10189	0.5	0.37	0.24	0.9	55.79	2.13	3.75	0.48
2007	Shahinshahr va Meyme	172	59972	1	0.97	0.7	1.34	90.25	2.13	3.90	0.48
2007	Mobarakeh	173	27213	0.58	0.53	0.33	0.92	81.82	2.13	3.75	0.48
2007	Aran va Bidgol	174	19168	0.57	0.32	0.31	0.94	85.36	2.11	3.96	0.48
2007	Tiran va Karvan	175	14045	0.42	NA	0.17	0.87	38.18	2.12	3.77	0.48
2007	Chadegan	176	6909	0.39	0.28	0.16	0.75	39.95	2.15	3.68	0.48
2007	Iranshahr	177	35809	0.35	0.3	0.19	0.57	43.06	-0.66	1.96	0.47
2007	Chah Bahar	178	35014	0.35	0.5	0.18	0.58	33.09	-0.62	1.76	0.47
2007	Khash	179	20085	0.31	0.43	0.14	0.56	32.47	-0.74	2.16	0.47
2007	Zabol	180	59601	0.37	0.45	0.22	0.57	38.94	-0.68	1.84	0.47
2007	Zahedan	181	86644	0.7	0.73	0.49	0.95	87.79	-0.61	2.02	0.47
2007	Saravan	182	37179	0.16	0.11	0.08	0.29	36.11	-0.63	1.76	0.47

2007	Nikshahr	183	27538	0.19	NA	0.08	0.4	21.61	-0.60	1.84	0.47
2007	Sarbaz	184	19124	0.23	NA	0.07	0.58	11.26	-0.70	1.88	0.47
2007	Baneh	185	21361	0.47	0.58	0.25	0.79	62.96	0.72	2.21	0.69
2007	Bijar	186	21514	0.32	0.27	0.16	0.56	53.96	0.74	2.19	0.69
2007	Saqqez	187	41134	0.51	0.59	0.3	0.78	65.31	0.72	2.25	0.69
2007	Sanandaj	188	84681	0.76	0.75	0.54	1.02	81.96	0.86	2.08	0.69
2007	Qorveh	189	39835	0.47	0.56	0.28	0.74	52.95	0.73	2.12	0.69
2007	Marivan	190	27035	0.42	0.24	0.23	0.69	63.61	0.84	2.17	0.69
2007	Divandarreh	191	15131	0.33	NA	0.14	0.68	27.80	0.84	2.07	0.69
2007	Kamyaran	192	19757	0.36	0.41	0.17	0.64	44.21	0.76	2.24	0.69
2007	Sarvabad	193	10411	0.24	0.19	0.1	0.49	5.72	0.74	2.30	0.69
2007	Tuyserkan	194	24584	0.38	0.31	0.2	0.64	45.22	1.27	2.81	0.46
2007	Malayer	195	60493	0.63	0.69	0.42	0.89	58.31	1.28	2.84	0.46
2007	Nahavand	196	37440	0.37	0.37	0.21	0.59	49.90	1.16	2.71	0.46
2007	Hamadan	197	136376	0.81	0.83	0.63	1.03	82.17	1.32	2.90	0.46
2007	Kabudarahang	198	25415	0.29	0.32	0.15	0.51	17.00	1.23	2.86	0.46
2007	Asadabad	199	20865	0.38	0.29	0.2	0.65	48.47	1.26	2.90	0.46
2007	Bahar	200	24414	0.4	0.33	0.21	0.67	42.71	1.26	2.92	0.46
2007	Razan	201	23035	0.43	0.61	0.23	0.73	19.47	1.29	2.88	0.46
2007	Borujen	202	23760	0.46	0.52	0.24	0.77	81.34	0.79	2.35	0.37
2007	Shahrekord	203	78469	0.63	0.63	0.43	0.86	73.29	0.79	2.51	0.37
2007	Farsan	204	15498	0.43	NA	0.18	0.87	63.86	0.79	2.74	0.37
2007	Lordakan	205	27480	0.29	0.23	0.15	0.5	20.14	0.90	2.63	0.37
2007	Ardal	206	8942	0.28	0.23	0.1	0.58	15.25	0.74	2.46	0.37
2007	Kuhrang	207	5407	0.26	NA	0.1	0.56	6.78	0.78	2.49	0.37
2007	Aligudarz	208	24424	0.47	0.58	0.25	0.78	60.09	1.14	3.03	0.41
2007	Borujerd	209	68625	0.93	0.95	0.67	1.23	75.85	1.17	2.95	0.41
2007	Khorramabad	210	97920	1	1.03	0.75	1.3	67.59	1.22	2.96	0.41
2007	Dalfan	211	21907	0.29	0.19	0.14	0.52	42.03	1.17	3.19	0.41
2007	Dorud	212	29820	0.46	0.42	0.26	0.73	66.10	1.09	2.88	0.41
2007	Kuhdasht	213	35504	0.29	0.24	0.15	0.48	50.71	1.29	3.05	0.41
2007	Azna	214	14374	0.42	0.42	0.2	0.74	53.99	1.22	2.98	0.41
2007	Poldokhtar	215	13794	0.46	0.46	0.22	0.83	38.10	1.22	2.99	0.41
2007	Selseleh	216	13096	0.47	NA	0.16	1.11	40.60	1.20	2.87	0.41
2007	Ilam	217	36707	1.05	1.22	0.69	1.5	81.91	1.33	2.79	0.42
2007	Darrehshahr	218	10664	0.31	NA	0.11	0.67	38.22	1.27	2.92	0.42
2007	Dehloran	219	10629	0.47	NA	0.19	1	64.27	1.29	2.74	0.42
2007	Shirvan va Chardavol	220	13627	0.29	0.15	0.13	0.54	22.91	1.26	2.78	0.42
2007	Mehran	221	9507	0.56	0.44	0.26	1.02	58.53	1.33	2.82	0.42
2007	Abdanan	222	8435	0.37	0.25	0.17	0.71	59.56	1.31	2.84	0.42
2007	Eyvan	223	9670	0.9	1.28	0.45	1.57	63.37	1.30	2.78	0.42
2007	Boyerahmad	224	34832	0.39	0.25	0.21	0.65	43.20	1.06	2.77	0.28
2007	Kohgiluyeh	225	37927	0.26	0.22	0.13	0.45	40.64	1.08	2.80	0.28
2007	Gachsaran	226	25876	0.55	0.56	0.31	0.87	63.42	1.10	2.77	0.28
2007	Dena	227	9480	0.33	NA	0.14	0.68	19.54	1.10	2.81	0.28
2007	Bushehr	228	39421	1.59	1.57	1.12	2.15	85.44	1.78	3.28	0.72
2007	Tangestan	229	12513	0.55	0.64	0.27	0.97	23.05	1.81	3.44	0.72

2007	Dashtestan	230	43610	0.82	0.98	0.53	1.18	62.04	1.68	3.34	0.72
2007	Dashti	231	14400	0.84	1.4	0.45	1.4	55.97	1.67	3.22	0.72
2007	Dayyer	232	8648	0.49	0.24	0.23	0.9	68.62	1.77	3.22	0.72
2007	Kangan	233	10781	0.71	0.99	0.36	1.25	68.80	1.78	3.27	0.72
2007	Genaveh	234	16589	0.84	0.75	0.46	1.35	73.76	1.77	3.37	0.72
2007	Deylam	235	5735	0.7	0.37	0.31	1.36	73.33	1.82	3.16	0.72
2007	Jam	236	6321	0.43	0.71	0.17	0.86	36.75	1.80	3.31	0.72
2007	Abhar	237	33894	0.41	0.42	0.23	0.66	63.75	0.95	2.67	0.46
2007	Khodabandeh	238	30362	0.31	0.4	0.16	0.53	26.27	0.81	2.48	0.46
2007	Zanjan	239	92987	0.7	0.7	0.49	0.93	77.96	0.79	2.63	0.46
2007	Ijrud	240	7624	0.3	NA	0.11	0.64	7.02	0.80	2.73	0.46
2007	Khorramdarreh	241	12572	0.57	NA	0.24	1.15	78.89	0.81	2.69	0.46
2007	Tarom	242	8964	0.31	NA	0.11	0.7	13.47	0.77	2.59	0.46
2007	Mahneshan	243	7831	0.3	0.26	0.11	0.61	14.41	0.76	2.50	0.46
2007	Damghan	244	18359	0.85	NA	0.37	1.65	67.79	2.76	5.26	0.49
2007	Semnan	245	39637	0.68	0.67	0.42	1.01	88.70	2.79	5.05	0.49
2007	Shahrud	246	49023	0.89	0.85	0.61	1.25	63.75	2.74	5.16	0.49
2007	Garmsar	247	17481	0.77	0.8	0.43	1.24	67.74	2.81	5.05	0.49
2007	Ardakan	248	13212	0.73	0.75	0.39	1.21	80.96	2.05	3.87	0.45
2007	Bafq	249	9968	0.7	0.62	0.35	1.22	65.78	2.04	3.75	0.45
2007	Taft	250	11450	0.49	0.32	0.24	0.87	32.38	2.06	3.74	0.45
2007	Mehriz	251	9112	0.82	0.64	0.43	1.41	55.38	2.09	3.99	0.45
2007	Yazd	252	99696	1.45	1.43	1.14	1.79	95.24	2.13	3.73	0.45
2007	Meybod	253	13631	0.74	0.59	0.39	1.24	89.79	2.11	3.84	0.45
2007	Abarkuh	254	9037	0.6	0.22	0.29	1.07	62.88	2.04	3.71	0.45
2007	Sadugh	255	5787	0.69	0.33	0.29	1.35	53.05	2.12	3.92	0.45
2007	Khatam	256	6232	0.6	NA	0.24	1.27	55.33	2.04	3.65	0.45
2007	Tabas	257	12619	0.54	0.32	0.27	0.94	55.31	2.10	3.84	0.45
2007	Abumusa	258	258	0.98	NA	0.36	2.18	98.42	0.49	2.53	0.32
2007	Bandarabbas	259	94106	1.08	1.15	0.81	1.38	68.43	0.42	2.65	0.32
2007	Bandar-e Lengeh	260	24821	0.37	0.26	0.21	0.62	47.22	0.46	2.71	0.32
2007	Qeshm	261	15740	0.45	0.41	0.23	0.79	39.55	0.45	2.65	0.32
2007	Minab	262	41818	0.39	0.45	0.22	0.63	24.44	0.56	2.57	0.32
2007	Bandar-e-Jask	263	11308	0.34	0.55	0.17	0.6	15.88	0.52	2.65	0.32
2007	Rudan	264	17676	0.24	0.12	0.11	0.44	34.29	0.50	2.62	0.32
2007	Hajiabad	265	12042	0.29	NA	0.12	0.62	31.01	0.53	2.50	0.32
2007	Bastak	266	11599	0.36	0.17	0.17	0.66	23.31	0.46	2.70	0.32
2007	Tehran	267	1911949	1.99	2	1.91	2.08	99.61	2.50	4.49	0.50
2007	Damavand	268	19888	0.9	NA	0.39	1.75	71.48	2.45	4.50	0.50
2007	Rey	269	51609	1.36	1.43	0.98	1.81	26.69	2.57	4.37	0.50
2007	Shemiranat	270	8820	1.46	1.54	0.8	2.37	54.82	2.50	4.26	0.50
2007	Varamin	271	100637	0.77	0.78	0.56	1.01	79.95	2.47	4.57	0.50
2007	Shahriyar	272	194544	0.32	0.29	0.23	0.44	85.11	2.46	4.38	0.50
2007	Eslamshahr	273	85611	0.41	0.37	0.26	0.58	89.39	2.55	4.59	0.50
2007	Robatkarim	274	105883	0.18	0.09	0.11	0.28	82.23	2.54	4.45	0.50
2007	Pakdasht	275	41340	0.38	NA	0.17	0.75	75.18	2.45	4.30	0.50
2007	Firuzkuh	276	8633	0.43	0.45	0.19	0.82	38.42	2.52	4.57	0.50

2007	Ardebil	277	107981	0.63	0.67	0.45	0.83	82.30	0.94	2.39	0.43
2007	Bilehsowar	278	10595	0.31	0.19	0.13	0.6	37.12	1.02	2.33	0.43
2007	Khalkhal	279	20068	0.54	NA	0.19	1.23	45.03	1.04	2.42	0.43
2007	Meshginshahr	280	31436	0.61	0.64	0.36	0.95	41.74	1.02	2.69	0.43
2007	Germi	281	17487	0.34	0.34	0.16	0.6	30.41	0.98	2.26	0.43
2007	Parsabad	282	29894	0.32	NA	0.13	0.63	52.82	0.94	2.54	0.43
2007	Kowsar	283	5768	0.34	NA	0.1	0.82	24.77	0.96	2.44	0.43
2007	Namin	284	11917	0.36	NA	0.15	0.74	34.55	0.94	2.35	0.43
2007	Neer	285	5145	0.32	NA	0.12	0.72	23.58	1.00	2.48	0.43
2007	Qom	286	199362	0.88	0.89	0.71	1.06	93.06	2.70	3.76	0.52
2007	Bueenzahra	287	30505	0.27	0.27	0.14	0.46	34.23	1.71	3.33	0.51
2007	Takestan	288	32956	0.55	0.56	0.32	0.85	57.58	1.68	3.34	0.51
2007	Qazvin	289	147521	0.66	0.63	0.5	0.84	79.24	1.75	3.37	0.51
2007	Abyek	290	17182	0.45	0.36	0.23	0.78	50.48	1.73	3.42	0.51
2007	Bandare Gaz	291	10837	0.54	0.18	0.27	0.92	53.42	1.26	2.97	0.57
2007	Torkman	292	24811	0.48	0.41	0.26	0.79	60.19	1.26	2.93	0.57
2007	Aliabad	293	24565	0.63	0.67	0.35	1.02	47.45	1.30	3.06	0.57
2007	Kordkuy	294	15423	0.78	1.02	0.43	1.27	48.82	1.32	2.98	0.57
2007	Gorgan	295	85046	1.07	1.08	0.8	1.39	70.95	1.36	2.78	0.57
2007	Gonbade Kavus	296	56299	0.82	0.92	0.55	1.15	48.91	1.34	2.97	0.57
2007	Minudasht	297	24616	0.46	0.58	0.24	0.76	33.71	1.42	2.86	0.57
2007	Aqqala	298	20437	0.27	0.2	0.13	0.5	29.61	1.35	2.97	0.57
2007	Kalaleh	299	26544	0.28	0.24	0.14	0.5	22.68	1.34	3.01	0.57
2007	Azadshahr	300	17025	0.45	0.36	0.23	0.78	54.29	1.35	3.01	0.57
2007	Ramyan	301	15298	0.31	0.4	0.14	0.57	39.77	1.29	3.02	0.57
2007	Esfarayen	302	25098	0.38	0.56	0.2	0.65	39.19	0.53	3.05	0.49
2007	Bojnurd	303	59583	0.35	0.24	0.21	0.54	57.92	0.63	3.03	0.49
2007	Jajarm	304	11248	0.3	NA	0.11	0.68	60.85	0.60	2.98	0.49
2007	Shirvan	305	31199	0.31	0.19	0.16	0.52	50.57	0.51	3.06	0.49
2007	Faruj	306	10919	0.24	0.17	0.1	0.47	24.79	0.53	2.78	0.49
2007	Maneh va Semelqan	307	16890	0.35	NA	0.13	0.73	23.77	0.57	2.96	0.49
2007	Birjand	308	54427	0.76	0.77	0.5	1.06	56.19	-0.21	2.82	0.27
2007	Sarbisheh	309	9957	0.4	NA	0.11	1.02	23.05	-0.15	2.78	0.27
2007	Qaenat	310	27698	0.34	0.36	0.18	0.59	33.23	1.05	2.90	0.27
2007	Nehbandan	311	9977	0.3	NA	0.1	0.72	26.12	-0.11	2.81	0.27
2007	Sarayan	312	7295	0.44	0.27	0.18	0.87	58.62	-0.15	2.73	0.27
2007	Ferdows	313	14231	0.49	0.55	0.25	0.87	66.65	1.05	3.07	0.27
2007	Karaj	314	371286	0.75	0.77	0.64	0.88	96.67	2.58	4.58	0.35
2007	Savojbolagh	315	44749	0.11	0.09	0.05	0.21	36.52	2.53	4.28	0.35
2007	Nazarabad	316	24596	0.39	NA	0.16	0.8	78.40	2.50	4.64	0.35
2008	Arak	1	135711	0.91	0.92	0.71	1.15	80.26	1.88	3.65	0.64
2008	Ashtiyan	2	4469	0.7	NA	0.27	1.5	35.40	1.88	3.62	0.64
2008	Tafresh	3	14217	0.42	0.26	0.21	0.74	27.81	1.84	3.50	0.64
2008	Khomeyn	4	26261	0.52	0.59	0.29	0.84	55.02	1.90	3.66	0.64
2008	Delijan	5	10170	0.74	0.59	0.38	1.29	69.12	1.88	3.76	0.64
2008	Saveh	6	47958	0.5	0.48	0.3	0.76	69.95	1.95	3.57	0.64
2008	Shazand	7	27378	0.35	0.22	0.18	0.59	30.04	1.92	3.59	0.64

2008	Mahalat	8	11719	0.58	0.34	0.29	1.01	81.07	1.94	3.67	0.64
2008	Zarandiyeh	9	12559	0.36	0.16	0.16	0.66	56.88	1.89	3.58	0.64
2008	Komijan	10	8593	0.45	NA	0.17	0.99	39.41	1.87	3.60	0.64
2008	Astara	11	17488	0.43	0.23	0.21	0.75	63.36	1.63	4.16	0.56
2008	Astanehye Ashrafiyeh	12	28459	0.63	0.61	0.37	0.97	45.02	1.51	4.16	0.56
2008	Bandar Anzali	13	35610	1.05	1.06	0.69	1.5	82.91	1.54	4.09	0.56
2008	Tavalesh	14	38547	0.6	0.74	0.36	0.9	34.15	1.56	4.08	0.56
2008	Rasht	15	218705	1.2	1.21	1.01	1.41	70.30	1.54	3.99	0.56
2008	Rudbar	16	23847	0.57	0.49	0.32	0.92	51.33	1.51	4.20	0.56
2008	Rudsar	17	39698	0.8	0.77	0.52	1.17	43.18	1.56	4.16	0.56
2008	Sumehsara	18	32697	0.44	0.24	0.25	0.71	31.51	1.57	4.23	0.56
2008	Fuman	19	25168	0.47	0.38	0.26	0.76	25.66	1.58	4.28	0.56
2008	Langrud	20	35649	0.88	0.92	0.56	1.27	61.02	1.54	4.25	0.56
2008	Lahijan	21	44074	0.8	0.74	0.53	1.14	47.54	1.55	4.17	0.56
2008	Shaft	22	16064	0.4	0.24	0.2	0.7	12.44	1.57	4.29	0.56
2008	Amlash	23	12050	0.58	NA	0.24	1.19	32.58	1.54	4.25	0.56
2008	Rezvanshahr	24	14996	0.56	0.66	0.28	0.97	29.35	1.56	4.25	0.56
2008	Siyahkal	25	12310	0.69	0.77	0.36	1.16	33.31	1.56	4.26	0.56
2008	Masal	26	11584	0.5	NA	0.21	1.03	28.44	1.49	4.17	0.56
2008	Amol	27	82779	1.04	1.1	0.77	1.35	55.94	2.40	4.41	0.62
2008	Babol	28	112714	2.1	2.2	1.75	2.48	47.42	2.38	4.39	0.62
2008	Behshahr	29	44777	0.71	0.7	0.46	1.03	62.18	2.37	4.24	0.62
2008	Tonekabon	30	48560	0.93	1.04	0.64	1.3	47.30	2.38	4.30	0.62
2008	Ramsar	31	17571	1.06	1.2	0.62	1.63	72.20	2.39	4.21	0.62
2008	Sari	32	117955	1.29	1.33	1.03	1.59	52.48	2.38	4.50	0.62
2008	Savadkuh	33	16321	0.36	0.12	0.17	0.64	46.95	2.37	4.37	0.62
2008	Qaemshahr	34	72320	0.72	0.71	0.5	0.98	60.01	2.46	4.35	0.62
2008	Nur	35	24898	0.54	0.64	0.3	0.86	39.32	2.33	4.47	0.62
2008	Noshahr	36	27967	0.34	0.36	0.17	0.58	34.87	2.37	4.37	0.62
2008	Babolsar	37	40644	0.86	0.97	0.57	1.24	52.14	2.39	4.18	0.62
2008	Mahmudabad	38	21456	0.65	0.75	0.36	1.06	33.03	2.34	4.31	0.62
2008	Neka	39	23538	0.45	0.34	0.24	0.75	36.88	2.43	4.27	0.62
2008	Chalus	40	28507	0.49	0.56	0.28	0.79	51.67	2.34	4.41	0.62
2008	Juybar	41	16399	0.57	0.48	0.29	0.97	39.10	2.30	4.48	0.62
2008	Ahar	42	31295	0.38	0.38	0.2	0.62	63.14	1.49	2.85	0.68
2008	Tabriz	43	369138	1.63	1.65	1.46	1.82	93.07	1.58	3.04	0.68
2008	Sarab	44	28090	0.54	0.57	0.31	0.85	43.27	1.43	2.94	0.68
2008	Maragheh	45	51903	0.54	0.58	0.34	0.79	69.09	1.44	3.14	0.68
2008	Marand	46	53356	0.66	0.6	0.43	0.94	59.21	1.45	2.81	0.68
2008	Miyaneh	47	40934	0.58	0.67	0.35	0.87	51.05	1.41	2.97	0.68
2008	Hashtrud	48	12571	0.41	0.32	0.2	0.74	30.37	1.42	3.04	0.68
2008	Bonab	49	27329	0.65	0.75	0.37	1.02	61.17	1.43	2.98	0.68
2008	Bostanabad	50	18445	0.46	0.54	0.24	0.79	19.70	1.46	3.02	0.68
2008	Shabestar	51	30560	0.39	0.32	0.22	0.63	46.87	1.45	2.99	0.68
2008	Kalibar	52	17433	0.32	0.45	0.15	0.58	14.42	1.48	2.84	0.68
2008	Haris	53	13910	0.55	0.59	0.28	0.97	46.10	1.43	2.85	0.68
2008	Jolfa	54	12724	0.42	0.31	0.19	0.78	61.43	1.40	3.01	0.68

2008	Malekan	55	20903	0.28	0.1	0.14	0.5	31.68	1.49	2.92	0.68
2008	Azarshahr	56	23404	0.42	0.43	0.22	0.72	59.64	1.47	2.87	0.68
2008	Osku	57	20274	0.38	0.4	0.19	0.67	53.85	1.42	3.13	0.68
2008	Charoymaq	58	5815	0.28	0.35	0.12	0.56	13.53	1.51	2.86	0.68
2008	Varzaqan	59	9568	0.29	0.2	0.13	0.57	11.18	1.49	2.81	0.68
2008	Ajabshir	60	14310	0.47	0.56	0.23	0.83	42.27	1.46	2.86	0.68
2008	Orumiyeh	61	189151	0.89	0.89	0.72	1.09	71.91	1.57	2.64	0.55
2008	Piranshahr	62	19325	0.46	0.55	0.23	0.78	52.74	1.62	2.73	0.55
2008	Khoy	63	76536	0.34	0.31	0.21	0.5	66.40	1.58	2.87	0.55
2008	Sardasht	64	18777	0.26	0.22	0.12	0.48	48.72	1.68	2.70	0.55
2008	Salmas	65	34854	0.48	0.35	0.27	0.75	54.70	1.61	2.60	0.55
2008	Maku	66	33106	0.21	0.06	0.1	0.37	49.57	1.53	2.66	0.55
2008	Mahabad	67	41017	0.32	0.25	0.18	0.51	69.52	1.63	2.57	0.55
2008	Miyandoab	68	51195	0.38	0.36	0.23	0.6	52.10	1.57	2.62	0.55
2008	Naqadeh	69	24754	0.5	0.41	0.27	0.82	68.23	1.65	2.83	0.55
2008	Bukan	70	41597	0.29	0.2	0.16	0.48	74.14	1.52	2.79	0.55
2008	Shahindezh	71	18965	0.37	0.32	0.18	0.65	51.36	1.55	2.54	0.55
2008	Takab	72	16554	0.32	NA	0.13	0.65	55.54	1.61	2.62	0.55
2008	Oshnaviyeh	73	12040	0.75	0.86	0.39	1.29	53.88	1.59	2.70	0.55
2008	Chaldoran	74	7891	0.36	NA	0.13	0.79	34.82	1.58	2.62	0.55
2008	Eslamabade Gharb	75	41451	0.28	0.19	0.15	0.45	53.09	1.12	2.76	0.65
2008	Kermanshah	76	210250	1.24	1.25	1.04	1.46	84.57	1.10	3.07	0.65
2008	Paveh	77	11765	0.56	0.84	0.28	1.01	50.92	1.07	2.77	0.65
2008	Sarpole Zahab	78	16352	0.4	0.25	0.19	0.72	40.94	1.10	2.70	0.65
2008	Sonqor	79	20998	0.49	0.46	0.26	0.8	48.80	1.06	2.89	0.65
2008	Qasreshirin	80	4570	0.77	0.9	0.34	1.49	71.01	1.09	2.96	0.65
2008	Kangavar	81	17142	0.66	0.81	0.36	1.09	62.63	1.06	2.87	0.65
2008	Gilanegharb	82	13568	0.31	0.15	0.14	0.58	34.87	1.08	2.78	0.65
2008	Javanrud	83	20504	0.43	0.5	0.22	0.73	56.55	1.12	2.86	0.65
2008	Sahneh	84	16966	0.45	0.35	0.23	0.77	44.25	1.09	2.89	0.65
2008	Harsin	85	17430	0.56	0.7	0.29	0.95	65.27	1.10	2.78	0.65
2008	Salas-e-Babajani	86	6088	0.33	NA	0.11	0.78	24.33	1.09	2.83	0.65
2008	Abadan	87	53256	2.04	2.13	1.55	2.58	84.71	1.82	3.37	0.73
2008	Andimeshk	88	30361	1	1.18	0.63	1.48	78.83	1.80	3.52	0.73
2008	Ahvaz	89	253701	2.16	2.19	1.92	2.43	83.30	1.79	3.31	0.73
2008	Izeh	90	33333	0.39	0.31	0.22	0.64	55.62	1.87	3.35	0.73
2008	Bandar-e-Mahshahr	91	45330	1.17	1.19	0.81	1.61	93.86	1.82	3.46	0.73
2008	Behbahan	92	36149	1.13	1.22	0.75	1.58	72.37	1.88	3.33	0.73
2008	Khorramshahr	93	29086	1.61	1.87	1.08	2.24	83.34	1.86	3.40	0.73
2008	Dezful	94	73489	1.23	1.32	0.93	1.59	73.96	1.82	3.48	0.73
2008	Dashte Azadegan	95	20272	0.78	0.83	0.44	1.25	56.05	1.81	3.31	0.73
2008	Ramhormoz	96	30991	0.92	1.01	0.57	1.36	56.39	1.74	3.43	0.73
2008	Shadegan	97	22326	0.85	0.95	0.5	1.33	39.82	1.83	3.39	0.73
2008	Shushtar	98	43490	0.87	0.87	0.57	1.24	63.62	1.78	3.26	0.73
2008	Masjedsoleyman	99	30464	0.61	0.54	0.35	0.95	67.40	1.81	3.40	0.73
2008	Shush	100	32606	0.92	1.05	0.58	1.37	46.45	1.87	3.41	0.73
2008	Baghmalek	101	16741	0.44	0.37	0.21	0.76	36.90	1.86	3.49	0.73

2008	Omidiyeh	102	15869	1.02	1.31	0.58	1.62	69.15	1.90	3.34	0.73
2008	Lali	103	5831	0.7	1.05	0.29	1.39	48.30	1.86	3.43	0.73
2008	Hendijan	104	7077	1.02	1.16	0.49	1.79	70.73	1.73	3.33	0.73
2008	Abadeh	105	21700	1.07	1.09	0.66	1.61	88.21	1.69	3.49	0.63
2008	Estahban	106	13391	0.72	0.74	0.38	1.21	70.58	1.71	3.57	0.63
2008	Eqlid	107	20058	0.89	1.11	0.51	1.39	63.16	1.64	3.55	0.63
2008	Jahrom	108	42105	0.79	0.8	0.51	1.14	64.51	1.70	3.51	0.63
2008	Darab	109	33958	0.65	0.6	0.39	0.99	42.87	1.69	3.70	0.63
2008	Sepidan	110	17293	0.38	0.24	0.19	0.68	21.31	1.76	3.55	0.63
2008	Shiraz	111	375070	1.66	1.67	1.48	1.85	87.87	1.63	3.40	0.63
2008	Fasa	112	40155	0.67	0.55	0.42	0.98	59.25	1.72	3.60	0.63
2008	Firuzabad	113	24150	0.61	0.51	0.35	0.99	58.30	1.65	3.61	0.63
2008	Kazerun	114	54243	0.64	0.67	0.42	0.93	52.18	1.82	3.58	0.63
2008	Lar (Larestan)	115	53162	0.99	1.03	0.69	1.35	58.26	1.59	3.51	0.63
2008	Marvdasht	116	63669	0.58	0.59	0.38	0.83	46.69	1.74	3.50	0.63
2008	Mamasany	117	35718	0.26	0.06	0.14	0.44	35.16	1.67	3.40	0.63
2008	Neyriz	118	21517	0.51	0.38	0.28	0.84	59.48	1.72	3.46	0.63
2008	Lamard	119	14686	0.57	0.42	0.29	0.99	44.15	1.69	3.44	0.63
2008	Bovanat	120	9829	0.47	NA	0.19	0.97	30.94	1.65	3.50	0.63
2008	Arsanjan	121	8032	0.91	NA	0.37	1.89	39.93	1.67	3.51	0.63
2008	Khorrambid	122	9235	0.5	0.22	0.23	0.93	78.31	1.71	3.33	0.63
2008	Zarrindasht	123	11048	0.56	NA	0.21	1.21	57.16	1.66	3.55	0.63
2008	Qirokarzin	124	12388	0.47	NA	0.19	0.97	55.97	1.68	3.48	0.63
2008	Mohr	125	9551	0.54	NA	0.22	1.13	38.10	1.69	3.71	0.63
2008	Farashband	126	8961	0.6	NA	0.25	1.24	55.63	1.71	3.47	0.63
2008	Baft	127	29076	0.35	0.2	0.18	0.57	34.98	0.98	3.83	0.71
2008	Bam	128	50123	0.42	0.42	0.25	0.64	38.59	0.86	3.83	0.71
2008	Jiroft	129	42620	0.49	0.49	0.29	0.76	40.98	0.97	4.05	0.71
2008	Rafsanjan	130	58071	0.72	0.67	0.49	1.01	57.30	0.90	3.98	0.71
2008	Zarand	131	29298	0.89	1.04	0.54	1.33	54.06	0.90	4.05	0.71
2008	Sirjan	132	48513	0.54	0.38	0.33	0.8	76.04	0.94	4.00	0.71
2008	Shahrebabak	133	19138	0.49	0.21	0.26	0.82	65.43	0.91	4.01	0.71
2008	Kerman	134	135230	1.16	1.17	0.93	1.43	87.64	0.87	3.82	0.71
2008	Kahnuj	135	41916	0.27	0.21	0.15	0.45	22.58	0.95	3.94	0.71
2008	Bardsir	136	15637	0.35	0.13	0.17	0.64	56.06	1.07	3.89	0.71
2008	Ravar	137	8288	0.56	NA	0.22	1.21	56.88	0.93	4.06	0.71
2008	Anbarabad	138	12943	0.37	NA	0.13	0.83	31.59	0.97	3.89	0.71
2008	Manujan	139	10005	0.32	0.21	0.13	0.63	30.22	1.03	4.00	0.71
2008	Taybad	140	24359	0.34	0.26	0.17	0.59	46.12	1.24	3.13	0.72
2008	Torbate Heydarieh	141	64417	0.49	0.44	0.32	0.72	47.47	1.23	3.07	0.72
2008	Torbate Jam	142	41445	0.61	0.66	0.38	0.91	44.85	1.27	3.15	0.72
2008	Darrehgaz	143	16218	0.6	0.72	0.31	1.01	56.08	1.27	3.25	0.72
2008	Sabzevar	144	95231	0.48	0.44	0.33	0.67	51.74	1.23	3.02	0.72
2008	Quchan	145	37378	0.48	0.48	0.28	0.75	52.32	1.24	3.19	0.72
2008	Kashmar	146	41623	0.57	0.58	0.34	0.86	50.13	1.31	3.18	0.72
2008	Gonabad	147	24595	0.75	0.88	0.44	1.17	49.77	1.26	3.19	0.72
2008	Mashhad	148	595536	1.4	1.41	1.26	1.53	90.51	1.25	3.37	0.72

2008	Neyshabur	149	93104	0.45	0.41	0.3	0.63	53.69	1.23	3.16	0.72
2008	Chenaran	150	22119	0.34	0.18	0.18	0.59	41.97	1.25	3.00	0.72
2008	Khaf	151	18535	0.31	0.11	0.15	0.56	45.37	1.23	3.18	0.72
2008	Sarakhs	152	14465	0.57	0.59	0.29	0.98	41.28	1.24	3.11	0.72
2008	Fariman	153	16693	0.41	0.12	0.2	0.72	54.84	1.25	3.22	0.72
2008	Bardeskan	154	14696	0.28	0.14	0.13	0.53	37.91	1.23	3.09	0.72
2008	Rashtkhar	155	10494	0.33	NA	0.12	0.75	19.34	1.27	3.15	0.72
2008	Kalat	156	7694	0.54	0.51	0.23	1.03	25.11	1.24	3.04	0.72
2008	Ardestan	157	10657	0.52	0.35	0.24	0.95	57.42	2.32	3.99	0.62
2008	Isfahan	158	459452	1.49	1.49	1.34	1.65	91.92	2.28	4.01	0.62
2008	Khomeynishahr	159	58806	0.74	0.71	0.49	1.04	96.99	2.34	3.97	0.62
2008	Khansar	160	8173	0.5	0.23	0.24	0.91	62.64	2.25	4.03	0.62
2008	Semirom	161	14530	0.44	0.14	0.21	0.78	51.31	2.38	3.88	0.62
2008	Faridan	162	18102	0.63	0.43	0.34	1.04	42.68	2.28	3.89	0.62
2008	Fereydunshahr	163	8114	0.43	0.24	0.2	0.8	48.27	2.33	3.94	0.62
2008	Falavarjan	164	46335	0.76	0.81	0.5	1.1	61.55	2.30	3.84	0.62
2008	Shahreza	165	40771	0.66	0.58	0.41	0.97	80.51	2.28	3.81	0.62
2008	Kashan	166	68251	1.03	0.97	0.75	1.36	86.44	2.30	3.92	0.62
2008	Golpayegan	167	20500	0.58	0.47	0.31	0.95	74.84	2.27	4.01	0.62
2008	Lanjan	168	48461	0.97	0.98	0.66	1.34	83.72	2.23	3.80	0.62
2008	Nayin	169	13267	0.52	0.29	0.27	0.9	68.31	2.36	3.88	0.62
2008	Najafabad	170	59940	1.26	1.3	0.92	1.65	91.85	2.29	3.97	0.62
2008	Natanz	171	10329	0.52	0.37	0.25	0.93	56.29	2.30	3.90	0.62
2008	Shahinshahr va Meyme	172	62608	0.92	0.86	0.65	1.24	90.42	2.23	3.94	0.62
2008	Mobarakeh	173	28246	0.62	0.58	0.36	0.98	81.82	2.26	3.92	0.62
2008	Aran va Bidgol	174	19841	0.65	0.52	0.37	1.05	85.23	2.29	4.01	0.62
2008	Tiran va Karvan	175	14513	0.36	0.14	0.17	0.67	38.26	2.31	4.08	0.62
2008	Chadegan	176	7089	0.41	0.28	0.17	0.78	40.11	2.33	4.14	0.62
2008	Iranshahr	177	37475	0.36	0.35	0.2	0.59	43.14	-0.41	1.88	0.57
2008	Chah Bahar	178	37713	0.28	0.29	0.14	0.48	33.30	-0.43	1.96	0.57
2008	Khash	179	20571	0.28	NA	0.11	0.57	32.90	-0.31	1.92	0.57
2008	Zabol	180	61380	0.37	0.44	0.22	0.57	39.51	-0.44	2.07	0.57
2008	Zahedan	181	89950	0.53	0.51	0.36	0.75	88.23	-0.42	1.95	0.57
2008	Saravan	182	38981	0.19	0.21	0.09	0.34	35.93	-0.46	2.08	0.57
2008	Nikshahr	183	28884	0.17	0.07	0.08	0.31	21.42	-0.35	1.99	0.57
2008	Sarbaz	184	20183	0.23	NA	0.07	0.59	11.62	-0.33	1.82	0.57
2008	Baneh	185	22410	0.47	0.55	0.25	0.79	63.95	0.98	2.18	0.78
2008	Bijar	186	21847	0.3	0.18	0.15	0.53	54.37	0.96	2.25	0.78
2008	Saqqez	187	42440	0.42	0.43	0.25	0.66	65.68	0.96	2.24	0.78
2008	Sanandaj	188	88159	1.14	1.2	0.87	1.46	82.07	0.96	2.46	0.78
2008	Qorveh	189	40988	0.58	0.74	0.36	0.88	53.70	1.00	2.15	0.78
2008	Marivan	190	28295	0.58	0.61	0.32	0.93	64.61	1.02	2.22	0.78
2008	Divandarreh	191	15484	0.33	0.26	0.16	0.59	28.57	1.08	2.44	0.78
2008	Kamyaran	192	20347	0.33	0.3	0.16	0.59	45.01	0.94	2.27	0.78
2008	Sarvabad	193	10601	0.27	0.37	0.12	0.52	6.03	0.92	2.22	0.78
2008	Tuyserkan	194	24986	0.7	0.99	0.4	1.1	45.49	1.49	3.03	0.58
2008	Malayer	195	62116	0.65	0.7	0.43	0.91	58.82	1.53	3.07	0.58

2008	Nahavand	196	38566	0.42	0.46	0.24	0.66	50.05	1.46	3.02	0.58
2008	Hamadan	197	142313	0.98	1.01	0.77	1.21	82.39	1.52	3.15	0.58
2008	Kabudarahang	198	26108	0.4	0.62	0.21	0.67	17.10	1.55	3.09	0.58
2008	Asadabad	199	21539	0.68	1.02	0.38	1.08	48.86	1.43	2.98	0.58
2008	Bahar	200	25137	0.6	0.8	0.34	0.96	42.68	1.55	2.97	0.58
2008	Razan	201	23735	0.35	0.34	0.18	0.59	19.78	1.48	2.74	0.58
2008	Borujen	202	24602	0.38	0.25	0.2	0.64	81.51	0.96	2.71	0.49
2008	Shahrekord	203	81237	0.91	0.98	0.66	1.21	73.55	1.00	2.62	0.49
2008	Farsan	204	16082	0.35	0.13	0.17	0.61	63.94	1.02	2.65	0.49
2008	Lordakan	205	28778	0.34	0.37	0.18	0.57	20.42	1.05	2.61	0.49
2008	Ardal	206	9222	0.29	NA	0.1	0.68	15.22	0.98	2.48	0.49
2008	Kuhrang	207	5547	0.28	0.37	0.11	0.56	6.79	1.01	2.73	0.49
2008	Aligudarz	208	25290	0.44	0.48	0.24	0.74	61.13	1.37	3.14	0.53
2008	Borujerd	209	71461	1.24	1.33	0.92	1.6	76.00	1.40	2.94	0.53
2008	Khorramabad	210	101901	0.71	0.68	0.52	0.95	68.02	1.36	3.14	0.53
2008	Dalfan	211	22876	0.37	0.46	0.19	0.64	42.33	1.37	2.98	0.53
2008	Dorud	212	30903	0.55	0.6	0.31	0.86	66.11	1.41	2.92	0.53
2008	Kuhdasht	213	37155	0.29	0.23	0.15	0.48	51.02	1.42	3.05	0.53
2008	Azna	214	14753	0.42	0.41	0.21	0.75	54.52	1.47	3.09	0.53
2008	Poldokhtar	215	14308	0.49	NA	0.2	1.01	38.72	1.43	2.98	0.53
2008	Selseleh	216	13629	0.48	NA	0.16	1.15	40.85	1.44	3.00	0.53
2008	Ilam	217	38454	0.72	0.66	0.44	1.06	81.96	1.45	3.01	0.56
2008	Darrehshahr	218	11155	0.32	NA	0.12	0.7	38.55	1.44	2.95	0.56
2008	Dehloran	219	11136	0.5	NA	0.2	1.07	64.57	1.52	3.02	0.56
2008	Shirvan va Chardavol	220	14024	0.34	NA	0.14	0.72	23.32	1.44	3.06	0.56
2008	Mehran	221	9548	0.69	0.87	0.33	1.25	59.55	1.53	3.07	0.56
2008	Abdanan	222	8737	0.37	0.24	0.17	0.7	60.06	1.47	2.84	0.56
2008	Eyvan	223	9991	0.99	1.44	0.5	1.69	63.90	1.45	2.99	0.56
2008	Boyerahmad	224	36983	0.56	0.6	0.32	0.87	43.85	1.24	3.04	0.40
2008	Kohgiluyeh	225	39093	0.21	0.05	0.1	0.36	41.89	1.39	2.90	0.40
2008	Gachsaran	226	26920	0.48	0.39	0.27	0.78	64.41	1.28	2.88	0.40
2008	Dena	227	9798	0.32	0.2	0.14	0.6	19.66	1.29	2.95	0.40
2008	Bushehr	228	41798	2.45	2.62	1.84	3.13	85.40	1.92	3.58	0.75
2008	Tangestan	229	13045	0.54	0.62	0.27	0.96	23.10	1.91	3.54	0.75
2008	Dashtestan	230	45058	0.36	0.18	0.21	0.57	62.34	1.96	3.40	0.75
2008	Dashti	231	14909	0.42	0.14	0.21	0.76	56.32	1.97	3.34	0.75
2008	Dayyer	232	9046	0.59	0.69	0.29	1.06	68.70	1.89	3.59	0.75
2008	Kangan	233	11887	0.54	0.36	0.26	0.96	68.60	1.90	3.31	0.75
2008	Genaveh	234	17253	1.17	1.45	0.7	1.83	73.78	1.90	3.41	0.75
2008	Deylam	235	5962	0.71	0.35	0.31	1.37	73.84	1.86	3.43	0.75
2008	Jam	236	6918	0.39	0.33	0.15	0.78	36.88	1.86	3.55	0.75
2008	Abhar	237	35050	0.38	0.35	0.21	0.62	64.50	1.03	2.78	0.54
2008	Khodabandeh	238	31490	0.23	0.13	0.12	0.4	26.58	0.92	2.74	0.54
2008	Zanjan	239	96988	0.6	0.57	0.41	0.81	78.46	0.99	2.69	0.54
2008	Ijrud	240	7833	0.3	NA	0.12	0.64	7.08	0.99	2.75	0.54
2008	Khorramdarreh	241	13055	0.46	0.16	0.22	0.82	79.01	0.97	2.82	0.54
2008	Tarom	242	9239	0.32	NA	0.11	0.71	13.85	0.96	2.81	0.54

2008	Mahneshan	243	7951	0.32	NA	0.11	0.72	15.04	0.99	2.70	0.54
2008	Damghan	244	18878	1.28	1.66	0.78	1.92	68.22	2.80	5.38	0.59
2008	Semnan	245	41638	1.17	1.37	0.8	1.62	88.54	2.86	5.35	0.59
2008	Shahrud	246	50447	1.18	1.21	0.83	1.59	64.26	2.94	5.43	0.59
2008	Garmsar	247	18008	0.68	0.55	0.37	1.09	68.24	2.94	5.28	0.59
2008	Ardakan	248	13597	0.55	0.15	0.28	0.94	81.42	2.19	4.08	0.56
2008	Bafq	249	10437	0.65	0.39	0.33	1.13	65.95	2.19	4.08	0.56
2008	Taft	250	11520	0.47	0.16	0.23	0.84	32.72	2.21	4.13	0.56
2008	Mehriz	251	9291	1.05	1.26	0.56	1.76	56.19	2.24	4.04	0.56
2008	Yazd	252	104639	1.82	1.83	1.49	2.2	95.26	2.26	4.01	0.56
2008	Meybod	253	14194	0.74	0.57	0.4	1.24	89.87	2.20	3.90	0.56
2008	Abarkuh	254	9352	0.74	0.64	0.37	1.29	63.09	2.21	4.14	0.56
2008	Sadugh	255	5995	0.83	NA	0.3	1.85	53.15	2.30	3.99	0.56
2008	Khatam	256	6455	0.58	0.31	0.26	1.09	56.03	2.25	4.12	0.56
2008	Tabas	257	13021	0.69	NA	0.29	1.38	55.77	2.22	4.14	0.56
2008	Abumusa	258	268	1.02	NA	0.38	2.28	98.51	0.49	2.92	0.39
2008	Bandarabbas	259	100116	1.12	1.19	0.86	1.42	68.80	0.46	2.86	0.39
2008	Bandar-e Lengeh	260	26141	0.68	0.98	0.41	1.04	47.54	0.51	2.84	0.39
2008	Qeshm	261	16609	0.53	0.64	0.27	0.91	39.77	0.51	2.77	0.39
2008	Minab	262	43680	0.37	0.38	0.21	0.59	24.97	0.49	2.60	0.39
2008	Bandar-e-Jask	263	11883	0.31	0.35	0.16	0.55	15.96	0.45	2.83	0.39
2008	Rudan	264	18575	0.24	0.11	0.11	0.46	34.30	0.48	2.84	0.39
2008	Hajiabad	265	12408	0.3	NA	0.12	0.63	31.51	0.57	2.59	0.39
2008	Bastak	266	12115	0.36	0.17	0.16	0.65	23.09	0.51	2.84	0.39
2008	Tehran	267	1986806	1.96	1.97	1.88	2.05	99.61	2.67	4.65	0.64
2008	Damavand	268	20541	0.89	0.89	0.51	1.38	71.86	2.63	4.55	0.64
2008	Rey	269	53971	1.3	1.32	0.94	1.73	26.93	2.70	4.71	0.64
2008	Shemiranat	270	9389	1.04	0.41	0.56	1.74	54.33	2.70	4.53	0.64
2008	Varamin	271	104854	0.91	0.94	0.68	1.17	80.22	2.67	4.60	0.64
2008	Shahriyar	272	209313	0.41	0.39	0.3	0.54	85.56	2.59	4.67	0.64
2008	Eslamshahr	273	89872	0.38	0.33	0.25	0.55	89.49	2.63	4.68	0.64
2008	Robatkarim	274	112588	0.27	0.24	0.17	0.4	81.75	2.67	4.62	0.64
2008	Pakdasht	275	44113	0.27	0.1	0.14	0.44	75.87	2.67	4.50	0.64
2008	Firuzkuh	276	8745	0.4	0.22	0.18	0.78	39.21	2.67	4.65	0.64
2008	Ardebil	277	112497	0.52	0.53	0.37	0.7	82.66	1.23	2.39	0.55
2008	Bilehsowar	278	10885	0.32	0.19	0.13	0.6	37.64	1.20	2.48	0.55
2008	Khalkhal	279	20485	0.55	NA	0.19	1.26	45.79	1.26	2.49	0.55
2008	Meshginshahr	280	32041	0.46	0.31	0.26	0.74	42.35	1.25	2.63	0.55
2008	Germi	281	17862	0.35	0.34	0.17	0.62	31.06	1.26	2.44	0.55
2008	Parsabad	282	31279	0.26	0.14	0.13	0.45	53.69	1.17	2.62	0.55
2008	Kowsar	283	5814	0.35	0.33	0.11	0.78	24.90	1.24	2.76	0.55
2008	Namin	284	12270	0.37	NA	0.15	0.76	34.91	1.29	2.49	0.55
2008	Neer	285	5251	0.34	NA	0.12	0.76	23.91	1.18	2.60	0.55
2008	Qom	286	208570	0.83	0.84	0.67	1.01	93.27	2.77	3.96	0.61
2008	Bueenzahra	287	31738	0.25	0.19	0.13	0.44	34.48	2.01	3.53	0.64
2008	Takestan	288	33970	0.61	0.66	0.36	0.94	58.42	1.93	3.38	0.64
2008	Qazvin	289	153837	1.01	1.03	0.81	1.25	79.63	1.99	3.53	0.64

2008	Abyek	290	17809	0.54	0.58	0.28	0.92	51.95	1.95	3.54	0.64
2008	Bandare Gaz	291	11084	0.58	0.35	0.29	1	53.50	1.42	2.99	0.64
2008	Torkman	292	25865	0.54	0.55	0.31	0.86	60.07	1.53	3.11	0.64
2008	Aliabad	293	25586	0.59	0.56	0.33	0.94	47.68	1.58	3.18	0.64
2008	Kordkuy	294	15865	0.54	0.37	0.28	0.91	49.52	1.54	3.08	0.64
2008	Gorgan	295	89605	1.41	1.46	1.09	1.77	71.68	1.63	3.16	0.64
2008	Gonbade Kavus	296	59086	0.72	0.78	0.48	1.02	49.02	1.53	3.22	0.64
2008	Minudasht	297	25481	0.4	0.4	0.21	0.67	33.91	1.48	3.03	0.64
2008	Aqqala	298	21379	0.25	0.1	0.12	0.46	29.67	1.58	3.17	0.64
2008	Kalaleh	299	27706	0.28	0.23	0.14	0.49	22.61	1.56	3.10	0.64
2008	Azadshahr	300	17627	0.38	0.12	0.19	0.67	54.40	1.57	3.12	0.64
2008	Ramyan	301	15838	0.26	0.13	0.12	0.49	39.88	1.58	3.13	0.64
2008	Esfarayen	302	25973	0.3	0.31	0.15	0.52	40.09	0.84	3.00	0.55
2008	Bojnurd	303	62317	0.67	0.75	0.44	0.95	58.09	0.75	2.83	0.55
2008	Jajarm	304	11548	0.28	0.17	0.11	0.57	61.14	0.83	3.09	0.55
2008	Shirvan	305	32045	0.55	0.74	0.31	0.86	51.12	0.76	3.12	0.55
2008	Faruj	306	11193	0.26	NA	0.1	0.57	24.90	0.75	3.15	0.55
2008	Maneh va Semelqan	307	17582	0.35	0.35	0.16	0.64	24.08	0.83	3.08	0.55
2008	Birjand	308	56105	0.79	0.81	0.54	1.1	56.67	0.13	2.77	0.34
2008	Sarbisheh	309	10193	0.4	NA	0.11	1.01	23.30	0.12	2.77	0.34
2008	Qaenat	310	28577	0.35	0.35	0.18	0.59	33.72	1.26	3.23	0.34
2008	Nehbandan	311	10149	0.31	NA	0.1	0.75	26.95	0.14	2.95	0.34
2008	Sarayan	312	7344	0.46	0.27	0.19	0.89	59.51	0.05	3.03	0.34
2008	Ferdows	313	14554	0.42	0.27	0.21	0.74	67.13	1.26	3.15	0.34
2008	Karaj	314	395471	1.19	1.23	1.05	1.35	96.77	2.62	4.49	0.39
2008	Savojbolagh	315	47012	0.1	0.04	0.04	0.2	37.10	2.68	4.66	0.39
2008	Nazarabad	316	25764	0.28	0.08	0.14	0.51	78.40	2.62	4.64	0.39
2009	Arak	1	142286	1.7	1.77	1.42	2.01	80.75	2.06	3.78	0.75
2009	Ashtiyan	2	4484	0.69	0.42	0.29	1.34	36.86	2.13	3.70	0.75
2009	Tafresh	3	14425	0.71	1.03	0.37	1.17	28.58	2.15	3.80	0.75
2009	Khomeyn	4	26875	0.69	0.86	0.4	1.06	56.13	2.00	3.78	0.75
2009	Delijan	5	10532	0.96	1.14	0.5	1.61	70.14	2.08	3.71	0.75
2009	Saveh	6	50077	0.56	0.55	0.35	0.83	70.77	2.05	3.74	0.75
2009	Shazand	7	27979	0.51	0.57	0.28	0.82	30.44	2.05	3.77	0.75
2009	Mahalat	8	12146	0.89	1.13	0.47	1.49	81.76	2.06	3.79	0.75
2009	Zarandiyeh	9	12783	0.46	NA	0.19	0.95	57.49	2.02	3.79	0.75
2009	Komijan	10	8861	0.46	0.45	0.2	0.9	39.72	2.01	3.67	0.75
2009	Astara	11	18261	0.79	1.11	0.43	1.29	64.62	1.75	4.40	0.66
2009	Astanehye Ashrafiyeh	12	28906	0.46	0.2	0.25	0.74	45.73	1.81	4.37	0.66
2009	Bandar Anzali	13	36889	0.98	0.92	0.65	1.4	83.25	1.87	4.35	0.66
2009	Tavalesh	14	40076	0.33	0.15	0.18	0.53	35.19	1.79	4.48	0.66
2009	Rasht	15	227709	1.07	1.06	0.89	1.26	71.33	1.76	4.34	0.66
2009	Rudbar	16	24311	0.6	0.48	0.34	0.96	51.84	1.81	4.50	0.66
2009	Rudsar	17	40543	1.18	1.26	0.81	1.62	43.78	1.78	4.42	0.66
2009	Sumehsara	18	33346	0.7	0.69	0.43	1.05	32.25	1.83	4.38	0.66
2009	Fuman	19	25666	0.62	0.67	0.36	0.98	26.23	1.83	4.40	0.66
2009	Langrud	20	36621	0.68	0.58	0.43	1.03	61.56	1.83	4.43	0.66

2009	Lahijan	21	45416	1.04	1.05	0.73	1.43	49.49	1.88	4.15	0.66
2009	Shaft	22	16333	0.51	NA	0.22	1.02	12.68	1.89	4.41	0.66
2009	Amlash	23	12259	0.48	0.15	0.23	0.85	33.31	1.77	4.37	0.66
2009	Rezvanshahr	24	15478	0.5	0.38	0.24	0.86	29.89	1.81	4.26	0.66
2009	Siyahkal	25	12571	0.67	0.6	0.35	1.14	34.47	1.81	4.43	0.66
2009	Masal	26	12054	0.52	0.49	0.25	0.92	29.31	1.91	4.32	0.66
2009	Amol	27	86142	1	1.02	0.75	1.3	56.59	2.50	4.58	0.71
2009	Babol	28	116537	1.39	1.42	1.13	1.71	48.01	2.56	4.55	0.71
2009	Behshahr	29	45904	1	1.06	0.68	1.39	62.94	2.50	4.56	0.71
2009	Tonekabon	30	50030	0.73	0.73	0.49	1.04	47.37	2.47	4.50	0.71
2009	Ramsar	31	18030	0.94	0.84	0.54	1.48	72.72	2.58	4.60	0.71
2009	Sari	32	123174	1.42	1.47	1.15	1.72	53.34	2.48	4.59	0.71
2009	Savadkuh	33	16464	0.41	0.23	0.2	0.72	46.87	2.49	4.54	0.71
2009	Qaemshahr	34	75420	0.89	0.91	0.64	1.18	60.65	2.54	4.39	0.71
2009	Nur	35	25668	0.62	0.78	0.35	0.99	39.70	2.48	4.60	0.71
2009	Noshahr	36	29177	0.4	0.48	0.21	0.65	34.86	2.54	4.47	0.71
2009	Babolsar	37	41925	0.93	1.03	0.62	1.32	52.48	2.48	4.53	0.71
2009	Mahmudabad	38	22162	0.59	0.54	0.32	0.97	33.58	2.47	4.59	0.71
2009	Neka	39	24390	0.5	0.41	0.27	0.82	37.40	2.54	4.44	0.71
2009	Chalus	40	29412	0.57	0.67	0.33	0.89	51.68	2.54	4.61	0.71
2009	Juybar	41	16869	0.59	0.47	0.31	1	39.24	2.55	4.66	0.71
2009	Ahar	42	32158	0.37	0.31	0.2	0.61	63.56	1.68	3.14	0.77
2009	Tabriz	43	385092	1.45	1.46	1.29	1.62	93.03	1.69	3.24	0.77
2009	Sarab	44	28862	0.66	0.76	0.38	1.01	43.84	1.65	3.10	0.77
2009	Maragheh	45	53939	0.56	0.59	0.36	0.82	69.35	1.72	3.04	0.77
2009	Marand	46	55048	0.59	0.47	0.37	0.85	59.54	1.73	3.00	0.77
2009	Miyaneh	47	41738	0.56	0.61	0.34	0.85	51.84	1.62	2.98	0.77
2009	Hashtrud	48	12791	0.43	0.31	0.21	0.78	31.11	1.78	3.10	0.77
2009	Bonab	49	28227	0.52	0.44	0.29	0.83	61.27	1.75	3.05	0.77
2009	Bostanabad	50	18980	0.55	0.74	0.29	0.91	19.94	1.68	3.17	0.77
2009	Shabestar	51	31234	0.48	0.49	0.27	0.76	46.93	1.78	3.00	0.77
2009	Kalibar	52	17862	0.29	0.22	0.13	0.53	14.54	1.72	3.18	0.77
2009	Haris	53	14298	0.73	1	0.37	1.22	46.50	1.65	2.99	0.77
2009	Jolfa	54	13051	0.49	NA	0.19	1.03	61.92	1.76	2.95	0.77
2009	Malekan	55	21683	0.44	0.56	0.22	0.75	31.53	1.76	3.12	0.77
2009	Azarshahr	56	24185	0.43	0.41	0.23	0.73	59.59	1.70	3.08	0.77
2009	Osku	57	21233	0.33	0.19	0.17	0.58	54.62	1.73	3.07	0.77
2009	Charoymaq	58	5937	0.3	NA	0.12	0.64	14.03	1.70	3.03	0.77
2009	Varzaqan	59	9756	0.35	0.4	0.15	0.65	11.56	1.70	3.06	0.77
2009	Ajabshir	60	14601	0.39	0.14	0.18	0.71	42.40	1.70	3.25	0.77
2009	Orumiyeh	61	197717	1.04	1.04	0.86	1.24	72.61	1.83	2.81	0.62
2009	Piranshahr	62	20323	0.4	0.31	0.2	0.69	53.48	1.73	2.80	0.62
2009	Khoy	63	79599	0.35	0.33	0.22	0.52	66.70	1.86	2.74	0.62
2009	Sardasht	64	19599	0.3	NA	0.12	0.61	49.71	1.83	2.79	0.62
2009	Salmas	65	36162	0.66	0.67	0.4	1	54.73	1.89	2.76	0.62
2009	Maku	66	34308	0.25	0.18	0.13	0.44	49.67	1.80	2.82	0.62
2009	Mahabad	67	42655	0.37	0.33	0.21	0.59	69.73	1.85	2.79	0.62

2009	Miyandoab	68	53178	0.45	0.46	0.28	0.68	52.27	1.92	2.54	0.62
2009	Naqadeh	69	25448	0.47	0.32	0.25	0.77	68.38	1.79	2.77	0.62
2009	Bukan	70	43700	0.34	0.28	0.19	0.54	74.78	1.78	2.81	0.62
2009	Shahindezh	71	19571	0.35	0.2	0.17	0.62	51.91	1.81	2.70	0.62
2009	Takab	72	16953	0.28	0.12	0.13	0.52	55.91	1.80	2.76	0.62
2009	Oshnaviyeh	73	12591	0.71	0.66	0.36	1.23	53.80	1.86	2.72	0.62
2009	Chaldoran	74	8157	0.39	0.5	0.15	0.8	34.81	1.83	2.82	0.62
2009	Eslamabade Gharb	75	42619	0.25	0.09	0.13	0.41	53.71	1.38	3.02	0.75
2009	Kermanshah	76	219166	1.24	1.24	1.04	1.45	84.68	1.27	2.92	0.75
2009	Paveh	77	12107	0.42	0.16	0.2	0.78	51.35	1.31	2.92	0.75
2009	Sarpole Zahab	78	16889	0.39	0.12	0.2	0.7	40.97	1.38	3.04	0.75
2009	Sonqor	79	21376	0.62	0.72	0.34	1	49.28	1.31	2.92	0.75
2009	Qasreshirin	80	4704	0.81	0.87	0.35	1.54	71.92	1.33	3.01	0.75
2009	Kangavar	81	17667	0.64	0.68	0.35	1.04	63.11	1.20	3.01	0.75
2009	Gilanegharb	82	13940	0.4	NA	0.16	0.83	35.36	1.41	3.04	0.75
2009	Javanrud	83	21434	0.32	0.1	0.16	0.57	57.53	1.34	2.91	0.75
2009	Sahneh	84	17430	0.63	0.78	0.33	1.04	44.85	1.27	2.96	0.75
2009	Harsin	85	17834	0.44	0.23	0.22	0.75	65.59	1.25	3.17	0.75
2009	Salas-e-Babajani	86	6250	0.35	NA	0.12	0.83	25.58	1.28	2.94	0.75
2009	Abadan	87	54508	1.79	1.81	1.36	2.3	85.07	1.92	3.48	0.80
2009	Andimeshk	88	31674	1.16	1.39	0.75	1.66	79.31	2.01	3.46	0.80
2009	Ahvaz	89	266977	1.7	1.71	1.49	1.94	83.59	1.94	3.53	0.80
2009	Izeh	90	34759	0.43	0.36	0.24	0.68	56.52	1.94	3.43	0.80
2009	Bandar-e-Mahshahr	91	47834	1.21	1.22	0.84	1.66	93.72	1.97	3.63	0.80
2009	Behbahan	92	37411	0.98	0.96	0.64	1.38	72.53	1.95	3.50	0.80
2009	Khorramshahr	93	30056	1.11	1.11	0.71	1.6	83.25	1.96	3.41	0.80
2009	Dezful	94	76604	1.11	1.16	0.82	1.44	73.98	2.04	3.51	0.80
2009	Dashte Azadegan	95	21042	0.79	0.8	0.45	1.26	55.74	2.02	3.54	0.80
2009	Ramhormoz	96	32015	0.81	0.78	0.5	1.22	57.88	2.00	3.57	0.80
2009	Shadegan	97	23532	0.85	0.9	0.5	1.32	40.08	1.99	3.38	0.80
2009	Shushtar	98	45227	0.9	0.88	0.59	1.28	64.87	1.93	3.33	0.80
2009	Masjedsoleyman	99	30974	0.59	0.46	0.34	0.91	67.53	1.99	3.63	0.80
2009	Shush	100	34082	0.99	1.13	0.63	1.44	46.57	1.98	3.47	0.80
2009	Baghmalek	101	17414	0.42	0.24	0.21	0.74	37.36	1.97	3.62	0.80
2009	Omidiyeh	102	16494	0.81	0.76	0.44	1.31	69.19	1.97	3.51	0.80
2009	Lali	103	6025	0.68	NA	0.25	1.46	48.78	1.89	3.48	0.80
2009	Hendijan	104	7334	1.07	1.12	0.53	1.91	70.95	2.02	3.65	0.80
2009	Abadeh	105	22453	1.07	1.05	0.65	1.59	88.04	1.90	3.59	0.72
2009	Estahban	106	13766	0.74	0.72	0.39	1.25	70.43	1.91	3.58	0.72
2009	Eqlid	107	20581	0.76	0.78	0.43	1.21	63.21	1.91	3.62	0.72
2009	Jahrom	108	43726	0.92	0.96	0.61	1.29	64.69	1.97	3.58	0.72
2009	Darab	109	35678	1.07	1.26	0.71	1.53	43.16	1.90	3.59	0.72
2009	Sepidan	110	17836	0.43	0.34	0.22	0.75	21.30	1.80	3.63	0.72
2009	Shiraz	111	393595	1.84	1.85	1.66	2.03	87.85	1.83	3.60	0.72
2009	Fasa	112	41774	1.05	1.1	0.71	1.46	59.77	1.88	3.59	0.72
2009	Firuzabad	113	25218	0.91	1.05	0.55	1.38	58.89	1.85	3.69	0.72
2009	Kazerun	114	55717	0.53	0.47	0.33	0.78	52.55	1.89	3.59	0.72

2009	Lar (Larestan)	115	55908	1.08	1.13	0.76	1.45	58.27	1.91	3.68	0.72
2009	Marvdasht	116	66380	0.69	0.72	0.46	0.96	47.15	1.82	3.66	0.72
2009	Mamasany	117	36809	0.43	0.44	0.24	0.68	36.03	1.96	3.56	0.72
2009	Neyriz	118	22395	0.64	0.64	0.36	1.03	59.69	1.92	3.61	0.72
2009	Lamard	119	15501	0.59	0.4	0.3	1.01	44.43	1.84	3.64	0.72
2009	Bovanat	120	10145	0.52	0.59	0.24	0.96	31.37	1.86	3.63	0.72
2009	Arsanjan	121	8375	1.22	1.76	0.62	2.09	40.05	1.77	3.66	0.72
2009	Khorrambid	122	9612	0.53	0.21	0.24	0.96	78.71	1.84	3.49	0.72
2009	Zarrindasht	123	11661	0.61	NA	0.24	1.33	57.50	1.94	3.81	0.72
2009	Qirokarzin	124	12893	0.41	0.16	0.19	0.76	56.44	1.88	3.58	0.72
2009	Mohr	125	10163	0.63	0.81	0.3	1.13	38.90	1.84	3.75	0.72
2009	Farashband	126	9201	0.66	0.67	0.31	1.18	56.67	1.82	3.82	0.72
2009	Baft	127	30157	0.53	0.59	0.3	0.84	34.40	1.11	4.17	0.76
2009	Bam	128	55199	0.57	0.65	0.36	0.83	38.95	1.12	4.13	0.76
2009	Jiroft	129	45695	0.61	0.68	0.38	0.91	40.79	1.09	4.09	0.76
2009	Rafsanjan	130	60625	0.89	0.87	0.62	1.21	57.22	1.10	4.25	0.76
2009	Zarand	131	30398	0.92	1.07	0.58	1.36	54.44	1.17	4.05	0.76
2009	Sirjan	132	50794	0.96	1	0.65	1.33	76.33	1.14	4.00	0.76
2009	Shahrebabak	133	19281	0.77	0.82	0.43	1.23	66.20	1.10	4.07	0.76
2009	Kerman	134	141015	1.53	1.57	1.26	1.82	87.52	1.05	4.03	0.76
2009	Kahnuj	135	44363	0.32	0.29	0.18	0.51	22.69	1.06	4.08	0.76
2009	Bardsir	136	15612	0.48	0.51	0.24	0.84	56.48	1.01	4.21	0.76
2009	Ravar	137	8516	0.5	0.23	0.22	0.94	56.34	1.11	4.00	0.76
2009	Anbarabad	138	13588	0.36	0.31	0.15	0.71	31.10	1.09	4.04	0.76
2009	Manujan	139	10551	0.33	0.2	0.13	0.65	30.03	1.10	4.18	0.76
2009	Taybad	140	25567	0.38	0.33	0.2	0.64	46.16	1.55	3.40	0.80
2009	Torbate Heydarieh	141	66939	0.62	0.6	0.41	0.87	47.77	1.42	3.37	0.80
2009	Torbate Jam	142	43265	0.61	0.63	0.38	0.92	45.21	1.41	3.19	0.80
2009	Darrehgaz	143	16565	0.49	0.35	0.25	0.84	56.38	1.43	3.30	0.80
2009	Sabzevar	144	98740	0.76	0.78	0.56	1	52.63	1.49	3.26	0.80
2009	Quchan	145	38279	0.49	0.46	0.29	0.76	53.16	1.47	3.29	0.80
2009	Kashmar	146	43201	0.38	0.19	0.21	0.6	50.52	1.47	3.32	0.80
2009	Gonabad	147	25185	0.64	0.62	0.37	1.01	49.90	1.50	3.23	0.80
2009	Mashhad	148	623458	1.15	1.15	1.03	1.27	90.53	1.46	3.17	0.80
2009	Neyshabur	149	96714	0.64	0.64	0.46	0.86	54.24	1.49	3.47	0.80
2009	Chenaran	150	23223	0.48	0.52	0.26	0.8	42.75	1.47	3.26	0.80
2009	Khaf	151	19345	0.38	0.32	0.19	0.66	45.44	1.42	3.28	0.80
2009	Sarakhs	152	14988	0.58	0.57	0.29	1	41.53	1.44	3.19	0.80
2009	Fariman	153	17334	0.7	0.95	0.38	1.15	55.32	1.53	3.16	0.80
2009	Bardeskan	154	15162	0.33	0.27	0.15	0.6	38.42	1.51	3.38	0.80
2009	Rashtkhar	155	10913	0.36	NA	0.13	0.81	19.54	1.49	3.19	0.80
2009	Kalat	156	7822	0.62	NA	0.23	1.35	25.47	1.41	3.30	0.80
2009	Ardestan	157	10742	0.55	0.35	0.26	0.98	58.36	2.49	4.11	0.73
2009	Isfahan	158	482093	1.54	1.54	1.39	1.7	92.06	2.48	4.11	0.73
2009	Khomeynishahr	159	61665	0.92	0.94	0.64	1.26	97.01	2.55	4.02	0.73
2009	Khansar	160	8319	0.53	0.22	0.26	0.95	62.79	2.47	4.10	0.73
2009	Semirom	161	14746	0.46	0.14	0.22	0.8	51.56	2.51	4.02	0.73

2009	Faridan	162	18493	0.76	0.63	0.42	1.23	43.14	2.53	4.10	0.73
2009	Fereydunshahr	163	8326	0.45	0.24	0.21	0.85	48.45	2.50	4.06	0.73
2009	Falavarjan	164	48233	0.84	0.9	0.56	1.2	61.48	2.54	3.89	0.73
2009	Shahreza	165	41942	0.67	0.57	0.42	0.98	80.88	2.55	4.04	0.73
2009	Kashan	166	70602	1.17	1.13	0.87	1.52	86.78	2.55	4.08	0.73
2009	Golpayegan	167	21041	0.55	0.36	0.3	0.91	75.34	2.58	4.04	0.73
2009	Lanjan	168	50703	1.33	1.42	0.95	1.78	84.04	2.56	4.08	0.73
2009	Nayin	169	13559	0.6	0.42	0.31	1.02	68.75	2.56	4.06	0.73
2009	Najafabad	170	62257	1.08	1.05	0.77	1.44	91.88	2.53	3.99	0.73
2009	Natanz	171	10477	0.61	0.55	0.3	1.07	56.78	2.49	4.26	0.73
2009	Shahinshahr va Meyme	172	65399	1.37	1.4	1.03	1.77	90.58	2.56	4.07	0.73
2009	Mobarakeh	173	29326	0.82	0.91	0.5	1.25	81.80	2.62	4.11	0.73
2009	Aran va Bidgol	174	20551	0.71	0.6	0.39	1.13	85.10	2.67	4.08	0.73
2009	Tiran va Karvan	175	15003	0.41	0.27	0.2	0.73	38.34	2.50	4.11	0.73
2009	Chadegan	176	7279	0.43	0.27	0.18	0.84	40.35	2.56	4.10	0.73
2009	Iranshahr	177	39265	0.35	0.28	0.19	0.56	43.23	-0.20	1.90	0.66
2009	Chah Bahar	178	40692	0.21	0.06	0.11	0.37	33.53	-0.19	1.98	0.66
2009	Khash	179	21087	0.3	NA	0.12	0.62	33.31	-0.20	2.17	0.66
2009	Zabol	180	63267	0.26	0.2	0.14	0.41	40.06	-0.21	1.93	0.66
2009	Zahedan	181	93442	0.81	0.87	0.59	1.08	88.65	-0.19	2.00	0.66
2009	Saravan	182	40901	0.23	0.3	0.12	0.4	35.73	-0.22	2.00	0.66
2009	Nikshahr	183	30317	0.21	NA	0.08	0.44	21.24	-0.24	2.03	0.66
2009	Sarbaz	184	21328	0.25	NA	0.07	0.62	12.00	-0.18	1.96	0.66
2009	Baneh	185	23536	0.42	0.35	0.22	0.72	64.91	1.15	2.36	0.85
2009	Bijar	186	22200	0.4	0.44	0.21	0.68	54.79	1.18	2.45	0.85
2009	Saqqez	187	43802	0.43	0.42	0.25	0.67	66.05	1.20	2.41	0.85
2009	Sanandaj	188	91813	1.11	1.15	0.84	1.41	82.18	1.21	2.50	0.85
2009	Qorveh	189	42211	0.45	0.48	0.26	0.71	54.44	1.16	2.24	0.85
2009	Marivan	190	29656	0.7	0.79	0.41	1.08	65.60	1.17	2.51	0.85
2009	Divandarreh	191	15861	0.37	0.39	0.18	0.67	29.34	1.24	2.43	0.85
2009	Kamyaran	192	20980	0.32	0.19	0.15	0.57	45.81	1.24	2.25	0.85
2009	Sarvabad	193	10808	0.26	0.18	0.11	0.51	6.38	1.23	2.34	0.85
2009	Tuyserkan	194	25402	0.5	0.53	0.28	0.81	45.76	1.72	3.13	0.68
2009	Malayer	195	63822	0.87	0.99	0.61	1.19	59.33	1.74	3.07	0.68
2009	Nahavand	196	39738	0.46	0.49	0.27	0.71	50.20	1.65	3.15	0.68
2009	Hamadan	197	148558	1.09	1.13	0.87	1.33	82.59	1.77	3.17	0.68
2009	Kabudarahang	198	26834	0.26	0.15	0.13	0.46	17.20	1.68	3.16	0.68
2009	Asadabad	199	22245	0.47	0.45	0.25	0.78	49.28	1.70	3.18	0.68
2009	Bahar	200	25888	0.44	0.39	0.24	0.73	42.66	1.82	3.00	0.68
2009	Razan	201	24475	0.4	0.41	0.21	0.66	20.10	1.66	3.24	0.68
2009	Borujen	202	25478	0.42	0.32	0.22	0.7	81.67	1.28	2.77	0.61
2009	Shahrekord	203	84124	1.12	1.21	0.83	1.43	73.81	1.18	2.69	0.61
2009	Farsan	204	16694	0.47	0.5	0.24	0.82	64.02	1.23	2.79	0.61
2009	Lordakan	205	30166	0.41	0.49	0.22	0.68	20.70	1.22	2.89	0.61
2009	Ardal	206	9522	0.29	0.21	0.11	0.63	15.21	1.20	2.52	0.61
2009	Kuhrang	207	5695	0.29	NA	0.11	0.62	6.78	1.23	2.89	0.61
2009	Aligudarz	208	26209	0.4	0.31	0.22	0.68	62.17	1.66	3.34	0.64

2009	Borujerd	209	74450	1.13	1.17	0.84	1.45	76.14	1.71	3.33	0.64
2009	Khorramabad	210	106106	1.06	1.08	0.82	1.34	68.45	1.55	3.22	0.64
2009	Dalfan	211	23902	0.36	0.35	0.18	0.61	42.60	1.72	3.21	0.64
2009	Dorud	212	32046	0.5	0.45	0.28	0.79	66.12	1.67	3.20	0.64
2009	Kuhdasht	213	38910	0.23	0.06	0.12	0.4	51.31	1.65	3.23	0.64
2009	Azna	214	15148	0.41	0.26	0.2	0.74	55.05	1.64	3.20	0.64
2009	Poldokhtar	215	14858	0.54	0.57	0.26	0.95	39.37	1.62	3.31	0.64
2009	Selseleh	216	14205	0.53	NA	0.17	1.25	41.13	1.61	3.29	0.64
2009	Ilam	217	40317	0.75	0.68	0.47	1.1	81.98	1.63	3.15	0.67
2009	Darrehshahr	218	11683	0.35	0.36	0.14	0.68	38.90	1.65	3.33	0.67
2009	Dehloran	219	11674	0.54	0.54	0.25	0.99	64.86	1.69	3.23	0.67
2009	Shirvan va Chardavol	220	14457	0.34	0.28	0.15	0.63	23.78	1.70	3.13	0.67
2009	Mehran	221	9623	0.7	NA	0.28	1.44	60.50	1.66	3.03	0.67
2009	Abdanan	222	9063	0.41	0.23	0.18	0.77	60.59	1.65	3.08	0.67
2009	Eyvan	223	10336	0.88	0.99	0.45	1.51	64.44	1.65	3.13	0.67
2009	Boyerahmad	224	39299	0.53	0.5	0.31	0.83	44.50	1.62	3.09	0.51
2009	Kohgiluyeh	225	40367	0.43	0.57	0.24	0.7	43.17	1.62	3.00	0.51
2009	Gachsaran	226	28040	0.8	0.96	0.48	1.23	65.36	1.69	3.15	0.51
2009	Dena	227	10139	0.34	0.2	0.15	0.63	19.75	1.69	3.11	0.51
2009	Bushehr	228	44357	2.26	2.37	1.71	2.91	85.34	2.17	3.48	0.75
2009	Tangestan	229	13609	0.54	0.59	0.27	0.96	23.17	2.13	3.46	0.75
2009	Dashtestan	230	46581	0.66	0.69	0.42	0.97	62.64	2.12	3.69	0.75
2009	Dashti	231	15455	0.44	0.13	0.22	0.76	56.67	2.07	3.61	0.75
2009	Dayyer	232	9476	0.56	0.44	0.27	1	68.72	2.05	3.77	0.75
2009	Kangan	233	13123	0.5	0.16	0.24	0.9	68.45	2.16	3.45	0.75
2009	Genaveh	234	17959	0.94	0.93	0.53	1.5	73.79	2.10	3.58	0.75
2009	Deylam	235	6199	0.85	NA	0.33	1.81	74.36	2.12	3.51	0.75
2009	Jam	236	7581	0.4	0.3	0.16	0.81	37.01	2.19	3.73	0.75
2009	Abhar	237	36271	0.42	0.39	0.24	0.68	65.24	1.22	2.91	0.60
2009	Khodabandeh	238	32686	0.3	0.31	0.16	0.51	26.88	1.20	2.87	0.60
2009	Zanjan	239	101198	0.7	0.69	0.5	0.92	78.95	1.15	2.87	0.60
2009	Ijrud	240	8051	0.32	NA	0.12	0.67	7.14	1.07	2.85	0.60
2009	Khorramdarreh	241	13562	0.61	NA	0.26	1.23	79.12	1.19	2.85	0.60
2009	Tarom	242	9552	0.34	NA	0.12	0.77	14.25	1.06	2.87	0.60
2009	Mahneshan	243	8091	0.33	0.25	0.12	0.67	15.76	1.19	2.82	0.60
2009	Damghan	244	19425	0.92	0.9	0.53	1.44	68.66	3.06	5.81	0.68
2009	Semnan	245	43785	0.92	0.98	0.6	1.3	88.35	2.99	5.71	0.68
2009	Shahrud	246	51938	0.97	0.91	0.68	1.33	64.76	3.06	5.63	0.68
2009	Garmsar	247	18571	0.75	0.64	0.42	1.23	68.71	3.03	5.66	0.68
2009	Ardakan	248	14005	0.99	1.27	0.56	1.58	81.87	2.29	4.05	0.65
2009	Bafq	249	10956	0.99	1.3	0.53	1.66	66.11	2.41	4.25	0.65
2009	Taft	250	11603	0.62	0.63	0.32	1.06	33.07	2.40	4.10	0.65
2009	Mehriz	251	9485	1.27	1.65	0.69	2.08	56.99	2.32	4.30	0.65
2009	Yazd	252	109871	1.69	1.67	1.37	2.04	95.29	2.40	4.40	0.65
2009	Meybod	253	14792	1.07	1.23	0.61	1.68	89.93	2.45	4.25	0.65
2009	Abarkuh	254	9682	0.71	0.41	0.36	1.25	63.29	2.37	4.24	0.65
2009	Sadugh	255	6215	1.06	1.56	0.47	1.97	53.25	2.42	4.36	0.65

2009	Khatam	256	6694	0.7	NA	0.27	1.5	56.69	2.34	4.23	0.65
2009	Tabas	257	13453	0.57	0.3	0.28	1	56.23	2.35	4.15	0.65
2009	Abumusa	258	280	1.07	NA	0.39	2.36	98.59	0.50	2.92	0.45
2009	Bandarabbas	259	106603	0.81	0.81	0.6	1.06	69.14	0.41	2.89	0.45
2009	Bandar-e Lengeh	260	27562	0.49	0.54	0.28	0.78	47.83	0.56	2.82	0.45
2009	Qeshm	261	17545	0.5	0.49	0.26	0.87	39.99	0.45	2.94	0.45
2009	Minab	262	45666	0.37	0.36	0.22	0.59	25.50	0.46	2.87	0.45
2009	Bandar-e-Jask	263	12509	0.33	NA	0.16	0.61	15.99	0.50	2.86	0.45
2009	Rudan	264	19529	0.25	0.11	0.12	0.46	34.31	0.39	3.07	0.45
2009	Hajiabad	265	12800	0.29	0.16	0.13	0.56	32.00	0.47	2.90	0.45
2009	Bastak	266	12677	0.4	0.32	0.19	0.73	22.81	0.48	2.94	0.45
2009	Tehran	267	2065702	1.75	1.75	1.67	1.83	99.61	2.80	4.98	0.75
2009	Damavand	268	21224	1.02	1.04	0.6	1.56	72.24	2.69	4.87	0.75
2009	Rey	269	56469	1.47	1.52	1.09	1.93	27.19	2.79	4.82	0.75
2009	Shemiranat	270	9998	1.66	1.73	0.95	2.6	53.83	2.75	4.83	0.75
2009	Varamin	271	109303	1	1.03	0.76	1.27	80.49	2.80	4.81	0.75
2009	Shahriyar	272	225343	0.5	0.5	0.38	0.64	85.99	2.72	4.68	0.75
2009	Eslamshahr	273	94394	0.52	0.51	0.35	0.72	89.59	2.78	4.88	0.75
2009	Robatkarim	274	119838	0.39	0.41	0.26	0.54	81.25	2.76	4.95	0.75
2009	Pakdasht	275	47111	0.38	0.33	0.22	0.61	76.53	2.67	5.00	0.75
2009	Firuzkuh	276	8869	0.47	0.43	0.21	0.9	40.01	2.72	4.74	0.75
2009	Ardebil	277	117241	0.64	0.67	0.47	0.85	83.00	1.55	2.61	0.66
2009	Bilehsowar	278	11195	0.44	0.73	0.2	0.82	38.19	1.46	2.62	0.66
2009	Khalkhal	279	20928	0.6	NA	0.21	1.39	46.56	1.53	2.60	0.66
2009	Meshginshahr	280	32670	0.73	0.79	0.44	1.1	42.96	1.47	2.63	0.66
2009	Germi	281	18256	0.34	0.22	0.16	0.61	31.71	1.49	2.72	0.66
2009	Parsabad	282	32760	0.27	0.13	0.14	0.47	54.58	1.45	2.70	0.66
2009	Kowsar	283	5866	0.41	0.66	0.14	0.91	25.09	1.41	2.78	0.66
2009	Namin	284	12644	0.34	0.15	0.15	0.63	35.28	1.38	2.60	0.66
2009	Neer	285	5363	0.37	NA	0.13	0.84	24.25	1.38	2.94	0.66
2009	Qom	286	218256	0.52	0.48	0.4	0.65	93.47	2.80	4.09	0.68
2009	Bueenzahra	287	33037	0.24	0.12	0.12	0.42	34.72	2.17	3.67	0.75
2009	Takestan	288	35044	0.47	0.35	0.27	0.74	59.27	2.01	3.52	0.75
2009	Qazvin	289	160487	0.89	0.88	0.7	1.1	80.01	2.13	3.77	0.75
2009	Abyek	290	18481	0.65	0.78	0.34	1.07	53.41	2.09	3.72	0.75
2009	Bandare Gaz	291	11345	0.72	0.69	0.38	1.22	53.57	1.66	3.47	0.69
2009	Torkman	292	26975	0.55	0.53	0.31	0.88	59.95	1.73	3.03	0.69
2009	Aliabad	293	26653	0.67	0.69	0.38	1.05	47.91	1.74	3.15	0.69
2009	Kordkuy	294	16331	0.61	0.48	0.33	1	50.23	1.70	3.20	0.69
2009	Gorgan	295	94452	1.13	1.12	0.86	1.43	72.40	1.73	3.32	0.69
2009	Gonbade Kavus	296	62028	0.69	0.7	0.46	0.97	49.14	1.63	3.29	0.69
2009	Minudasht	297	26390	0.5	0.62	0.27	0.82	34.09	1.69	3.22	0.69
2009	Aqqala	298	22380	0.42	0.66	0.21	0.72	29.71	1.66	3.17	0.69
2009	Kalaleh	299	28938	0.29	0.22	0.15	0.51	22.55	1.73	3.34	0.69
2009	Azadshahr	300	18256	0.46	0.34	0.24	0.79	54.48	1.70	3.25	0.69
2009	Ramyan	301	16405	0.29	0.25	0.13	0.54	40.00	1.66	3.19	0.69
2009	Esfarayen	302	26913	0.31	0.3	0.16	0.54	41.02	1.00	3.16	0.60

2009	Bojnurd	303	65195	0.68	0.75	0.45	0.95	58.25	0.99	2.95	0.60
2009	Jajarm	304	11942	0.29	0.17	0.11	0.58	61.72	0.96	3.00	0.60
2009	Shirvan	305	32939	0.36	0.3	0.2	0.6	51.66	0.96	3.22	0.60
2009	Faruj	306	11509	0.26	0.16	0.11	0.51	25.02	0.97	3.36	0.60
2009	Maneh va Semelqan	307	18311	0.38	NA	0.15	0.8	24.39	1.01	3.24	0.60
2009	Birjand	308	57894	0.89	0.92	0.61	1.22	57.13	0.42	2.96	0.43
2009	Sarbisheh	309	10442	0.43	NA	0.12	1.09	23.58	0.44	2.95	0.43
2009	Qaenat	310	29512	0.36	0.34	0.19	0.6	34.23	1.49	3.43	0.43
2009	Nehbandan	311	10337	0.34	NA	0.11	0.8	27.78	0.52	3.01	0.43
2009	Sarayan	312	7401	0.54	NA	0.2	1.2	60.41	0.53	3.06	0.43
2009	Ferdows	313	14909	0.56	0.65	0.29	0.97	67.57	1.47	3.38	0.43
2009	Karaj	314	421367	1.04	1.07	0.91	1.18	96.86	2.83	4.74	0.44
2009	Savojbolagh	315	49424	0.11	0.08	0.05	0.22	37.69	2.76	4.75	0.44
2009	Nazarabad	316	26996	0.35	0.23	0.17	0.6	78.37	2.82	4.95	0.44
2010	Arak	1	149252	1.25	1.29	1.03	1.51	81.23	2.24	3.84	0.82
2010	Ashtiyan	2	4509	0.87	1.26	0.37	1.69	38.31	2.18	4.07	0.82
2010	Tafresh	3	14654	0.41	0.13	0.2	0.72	29.39	2.25	3.72	0.82
2010	Khomeyn	4	27534	0.51	0.49	0.28	0.81	57.23	2.20	3.73	0.82
2010	Delijan	5	10919	1.19	1.66	0.64	1.95	71.16	2.30	3.87	0.82
2010	Saveh	6	52315	0.89	1.04	0.6	1.25	71.59	2.21	3.85	0.82
2010	Shazand	7	28608	0.65	0.83	0.38	1.02	30.85	2.28	3.95	0.82
2010	Mahalat	8	12600	0.82	0.93	0.43	1.36	82.45	2.15	3.96	0.82
2010	Zarandiyeh	9	13021	0.42	0.3	0.2	0.77	58.08	2.20	4.01	0.82
2010	Komijan	10	9148	0.47	0.44	0.2	0.91	40.02	2.13	3.89	0.82
2010	Astara	11	19093	0.74	0.95	0.4	1.21	65.85	2.13	4.59	0.75
2010	Astanehye Ashrafiyeh	12	29391	0.99	1.17	0.63	1.45	46.44	2.10	4.55	0.75
2010	Bandar Anzali	13	38247	1.1	1.08	0.75	1.53	83.58	2.06	4.61	0.75
2010	Tavalesh	14	41704	0.63	0.72	0.39	0.93	36.24	2.05	4.48	0.75
2010	Rasht	15	237305	1.31	1.32	1.12	1.52	72.34	2.08	4.38	0.75
2010	Rudbar	16	24797	0.63	0.55	0.36	0.99	52.33	2.03	4.49	0.75
2010	Rudsar	17	41448	1.09	1.14	0.75	1.51	44.36	2.10	4.59	0.75
2010	Sumehsara	18	34046	0.57	0.45	0.34	0.88	33.02	2.04	4.67	0.75
2010	Fuman	19	26202	0.55	0.51	0.31	0.89	26.83	2.08	4.68	0.75
2010	Langrud	20	37652	0.83	0.81	0.53	1.21	62.12	2.05	4.40	0.75
2010	Lahijan	21	46893	1.04	1.01	0.72	1.43	51.44	2.07	4.65	0.75
2010	Shaft	22	16618	0.62	0.81	0.32	1.05	12.92	2.07	4.60	0.75
2010	Amlash	23	12490	0.67	0.76	0.34	1.15	34.04	2.06	4.62	0.75
2010	Rezvanshahr	24	15988	0.59	0.62	0.3	1.01	30.43	2.06	4.72	0.75
2010	Siyahkal	25	12857	0.79	0.88	0.43	1.31	35.67	1.99	4.51	0.75
2010	Masal	26	12557	0.53	0.47	0.26	0.93	30.19	2.02	4.53	0.75
2010	Amol	27	89694	0.8	0.78	0.58	1.06	57.24	2.67	4.59	0.78
2010	Babol	28	120567	1.2	1.2	0.96	1.47	48.61	2.66	4.73	0.78
2010	Behshahr	29	47102	1.04	1.12	0.72	1.42	63.70	2.66	4.75	0.78
2010	Tonekabon	30	51582	0.75	0.75	0.5	1.05	47.44	2.59	4.79	0.78
2010	Ramsar	31	18514	1.31	1.53	0.81	1.97	73.22	2.59	4.76	0.78
2010	Sari	32	128738	0.99	0.98	0.78	1.23	54.22	2.63	4.76	0.78
2010	Savadkuh	33	16619	0.41	0.23	0.2	0.72	46.80	2.64	4.71	0.78

2010	Qaemshahr	34	78713	0.8	0.79	0.57	1.07	61.29	2.69	4.72	0.78
2010	Nur	35	26476	0.33	0.08	0.17	0.57	40.09	2.64	4.56	0.78
2010	Noshahr	36	30454	0.32	0.26	0.17	0.55	34.83	2.62	4.80	0.78
2010	Babolsar	37	43288	0.67	0.63	0.42	0.97	52.82	2.60	4.59	0.78
2010	Mahmudabad	38	22915	0.59	0.52	0.33	0.95	34.14	2.66	4.79	0.78
2010	Neka	39	25288	0.68	0.79	0.38	1.06	37.94	2.58	4.65	0.78
2010	Chalus	40	30358	0.81	1.1	0.49	1.22	51.71	2.66	4.78	0.78
2010	Juybar	41	17366	0.62	0.56	0.33	1.06	39.35	2.62	4.69	0.78
2010	Ahar	42	33055	0.3	0.12	0.16	0.5	63.97	1.95	3.18	0.84
2010	Tabriz	43	401829	1.27	1.27	1.12	1.43	92.98	1.95	3.33	0.84
2010	Sarab	44	29674	0.55	0.53	0.31	0.86	44.42	1.96	3.26	0.84
2010	Maragheh	45	56071	0.44	0.39	0.27	0.67	69.61	1.99	3.30	0.84
2010	Marand	46	56807	0.58	0.46	0.37	0.83	59.87	1.95	3.20	0.84
2010	Miyaneh	47	42583	0.39	0.28	0.22	0.6	52.62	1.93	3.15	0.84
2010	Hashtrud	48	13025	0.47	0.46	0.23	0.84	31.86	1.85	3.10	0.84
2010	Bonab	49	29166	0.65	0.7	0.37	1.01	61.38	1.89	3.10	0.84
2010	Bostanabad	50	19535	0.4	0.31	0.2	0.69	20.19	1.95	3.13	0.84
2010	Shabestar	51	31930	0.37	0.24	0.2	0.62	47.00	2.01	3.06	0.84
2010	Kalibar	52	18322	0.32	NA	0.13	0.67	14.67	1.96	3.26	0.84
2010	Haris	53	14715	0.58	0.56	0.3	1.01	46.91	1.98	3.21	0.84
2010	Jolfa	54	13390	0.5	NA	0.2	1.05	62.41	1.93	3.22	0.84
2010	Malekan	55	22503	0.37	0.36	0.19	0.65	31.39	1.94	3.29	0.84
2010	Azarshahr	56	25000	0.53	0.64	0.29	0.87	59.54	1.85	3.20	0.84
2010	Osku	57	22257	0.31	0.09	0.15	0.54	55.38	1.94	3.29	0.84
2010	Charoymaq	58	6062	0.31	NA	0.12	0.66	14.50	2.03	3.28	0.84
2010	Varzaqan	59	9954	0.32	0.2	0.14	0.6	11.98	1.96	3.22	0.84
2010	Ajabshir	60	14909	0.45	0.4	0.22	0.8	42.55	1.94	3.24	0.84
2010	Orumiyeh	61	206816	0.91	0.91	0.74	1.1	73.29	2.03	2.74	0.68
2010	Piranshahr	62	21389	0.4	0.3	0.2	0.68	54.20	2.05	2.91	0.68
2010	Khoy	63	82817	0.41	0.41	0.26	0.59	66.98	2.05	2.95	0.68
2010	Sardasht	64	20484	0.29	0.3	0.14	0.54	50.70	2.03	2.85	0.68
2010	Salmas	65	37531	0.47	0.32	0.27	0.75	54.76	2.05	2.82	0.68
2010	Maku	66	35569	0.27	0.23	0.14	0.46	49.78	2.09	2.99	0.68
2010	Mahabad	67	44385	0.38	0.37	0.22	0.6	69.95	2.03	2.91	0.68
2010	Miyandoab	68	55251	0.55	0.63	0.35	0.81	52.43	2.05	2.85	0.68
2010	Naqadeh	69	26173	0.72	0.84	0.42	1.11	68.50	2.01	2.95	0.68
2010	Bukan	70	45948	0.41	0.45	0.24	0.65	75.40	2.00	2.82	0.68
2010	Shahindezh	71	20209	0.44	NA	0.18	0.92	52.47	2.04	2.95	0.68
2010	Takab	72	17371	0.28	0.11	0.13	0.52	56.24	2.00	2.90	0.68
2010	Oshnaviyeh	73	13175	0.81	0.95	0.42	1.36	53.77	2.07	2.77	0.68
2010	Chaldoran	74	8438	0.36	0.24	0.14	0.71	34.79	2.13	3.01	0.68
2010	Eslamabade Gharb	75	43870	0.23	0.05	0.12	0.39	54.34	1.49	3.12	0.82
2010	Kermanshah	76	228556	1.19	1.2	1.01	1.39	84.77	1.60	2.89	0.82
2010	Paveh	77	12482	0.5	0.47	0.24	0.88	51.70	1.57	2.99	0.82
2010	Sarpole Zahab	78	17455	0.49	0.46	0.24	0.85	40.98	1.55	3.05	0.82
2010	Sonqor	79	21779	0.58	0.62	0.32	0.95	49.76	1.55	3.15	0.82
2010	Qasreshirin	80	4850	0.82	0.85	0.36	1.57	72.75	1.55	3.14	0.82

2010	Kangavar	81	18214	0.59	0.54	0.32	0.98	63.61	1.52	3.09	0.82
2010	Gilanegharb	82	14336	0.33	0.14	0.15	0.62	35.88	1.52	3.13	0.82
2010	Javanrud	83	22441	0.44	0.45	0.22	0.75	58.51	1.58	3.09	0.82
2010	Sahneh	84	17916	0.43	0.22	0.22	0.74	45.44	1.57	3.00	0.82
2010	Harsin	85	18253	0.54	0.55	0.28	0.92	65.91	1.58	3.00	0.82
2010	Salas-e-Babajani	86	6432	0.37	NA	0.12	0.86	26.85	1.61	3.12	0.82
2010	Abadan	87	55813	1.87	1.9	1.44	2.37	85.42	2.12	3.70	0.85
2010	Andimeshk	88	33066	0.77	0.76	0.47	1.15	79.77	2.06	3.64	0.85
2010	Ahvaz	89	281079	1.5	1.5	1.3	1.72	83.87	2.05	3.51	0.85
2010	Izeh	90	36285	0.62	0.73	0.37	0.94	57.38	2.11	3.52	0.85
2010	Bandar-e-Mahshahr	91	50502	1.63	1.75	1.2	2.14	93.56	2.09	3.57	0.85
2010	Behbahan	92	38732	0.99	0.98	0.65	1.41	72.68	2.07	3.74	0.85
2010	Khorramshahr	93	31067	1.36	1.47	0.91	1.93	83.15	2.08	3.59	0.85
2010	Dezful	94	79896	0.92	0.93	0.67	1.22	73.99	2.06	3.50	0.85
2010	Dashte Azadegan	95	21859	0.98	1.16	0.58	1.51	55.45	2.13	3.69	0.85
2010	Ramhormoz	96	33144	0.99	1.07	0.64	1.45	59.35	2.08	3.56	0.85
2010	Shadegan	97	24820	0.71	0.6	0.41	1.12	40.34	2.19	3.72	0.85
2010	Shushtar	98	47131	0.94	0.94	0.63	1.32	66.08	2.12	3.66	0.85
2010	Masjedsoleyman	99	31522	0.76	0.78	0.46	1.15	67.63	2.10	3.69	0.85
2010	Shush	100	35644	0.68	0.6	0.41	1.03	46.70	2.06	3.62	0.85
2010	Baghmalek	101	18135	0.6	0.8	0.32	1.02	37.78	2.09	3.59	0.85
2010	Omidiyeh	102	17155	1.02	1.21	0.59	1.59	69.23	2.10	3.68	0.85
2010	Lali	103	6240	0.61	0.33	0.25	1.21	49.36	2.15	3.54	0.85
2010	Hendijan	104	7606	1.15	1.36	0.57	2.01	71.15	2.11	3.76	0.85
2010	Abadeh	105	23243	1.2	1.26	0.76	1.78	87.88	2.07	3.79	0.78
2010	Estahban	106	14158	0.73	0.7	0.39	1.25	70.27	2.05	3.73	0.78
2010	Eqlid	107	21129	0.9	1.05	0.53	1.41	63.25	1.96	3.90	0.78
2010	Jahrom	108	45430	0.93	0.96	0.62	1.31	64.86	2.02	3.97	0.78
2010	Darab	109	37518	0.69	0.66	0.42	1.04	43.42	2.04	3.68	0.78
2010	Sepidan	110	18406	0.72	1.11	0.4	1.18	21.30	2.01	3.96	0.78
2010	Shiraz	111	413282	1.9	1.91	1.72	2.09	87.82	2.10	3.78	0.78
2010	Fasa	112	43490	0.99	1	0.66	1.39	60.28	2.00	3.72	0.78
2010	Firuzabad	113	26354	0.69	0.62	0.4	1.08	59.46	2.06	3.84	0.78
2010	Kazerun	114	57261	0.86	0.95	0.59	1.19	52.92	1.99	3.75	0.78
2010	Lar (Larestan)	115	58850	1.15	1.21	0.83	1.52	58.26	2.08	3.85	0.78
2010	Marvdasht	116	69232	0.73	0.78	0.5	1.01	47.59	2.02	3.84	0.78
2010	Mamasany	117	37985	0.51	0.58	0.3	0.8	36.90	2.03	3.85	0.78
2010	Neyriz	118	23330	0.56	0.44	0.31	0.9	59.87	2.08	3.69	0.78
2010	Lamard	119	16380	0.72	0.76	0.39	1.18	44.68	2.04	3.77	0.78
2010	Bovanat	120	10474	0.47	0.38	0.22	0.87	31.78	2.12	3.68	0.78
2010	Arsanjan	121	8740	0.96	0.96	0.47	1.68	40.14	2.02	3.74	0.78
2010	Khorrambid	122	10012	0.59	0.41	0.28	1.06	79.10	2.10	3.76	0.78
2010	Zarrindasht	123	12329	0.47	0.17	0.21	0.87	57.81	2.07	3.68	0.78
2010	Qirokarzin	124	13430	0.46	0.31	0.22	0.84	56.97	2.09	3.92	0.78
2010	Mohr	125	10826	0.57	0.57	0.27	1.03	39.69	2.04	3.82	0.78
2010	Farashband	126	9465	0.71	0.86	0.34	1.26	57.69	2.06	3.77	0.78
2010	Baft	127	31346	0.52	0.57	0.3	0.82	33.80	1.23	4.21	0.79

2010	Bam	128	60886	0.31	0.21	0.18	0.49	39.29	1.21	4.22	0.79
2010	Jiroft	129	49038	0.49	0.47	0.29	0.74	40.59	1.26	4.27	0.79
2010	Rafsanjan	130	63333	0.92	0.93	0.65	1.25	57.15	1.18	4.25	0.79
2010	Zarand	131	31561	0.98	1.16	0.62	1.44	54.83	1.27	4.14	0.79
2010	Sirjan	132	53209	0.93	0.95	0.65	1.29	76.61	1.31	4.23	0.79
2010	Shahrebabak	133	19447	0.72	0.72	0.4	1.16	66.95	1.27	4.20	0.79
2010	Kerman	134	147127	1.37	1.39	1.12	1.64	87.38	1.21	4.26	0.79
2010	Kahnuj	135	46987	0.38	0.42	0.21	0.59	22.81	1.28	4.28	0.79
2010	Bardsir	136	15597	0.44	0.38	0.21	0.78	56.91	1.20	4.28	0.79
2010	Ravar	137	8759	0.5	0.23	0.21	0.93	55.78	1.25	4.22	0.79
2010	Anbarabad	138	14283	0.36	0.3	0.15	0.71	30.62	1.20	4.30	0.79
2010	Manujan	139	11135	0.36	NA	0.13	0.8	29.87	1.23	4.22	0.79
2010	Taybad	140	26861	0.37	0.31	0.19	0.63	46.17	1.70	3.41	0.85
2010	Torbate Heydarieh	141	69614	0.42	0.32	0.27	0.62	48.06	1.69	3.26	0.85
2010	Torbate Jam	142	45187	0.66	0.7	0.42	0.97	45.58	1.69	3.55	0.85
2010	Darrehgaz	143	16938	0.75	1.02	0.41	1.2	56.66	1.69	3.39	0.85
2010	Sabzevar	144	102501	0.84	0.87	0.62	1.08	53.51	1.72	3.50	0.85
2010	Quchan	145	39235	0.61	0.65	0.37	0.91	53.98	1.67	3.62	0.85
2010	Kashmar	146	44894	0.65	0.67	0.41	0.95	50.90	1.73	3.40	0.85
2010	Gonabad	147	25820	0.6	0.53	0.34	0.95	49.99	1.61	3.28	0.85
2010	Mashhad	148	652954	1.38	1.39	1.25	1.51	90.53	1.74	3.44	0.85
2010	Neyshabur	149	100541	0.3	0.18	0.19	0.44	54.79	1.65	3.31	0.85
2010	Chenaran	150	24400	0.41	0.33	0.22	0.69	43.53	1.66	3.38	0.85
2010	Khaf	151	20200	0.35	0.21	0.17	0.62	45.52	1.67	3.35	0.85
2010	Sarakhs	152	15542	0.63	0.68	0.32	1.07	41.77	1.70	3.30	0.85
2010	Fariman	153	18013	0.49	0.34	0.25	0.84	55.80	1.67	3.49	0.85
2010	Bardeskan	154	15664	0.3	0.13	0.14	0.55	38.90	1.66	3.42	0.85
2010	Rashtkhar	155	11359	0.32	0.18	0.13	0.65	19.76	1.64	3.35	0.85
2010	Kalat	156	7960	0.62	NA	0.23	1.35	25.83	1.72	3.37	0.85
2010	Ardestan	157	10840	0.72	0.87	0.36	1.25	59.31	2.80	4.36	0.82
2010	Isfahan	158	506053	1.54	1.53	1.4	1.69	92.19	2.68	4.13	0.82
2010	Khomeynishahr	159	64689	0.94	0.96	0.67	1.26	97.03	2.83	4.20	0.82
2010	Khansar	160	8477	0.53	0.22	0.26	0.94	62.93	2.75	4.27	0.82
2010	Semirom	161	14972	0.59	0.53	0.3	1.02	51.80	2.69	4.32	0.82
2010	Faridan	162	18904	0.81	0.72	0.46	1.3	43.60	2.67	4.31	0.82
2010	Fereydunshahr	163	8548	0.46	0.23	0.21	0.88	48.63	2.78	4.34	0.82
2010	Falavarjan	164	50237	0.77	0.78	0.51	1.1	61.38	2.79	4.14	0.82
2010	Shahreza	165	43163	0.84	0.82	0.54	1.2	81.24	2.79	4.21	0.82
2010	Kashan	166	73062	1.34	1.33	1.02	1.72	87.11	2.76	4.21	0.82
2010	Golpayegan	167	21613	0.89	1.06	0.52	1.38	75.84	2.73	4.12	0.82
2010	Lanjan	168	53069	1.17	1.2	0.82	1.58	84.35	2.72	4.33	0.82
2010	Nayin	169	13865	0.65	0.55	0.33	1.09	69.17	2.66	4.20	0.82
2010	Najafabad	170	64679	1.49	1.54	1.12	1.91	91.89	2.78	4.28	0.82
2010	Natanz	171	10632	0.6	0.54	0.29	1.05	57.29	2.78	4.16	0.82
2010	Shahinshahr va Meyme	172	68363	1.27	1.28	0.95	1.64	90.71	2.72	4.10	0.82
2010	Mobarakeh	173	30451	0.66	0.61	0.39	1.02	81.78	2.70	4.27	0.82
2010	Aran va Bidgol	174	21300	1.03	1.24	0.61	1.58	84.98	2.65	4.06	0.82

2010	Tiran va Karvan	175	15518	0.48	0.51	0.23	0.85	38.42	2.77	4.18	0.82
2010	Chadegan	176	7480	0.43	0.26	0.18	0.81	40.58	2.72	4.18	0.82
2010	Iranshahr	177	41191	0.34	0.26	0.18	0.55	43.34	-0.06	2.06	0.73
2010	Chah Bahar	178	43970	0.24	0.15	0.12	0.41	33.78	-0.03	2.06	0.73
2010	Khash	179	21639	0.27	0.2	0.13	0.5	33.72	0.01	2.08	0.73
2010	Zabol	180	65279	0.35	0.38	0.21	0.55	40.62	0.02	2.04	0.73
2010	Zahedan	181	97126	1.07	1.17	0.8	1.37	89.04	0.03	2.05	0.73
2010	Saravan	182	42946	0.17	0.1	0.08	0.3	35.53	-0.08	2.26	0.73
2010	Nikshahr	183	31841	0.2	0.2	0.09	0.36	21.08	0.01	2.00	0.73
2010	Sarbaz	184	22572	0.25	NA	0.07	0.64	12.41	0.05	2.15	0.73
2010	Baneh	185	24744	0.51	0.59	0.27	0.84	65.84	1.44	2.46	0.89
2010	Bijar	186	22574	0.31	0.17	0.16	0.54	55.21	1.34	2.46	0.89
2010	Saqqez	187	45228	0.43	0.4	0.26	0.66	66.42	1.44	2.70	0.89
2010	Sanandaj	188	95653	1.15	1.2	0.88	1.46	82.27	1.42	2.47	0.89
2010	Qorveh	189	43511	0.48	0.51	0.28	0.74	55.19	1.43	2.52	0.89
2010	Marivan	190	31130	0.64	0.68	0.38	1	66.56	1.33	2.49	0.89
2010	Divandarreh	191	16268	0.34	0.25	0.17	0.62	30.14	1.33	2.51	0.89
2010	Kamyaran	192	21656	0.34	0.28	0.17	0.6	46.59	1.32	2.41	0.89
2010	Sarvabad	193	11036	0.28	NA	0.11	0.59	6.78	1.39	2.49	0.89
2010	Tuyserkan	194	25833	0.47	0.44	0.26	0.76	46.03	1.99	3.32	0.76
2010	Malayer	195	65615	0.53	0.51	0.35	0.75	59.83	2.02	3.16	0.76
2010	Nahavand	196	40960	0.5	0.57	0.3	0.77	50.35	2.05	3.19	0.76
2010	Hamadan	197	155114	0.94	0.96	0.75	1.16	82.80	1.99	3.33	0.76
2010	Kabudarahang	198	27594	0.24	0.07	0.12	0.43	17.29	1.98	3.20	0.76
2010	Asadabad	199	22984	0.47	0.43	0.25	0.77	49.67	1.99	3.23	0.76
2010	Bahar	200	26665	0.48	0.45	0.26	0.78	42.65	1.99	3.33	0.76
2010	Razan	201	25259	0.4	0.4	0.21	0.67	20.41	1.97	3.35	0.76
2010	Borujen	202	26390	0.55	0.62	0.31	0.88	81.82	1.44	2.87	0.71
2010	Shahrekord	203	87144	1	1.07	0.75	1.29	74.06	1.41	2.85	0.71
2010	Farsan	204	17340	0.44	0.36	0.22	0.76	64.08	1.39	3.02	0.71
2010	Lordakan	205	31647	0.3	0.2	0.16	0.51	20.99	1.42	2.99	0.71
2010	Ardal	206	9844	0.33	NA	0.11	0.77	15.22	1.46	2.83	0.71
2010	Kuhrang	207	5850	0.29	NA	0.11	0.62	6.78	1.44	2.74	0.71
2010	Aligudarz	208	27189	0.46	0.45	0.25	0.75	63.20	1.90	3.33	0.73
2010	Borujerd	209	77602	0.95	0.94	0.69	1.24	76.29	1.87	3.46	0.73
2010	Khorramabad	210	110551	1.07	1.09	0.83	1.35	68.87	1.86	3.42	0.73
2010	Dalfan	211	24989	0.33	0.25	0.17	0.58	42.86	1.93	3.45	0.73
2010	Dorud	212	33257	0.49	0.43	0.28	0.78	66.14	1.92	3.35	0.73
2010	Kuhdasht	213	40772	0.36	0.37	0.2	0.58	51.62	1.82	3.51	0.73
2010	Azna	214	15563	0.57	0.77	0.29	0.98	55.59	1.88	3.43	0.73
2010	Poldokhtar	215	15443	0.73	1.1	0.38	1.22	40.02	1.87	3.39	0.73
2010	Selseleh	216	14831	0.54	NA	0.18	1.26	41.43	1.95	3.37	0.73
2010	Ilam	217	42302	1.31	1.49	0.91	1.81	82.01	1.86	3.28	0.76
2010	Darrehshahr	218	12247	0.31	0.17	0.13	0.62	39.25	1.82	3.37	0.76
2010	Dehloran	219	12247	0.49	0.35	0.22	0.91	65.20	1.80	3.34	0.76
2010	Shirvan va Chardavol	220	14927	0.37	NA	0.15	0.77	24.27	1.88	3.20	0.76
2010	Mehran	221	9737	0.63	0.43	0.29	1.15	61.31	1.91	3.26	0.76

2010	Abdanan	222	9415	0.4	0.22	0.19	0.75	61.12	1.83	3.16	0.76
2010	Eyvan	223	10704	0.75	0.57	0.38	1.33	64.95	1.82	3.23	0.76
2010	Boyerahmad	224	41796	0.76	0.89	0.47	1.11	45.14	1.94	3.25	0.59
2010	Kohgiluyeh	225	41755	0.35	NA	0.14	0.71	44.48	1.96	3.34	0.59
2010	Gachsaran	226	29231	0.67	0.71	0.39	1.04	66.30	1.94	3.17	0.59
2010	Dena	227	10504	0.34	0.19	0.15	0.64	19.81	2.02	3.26	0.59
2010	Bushehr	228	47112	1.66	1.65	1.2	2.2	85.26	2.23	3.75	0.74
2010	Tangestan	229	14205	0.46	0.28	0.22	0.82	23.22	2.15	3.70	0.74
2010	Dashtestan	230	48184	0.4	0.25	0.24	0.62	62.92	2.27	3.62	0.74
2010	Dashti	231	16042	0.5	0.38	0.25	0.87	57.01	2.21	3.67	0.74
2010	Dayyer	232	9936	0.5	0.21	0.24	0.9	68.76	2.28	3.89	0.74
2010	Kangan	233	14505	0.63	0.6	0.32	1.08	68.28	2.31	3.73	0.74
2010	Genaveh	234	18713	1.03	1.11	0.6	1.6	73.78	2.18	3.78	0.74
2010	Deylam	235	6451	1.03	1.62	0.47	1.88	74.87	2.31	3.72	0.74
2010	Jam	236	8314	0.39	0.28	0.16	0.78	37.13	2.25	3.79	0.74
2010	Abhar	237	37562	0.37	0.27	0.2	0.6	65.94	1.39	3.13	0.65
2010	Khodabandeh	238	33943	0.24	0.12	0.12	0.42	27.18	1.36	2.99	0.65
2010	Zanjan	239	105633	0.63	0.6	0.45	0.84	79.42	1.40	3.03	0.65
2010	Ijrud	240	8281	0.32	NA	0.13	0.7	7.23	1.28	3.16	0.65
2010	Khorramdarreh	241	14093	0.65	0.72	0.33	1.1	79.22	1.34	3.16	0.65
2010	Tarom	242	9900	0.35	0.4	0.14	0.71	14.66	1.36	3.14	0.65
2010	Mahneshan	243	8247	0.35	NA	0.12	0.78	16.45	1.26	2.95	0.65
2010	Damghan	244	20003	1.12	1.26	0.67	1.69	69.07	3.14	5.87	0.75
2010	Semnan	245	46083	0.89	0.92	0.59	1.25	88.14	3.17	5.96	0.75
2010	Shahrud	246	53496	1.04	0.99	0.73	1.4	65.26	3.16	5.96	0.75
2010	Garmsar	247	19174	0.71	0.51	0.39	1.13	69.15	3.17	5.91	0.75
2010	Ardakan	248	14440	0.67	0.41	0.36	1.12	82.30	2.60	4.53	0.72
2010	Bafq	249	11540	0.73	0.53	0.38	1.23	66.36	2.61	4.47	0.72
2010	Taft	250	11700	0.58	0.47	0.3	0.99	33.45	2.64	4.34	0.72
2010	Mehriz	251	9690	0.75	0.2	0.38	1.29	57.78	2.64	4.36	0.72
2010	Yazd	252	115410	1.96	1.96	1.63	2.33	95.31	2.54	4.42	0.72
2010	Meybod	253	15425	0.71	0.39	0.39	1.18	90.00	2.55	4.29	0.72
2010	Abarkuh	254	10036	0.72	0.4	0.37	1.24	63.48	2.56	4.41	0.72
2010	Sadugh	255	6450	0.87	0.91	0.38	1.66	53.35	2.51	4.20	0.72
2010	Khatam	256	6951	0.67	0.58	0.29	1.27	57.28	2.55	4.32	0.72
2010	Tabas	257	13916	0.78	0.86	0.41	1.3	56.68	2.57	4.37	0.72
2010	Abumusa	258	293	1.07	NA	0.4	2.4	98.67	0.48	2.96	0.51
2010	Bandarabbas	259	113604	0.7	0.67	0.5	0.92	69.46	0.45	2.97	0.51
2010	Bandar-e Lengeh	260	29096	0.7	0.96	0.42	1.07	48.09	0.42	3.04	0.51
2010	Qeshm	261	18560	0.39	0.12	0.19	0.68	40.18	0.54	2.87	0.51
2010	Minab	262	47785	0.39	0.39	0.23	0.61	26.04	0.50	3.10	0.51
2010	Bandar-e-Jask	263	13190	0.29	0.16	0.15	0.51	16.05	0.36	3.01	0.51
2010	Rudan	264	20543	0.35	0.51	0.17	0.62	34.32	0.41	3.06	0.51
2010	Hajiabad	265	13216	0.29	0.15	0.13	0.57	32.49	0.50	3.05	0.51
2010	Bastak	266	13295	0.7	1.37	0.35	1.19	22.51	0.36	2.93	0.51
2010	Tehran	267	2148875	1.84	1.84	1.76	1.92	99.61	2.93	4.96	0.84
2010	Damavand	268	21941	0.94	0.91	0.55	1.47	72.60	2.84	4.96	0.84

2010	Rey	269	59120	1.31	1.3	0.96	1.72	27.46	2.89	5.19	0.84
2010	Shemiranat	270	10661	1.9	2.15	1.12	2.91	53.32	2.87	5.02	0.84
2010	Varamin	271	113995	0.66	0.62	0.48	0.86	80.76	2.84	4.97	0.84
2010	Shahriyar	272	242752	0.68	0.69	0.54	0.83	86.41	2.88	4.88	0.84
2010	Eslamshahr	273	99197	0.64	0.67	0.46	0.86	89.69	2.93	4.90	0.84
2010	Robatkarim	274	127686	0.56	0.64	0.4	0.75	80.73	2.95	5.00	0.84
2010	Pakdasht	275	50351	0.51	0.57	0.31	0.77	77.18	2.78	4.92	0.84
2010	Firuzkuh	276	9004	0.51	NA	0.2	1.08	40.79	2.86	4.94	0.84
2010	Ardebil	277	122227	0.72	0.76	0.53	0.94	83.34	1.60	2.90	0.74
2010	Bilehsowar	278	11530	0.37	0.35	0.16	0.71	38.76	1.71	2.87	0.74
2010	Khalkhal	279	21397	0.62	NA	0.22	1.41	47.31	1.67	2.84	0.74
2010	Meshginshahr	280	33329	0.63	0.6	0.37	0.95	43.56	1.67	2.95	0.74
2010	Germi	281	18669	0.41	0.43	0.2	0.71	32.38	1.71	2.88	0.74
2010	Parsabad	282	34344	0.34	0.31	0.18	0.57	55.48	1.81	3.01	0.74
2010	Kowsar	283	5922	0.38	NA	0.11	0.95	25.28	1.70	2.88	0.74
2010	Namin	284	13036	0.35	0.15	0.16	0.65	35.67	1.72	3.11	0.74
2010	Neer	285	5480	0.37	NA	0.13	0.83	24.60	1.67	2.91	0.74
2010	Qom	286	228447	0.62	0.6	0.49	0.77	93.67	3.02	4.32	0.73
2010	Bueenzahra	287	34406	0.28	0.24	0.14	0.48	34.96	2.28	3.69	0.83
2010	Takestan	288	36182	0.5	0.4	0.29	0.78	60.10	2.30	3.66	0.83
2010	Qazvin	289	167493	0.7	0.66	0.54	0.88	80.37	2.29	3.75	0.83
2010	Abyek	290	19203	0.48	0.32	0.25	0.82	54.87	2.25	3.74	0.83
2010	Bandare Gaz	291	11620	0.82	1	0.44	1.36	53.65	1.89	3.46	0.74
2010	Torkman	292	28155	0.51	0.43	0.29	0.82	59.82	1.88	3.42	0.74
2010	Aliabad	293	27776	0.73	0.81	0.43	1.13	48.15	1.88	3.33	0.74
2010	Kordkuy	294	16819	1.01	1.38	0.58	1.58	50.92	1.83	3.32	0.74
2010	Gorgan	295	99612	1.27	1.29	0.99	1.58	73.11	1.93	3.41	0.74
2010	Gonbade Kavus	296	65136	0.47	0.38	0.3	0.69	49.25	1.89	3.43	0.74
2010	Minudasht	297	27344	0.44	0.45	0.23	0.72	34.29	1.81	3.44	0.74
2010	Aqqala	298	23445	0.28	0.18	0.13	0.51	29.76	1.86	3.35	0.74
2010	Kalaleh	299	30246	0.34	0.35	0.17	0.57	22.49	1.86	3.53	0.74
2010	Azadshahr	300	18916	0.75	1.08	0.41	1.22	54.56	1.86	3.36	0.74
2010	Ramyan	301	16998	0.27	0.12	0.12	0.5	40.10	1.85	3.40	0.74
2010	Esfarayen	302	27918	0.31	0.29	0.16	0.53	41.99	1.16	3.30	0.64
2010	Bojnurd	303	68228	0.53	0.54	0.35	0.77	58.42	1.25	3.31	0.64
2010	Jajarm	304	12446	0.3	0.16	0.12	0.59	62.59	1.15	3.41	0.64
2010	Shirvan	305	33880	0.52	0.64	0.3	0.81	52.19	1.16	3.24	0.64
2010	Faruj	306	11868	0.25	0.16	0.11	0.5	25.16	1.21	3.32	0.64
2010	Maneh va Semelqan	307	19078	0.33	0.22	0.15	0.62	24.69	1.24	3.43	0.64
2010	Birjand	308	59801	0.88	0.89	0.61	1.2	57.58	0.71	3.31	0.50
2010	Sarbisheh	309	10705	0.44	NA	0.13	1.13	23.89	0.74	3.11	0.50
2010	Qaenat	310	30500	0.41	0.46	0.22	0.67	34.73	1.58	3.39	0.50
2010	Nehbandan	311	10544	0.34	NA	0.11	0.82	28.69	0.74	3.18	0.50
2010	Sarayan	312	7472	0.55	NA	0.2	1.2	61.29	0.70	3.09	0.50
2010	Ferdows	313	15298	0.44	0.25	0.22	0.77	67.99	1.69	3.51	0.50
2010	Karaj	314	449096	0.77	0.79	0.66	0.89	96.94	2.80	4.90	0.47
2010	Savojbolagh	315	51998	0.11	0.04	0.05	0.2	38.29	2.92	4.94	0.47

2010	Nazarabad	316	28289	0.31	0.15	0.15	0.55	78.35	2.86	4.99	0.47
